# Study of optical rotation based on the molecular structure in fused oligomers of macrocycles†

**DOI:** 10.1039/d4ra03709j

**Published:** 2024-07-01

**Authors:** Ryo Katoono, Yudai Obara, Kazuki Sakamoto, Rei Miyashita

**Affiliations:** a Department of Chemistry, Faculty of Science, Hokkaido University Sapporo 060-0810 Japan katoono@sci.hokudai.ac.jp +81 11 706-4616

## Abstract

We designed a unique oligomer form in which several helically twisted macrocycles (*M*- or *P*-helicity) are arranged through fusion. We investigated the optical rotation of a series of fused oligomers of macrocycles with a difference in the number and arrangement of elements associated with point-chiral auxiliary. Some oligomers cooperatively attained a situation where an identical sense of twisting was preferred throughout the entire molecule. On the basis of these results, we estimated diastereomeric excess induced in each oligomer. We revealed that the molar optical rotation per element was modulated with a rotational angle between elements: an increase *via* 0° rotational arrangement, a decrease *via* 180° rotational arrangement, or a decrease *via* cyclic arrangement. Alternatively, for other oligomers in which several diastereomeric conformers coexist, we uniquely attempted to consider the optical rotation based on the molecular structure through the assessment of a change ratio of the absorption dissymmetry factor before and after complexation with an achiral guest. We found that molar optical rotation could be different based on the arrangement, although actual measured values were similar.

## Abstract 2

The fusion of macrocyclic elements, which adopt twisted forms with *M*- or *P*-helicity, provides a variety of chiral architectures. The correlation between optical rotation and the molecular structure is discussed.

## Graphical abstract

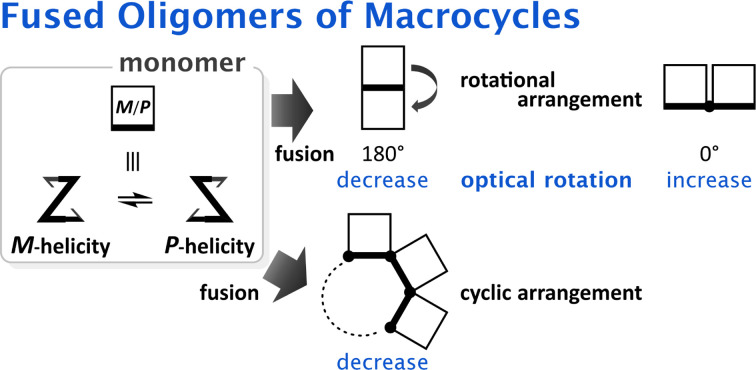

## Introduction

**Scheme 1:**
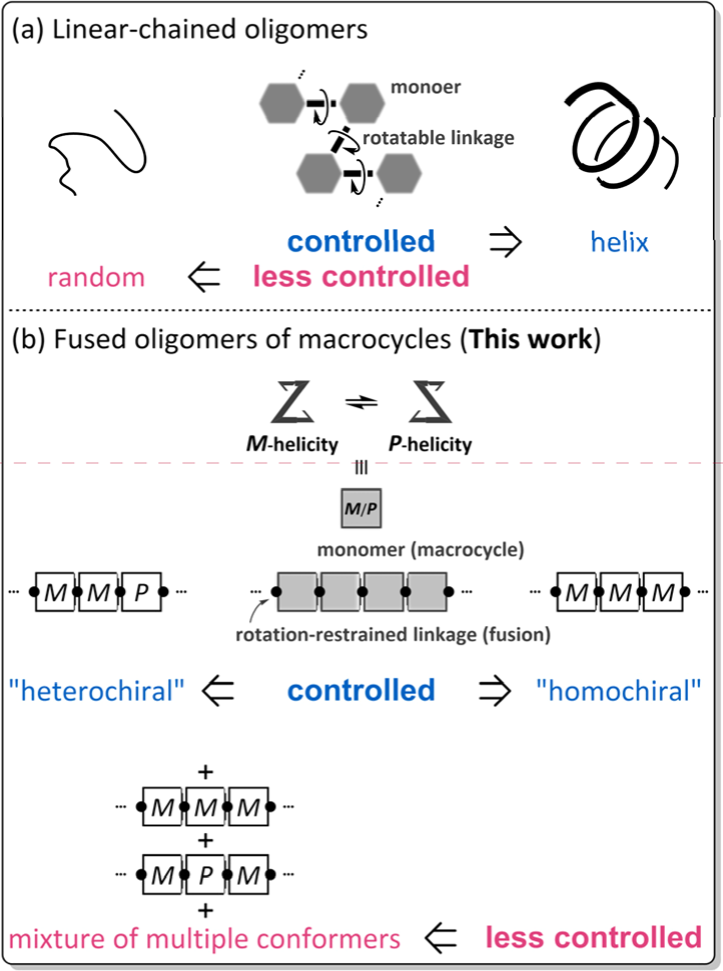
Types of oligomers: (a) linear-chained oligomers and (b) fused oligomers of macrocycles.

The term oligomer refers to a molecule composed of multiple repeats of a small molecule (monomer). A typical case is known as so-called linear-chained oligomers (Scheme 1a),^1–4^ in which the structure of a small molecule is invariant and a rotatable bond is used to link the monomers. The conformational control of such a linear-chained oligomer is attained by an accumulative result of multiple controls of local conformation (*e.g.*, dihedral angle) about the rotatable linkage since the monomer is invariant. The term can cover another form of oligomers;^5–8^ for example, in which the monomer is a macrocycle arranged in a rotation-restrained manner.^6–8^ We are particularly interested in a unique case in which the macrocyclic monomer is variant in terms of chirality^9–13^ (Scheme 1b). The conformational control of such a fused oligomer of macrocycles would be attained by an accumulative result of multiple controls of local conformation (*e.g.*, dynamic chiral sense of twisting) in each macrocyclic element since the linkage is invariant. In such fused oligomers of macrocycles, there are cases where all elements prefer the same sense throughout the entire molecule (for descriptive purposes, we call this a “homochiral” situation)^10–12^ or different senses coexist (“heterochiral” situations)^13^ in a molecule. Such conformational interinfluence could be a driving force for a molecule to predominantly adopt a particular conformation among multiple conformations.^14,15^ It should be noted that conformational interinfluence can lead to a homochiral situation and sometimes a specific heterochiral situation (Scheme 1b, controlled). If each macrocyclic element has no preference for a particular sense of twisting and no interinfluence could work between elements, multiple conformers with an identical and different sense of twisting would be statistically populated in the solution (Scheme 1b, less controlled).

**Scheme 2:**
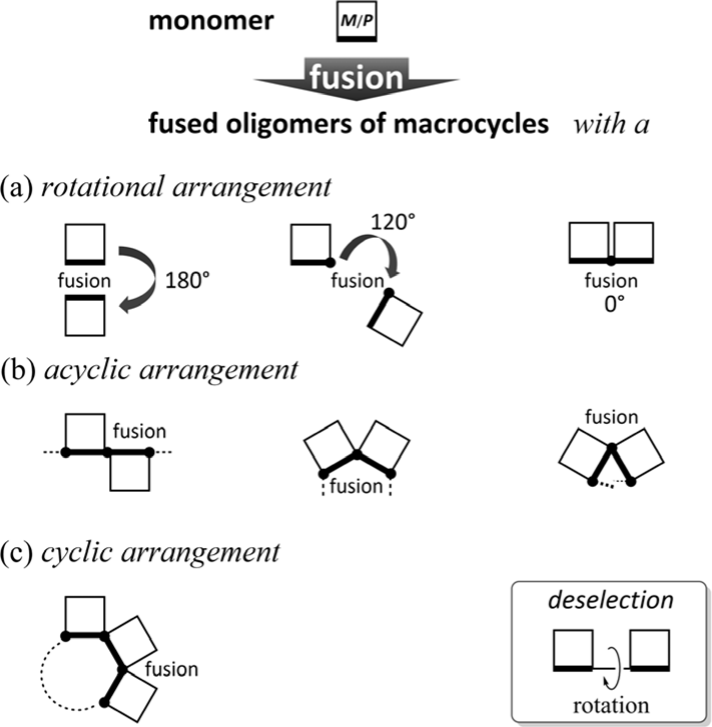
Diverse arrangements of dynamic chiral macrocyclic elements: (a) rotational, (b) acyclic, and (c) cyclic.

We assumed diverse arrangements of elements: rotational, acyclic, and cyclic (Scheme 2), which present diverse chiral architectures. This point would be an advantage of this oligomer system for studying optical rotation based on the molecular structure. This work investigated the molar optical rotation per element ([*M*]_D_/*N*, *N*: number of macrocyclic elements) of a series of fused oligomers with a difference in the number and arrangement of elements. Attention should be paid to the fact that the correlation between the chiroptical values of a monomer and its oligomers is not settled on solid ground. The chiroptical value would be affected by molecular size, shape (number and arrangement of chromophores), and diastereomeric ratios. Therefore, discussing the chiroptical values would not be valid based solely on how an actual measured value is larger or smaller than a monomer. We should pay more attention to both minor and the most major conformers for dynamic chiral molecules, especially in less controlled cases (Scheme 1b).

**Fig. 1:**
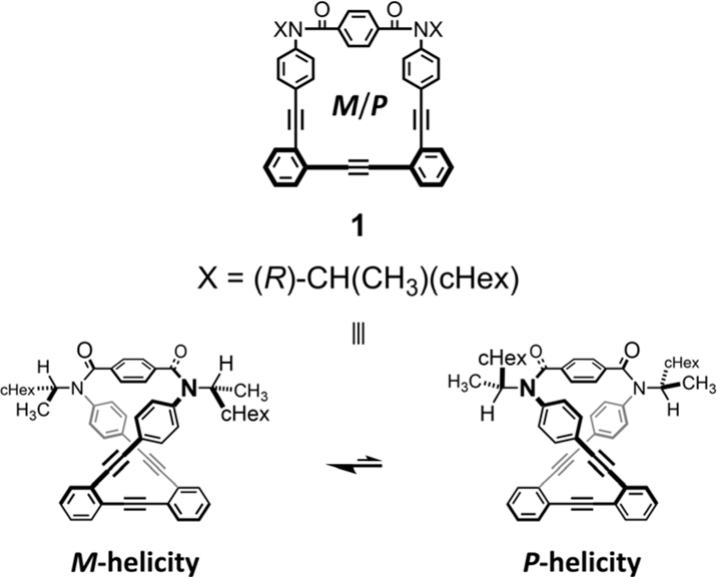
Chemical structure of chiral macrocyclic element 1^9^ (*N* = 1) and conformational interconversion between two chiral forms with *M*- or *P*-helicity.

**Fig. 2:**
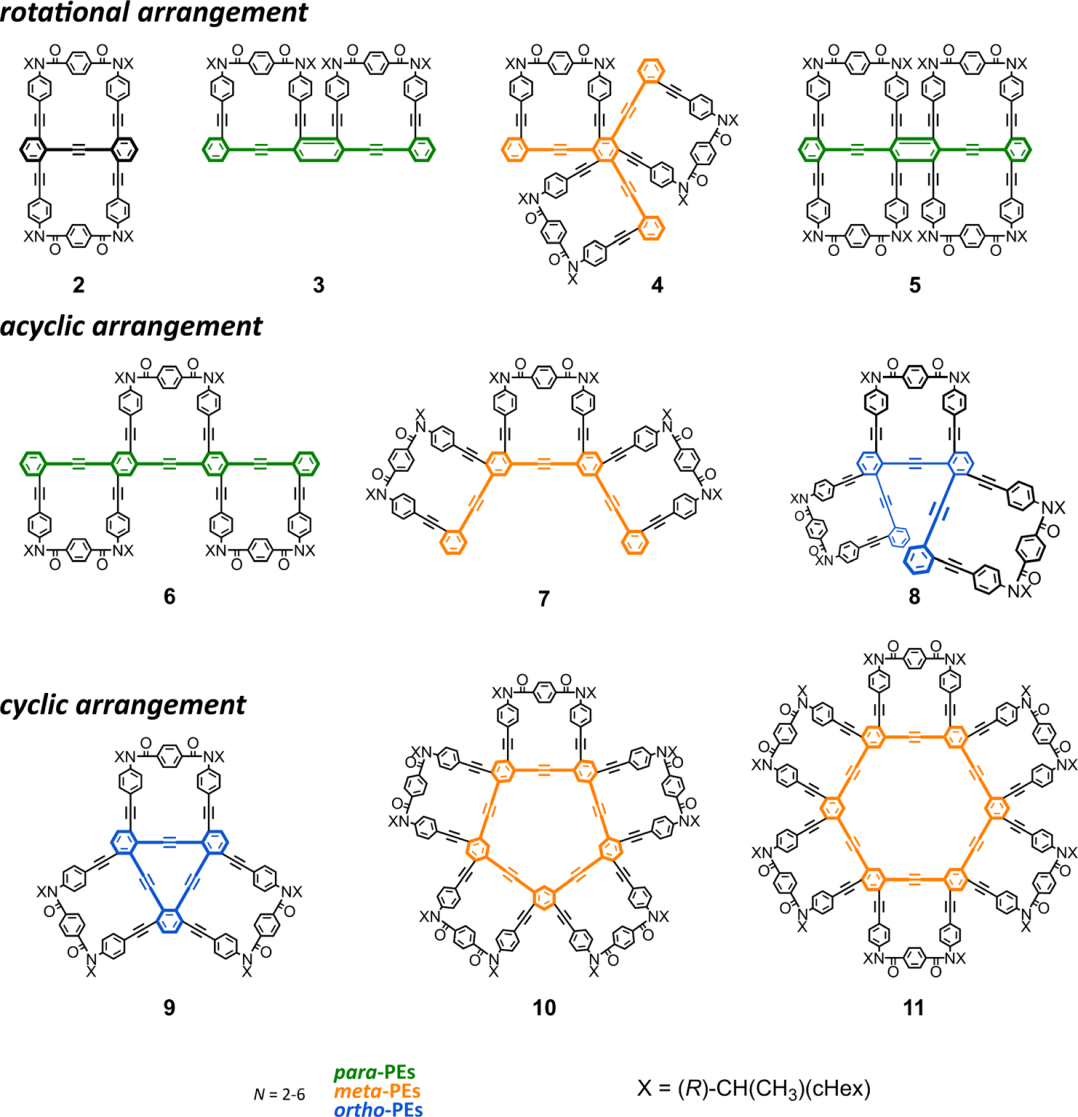
Chemical structures of fused oligomers of macrocycles 2–11 (*N* = 2–6). PE: phenylene-ethynylene. Bis-, tris- or tetrakis(macrocycle) 3, 5, 6, and 8 were newly synthesized.

As a monomer of fused oligomers, we used a macrocycle (1)^9^ that can adopt two chiral forms with *P*- or *M*-helicity by twisting clockwise or counterclockwise (Fig. 1). As previously reported, these two helical forms are conformationally interconvertible in solution. The population can be estimated using a diastereomeric ratio by NMR spectroscopy. Thus, we arranged the macrocyclic element covalently in a single molecule through ring fusion of a part of the element (2–11) (Fig. 2). These fused oligomers would exist as a mixture of multiple conformers in solution according to the helical sense of each element (*e.g.*, *M*_2_, *M*_1_*P*_1_, and *P*_2_ from two elements; *M*_3_, *M*_2_*P*_1_, *M*_1_*P*_2_, and *P*_3_ from three elements, *etc.*). If we could determine the population of multiple conformers that coexist in a solution, a measured value would be interpreted according to the population. However, it is not always easy when the number of elements increases.

If only a single pair of diastereomeric (pseudo-enantiomeric) conformers with *M*- or *P*-helicity could be present in the solution (Scheme 1b, controlled), that would be suitable for estimating the population as a diastereomeric ratio (*M*_*N*_/*P*_*N*_), as with a monomeric case (*N* = 1). This was the case for 2,^10^4,^11^5, 10,^12^ and 11.^12^ Fortunately, the diastereomeric ratio was quantitatively estimated for 2, 4, and 5. This allowed us to discuss their optical rotations based on their molecular structure and found that the molar optical rotation could be modulated at a rotational angle between elements.

Alternatively, multiple pairs of diastereomers can be present in equilibrium (3 and 6–8^12^) (Scheme 1b, less controlled). In such a solution, chiroptical values would be obtained as an average property resulting from contributions from all conformations, for which it is impossible to consider as-measured values based solely on a particular conformation. Here, we uniquely attempted to evaluate optical rotations emerging from even such an indefinite system due to the coexistence of multiple conformers, suggesting that a rotational angle between elements could contribute to a difference in the molar optical rotation of tris(macrocycle)s 6–8.

Conformational analyses of the fused oligomers of macrocycles in solution were primarily based on NMR spectroscopy, and we measured optical rotation^5,16–22^ and electronic circular dichroism (CD).^5,16^

## Results and discussion

### Synthesis of fused oligomers of macrocycles

**Scheme 3:**
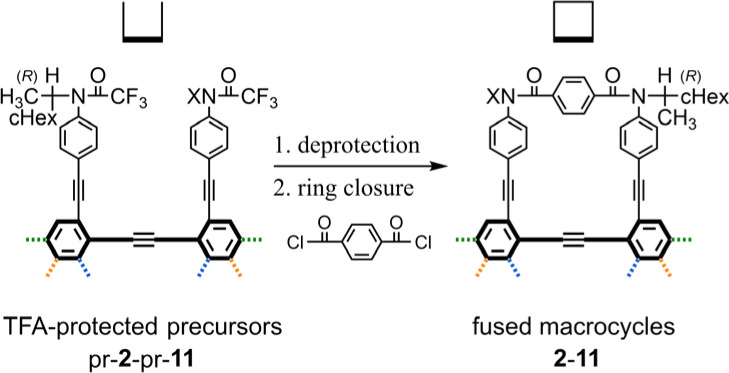
Synthetic strategy based on a multi-fold ring closure.

We synthesized a series of fused oligomers of macrocycles 2–11 (Fig. 2) based on a simple and versatile strategy that involved a multi-fold ring-closing condensation reaction of a couple of anilines with terephthaloyl chloride (Scheme 3). The precursor anilines were obtained in a TFA-protected form, which was used as a control of the corresponding macrocycle in optical rotation measurements (pr-1-pr-11). Bis-, tris- or tetrakis(macrocycle) 3, 5, 6, and 8 were newly synthesized here (ESI_1, Schemes S1–S3) (ESI_2)† and analyzed along with other analogs.

### ^1^H NMR spectra of fused oligomers of macrocycles

To consider an innate conformational preference (homochiral or heterochiral) in each framework without any chiral auxiliary, we implemented conformational searches with model molecules 2M–11M [X = CH_3_] using software summarized in the ESI (ESI_1, Fig. S1–S6).† In solution, conformational preferences of 2–11 with chiral auxiliaries [X = (*R*)-CH(CH_3_)(cHex)] would be modulated by cooperation or competition between such an innate preference of the framework and accumulative helical-sense preferences induced in each macrocyclic element by the intramolecular transmission of point chirality (*R*).

**Fig. 3:**
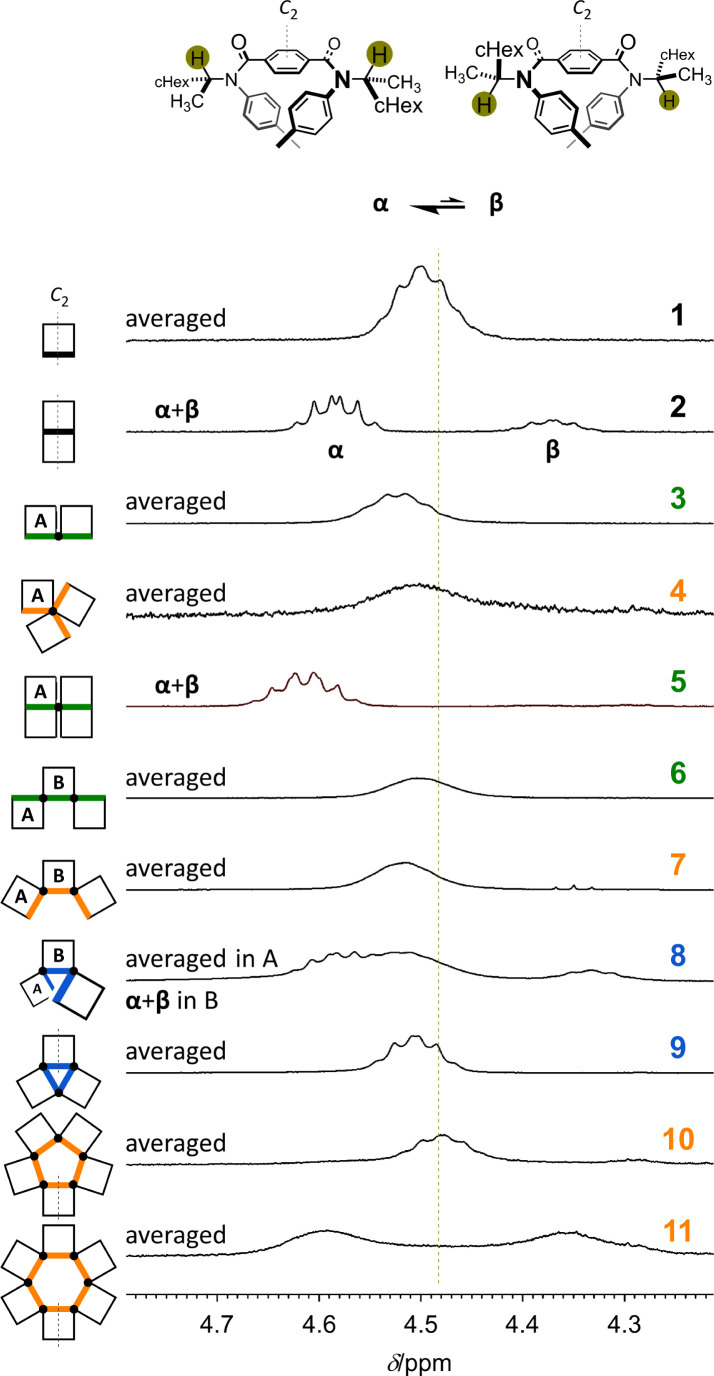
Partial ^1^H NMR spectra (400 MHz, methine protons) of 1–11, measured in CDCl_3_ at room temperature. The lack of a two-fold axis led to non-equivalency within a macrocyclic element (A in 3–8) and non-equivalency between elements (A ≠ B, ABA in 6–8).

NMR spectroscopy would be an effective tool for estimating a ratio of conformers when both major and minor peaks could stand separately within an NMR timescale. The ^1^H NMR spectrum of 1, measured at room temperature, showed only one set of averaged resonances, indicating that two twisted forms (*M*-1 and *P*-1) interconverted within the timescale (Fig. 3, S7, and S8†). Alternatively, in the spectrum of 2 (Fig. 3, S7, and S9†), two sets of resonances (α + β) were present and assigned to diastereomeric conformers of *MM*-2 (α) and *PP*-2 (β). These independent chemical shifts for methine protons (Hα: 4.588 ppm and Hβ: 4.375 ppm)^23^ were revealed to assume a central value. According to this value, we found an averaged signal for the corresponding protons in 1 that appeared downfield. This was also the case for other fused macrocycles 3, 4, 6, 7, and 9, indicative of fast interconversions among conformers. While for 5 (Fig. 3, S7, S10, and S11†) and partly for 8 (Fig. 3, S7, S12, and S13†), such interconversion slowed, such as in 2. Though it could not be assigned to a particular (homochiral or heterochiral) conformation of 10 or 11, the coexistence of two forms with *M*- or *P*-helicity (in a conformer or among conformers) was confirmed at room temperature. Based on chemical exchanges in ^1^H NMR spectroscopy (Fig. S14†), we reported that the diastereomeric ratio (α/β) induced at 223 K in chloroform was estimated to be 2.3 for *M*-1 and *P*-1, 4.7 for *MM*-2 and *PP*-2, and 9.9 for *MMM*-4 and *PPP*-4 by linear extrapolation.^11^ In a similar manner, the value of α/β was estimated to be *ca.* 51 for *MMMM*-5 and *PPPP*-5 (Fig. S10 and Table S1†).

Alternatively, in the VT-NMR spectra of bis(macrocycle) 3 (Fig. S15 and S16†), lowering temperatures revealed that a heterochiral conformer (*MP*), as well as a diastereomeric pair of homochiral conformers (*MM* and *PP*), was involved in the equilibrium (Fig. S14†). Participation of heterochiral conformer(s) in the equilibrium was similarly observed in the spectra of tris(macrocycle)s 6–8 with an acyclic arrangement (Fig. S12, S17 and S18†), where the conformational distribution was indeterminate due to broadening and overlapping.

### Molar optical rotation of fused oligomers of macrocycles

For dynamic chiral molecules,^24–27^ optical activities vary with the ratio of two enantiomeric forms. A purely isolated enantiomer obtained the greatest and specific value, and the value disappears in a racemate. To disrupt the equivalent balance, a point-chiral auxiliary [X = (*R*)-CH(CH_3_)(cHex)] was attached to the macrocyclic element. We can see an optical activity through the intramolecular transmission of the point chirality (*R*) to the macrocyclic element (*M*/*P*).

**Fig. 4:**
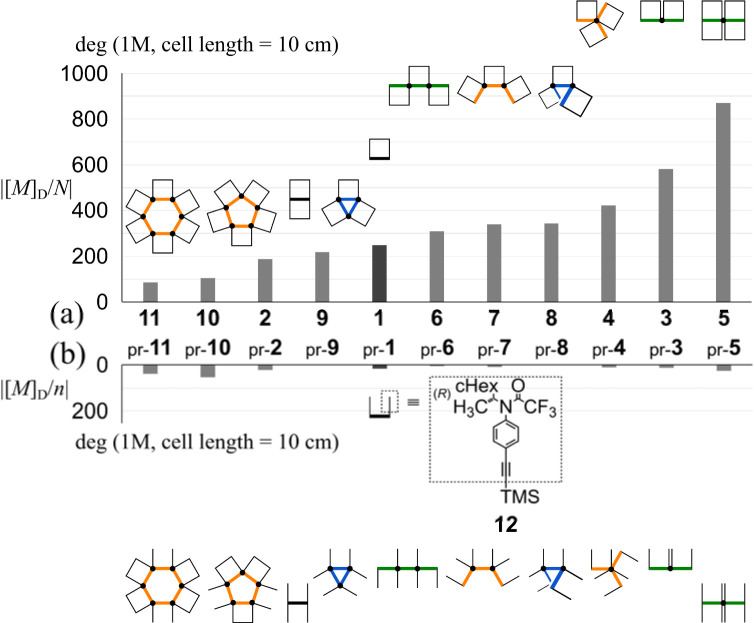
Chemical structure of 12^28^ and absolute values of [*M*]_D_ per element (a) *N*: number of macrocyclic elements and (b) *n*: number of point-chiral elements.

Although the monomeric element 1 (*N* = 1) and its acyclic analog pr-1 possess the same number of auxiliaries (*n* = 2), the value of the molar optical rotation [*M*]_D_ of 1 was significantly greater than that of pr-1 (Fig. 4). This single-digit increase would be due to an imbalance induced between the two helically-twisted forms of the macrocycle since similar values of [*M*]_D_/*n* were recorded with pr-1 (*n* = 2) and 12^28^ (*n* = 1) without any macrocyclic structure (Table 1). The signs of the optical rotations induced for fused macrocycles 2–11 were consistent with that of 1,^29^ although there were differences in the number and arrangement of elements. This result was considered consistent with the observation of downfield shifts for methine protons with respect to the central value of Hα and Hβ in the ^1^H NMR spectrum of 2 (Fig. 3).

**Table 1:** Specific optical rotations ([*α*]_D_ [deg], *c* [g dL^−1^]) and molar optical rotations ([*M*]_D_ [deg], 1 M, cell length = 10 cm) measured in CHCl_3_ at room temperature and absolute values of [*M*]_D_ per element (*N*: number of macrocyclic elements and *n*: number of point-chiral elements)

Molecule		11	10	2	9	1	6	7	8	4	3	5
[*α*]_D_		−126	−153	−280	−318	−328	−437	−482	−485	−596	−809	−1338
[*M*]_D_		−513	−519	−375	−650	−249	−928	−023	−029	−263	−165	−479
|[*M*]_D_/*N*|		85	104	188	217	249	309	341	343	421	582	870
Molecule	12	pr-11^a^	pr-10^a^	pr-2	pr-9	pr-1	pr-6	pr-7	pr-8	pr-4	pr-3	pr-5
[*α*]_D_	−35	−108	−149	−59	−3.2	−39	−13	−23	+3.7	−27	−33	−72
[*M*]_D_	−14	−454	−526	−86	−7.2	−32	−29	−53	+8.5	−63	−51	−206
|[*M*]_D_/*n*|	14	38	53	22	1.2	16	4.8	8.8	1.4	10	13	26

a Due to a difference in the synthetic strategy, three (out of five on 5PAM) and four (out of six on 6PAM) macrocycles were present in pr-10 and pr-11. Consequently, the values of [*M*]_D_/*n* were greater than those of pr-2-pr-9.

The intensity of the optical rotation was considered as a value per element. The absolute values of [*M*]_D_/*N* increased for 6–8 (acyclic) and 3–5 (rotational), and decreased for 2 (rotational), 9–11 (cyclic), compared to that for 1 (upper, Fig. 4 and Table 1). These values should be considered an average property; therefore, a comparison in the intensity was allowed only for 1, 2, 4, and 5. Based on the measured values of [*M*]_D_ and each diastereomeric excess at room temperature, the maximum value of [*M*]_D_/*N* at 100 de% was calculated to be 1311 deg for 1 (from 19 de% at room temperature), 552.9 for 2 (34 de%), 1138 for 4 (37 de%), and 1359 for 5 (64 de%). In comparing 1 and 2, a molecule that can be regarded as a doubled form, where a pair of motifs are arranged so that they are rotated 180° with respect to each other, could contribute to a decrease in intensity, at least in the current system. The comparison of 2 and 5 would support that a rotational arrangement with 0° could contribute to an increase in intensity (*Cf.*1 and 3). Relatively small values of [*M*]_D_/*N* were obtained for 9–11 with a cyclic arrangement, indicating that the cyclic arrangement could be a reason for the decrease^21^ or canceled senses of twisting in a heterochiral conformation might lead to the decrease.

For a series of the corresponding acyclic analogs (pr-2-pr-9) (lower, Fig. 4 and Table 1), we observed similar values of [*M*]_D_/*n*, which were comparable to the value for 12 (*n* = 1), regardless of the *n* and PE frameworks. These results indicated that the value was considered an additive property and the point-chiral element could work without regard to the location,^23^ as it was associated with 12, due to the high conformational flexibility of acyclic frameworks.^20^

**Scheme 4:**
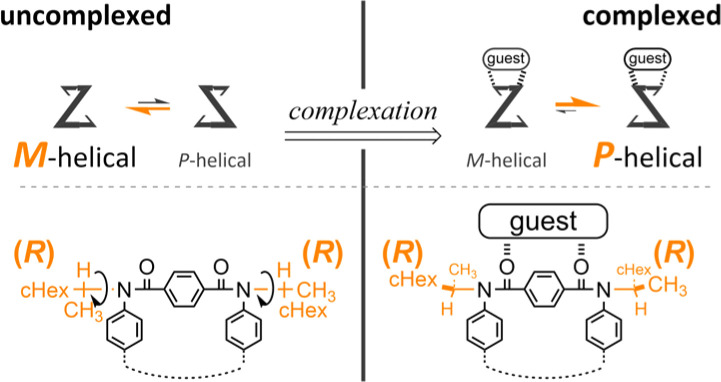
Complexation-induced reversal of helical-sense preferences (two-way intramolecular transmission of point chirality).

**Scheme 5:**
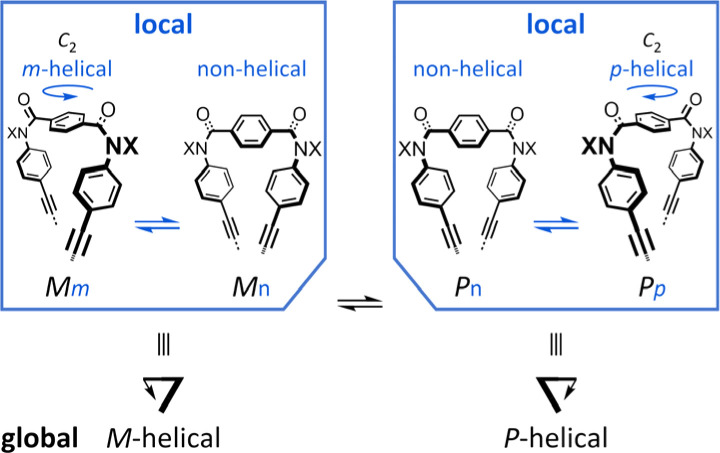
Multiple conformations in the global helicity (*M*- and *P*-helical) and local helicity (non-, *m*- and *p*-helical).

Attempt to consider optical rotation by assessment of a change ratio of dissymmetry (Δ*ε*/*ε*) before and after complexation. Here, we attempt to consider the optical rotations of bis- or tris(macrocycle) 3 and 6–9, for which multiple conformers coexist in solution (populations unknown), as shown by ^1^H NMR spectra measured at low temperatures. For such an indefinite case, we conceived using a guest molecule to form a complex. We planned to assess a change ratio of the absorption dissymmetry factor (Δ*ε*/*ε*) before and after complexation. This was inspired by and based on the complexation-induced reversal of helical-sense preferences (two-way intramolecular transmission of point chirality) (Scheme 4):^9,10^ (i) in the absence of a guest, an *M*-helical form is preferred due to an intramolecular transmission of point chirality (*R*), and (ii) in the presence of a guest, a *P*-helical form turns to be favorable due to an intramolecular transmission of the same point chirality (*R*). To be strict, we need to consider multiple conformations in terms of the local helicity (*m*, *n*, or *p*-helicity) as well as global helicity (*M*- or *P*-helicity) (Scheme 5) since a helical-sense preference would be related to an energy difference between two local *C*_2_-symmetrical conformations with *m*- or *p*-helicity (*Mm* and *Pp*).

The proximity of a guest through the formation of hydrogen bonds at the terephthaloyl bridge would lead to some change in the local conformation (*e.g.*, the dihedral angle of the carbonyl group with respect to the central phenylene ring in the terephthaloyl bridge and the spatial location in the chiral auxiliary). That is, the population of local non-helical conformers (*M*_*n*_ and *P*_*n*_) would be reduced in a complex state. Consequently, an intramolecular point-chirality transmission would work relatively more efficiently than an uncomplexed state.

**Scheme 6:**
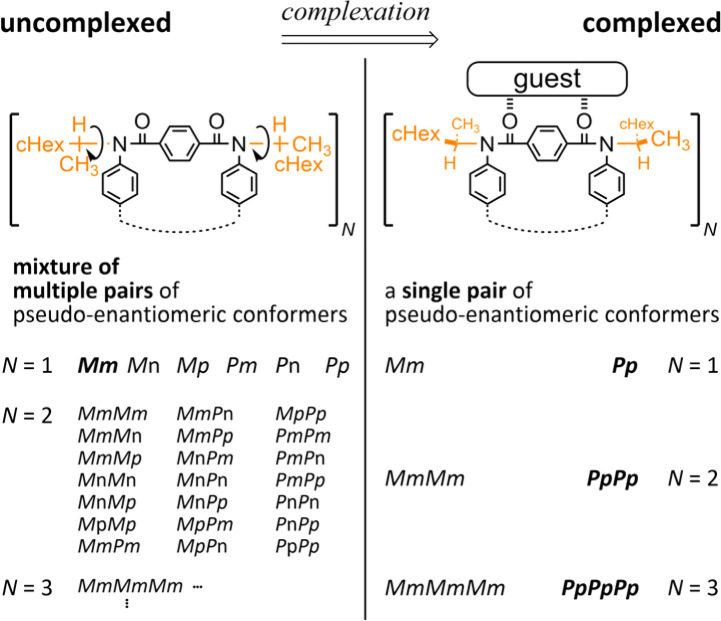
Complexation-induced change in the population from multiple to single pair of conformers.

Thus, we assumed that such a homochiral situation could be enforcedly preferred in a complex state of fused oligomers even in any number and arrangement of elements (Scheme 6), which might help simplify an indefinite system. In some cases, there would be no change in the preferred forms before and after complexation (2), or complexation could accompany a change in the population of conformers from multiple pairs to a single pair of diastereomers (3, 6–9). In the former case, the change ratio (complexed/uncomplexed) would be close to 1 if a macrocyclic element did not significantly change local conformations upon complexation. In the latter case, the change ratio would be relatively more excellent since the chiroptical values obtained from an original solution (in the absence of a guest) could be underestimated due to the coexistence of multiple conformers containing heterochiral ones.

To consider the original state, we first implemented VT-CD measurements of 3, 6–9 without a guest (Fig. S19A†). In the absorption region of each fused macrocycle (Fig. S20†), the Cotton effects involving several extreme values were induced through an intramolecular transmission of point chirality in each element. The general appearance of the CD spectra of 3, 6, 8, and 9 was similar to that of 1 or 2, minus-plus-minus from the longer-wavelength region, while the spectrum of 7 seemed a little more intricate. The intensities of these Cotton effects changed with temperature, while the appearance was almost maintained by showing several isosbestic points. The absorption slightly changed with temperature (Fig. S19B†). These results showed that varying temperatures changed a diastereomeric ratio in each element while maintaining global forms.

Next, we used an achiral guest (13) that can be captured by the terephthaloyl bridge in a 1 : 1 ratio to minimize the contributions from multiple conformations (Scheme 6). Based on the preliminary results of complexation experiments (Fig. S21–S24†), 2, 4, and 8 equivalents or 3, 6, and 9 equivalents of 13 were added to a solution of bis(macrocycle) 3 or tris(macrocycle)s 6–9 to form a 1 : 2 or 1 : 3 complex in dichloromethane at room temperature.

**Fig. 5:**
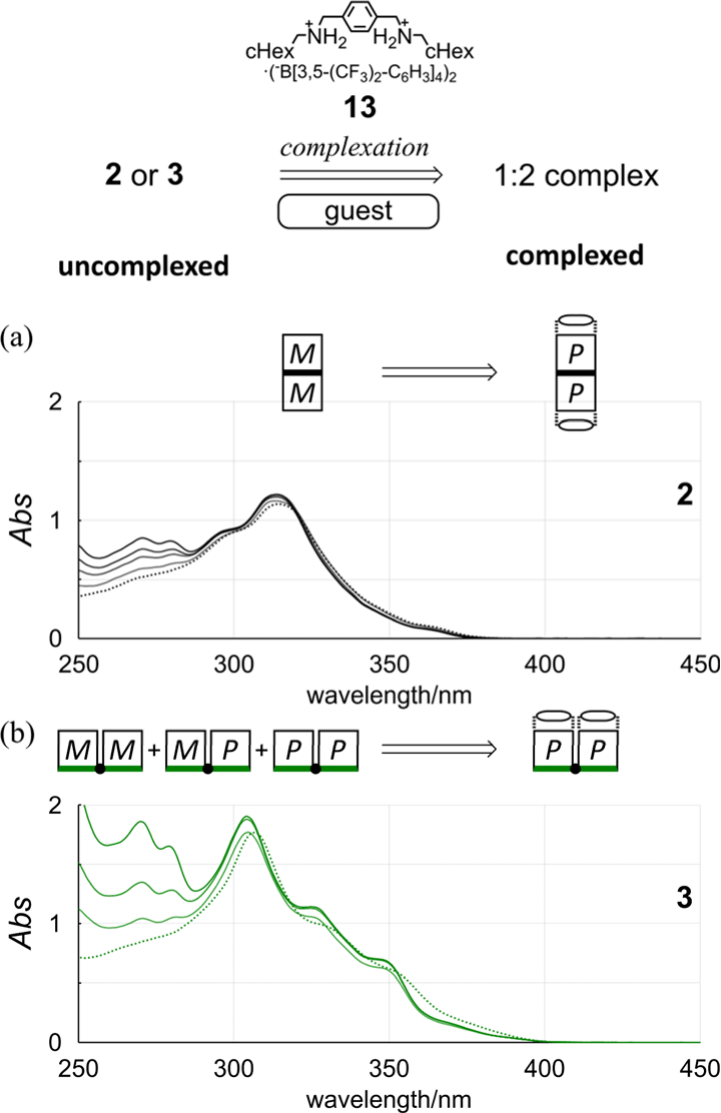
Chemical structure of 13 and UV spectra of (a) 2 (8.37 × 10^−5^ M) and (b) 3 (1.23 × 10^−4^ M) in the presence of 13 [0 equiv. (2 or 3 only, dashed line); 1–4 equiv. for 2; 2, 4 and 8 equiv. for 3]. All spectra were measured in CH_2_Cl_2_ at room temperature. Cell length = 0.1 cm.

**Fig. 6:**
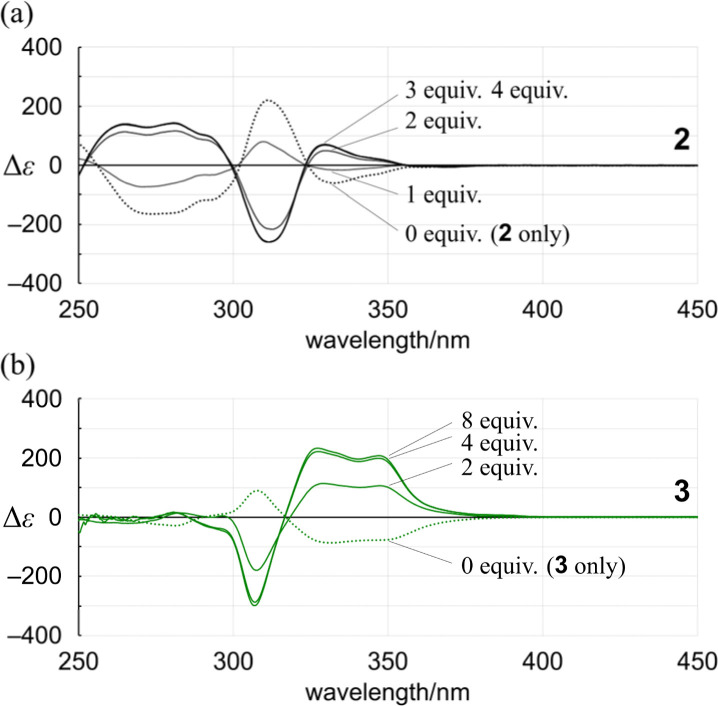
CD spectra of (a) 2 (8.37 × 10^−5^ M) and (b) 3 (1.23 × 10^−4^ M) in the presence of 13 [0 equiv. (2 or 3 only, dashed line); 1, 2, 3 and 4 equiv. for 2; 2, 4 and 8 equiv. for 3]. All spectra were measured in CH_2_Cl_2_ at 293 K. Δ*ε* [L mol^−1^·cm^−1^].

In the UV spectra of 1 and 2 in the presence of 13 (Fig. 5a and S25†), there was only a slight decrease in absorption in the longer-wavelength region induced by complexation. Since only a single pair of twisted forms (for 1) or homochiral forms (for 2) was present in the solution before and after complexation (Fig. S1†), these complexation-induced slight decreases in absorption were considered to be due to a subtle change in local conformation (*e.g.*, degree of distortion about *p*-phenylenes). In the CD spectra of 1 and 2 (Fig. 6a and S26†), pseudo-mirrored Cotton effects emerged, accompanied by the reversal of helical-sense preferences (Scheme 4).

**Fig. 7:**
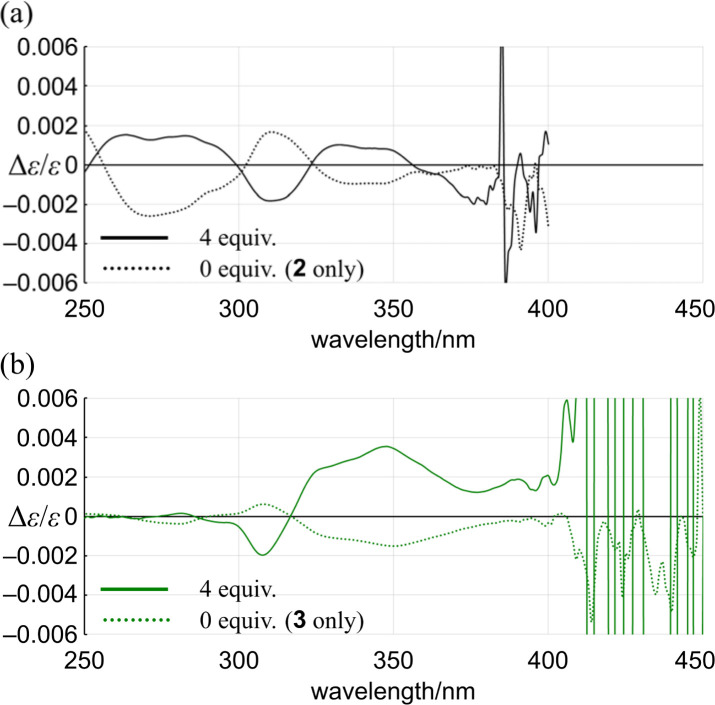
Plots of Δ*ε*/*ε* for (a) 2 and (b) 3*versus* wavelength in the presence (solid line) or absence (dashed line) of 13.

Alternatively, we found that noticeable changes in absorption were induced for bis- (3) and tris(macrocycle)s (6–9) (Fig. 5b and S25†),^30^ which showed a change in the population of conformers before and after complexation. In these solutions, a local reversal or sustention of the helical-sense preference by each element could be included, which leads to a change in the global form, such as *MP* to *PP* (*N* = 2) or *MMP* to *PPP* (*N* = 3) upon complexation (Scheme 6). During a change in the Cotton effects, the lack of an isosbestic point indicated that several species with a difference in the global form were in equilibrium. This was remarkable in the spectrum of 7 (Fig. S26†), where the complexation-induced Cotton effects discontinuously varied with equivalents and ultimately reached a pseudo-mirrored image. In the CD spectra of 3, 6, 8, and 9 in the presence of 13 (Fig. 6b and S26†), roughly speaking, pseudo-mirrored Cotton effects were induced, although extreme wavelengths were slightly changed. To assess a change ratio of dissymmetry before and after complexation (Table 2), the vertical axis was converted from Δ*ε* (Fig. 6) to Δ*ε*/*ε* (Fig. 7).

**Table 2:** Changes in Δ*ε*/*ε* at extreme wavelengths: 1–9 before and after complexation with 13

	Uncomplexed		Complexed			
Molecule	*λ*/nm	|Δ*ε*/*ε*|	*λ*/nm	|Δ*ε*/*ε*|	Equiv. of 13	Change ratio (complexed/uncomplexed)
1	350	0.00325	353	0.00480	2	1.5
	301	0.00114	301	0.00139		1.2
2	337	0.00097	333	0.00103	4	1.1
	310	0.00168	310	0.00181		1.1
3	351	0.00150	348	0.00354	4	2.4
	308	0.00063	308	0.00197		3.1
6	390	0.00060	391	0.00101	6	1.7
	341	0.00033	340	0.00078		2.4
	323	0.00072	321	0.00169		2.4
7	401	0.00068	400	0.00121	6	1.8
	347	0.00025	345	0.00092		3.7
	327	0.00018	325	0.00060		3.3
8	388	0.00215	385	0.00493	6	2.3
	320	0.00051	323	0.00076		1.5
9	386	0.00069	388	0.00350	6	5.1
	323	0.00026	321	0.00083		3.2

For 1 and 2, only one pair of diastereomers was present in the uncomplexed and complexed states; the change ratio was 1.1–1.5 (Fig. 7a and S27† and Table 2). These results showed that the intramolecular transmission of the point chirality (*R*) to the macrocyclic element (*M*/*P*) worked more efficiently in a complex state. About 9, we previously reported that heterochiral conformers (*MMP* and *MPP*) were predominant in the absence of a guest and underwent a change in global conformation from *C*_2_ to *D*_3_ by complexation.^13^ The most significant value (>5) was obtained for 9 (Fig. S27† and Table 2). Isomeric tris(macrocycle)s (6–8) showed a ratio between these extremes (Fig. S27† and Table 2). We considered these results to mean that homochiral and heterochiral conformers coexisted before complexation (Scheme 1b, less controlled), and the population of homochiral conformers was comparable for 6 and 8, which was higher than that of 7. The measured optical rotation value was considered the most underestimated for 7 (Fig. 4). The coexistence of homochiral and heterochiral conformers in 3, shown by NMR spectroscopy (Fig. S15b†), would also be explained by this ratio (Fig. 7b and Table 2), indicating that the actual measured value for 3 was underestimated (Fig. 4).

## Conclusions

We have demonstrated a series of fused oligomers of macrocycles (2–11), in which a dynamic chiral macrocycle (1) (*M*- or *P*-helicity) is repeatedly arranged through fusion. A diversity of arrangements (rotational, acyclic, and cyclic) provided a variety of chiral architectures, which led us to consider optical rotation based on the molecular structure.

In particular, a diastereomeric excess was fortunately estimated for 2, 4, and 5 with a rotational arrangement under an innate or induced preference for a homochiral situation. The rotational angle could modulate the molar optical rotation. The arrangement of elements rotated 180° relative to each other could be responsible for decreased optical rotation, at least in the current system.

Though a diastereomeric ratio for 9–11 could not be estimated at room temperature,^31^ their optical rotations seemed relatively small in a cyclic arrangement.

For bis(macrocycle) 3 and tris(macrocycle)s 6–8, in which multiple conformers coexisted (populations unknown), we uniquely assessed a change ratio of dissymmetry (Δ*ε*/*ε*) before and after complexation with an achiral guest. Based on a difference in the change ratio, we found that a rotational angle between elements could contribute to a difference in the optical rotation, even though a similar value of [*M*]_D_/*N* was observed for three isomeric macrocycles 6–8 with an acyclic arrangement, in which the conformational distribution (degree of mixing, *e.g.*, *MMM* and *MMP* in each original state) was different.

## Conflicts of interest

There are no conflicts to declare.

## Supplementary Material

RA-014-D4RA03709J-s001

RA-014-D4RA03709J-s002
