# Supplementary material for: Study of optical rotation based on the molecular structure in fused oligomers of macrocycles
Source: RSC Adv. 2024 Jul 1;14(29):20735–42. doi: 10.1039/d4ra03709j (PMC11215750; doi:10.1039/d4ra03709j)
Supplement: RA-014-D4RA03709J-s001 [file RA-014-D4RA03709J-s001.pdf]

Electronic Supplementary Material (ESI) for RSC Advances.
This journal is © The Royal Society of Chemistry 2024

Electronic Supplementary Information_1

Consideration of optical rotation based on the molecular structure in fused oligomers of macrocycles

Ryo Katoono*, Yudai Obara, Kazuki Sakamoto and Rei Miyashita
Department of Chemistry, Faculty of Science, Hokkaido University

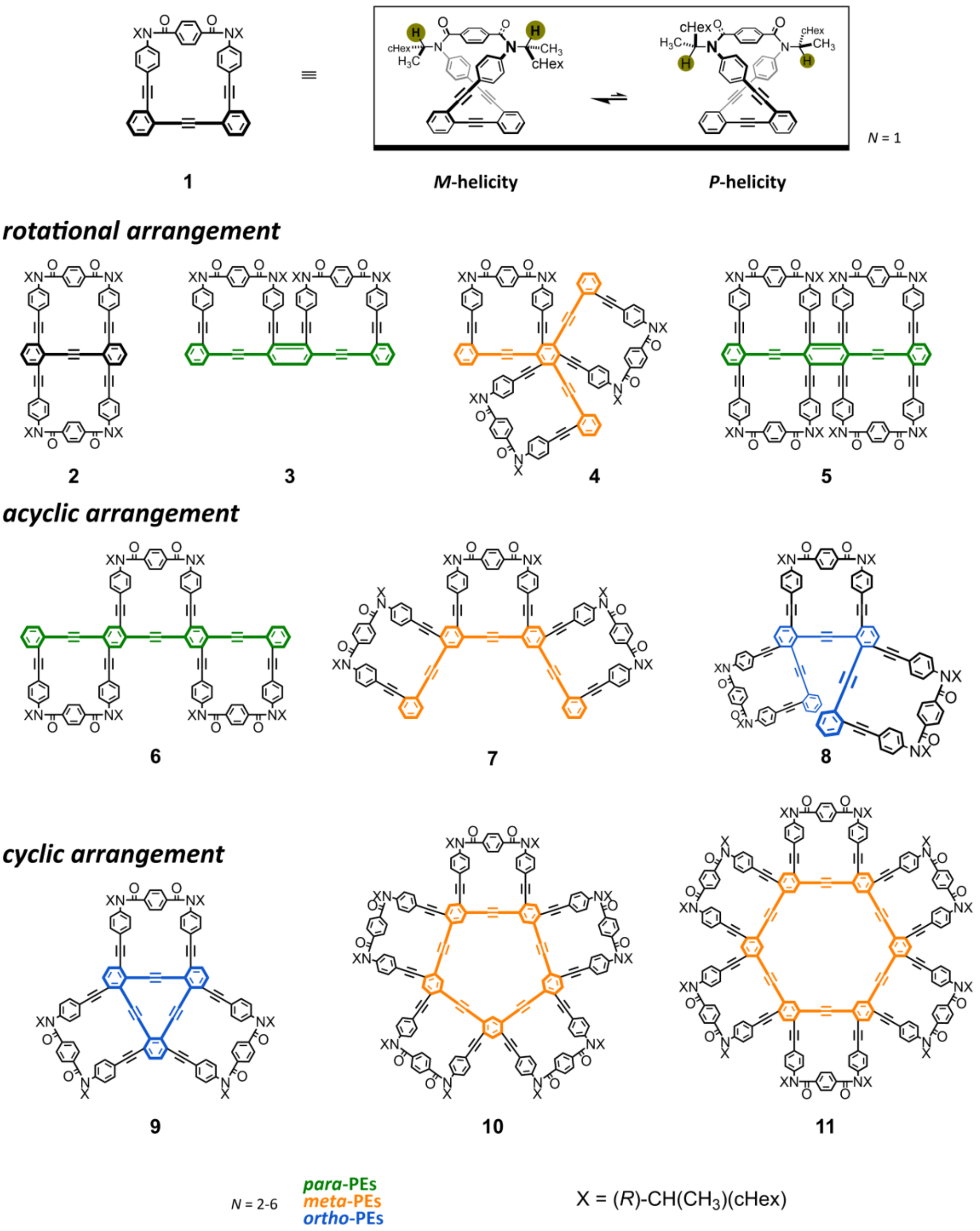

Contents (ESI_1)

Supplementary Figures (**Figures S1-S27**)	page
Conformational searches using MacroModel software.	S3-S9
^{1}H NMR spectra of **1-11**.	S10-S16
Quantitative estimation of the diastereomeric ratio induced in **1**, **2**, **4**, and **5**.	S17-S22
VT-CD spectra of **3**, **6**, **7**, **8**, and **9**.	S23-S24
UV spectra of **1-11**.	S25
Complexation experiment [X = butyl] with a chiral guest (**14**).	S26-S35
UV and CD spectra of **3**, **6**, **7**, **8**, and **9** in the presence of an achiral guest (**13**).	S36-S37
Plots of $\Delta\varepsilon/\varepsilon$ for **3**, **6**, **7**, **8**, and **9** before and after complexation (**13**).	S38
Experimental (**Schemes S1-S3**)	S39-S59

Contents (ESI_2)

Copies of ^{1}H/^{13}C NMR spectra and LRMS of new compounds

Supplementary Figures

Conformational searches using MacroModel software.

First, we note the results of conformational searches using software for the monomeric macrocycles (*N* = 1) **1M** [X = CH_3] and **1** [X = (*R*)-CH(CH_3)(cHex)] (**Fig. S1**): only a twisted form was found for model **1M**, and an *M*-helical form (*M*-**1**) was estimated to be more stable than a *P*-helical form (*P*-**1**) (+1.30 kJ mol^{-1}).[1] This was also the case of bis(macrocycle)s (*N* = 2) **2M** and **2** (**Fig. S1**): only a homochiral form was found for model **2M**, and *MM*-**2** was estimated to be more stable than *PP*-**2** (+2.65 kJ mol^{-1}).[2] These differences in the conformation energy between two twisted forms with *M* (*MM*)- or *P* (*PP*)-helicity of **1** (**2**) would be due to a difference in the spatial location of H, CH_3, and cHex groups with respect to the carbonyl group.

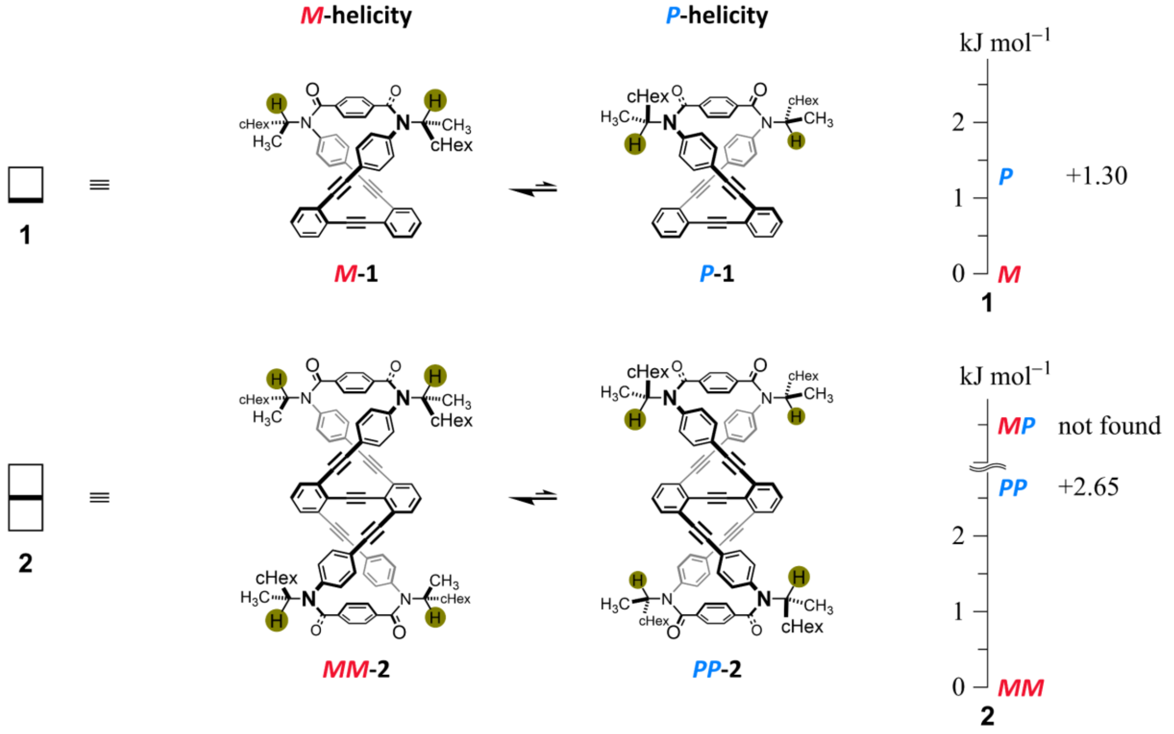

Fig. S1. Conformation energies for diastereomeric forms of **1** (*M*-**1** and *P*-**1**) and **2** (*MM*-**2** and *PP*-**2**) [X = (*R*)-CH(CH_3)(cHex)], obtained by conformational searches using MacroModel software. Conditions: MacroModel v9.9 OPLS_2005, Monte Carlo Multiple Minimum (MCMM), non-solvated. cHex: cyclohexyl.

Next, for other fused oligomers of macrocycles (*N* = 2 or greater), conformational searches were implemented only for model molecules [X = CH_3], **A-D** (**Fig. S2**)[3] and **3M-11M** (**Figs. S2-S6**). The controllability of conformation would be due to the arrangement. For example, there would be some conformational constraints in a cyclic arrangement, where both a newly generated macrocycle and the peripheral macrocyclic elements possess their conformational preferences, which can be competitive. Alternatively, such a constraint seems easy to resolve in an

---

1 R. Katoono, Y. Tanaka, K. Kusaka, K. Fujiwara and T. Suzuki, *J. Org. Chem.*, 2015, **80**, 7613.
2 R. Katoono, S. Kawai, K. Fujiwara and T. Suzuki, *Chem. Sci.*, 2015, **6**, 6592.
3 R. Katoono, T. Kudo and S. Kawai, *Org. Biomol. Chem.*, 2023, **21**, 2562.

acyclic arrangement. Rotational is one form of acyclic. However, rotational was discriminated from acyclic since some constraints might be supposed to be when all elements must share a single fused point. These arrangement-driven constraints could factor in the designation of a preferred conformation.

For tris(macrocycle) **4M**[3] with a rotational arrangement (**Fig. S2**), a homochiral (*MMM*) form was found to be more stable, like the case of **2M** (N = 2). These examples show that conformational interinfluence works cooperatively, allowing each macrocyclic element to prefer the same sense (*Cf.* **Scheme 1b**, controlled). Alternatively, a search for tetrakis(macrocycle) **5M** found that the conformation energy of a homochiral (*MMMM*) form was relatively higher than that of a heterochiral form [*MPMP* in the rings 1, 2, 3, and 4 in this order (**Figs. S2** and **S3**)], which was only found among other heterochiral forms (*MMPP* or *MPPM* was not found, like the case that *MP* was not found for **2M**). This example shows that conformational interinfluence works competitively to allow the molecule to adopt a *meso*-like conformation predominantly (*Cf.* **Scheme 1b**, controlled). These innate conformational preferences in **4M** (N = 3) and **5M** (N = 4) seemed similar to those predicted for substructural bis(macrocycle)s **A** (N = 2) and **3M** (N = 2), respectively (**Fig. S2**).

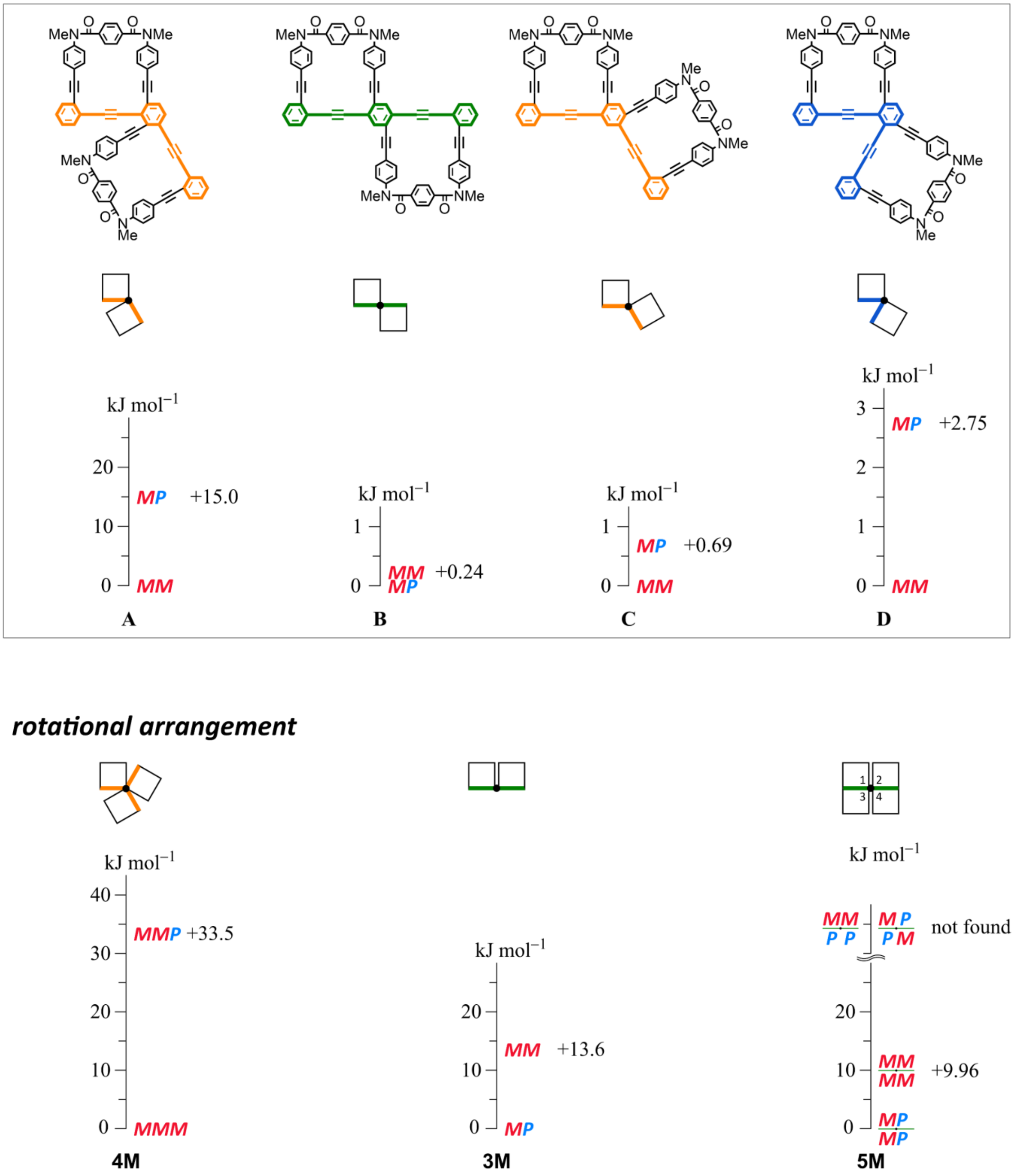

Fig. S2. Conformation energies for diastereomeric (homochiral and heterochiral) forms of model bis(macrocycle)s **A-D** and **3M** (N = 2) [X = CH_3] and the corresponding model molecules **4M** (N = 3) and **5M** (N = 4) [X = CH_3], obtained by conformational searches using MacroModel software. Only a particular form of enantiomers is depicted—conditions: MacroModel v11.8 OPLS3e, MCMM, non-solvated.

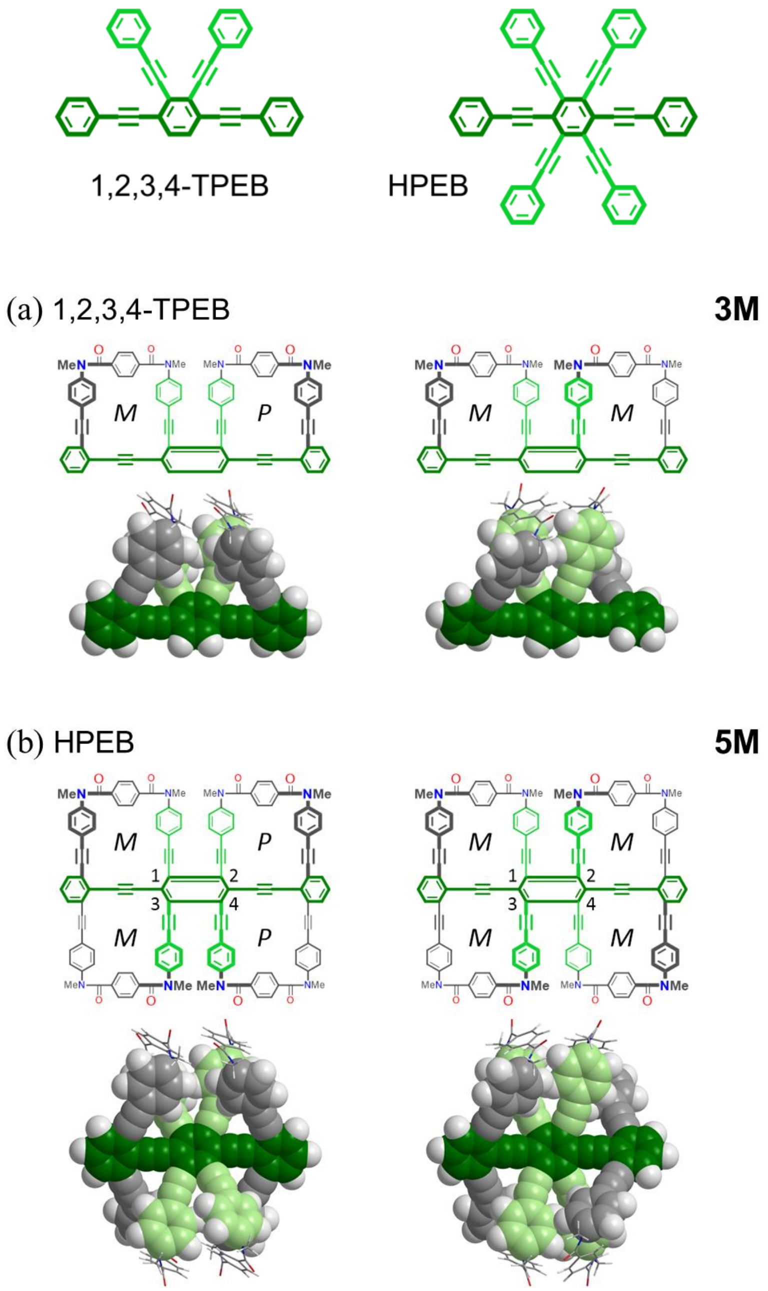

Fig. S3. Energy-minimized structures for heterochiral (left) and homochiral (right) forms of model bis- or tetrakis(macrocycle) [X = CH_3]: (a) **3M** and (b) **5M**, obtained by conformational searches using MacroModel software. Only a particular form of enantiomers is depicted. Space-filling representation for phenylene ethynylenes and stick representation for terephthaloyl bridges.

Searches for model bis(macrocycle)s **B**, **C**, and **D** showed that a difference in the conformation energy between homochiral (*MM*) and heterochiral (*MP*) forms could be changed according to a difference in the arrangement (**Fig. S2**). Similar results were obtained by the corresponding tris(macrocycles)s **6M**, **7M**, and **8M** with an acyclic arrangement (**Figs. S4** and **S5**).

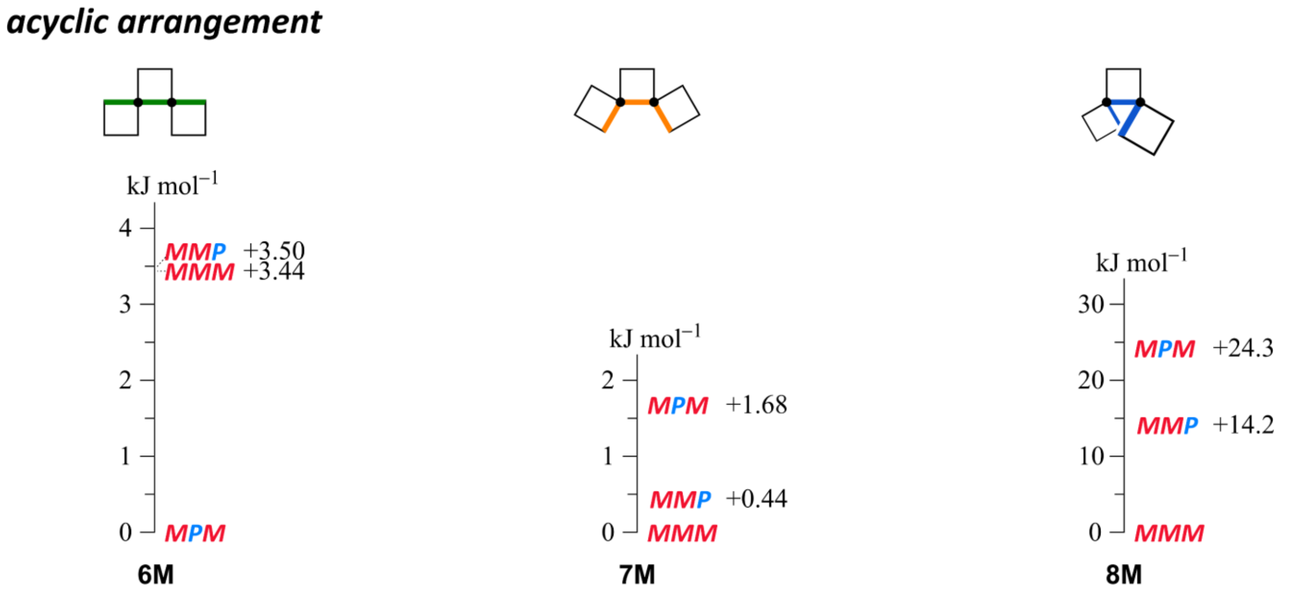

Fig. S4. Conformation energies for diastereomeric (homochiral and heterochiral) forms of model tris(macrocycle)s **6M-8M** [X = CH_3], obtained by conformational searches using MacroModel software. Only a particular form of enantiomers is depicted—conditions: MacroModel v11.8 OPLS3e, MCMM, non-solvated.

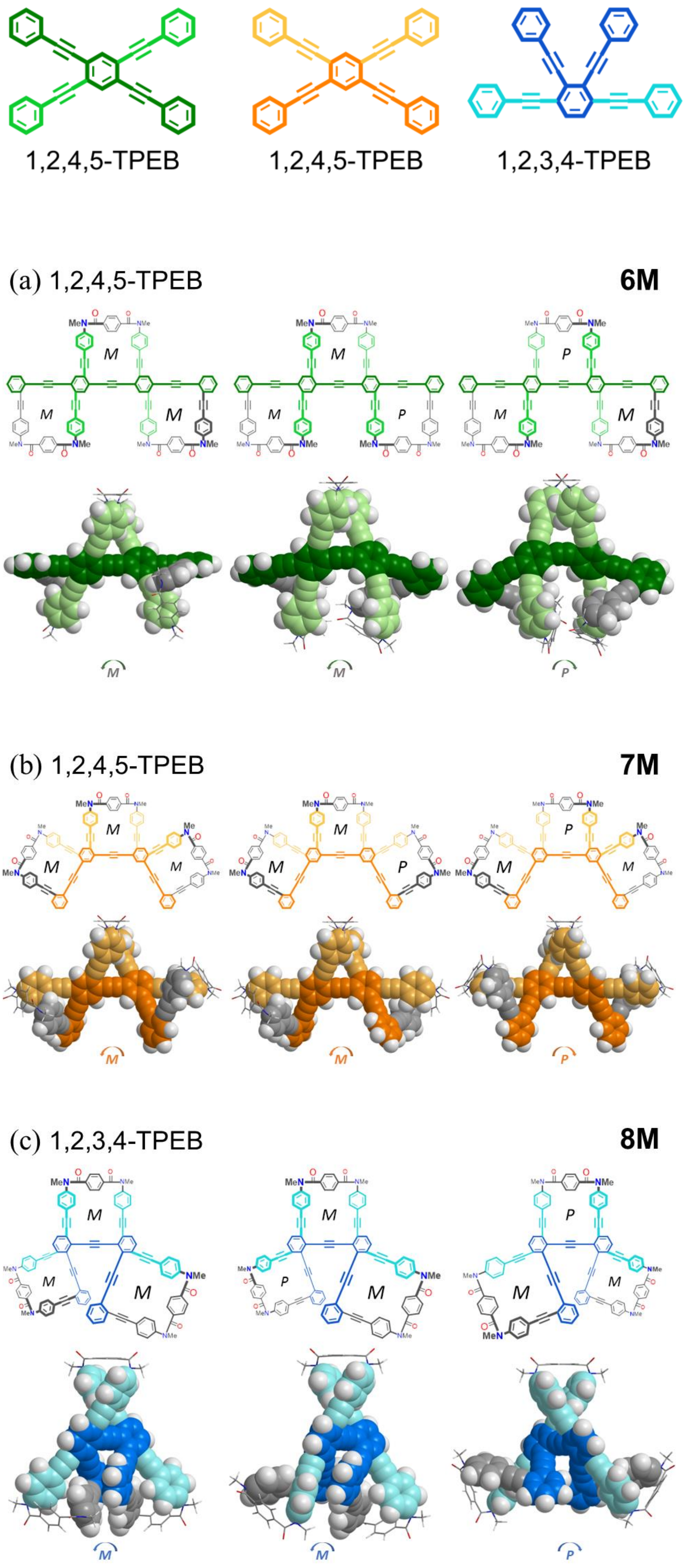

Fig. S5. Energy-minimized structures for *MMM* (left), *MMP* (center), and *MPM* (right) of model tris(macrocycle)s [X = CH_3]: (a) **6M**, (b) **7M**, and (c) **8M**, obtained by conformational searches using MacroModel software. Only a particular form of enantiomers is depicted. Space-filling representation for phenylene ethynylenes and stick representation for terephthaloyl bridges.

For model macrocycles of macrocycles **9M**[5] ($N = 3$), **10M**[4] ($N = 5$), and **11M**[4] ($N = 6$), a heterochiral form was found as the lowest-energy conformation, and an element seemed keen to prefer an opposite sense with respect to the sense in the neighboring element in each cyclic arrangement (**Fig. S6**).

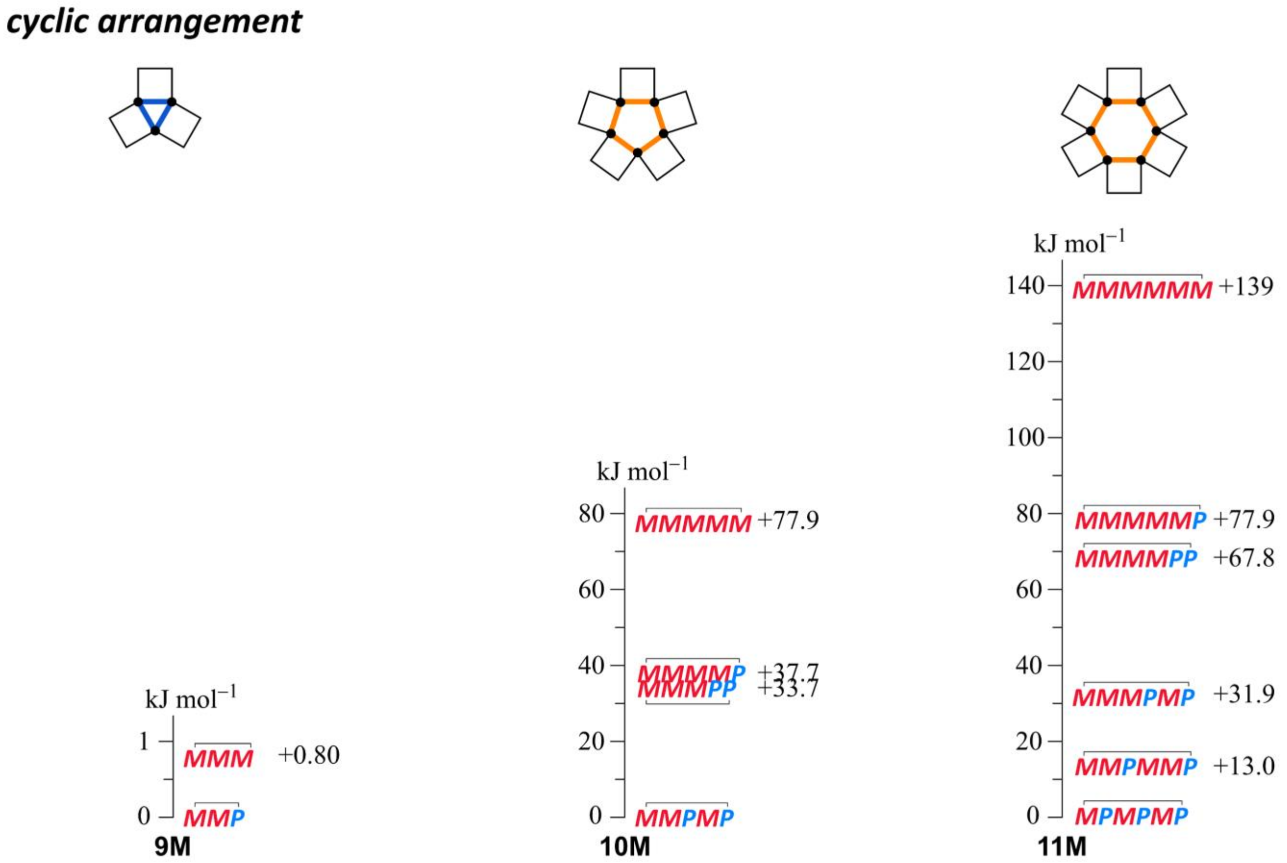

Fig. S6. Conformation energies for diastereomeric (homochiral and heterochiral) forms of model macrocycles of macrocycles **9M-11M** [X = CH_3], obtained by conformational searches using MacroModel software. Only a particular form of enantiomers is depicted—conditions: MacroModel v11.8 OPLS3e, MCMM and Low-frequency-mode, non-solvated.

---

4 R. Katoono, *Chem. Lett.*, 2023, **52**, 627.
5 R. Katoono, K. Sakamoto and T. Suzuki, *Chem. Commun.*, 2019, **55**, 5503.

^{1}H NMR spectra of fused oligomers of macrocycles.

The fusion between macrocyclic elements led to a change in the molecular symmetry. A twofold axis in the monomeric element **1** was seen in each element of **2**, **9**, **10**, and **11**, while this axis disappeared in some elements of **3-8**. This lack of a two-fold axis led to non-equivalency within a macrocyclic element (A in **3-8**) and non-equivalency between elements (A ≠ B, ABA in **6**, **7**, and **8**) (**Fig. S7**).

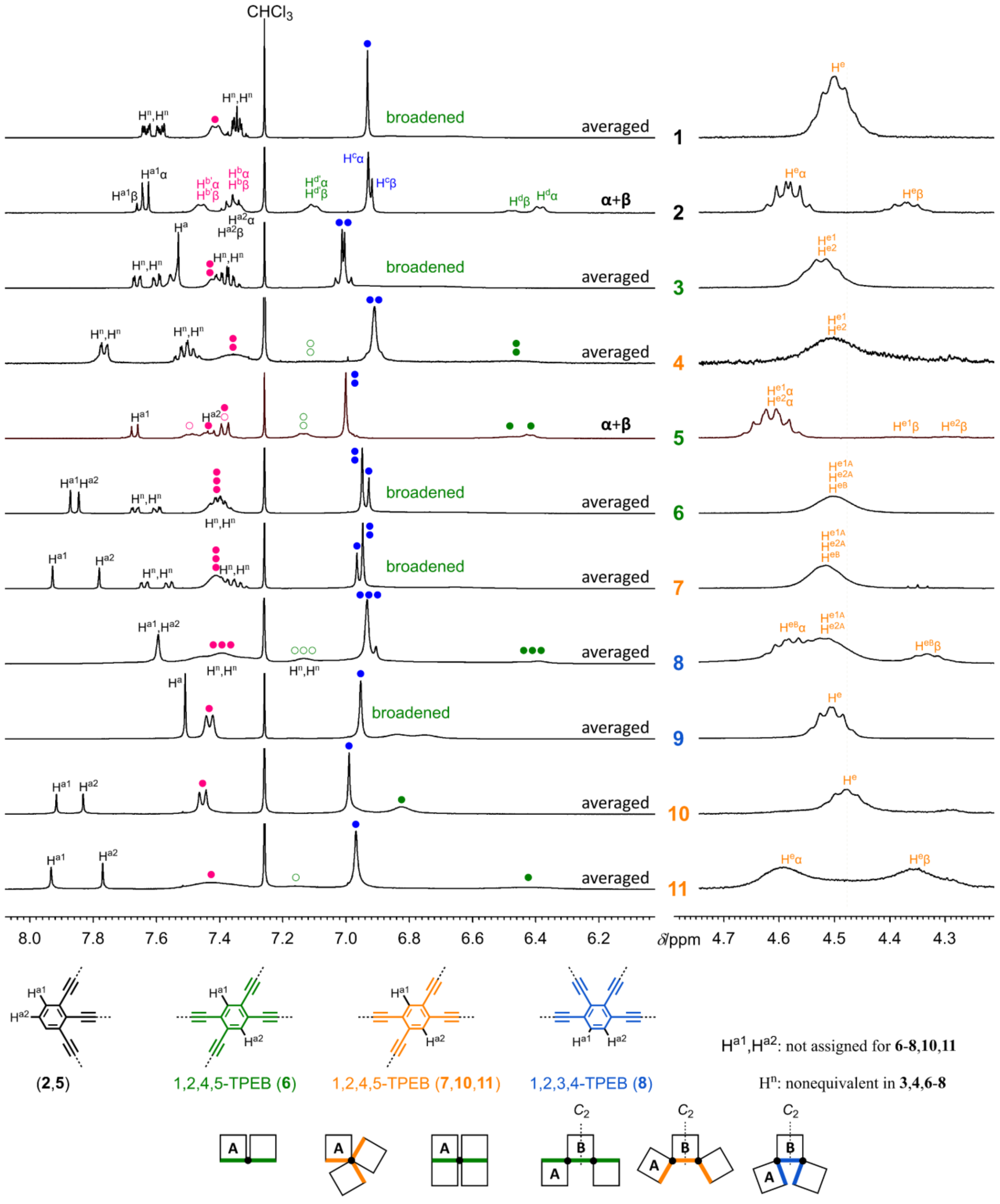

Fig. S7. Partial ^{1}H NMR spectra (400 MHz, left: aromatic protons and right: methine protons) of **1-11**, measured in $CDCl_3$ at room temperature.

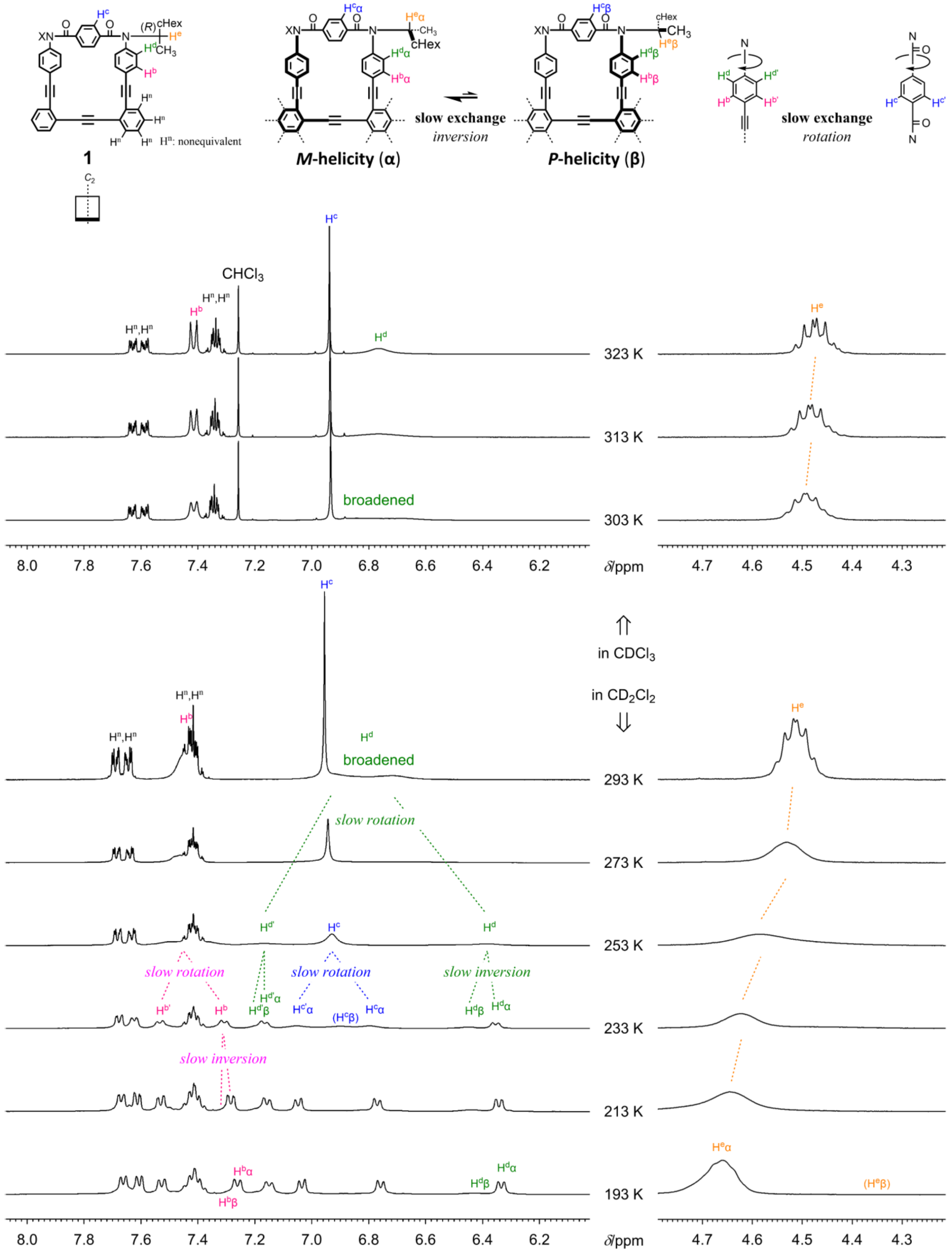

Fig. S8. Partial VT-^{1}H NMR spectra (400 MHz, left: aromatic protons and right: methine protons) of **1** (N = 1), measured in CD_2Cl_2 at 193-293 K and $CDCl_3$ at 303-323 K.

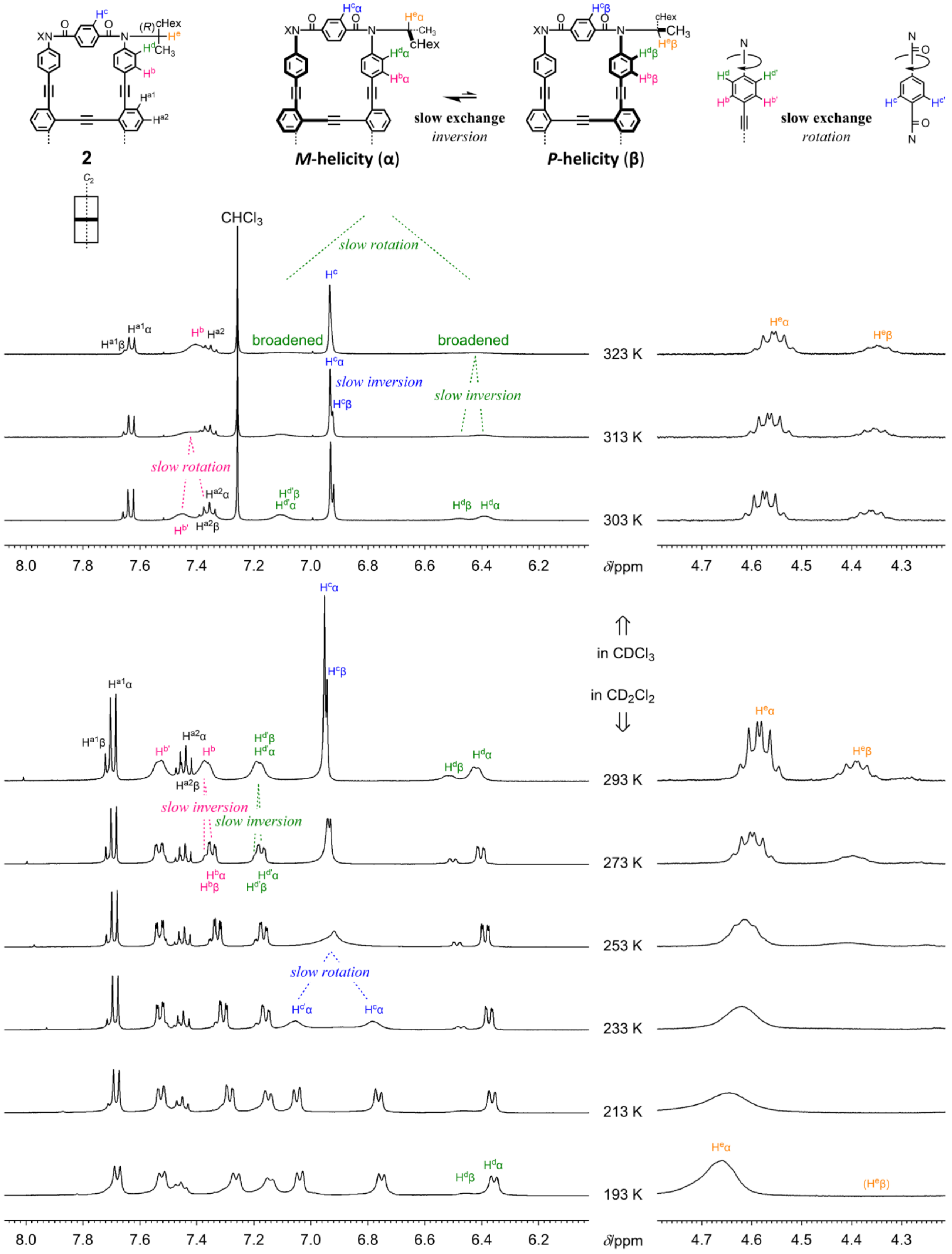

Fig. S9. Partial VT-^{1}H NMR spectra (400 MHz, left: aromatic protons and right: methine protons) of **2** (N = 2), measured in CD_2Cl_2 at 193-293 K and $CDCl_3$ at 303-323 K.

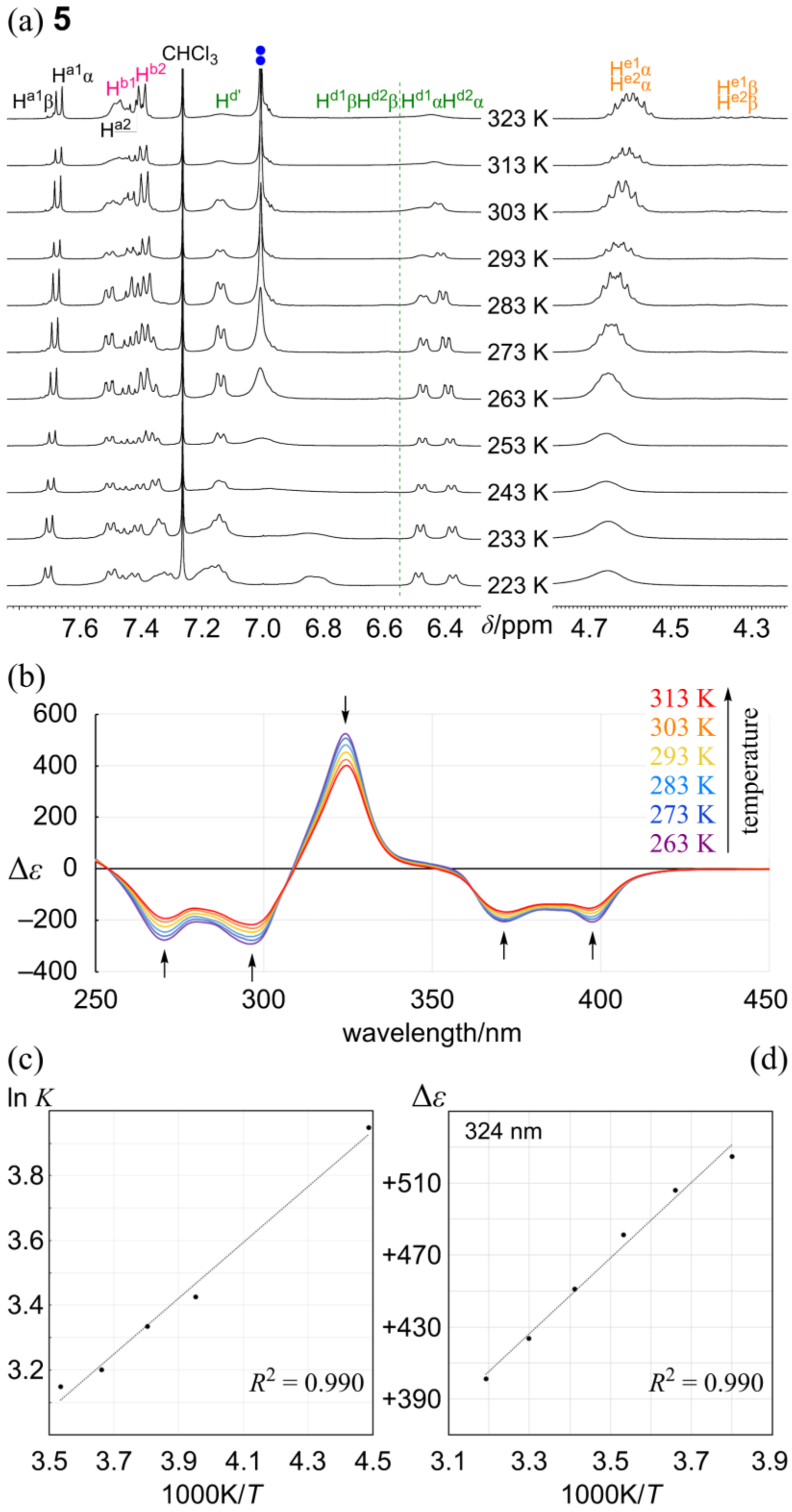

Fig. S10. (a) Partial VT-^{1}H NMR spectra (400 MHz, left: aromatic protons and right: methine protons) of **5** ($N = 4$), measured in $CDCl_3$ at 223-323 K; (b) VT-CD spectra of **5**, measured in CH_2Cl_2 at 263-313 K; (c) a plot of ln K versus $1/T$ and (d) a plot of $\Delta\varepsilon$ (at 324 nm) versus $1/T$ for the conformational interconversion between two diastereomeric conformers (*MMMM* and *PPPP*) in a solution of **5**. K = [α (major)]/[β (minor)]. $\Delta\varepsilon$ [L·mol^{-1}·cm^{-1}].

Cf.

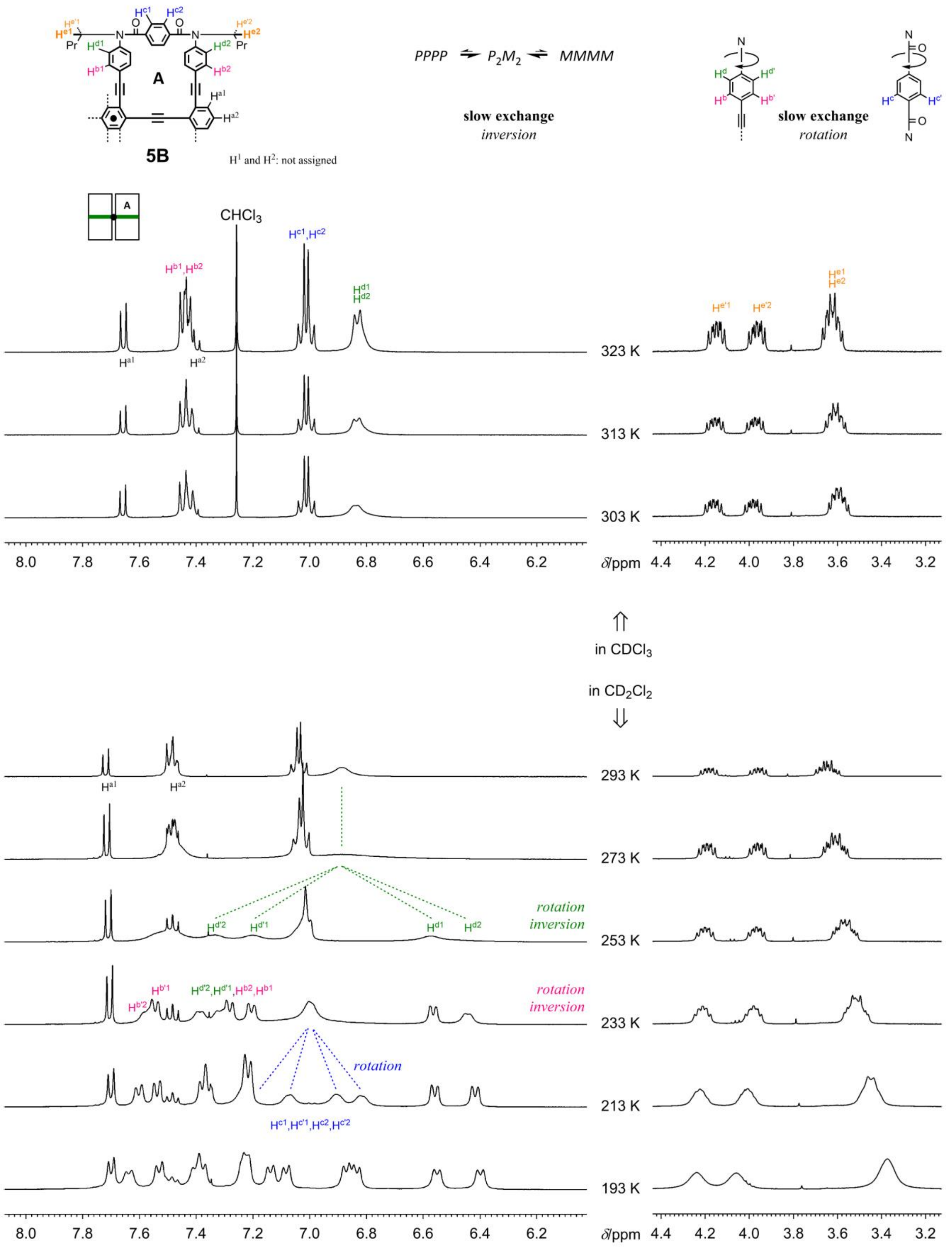

Fig. S11. Partial VT-^{1}H NMR spectra (400 MHz; left: aromatic protons and right: anisochronous methylene protons) of **5B** ($N = 4$), measured in CD_2Cl_2 at 193-293 K and $CDCl_3$ at 303-323 K.

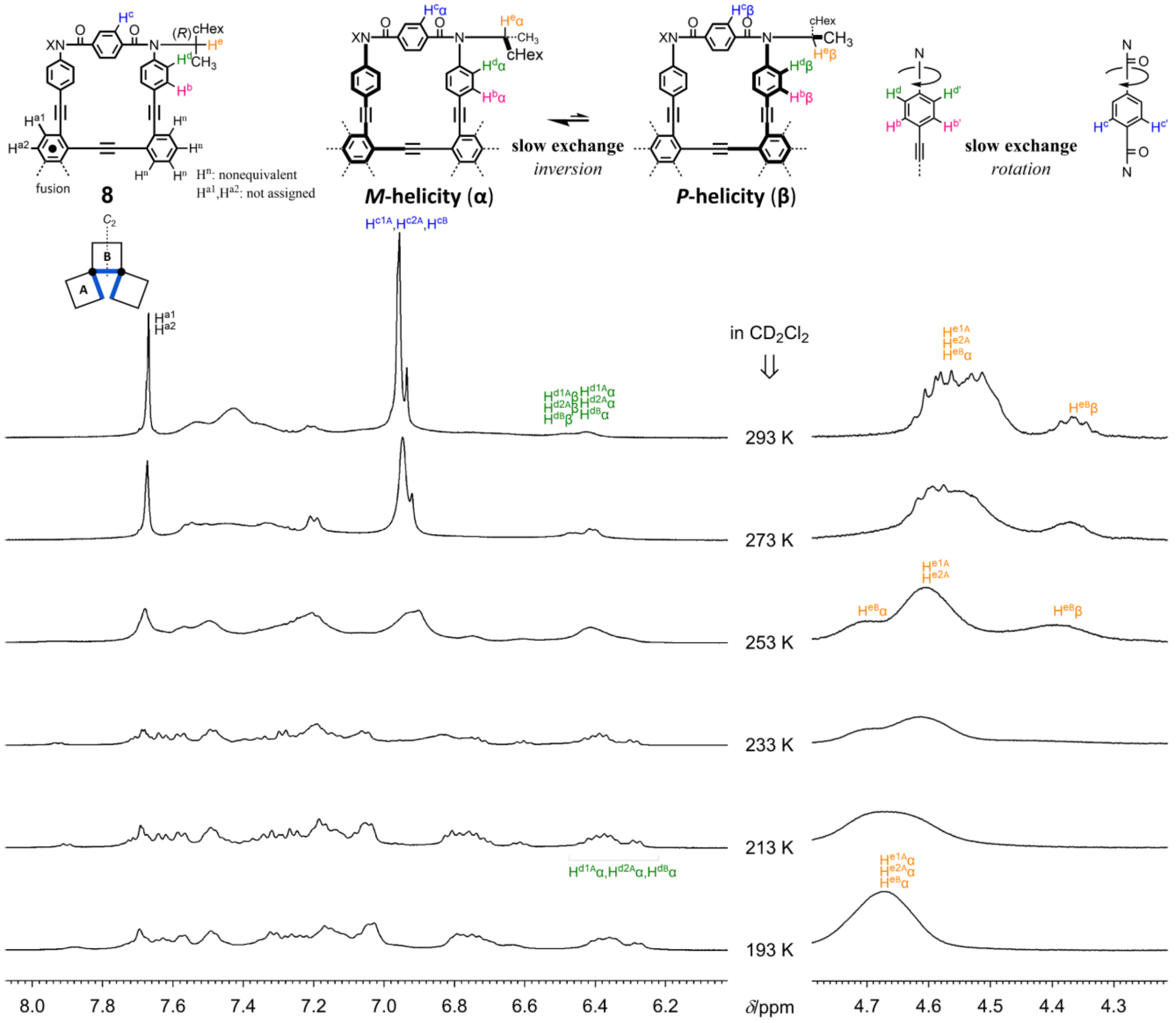

Fig. S12. Partial VT-^{1}H NMR spectra (400 MHz, left: aromatic protons and right: methine protons) of **8** (N = 3), measured in CD_2Cl_2 at 193-293 K.

Cf.

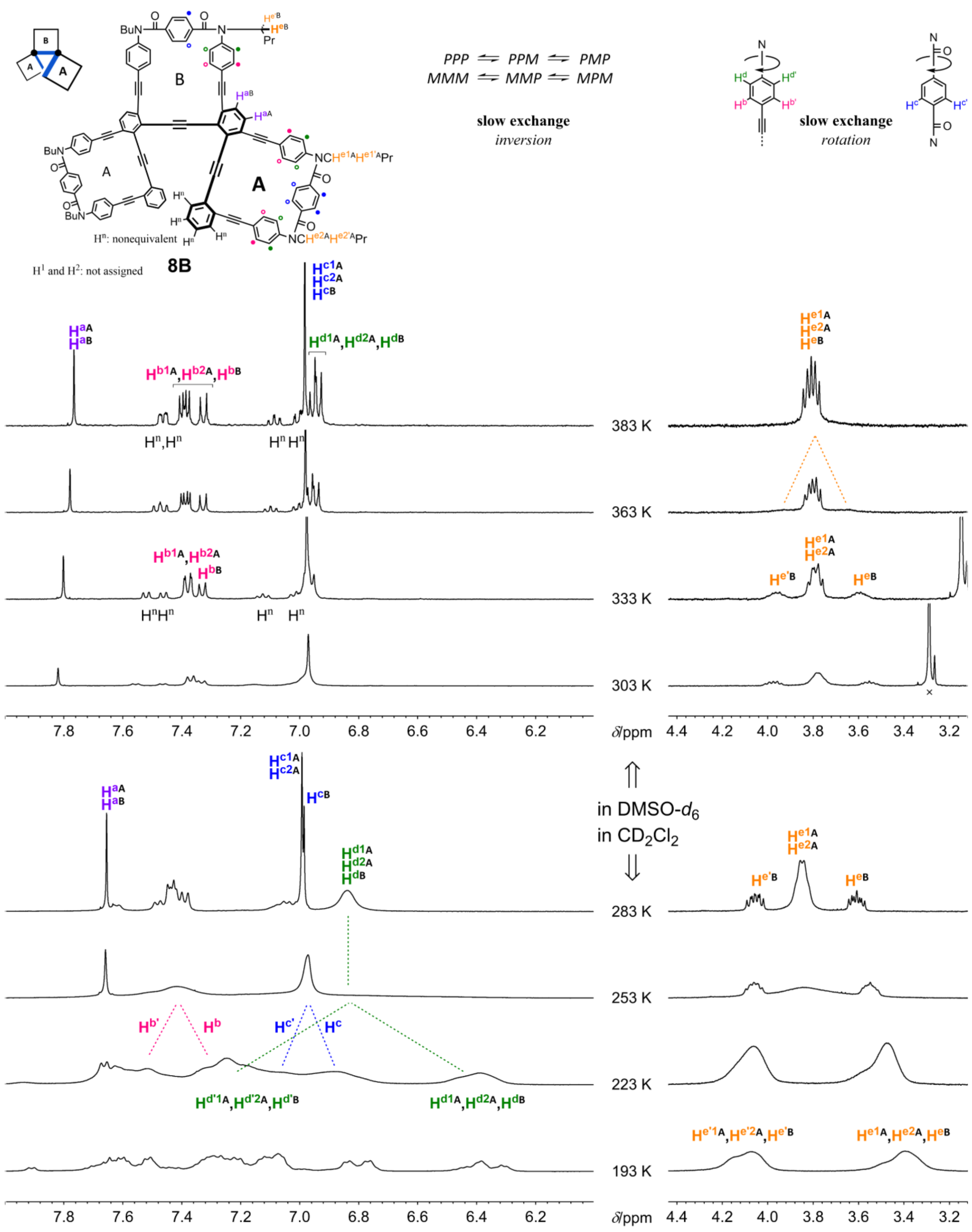

Fig. S13. Partial VT-^{1}H NMR spectra (400 MHz; left: aromatic protons and right: anisochronous methylene protons) of **8B** (N = 3), measured in CD_2Cl_2 at 193-283 K and DMSO-d_6 at 303-383 K.

Quantitative estimation of the diastereomeric ratio induced in 1, 2, 4, and 5.

At lower temperatures, the global interconversion between two helically-twisted forms (α and β) and local rotations of *p*-phenylene rings (H^{b} and $H^{b'}$, H^{c} and $H^{c'}$, and H^{d} and $H^{d'}$) slowed (**Fig. S14**). These paired signals are based on chemical exchange and coalesce/decoalesce according to each barrier and difference in the chemical shift (**Figs. S8-S13** and **S15-S18**).[6] As representative examples of the conformational interconversion between two diastereomeric (pseudo-enantiomeric) conformers, we noted two different results of VT-^{1}H NMR measurements for bis(macrocycle)s **2** and **3** (**Fig. S15**), which are both considered to be a substructure of **5** (N = 4). In the spectra of **2** (**Figs. S9** and **S15a**), only two species were present throughout the entire range of temperature, and they were assigned to *MM*-**2** and *PP*-**2** since this observation was significantly similar to the result of **1** (*M*-**1** and *P*-**1**), measured at below 253 K (**Fig. S8**). Alternatively, in the spectra of **3** (**Fig. S15b**), lowering temperatures revealed that a heterochiral conformer (*MP*), as well as a diastereomeric pair of homochiral conformers (*MM* and *PP*), was involved in the equilibrium. This coexistence of homochiral and heterochiral conformers was also confirmed by **3B** (X = Bu) (**Fig. S16**). The latter was preferred over the former (major : minor = 1.75 : 1). Participation of heterochiral conformer(s) in the equilibrium was similarly observed in the spectra of tris(macrocycle)s **6**, **7**, and **8** with an acyclic arrangement (**Figs. S12**, **S17**, and **S18**), where the conformational distribution was indeterminate due to broadening and overlapping.

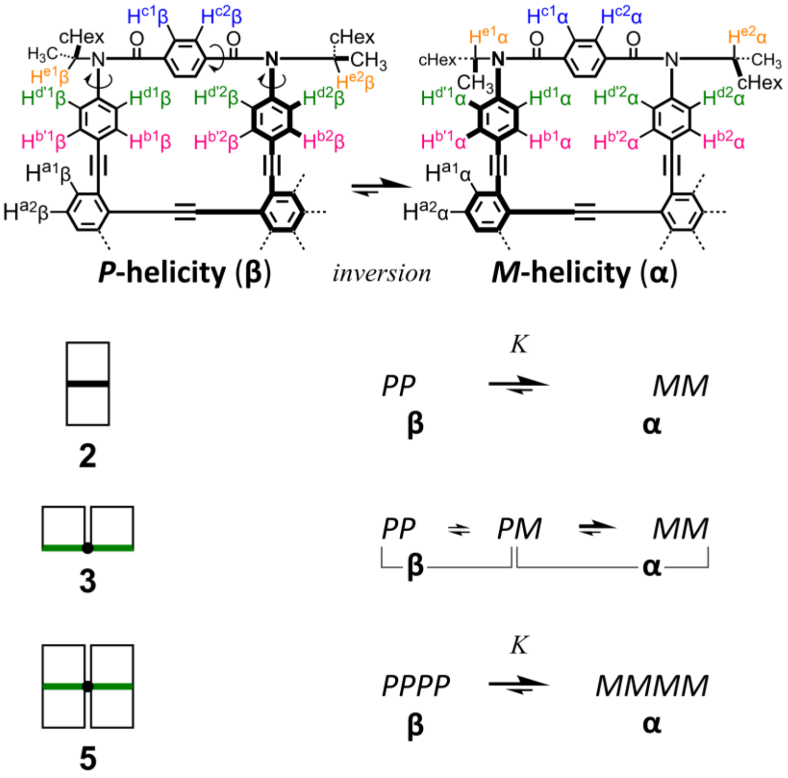

Fig. S14. Conformational interconversion between two diastereomeric (pseudo-enantiomeric) conformers with *M*-or *P*-helicity (M_N and P_N) with [**3** (N = 2)] or without [**2** (N = 2) and **5** (N = 4)] participation of a heterochiral conformer.

---

6 A. D. Bain, *Prog. Nucl. Magn. Reson. Spectrosc.*, 2003, **43**, 63.

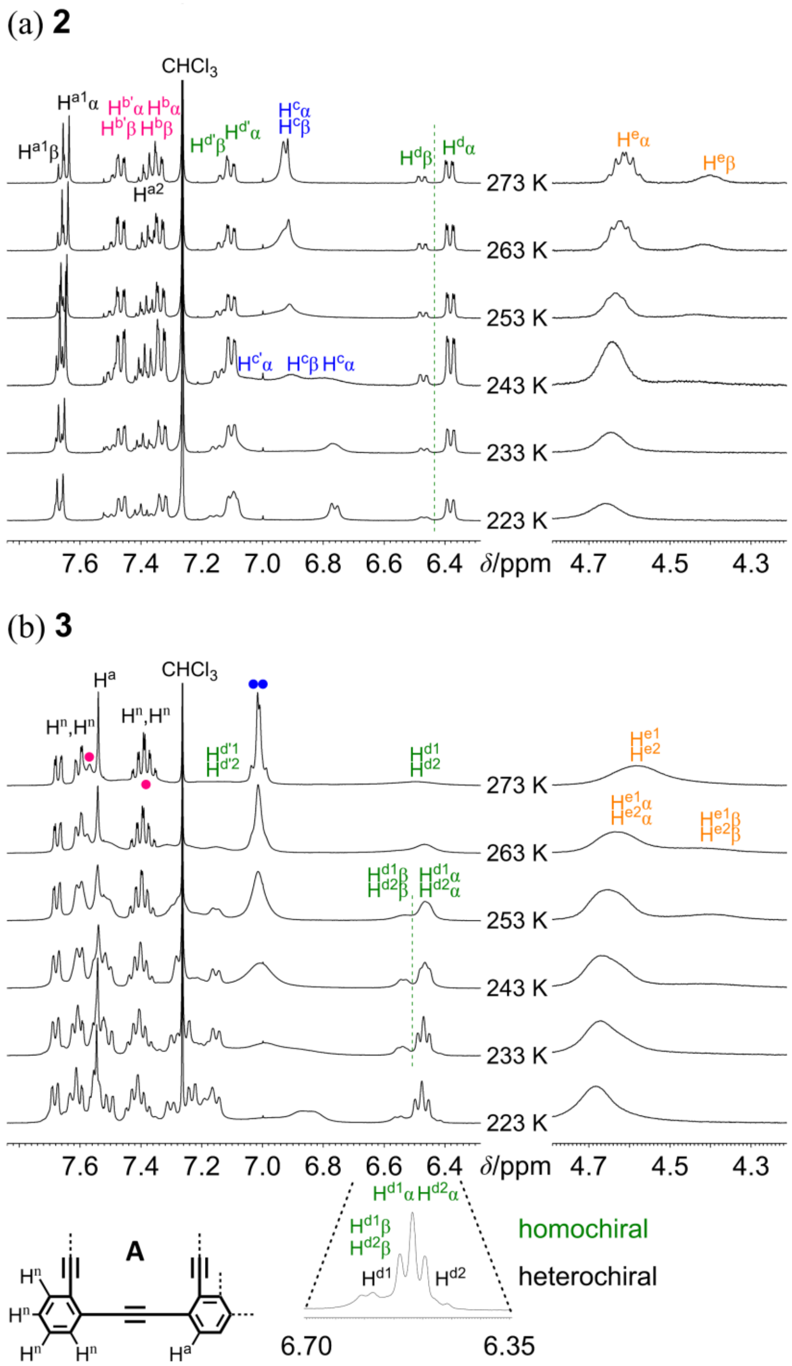

Fig. S15. Partial VT-^{1}H NMR spectra (400 MHz, left: aromatic protons and right: methine protons) of (a) **2** ($N = 2$) and (b) **3** ($N = 2$), measured in $CDCl_3$ at 223-273 K.

Cf.

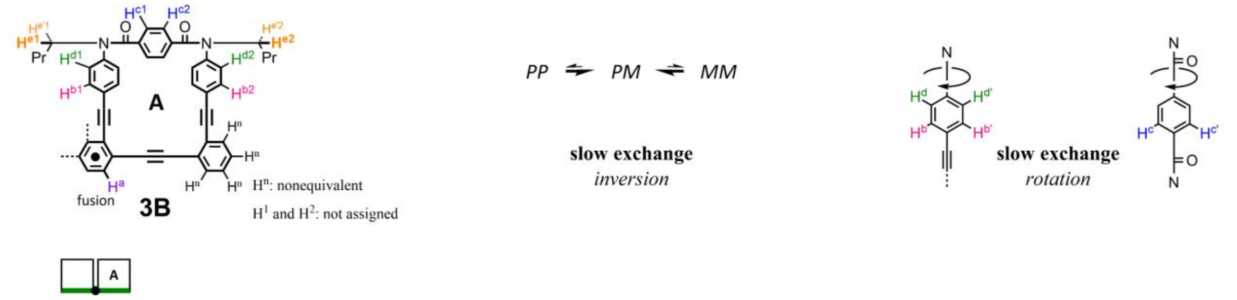

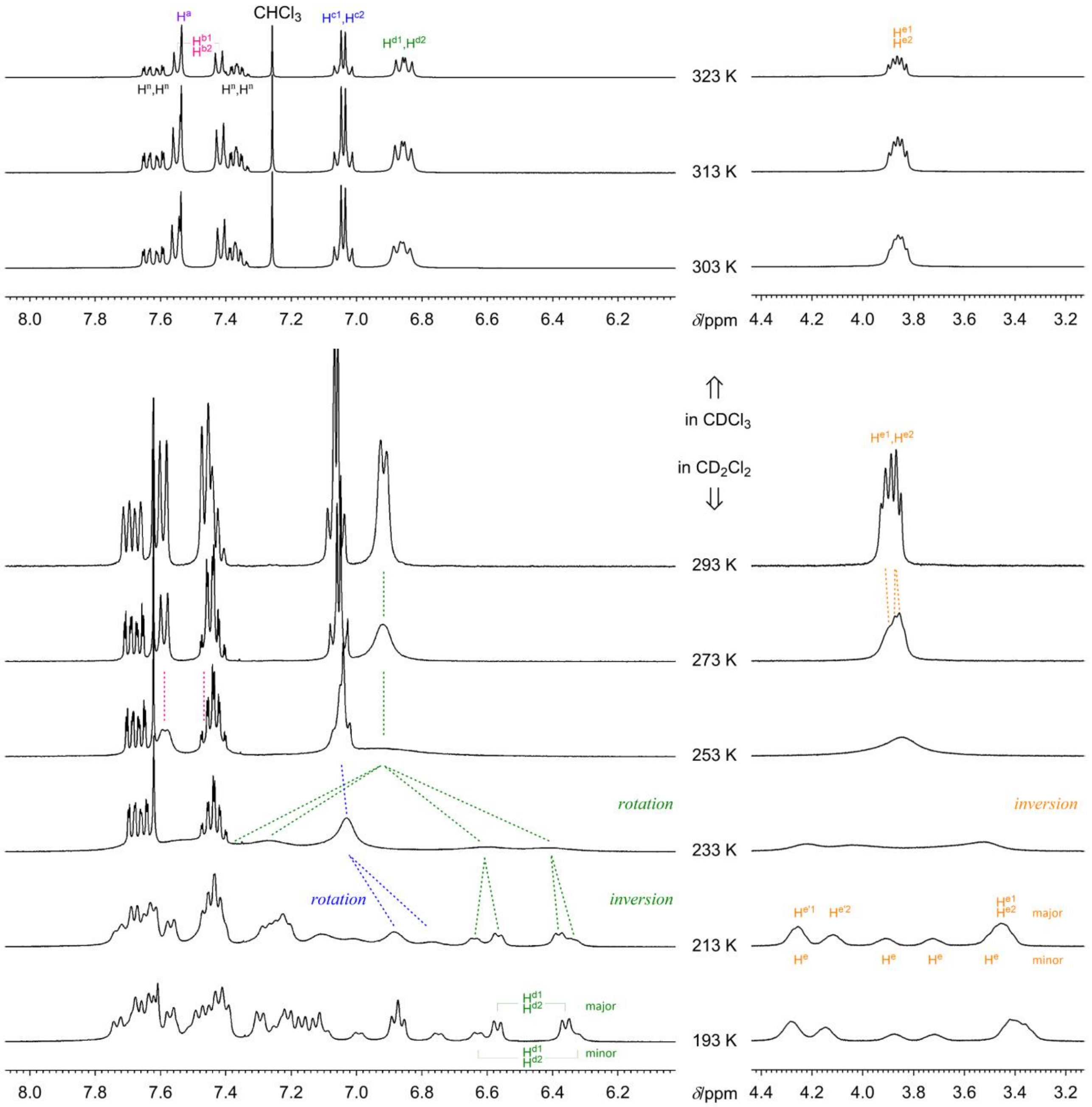

Fig. S16. Partial VT-^{1}H NMR spectra (400 MHz; left: aromatic protons and right: anisochronous methylene protons) of **3B** ($N = 2$), measured in CD_2Cl_2 at 193-293 K and $CDCl_3$ at 303-323 K.

We previously reported a "macrocycle of macrocycles", in which the interinfluence allowed a macrocyclic element to prefer the same (**10** and **11**)[4] or different (**9**)[5] sense according to the senses of the neighboring elements by NMR spectroscopy at low temperatures, although a quantitative estimation was not possible.

In particular, we estimated a diastereomeric ratio of a single pair of pseudo-enantiomeric conformers with *M*- or *P*-helicity induced in **2** ($N = 2$)[2], **4** ($N = 3$)[3], and this time **5** ($N = 4$) based on NMR spectroscopy under an innate or induced conformational preference for a homochiral situation, as with the monomeric case of **1** ($N = 1$)[1].

Based on chemical exchanges in ^{1}H NMR spectroscopy, we reported that a diastereomeric ratio of α/β induced at 223 K in chloroform was estimated to be 2.3 for *M*-**1** and *P*-**1**, 4.7 for *MM*-**2** and *PP*-**2**, and 9.9 for *MMM*-**4** and *PPP*-**4** by linear extrapolation.[3] The ^{1}H NMR spectra of **5** (**Fig. S10a**) showed that only homochiral conformers were predominantly present in solution, like the case of **2** (**Fig. S15a**), not **3** (**Fig. S15b**). Similarly, a linear extrapolation estimated the diastereomeric ratio (α/β) at 223 K was ca. 51 for *MMMM*-**5** and *PPPP*-**5** (**Fig. S10c**) [The diastereomeric ratio was estimated, assuming the minor conformer was assigned to *PPPP*-**5**]. Although there was an undeniable sense that uncertainty about integration remained due to such a tremendous bias, the presence of a minor diastereomer was ensured by VT-CD measurements.

On varying temperatures (263-313 K), in the CD spectra of **5** (**Fig. S10b**), there was no change in terms of the spectral appearance, but the intensity of induced Cotton effects ($\Delta\varepsilon$, molar circular dichroism [$L\cdot mol^{-1}\cdot cm^{-1}$]) changed with temperature while maintaining several isosbestic points. This result supported the idea that two pseudo-enantiomeric conformers were involved in the equilibrium. To calculate a diastereomeric excess (de) at an arbitrary temperature, we plotted several values of $\Delta\varepsilon$ measured at 324 nm versus $1/T$ and obtained a linear relationship (**Fig. S10d**). Based on this relationship, we estimated an imaginary value of $\Delta\varepsilon$ at 223 K (**Table S1**). Using the above-noted extrapolated value of α/β at 223 K by NMR spectroscopy, we calculated $|\Delta\varepsilon_{max}|$ and converted measured values of $\Delta\varepsilon$ at several temperatures to de values (**Table S1**).

Tetrakis(macrocycle)s **5** [X = (*R*)-1-cyclohexylethyl] and **5B** [X = butyl] were valid to represent two types of conformational interinfluence, cooperation and competition. A particular conformation of homochiral *MMMM*-**5** (cooperation) or heterochiral *MPMP*-**5B** (competition) was predominantly present in each solution. These indefectible controls would be attained due to a no-way-out situation in which all elements were assembled around the single fused point. This was also considered the case for **2** and **4** (not **3**) (**Fig. S14**).

Table S1. Calculated values of $\Delta\varepsilon_{223K}$ [$L\cdot mol^{-1}\cdot cm^{-1}$], $|\Delta\varepsilon_{max}|$, K, and diastereomeric excess (de)[a], based on the results of VT-NMR and VT-CD measurements for the conformational interconversion between two diastereomeric (pseudo-enantiomeric) conformers (*MMMM* and *PPPP*) in a solution of **5**, measured in $CDCl_3$ at 223-323 K (NMR, **Fig. S10a**) and CH_2Cl_2 at 263-313 K (CD, **Fig. S10b**).

temperature	$\Delta\varepsilon$ measured at 324 nm	K	de[a]
313 K	401.3	3.67	57%
303 K	423.8	4.05	60%
293 K	451.3	4.61	64%
283 K	481.3	5.37	69%
273 K	506.1	6.18	72%
263 K	524.8	6.93	75%
	$\Delta\varepsilon_{223K}$ (extrapolation, **Fig. S10d**) 674	Hα/Hβ (extrapolation, **Fig. S10c**) 51	96%
	$|\Delta\varepsilon_{max}|$[a] 702		

[a]An imaginary value of $|\Delta\varepsilon_{max}|$ is related to measured values of $\Delta\varepsilon$ and de according to the following equation: de = $\Delta\varepsilon/|\Delta\varepsilon_{max}|$.

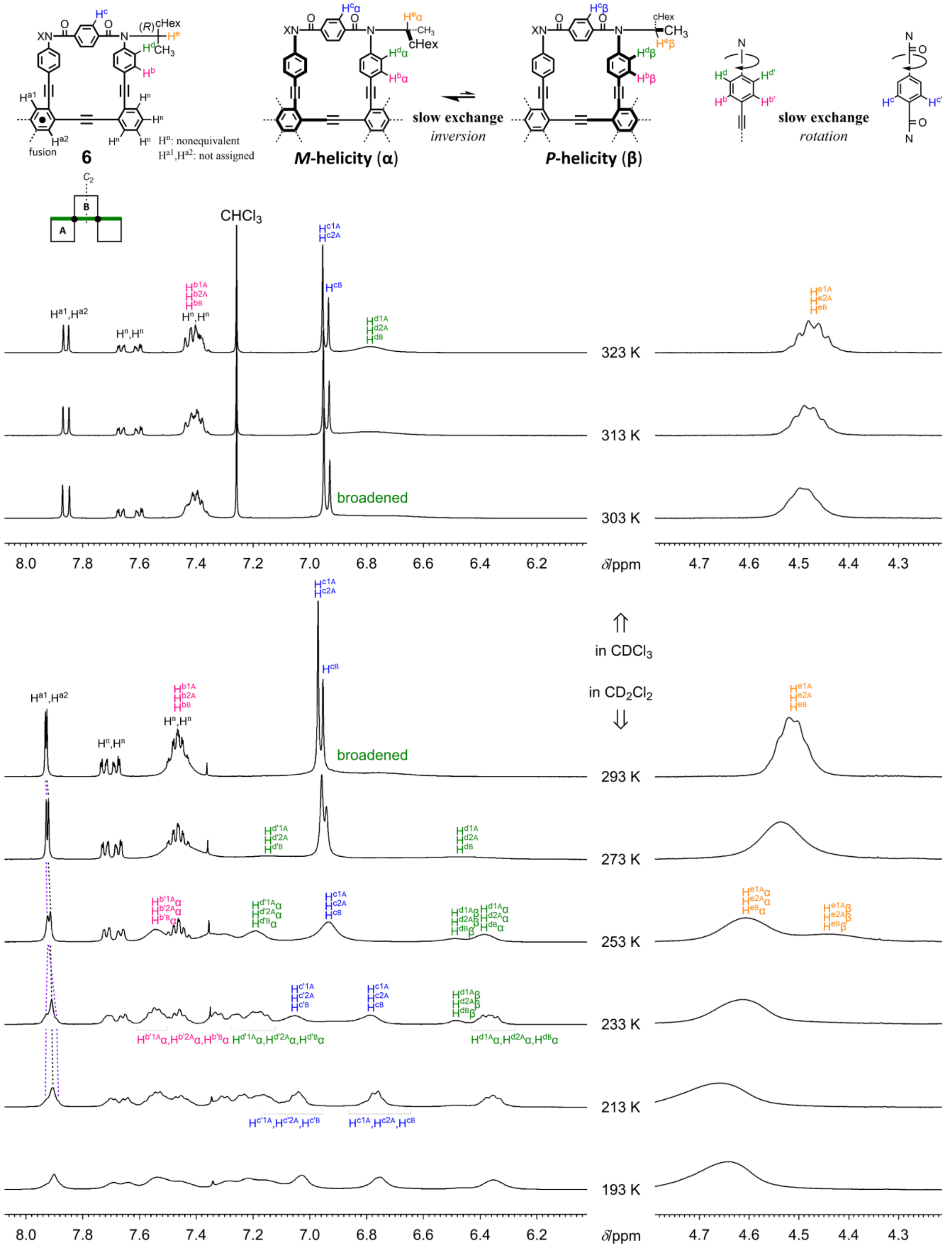

Fig. S17. Partial VT-^{1}H NMR spectra (400 MHz, left: aromatic protons and right: methine protons) of **6** (N = 3), measured in CD_2Cl_2 at 193-293 K and $CDCl_3$ at 303-323 K.

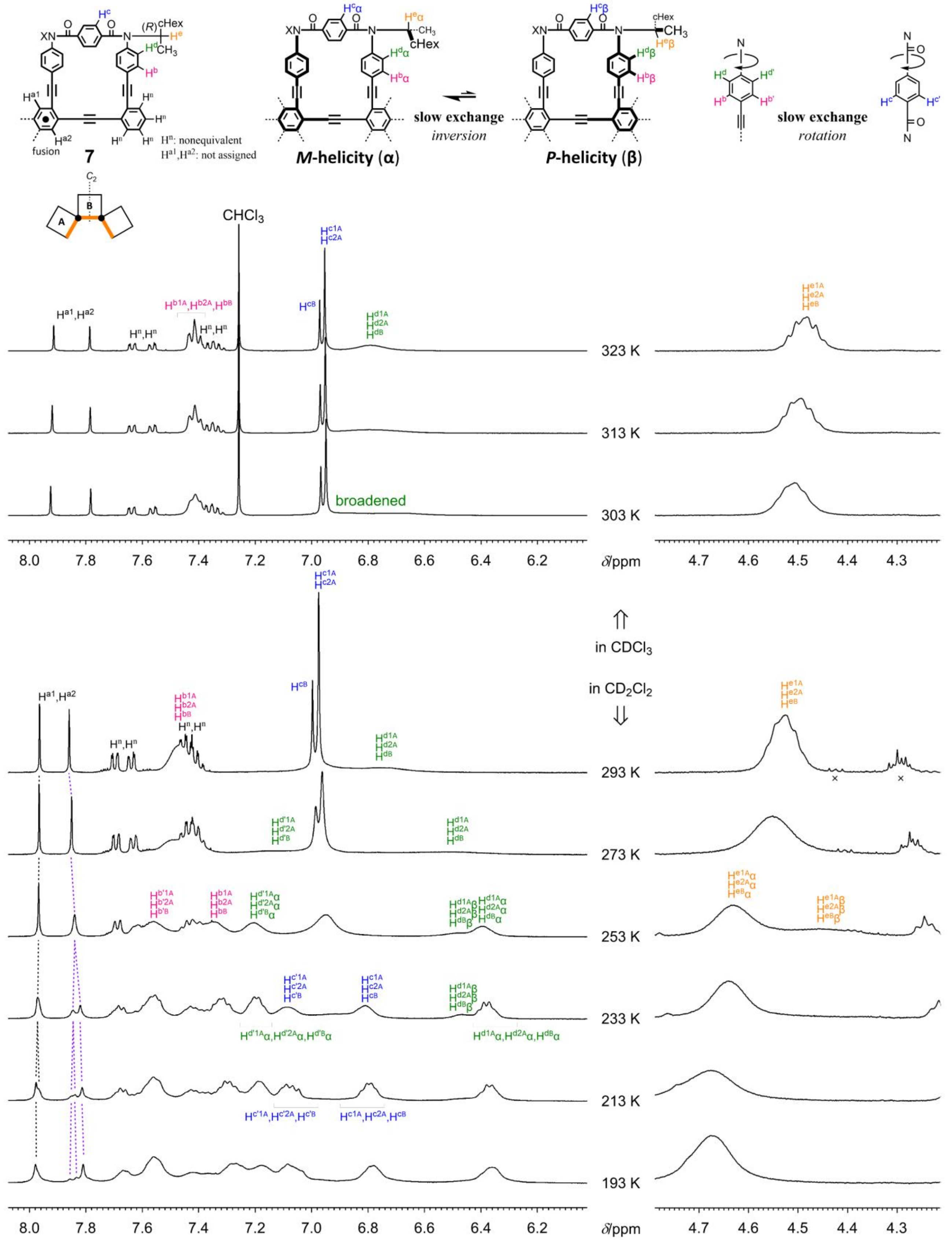

Fig. S18. Partial VT-^{1}H NMR spectra (400 MHz, left: aromatic protons and right: methine protons) of **7** (N = 3), measured in CD_2Cl_2 at 193-293 K and $CDCl_3$ at 303-323 K.

UV and CD spectra in the absence of an achiral guest.

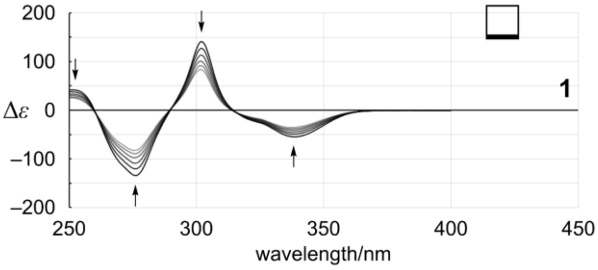

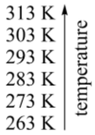

rotational arrangement

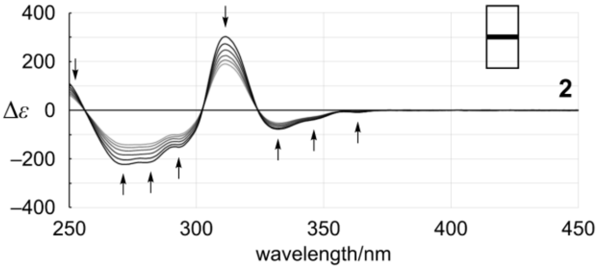

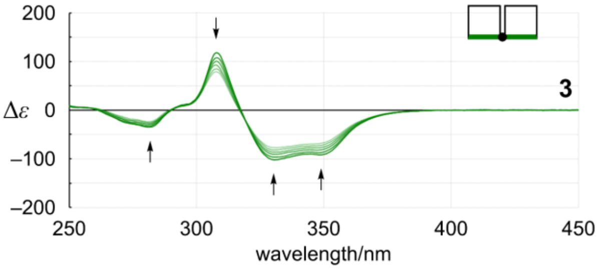

acyclic arrangement

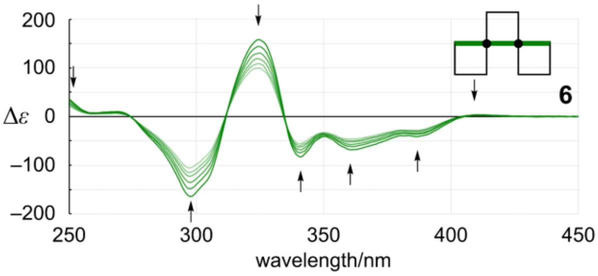

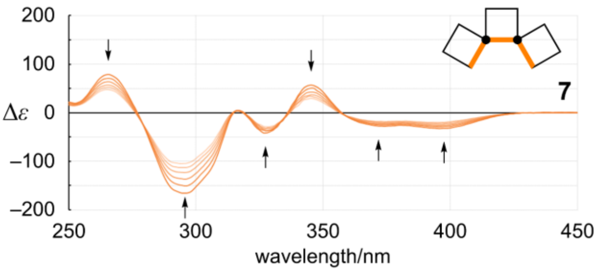

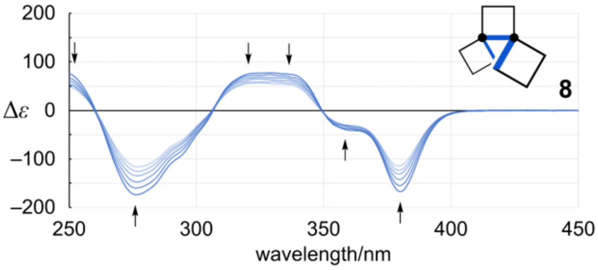

cyclic arrangement

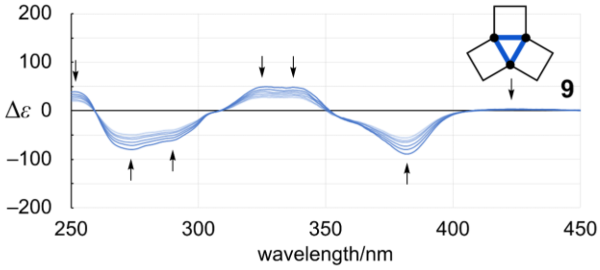

Fig. S19A. VT-CD spectra of **1**, **2**, **3**, **6**, **7**, **8**, and **9**, measured in CH_2Cl_2 at 263-313 K. Δε [L·mol^{-1}·cm^{-1}].

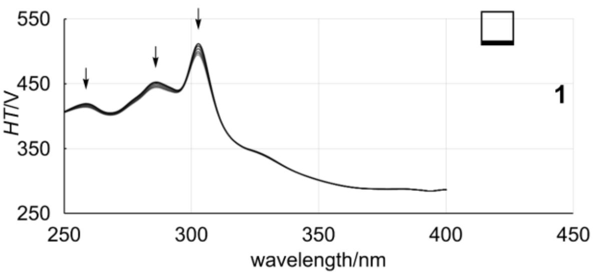

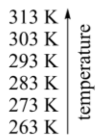

rotational arrangement

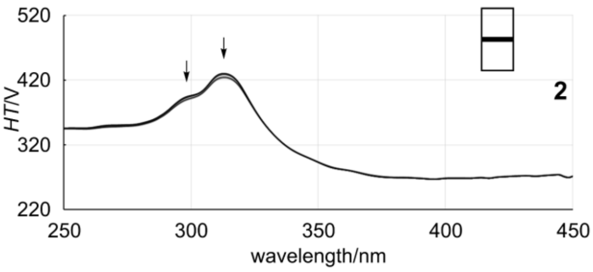

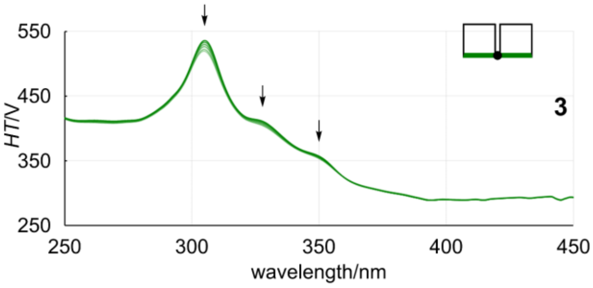

acyclic arrangement

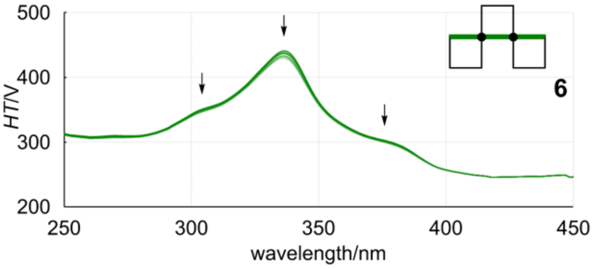

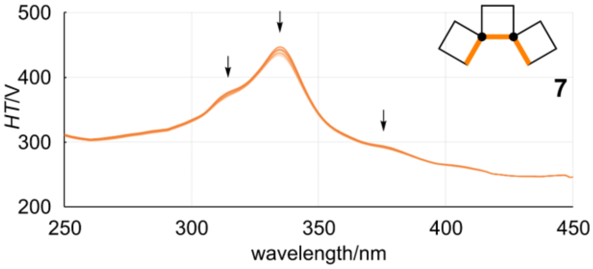

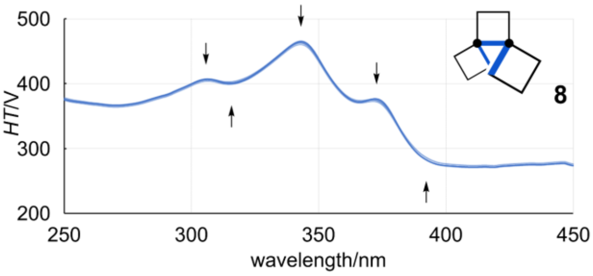

cyclic arrangement

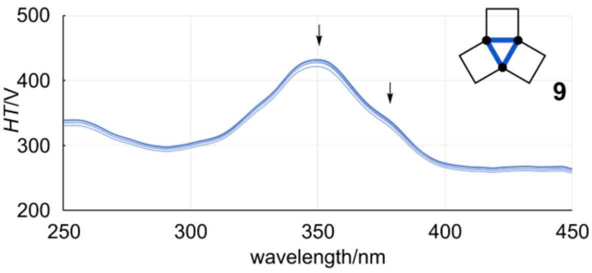

Fig. S19B. Changes of *HT*/V with temperature in a solution of **1**, **2**, **3**, **6**, **7**, **8**, or **9** for VT-CD measurements in CH_2Cl_2 (**Fig. S19A**). Cell length = 1 cm.

Absorption spectra of fused oligomers of macrocycles.

In terms of the electronic structure, the absorptions of most fused macrocycles (except for **3**) could not be explained in terms of a multiple of the absorption of **1** since new absorptions characteristic of 1,2,4,5-tetrakis(phenylethynyl)benzene[7] (**6**: 336 and 377 nm, **7**: 335 and 377 nm), 1,2,3,4-tetrakis(phenylethynyl)benzene[8,9] (**8**: 343 and 373 nm, **9**: 351 and ca. 380 nm) or hexakis(phenylethynyl)benzene[9,10] (**4**: 364 and ca. 387 nm, **5**: 368 and 390 nm) emerged in each spectrum (**Fig. S20**). In addition, the absorption end for **6** and **7** extended to 433 and 445 nm, respectively, which would be related to generating a longer *p*-PE by fusion of two 1,2,4,5-TPEBs. The intensity was greater for **7** than **6** due to a difference in coplanarity or linearity of the corresponding *p*-PE (*Cf*. **Fig. S5**). A similar extension due to the generation of longer *p*-PEs was seen for **10** and **11**. The spectrum of **8** would reflect the basis of model bis(macrocycle) **D**, although a longer *o*-PE was generated by a fusion of three macrocycles. In the spectrum of **3**, the spectral pattern for **1** seemed to remain in its entirety, while that almost disappeared in the spectrum of **9**.

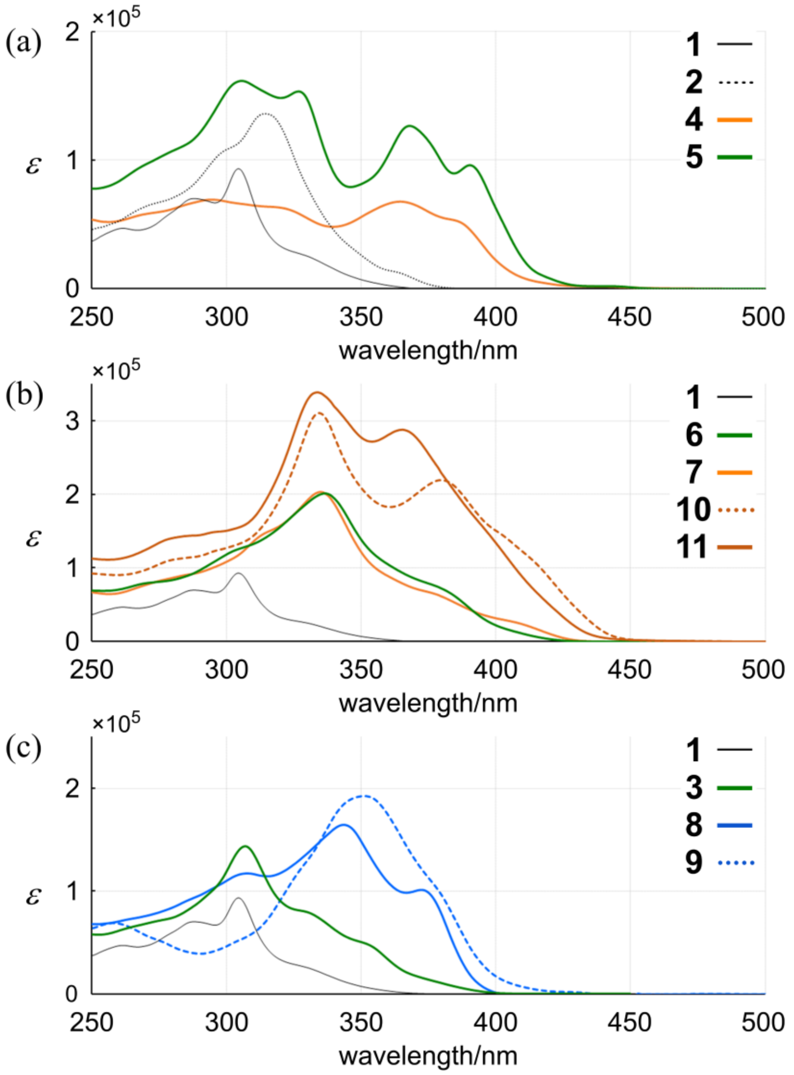

Fig. S20. UV spectra of (a) **1** (black solid line), **2** (black dotted line), **4** (HPEB, orange line), and **5** (HPEB, green line); (b) **1** (black line), **6** (1,2,4,5-TPEB, green line), **7** (1,2,4,5-TPEB, orange line), **10** (5PAM, brown dashed line) and **11** (6PAM, brown solid line); (c) **1** (black line), **3** (1,2,3,4-TPEB, green line), **8** (1,2,3,4-TPEB, blue solid line) and **9** ([12]DBA, blue dashed line). All spectra were measured in CH_2Cl_2 at room temperature. HPEB and TPEB: hexakis- and tetrakis(phenylethynyl)benzene, PAM: phenylacetylene macrocycle, DBA: dehydrobenzoannulene. ε [$L \cdot mol^{-1} \cdot cm^{-1}$].

---

7 K. Kondo, S. Yasuda, T. Sakaguchi and M. Miya, *J. Chem. Soc., Chem. Commun.*, 1995, 55.
8 K. Tahara, T. Yoshimura, M. Ohno, M. Sonoda and Y. Tobe, *Chem. Lett.*, 2007, **36**, 838.

---

9 R. Katoono and K. Arisawa, *RSC Adv.*, 2023, **13**, 11712.
10 R. Katoono, K. Kusaka, S. Kawai, Y. Tanaka, K. Hanada, T. Nehira, K. Fujiwara and T. Suzuki, *Org. Biomol. Chem.*, 2014, **12**, 9532.

Complexation [X = butyl] with a chiral guest $(S)_2$-14 or $(R)_2$-14.

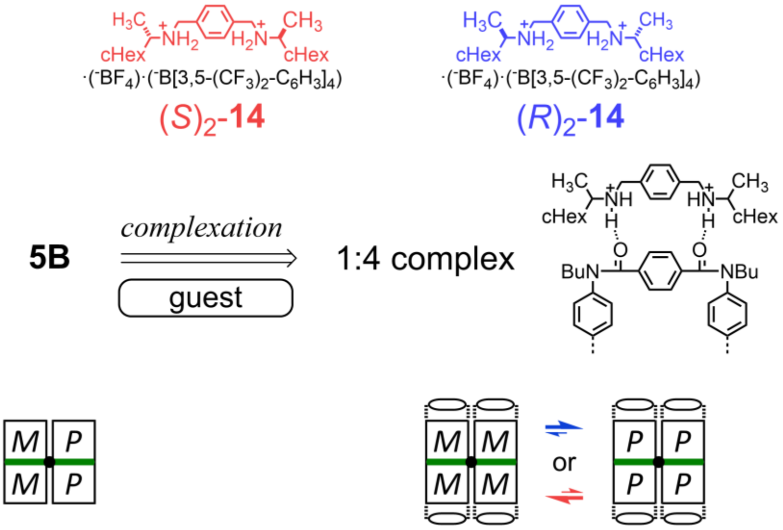

As mentioned above, a conformational search for model **5M** [X = methyl] (N = 4) predicted that only a particular heterochiral form (*MPMP*) was found out of other heterochiral forms (**Figs. S2** and **S3**). To investigate an innate conformational preference of the tetrakis(macrocycle) framework in solution, we implemented conformational analyses of **5B** [X = butyl] without any internal chiral source. Only a single set of resonances was found in the ^{1}H NMR spectra of **5B** measured at any temperature (**Fig. S11**). Based solely on this result, we could not assign the predominant species to which homochiral (*MMMM* and *PPPP*) or heterochiral (e.g., *MPMP*) conformers since a similar pattern could be obtained from either of these conformers. Instead, through complexation with either of enantiomeric guests, $(S)_2$-**14** and $(R)_2$-**14**, as an external chiral source, we examined whether or not homochiral conformers could be present in a complexed state and a particular sense could be preferred by an intermolecular transmission of point chirality (**Figs. S21-S24**).

In the absorption region of **5B** [λ_{max} 390 (log ε 4.98), 368 (5.10), 327 (5.19) and 306 (5.20)], which was similar in its entirety to that of **5** [390 (4.98), 368 (5.10), 327 (5.19) and 306 (5.21)] (**Fig. S20**), we found that several Cotton effects were induced by mixing with the chiral guest (**Fig. S23A**). The spectral appearance was consistent with that of **5** itself (**Fig. S10**). These results showed that homochiral conformers were present in a complex state, and a particular sense was preferred according to the external chirality in each guest. By a Job plot based on the complexation-induced Cotton effects (**Fig. S23B**), the stoichiometric ratio in a complex was determined to be 1:4. Sigmoidal titration curves emerged by plotting $\Delta\varepsilon$ values at several extreme wavelengths versus equivalents of the guest, which were curve-fitted with a program[11] to reveal that the fitting pattern was close to that for the complexation of **3B** rather than **1B** or **2B** (**Fig. S21**). If we consider that there was no change in the predominant conformation of **1B** or **2B** (*Cf.* **Fig. S1**) before and after complexation with a chiral guest, the complexation of **5B** (and **3B**) accompanied some change in the population of conformers. The intensity of complexation-induced Cotton effects in

---

11 S. Akine, TitrationFit, a program for analyses of host-guest complexation, Kanazawa University, Kanazawa, Japan, 2013.

the presence of $(R)_2$-**14** (16 equiv.) changed with temperature (**Figs. S23A** and **S23C**), like the case of **5** itself (**Figs. S10** and **S23C**), which indicated that there was an equilibrium between two diastereomeric complexes with homochiral conformations (*MMMM*-**5B** and *PPPP*-**5B**). Through this CD titration experiment, we obtained an imaginary value of $\Delta\varepsilon$ ($|\Delta\varepsilon_{100\%\text{complex}}|$, where all of the molecules of **5B** were assumed to be in a complexed state [*MMMM*-**5B**·$(R)_2$-**14** and *PPPP*-**5B**·$(R)_2$-**14**]) by a curve-fitting method (**Fig. S21**). According to the $|\Delta\varepsilon_{100\%\text{complex}}|$ (254.9±7.7), the imaginary value of diastereomeric excess induced in a complex was calculated to be 36.3±1.1%. The presence of two species in a complexed state was also confirmed in the ^{1}H NMR spectrum of **5B** in the presence of $(R)_2$-**14** (**Fig. S23A**). By mixing the host and guest, significant upfield shifts were induced for the central phenylene protons (both H^c in the host and H^A in the guest) to demonstrate that the guest was captured at the terephthaloyl bridge. It should be noted that complexation-induced changes in the chemical shift were observed even for peripheral aromatic protons H^{a1} and H^{a2} in the host, located far from the binding site. These complexation-induced shifts were considered not to be due to the proximity of the host and guest but suggested that the complexation accompanied some change in conformation [this was also confirmed with **3B** (**Fig. S22**) and **8B** (**Fig. S24**)]. From these results, we concluded that a heterochiral conformation (*MPMP*) was predominantly present in a solution of **5B** itself (uncomplexed state). This result represented an example of a complexation-induced change in the population of conformers (*Cf.* **Scheme 6** in the main text).

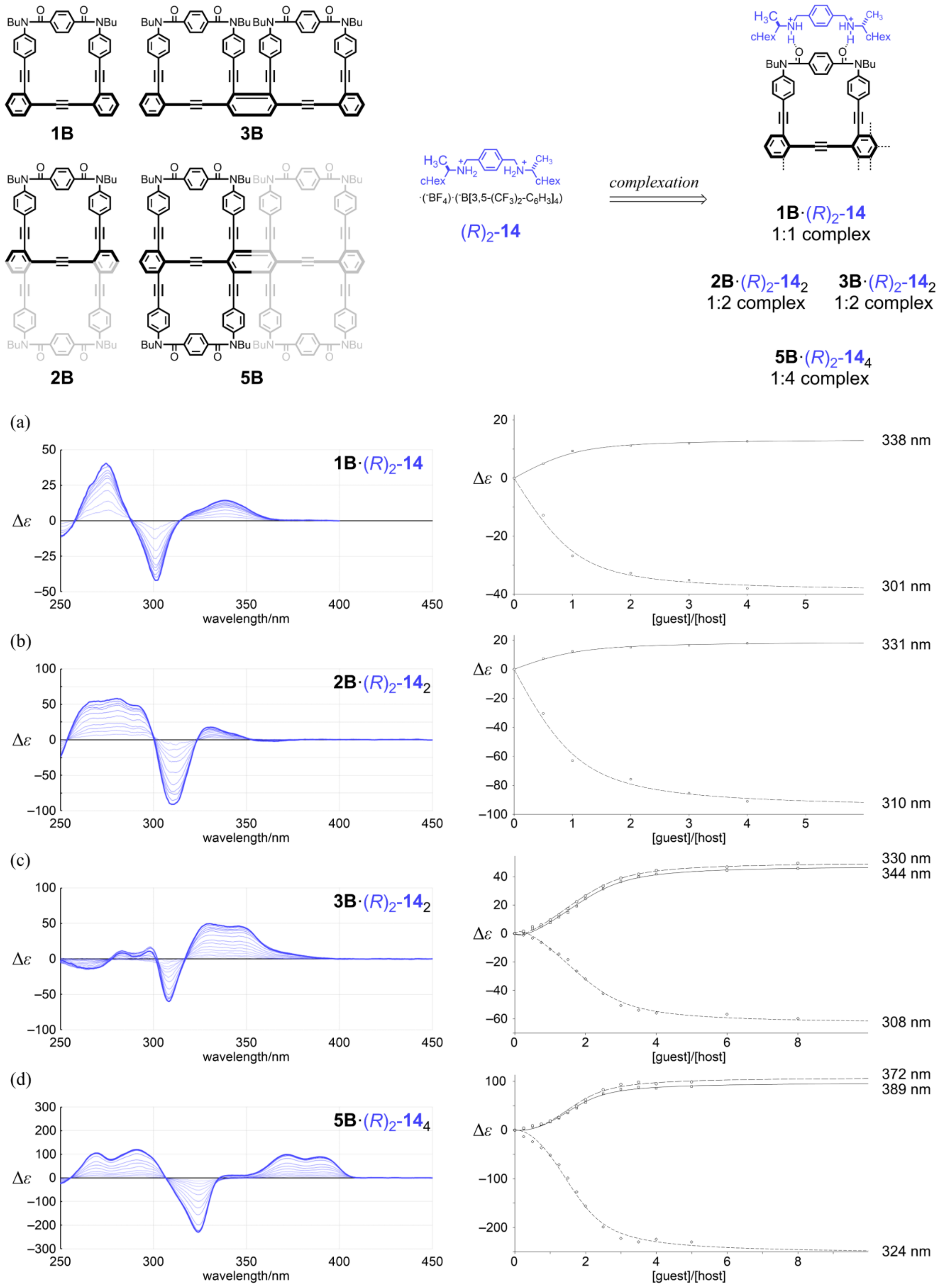

Fig. S21. [left] CD spectra of (a) **1B** (2.3×10^{-4} M) in the presence of (R)$_2$-**14** (0.25, 0.5, 0.75, 1, 1.5, 2, 3, 4, 6 and 8

equiv.); (b) **2B** (1.3×10^{-4} M) in the presence of (*R*)$_2$-**14** (0.25, 0.5, 0.75, 1, 1.5, 2, 3, 4, 6 and 8 equiv.); (c) **3B** (1.3×10^{-4} M) in the presence of (*R*)-**14** (0.25, 0.5, 0.75, 1, 1.25, 1.5, 1.75, 2, 2.5, 3, 3.5, 4, 6 and 8 equiv.); (d) **5B** (7.4×10^{-5} M) in the presence of (*R*)-**14** (0.5, 1, 1.5, 2, 2.5, 3, 3.5, 4, 5, 6, 7, 8 and 10 equiv.). [right] Titration curves 1:1- or 1:2-fitted with a program of TitrationFit software[11], based on plots of complexation-induced molar CDs ($\Delta\varepsilon$) versus equivalents of (*R*)$_2$-**14**. (a) **1B** ([host] = [**1B**], 1:1-fitting) [$\Delta\varepsilon$ at 301 nm (circle) and 338 nm (square)]; (b) **2B** ([host] = 2 × [**2B**], 1:1-fitting) [$\Delta\varepsilon$ at 310 nm (circle) and 331 nm (square)]; (c) **3B** ([host] = [**3B**], 1:2-fitting) [$\Delta\varepsilon$ at 308 nm (diamond), 330 nm (circle) and 344 nm (square)]; (d) **5B** ([host] = 2 × [**5B**], 1:2-fitting) [$\Delta\varepsilon$ at 324 nm (diamond), 372 nm (circle) and 389 nm (square)]. All spectra were measured in CH_2Cl_2 at 293 K.

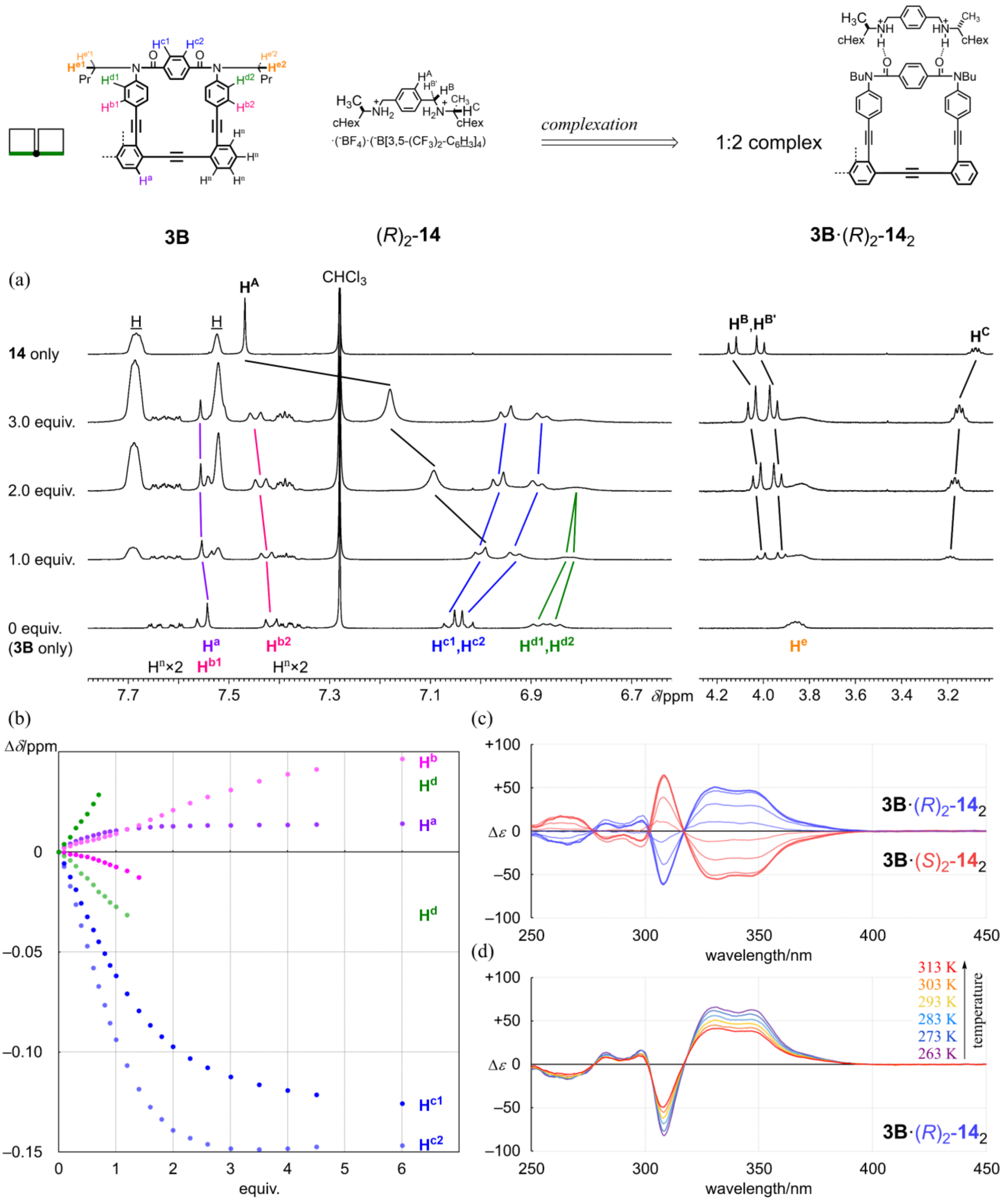

Fig. S22A. (a) ^{1}H NMR spectra of **3B** (1.0 mM) in the presence of (R)$_2$-**14** [0 (**3B** only), 1, 2 and 3 equiv.], and ^{1}H NMR spectrum of (R)$_2$-**14**, measured in $CDCl_3$ containing 3 vol% CD_3CN at 303 K ($CHCl_3$ δ 7.28 ppm); (b) plots of complexation-induced changes in the chemical shift ($\Delta\delta$/ppm = $\delta_{3B\cdot 14}$–δ_{3B}) versus equivalents of (R)$_2$-**14**; (c) CD spectra of **3B** (1.3 × 10^{-4} M) in the presence of (R)$_2$-**14** [1, 2, 4 and 8 equiv. (blue lines)] or (S)$_2$-**14** [1, 2, 4 and 8 equiv. (red lines)], measured in CH_2Cl_2 at 293 K; (d) VT-CD spectra of **3B** in the presence of (R)$_2$-**14** (8 equiv.), measured in CH_2Cl_2 at 263-313 K.

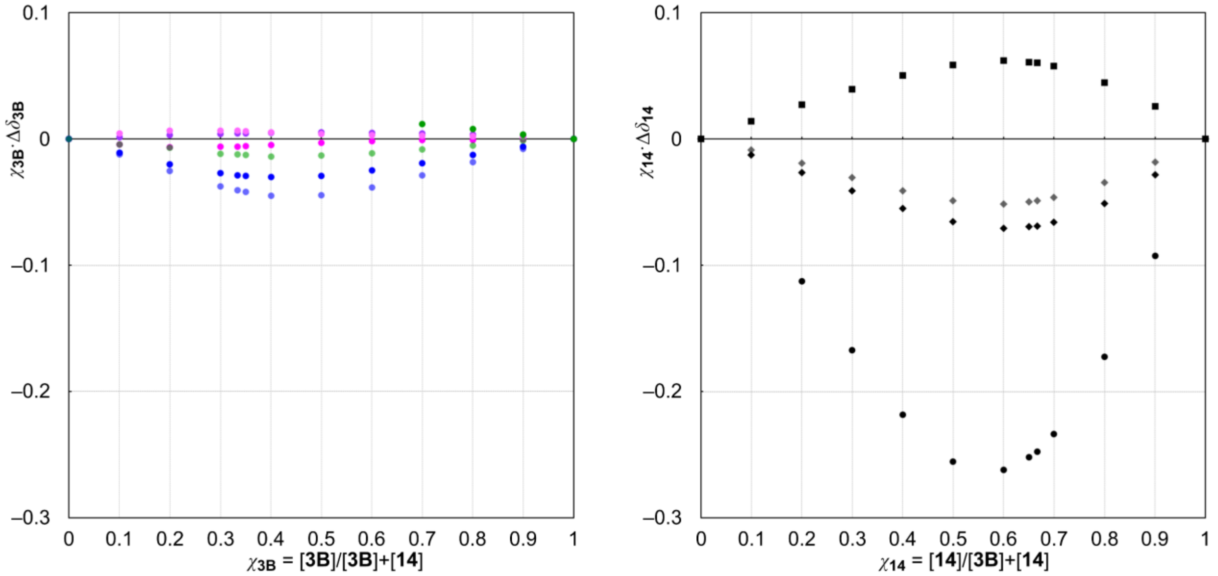

Fig. S22B. Job plots for complexation of **3B** with (R)$_2$-**14** ([**3B**] + [**14**] = 2.0 mM), based on complexation-induced changes in the chemical shift ([left] $\Delta\delta_{\mathbf{3B}}$/ppm = $\delta_{\mathbf{3B}\cdot\mathbf{14}}$–$\delta_{\mathbf{3B}}$ and [right] $\Delta\delta_{\mathbf{14}}$/ppm = $\delta_{\mathbf{3B}\cdot\mathbf{14}}$–$\delta_{\mathbf{14}}$), measured in $CDCl_3$ containing 3 vol% CD_3CN at 303 K.

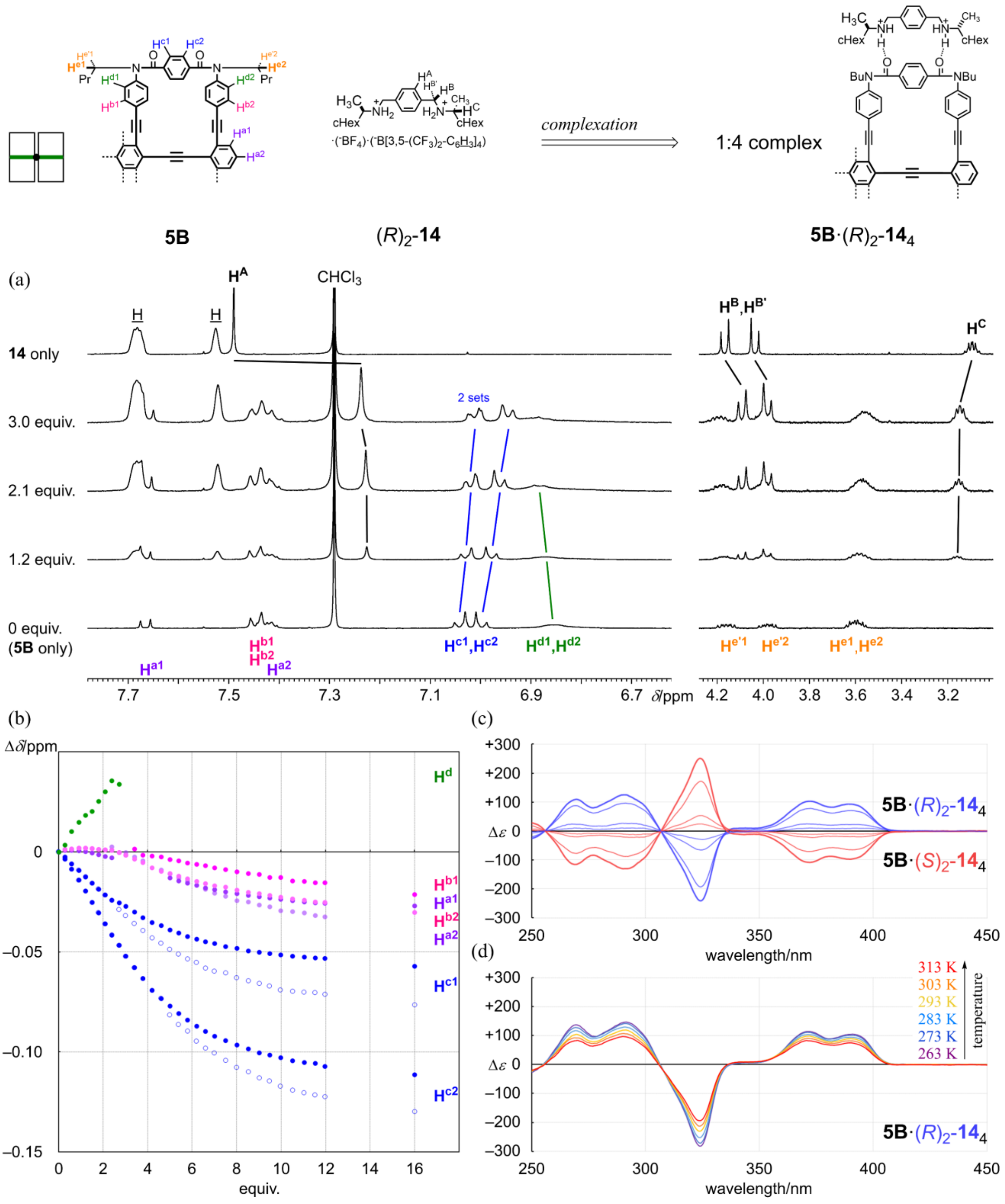

Fig. S23A. (a) ^{1}H NMR spectra of **5B** (0.50 mM) in the presence of (R)$_2$-**14** [0 (**5B** only), 1.2, 2.1 and 3.0 equiv.], and ^{1}H NMR spectrum of (R)$_2$-**14**, measured in $CDCl_3$ containing 4.5 vol% CD_3CN at 303 K ($CHCl_3$ δ 7.29 ppm); (b) plots of complexation-induced changes in the chemical shift ($\Delta\delta$/ppm = $\delta_{\mathbf{5B\cdot14}}$–$\delta_{\mathbf{5B}}$) versus equivalents of (R)$_2$-**14**; (c) CD spectra of **5B** (7.4×10^{-5} M) in the presence of (R)$_2$-**14** [1, 2, 4 and 8 equiv. (blue lines)] or (S)$_2$-**14** [1, 2, 4 and 8 equiv. (red lines)], measured in CH_2Cl_2 at 293 K; (d) VT-CD spectra of **5B** in the presence of (R)$_2$-**14** (16 equiv.), measured in CH_2Cl_2 at 263-313 K.

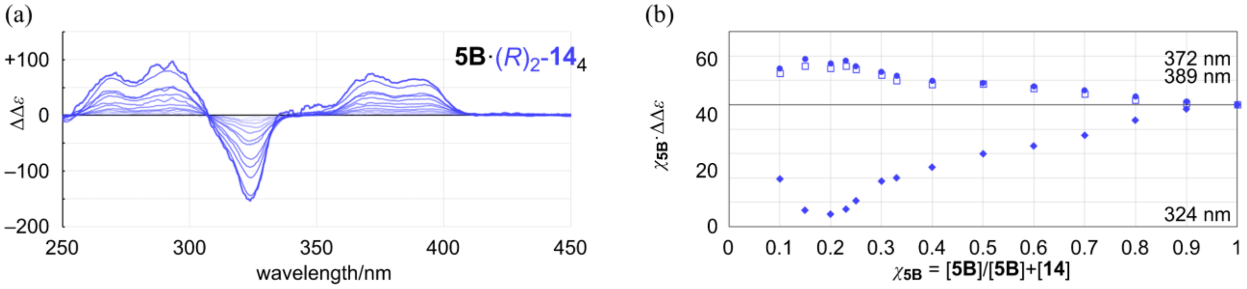

Fig. S23B. (a) CD spectra of **5B** in the presence of $(R)_2$-**14** ([**5B**] + [**14**] = 1.8×10^{-4} M); (b) Job plots for complexation of **5B** with $(R)_2$-**14**, based on changes in the molar CD ($\Delta\Delta\varepsilon = \Delta\varepsilon_{\mathbf{5B}\cdot\mathbf{14}}$) at 324, 372 and 389 nm. All spectra were measured in CH_2Cl_2 at 293 K.

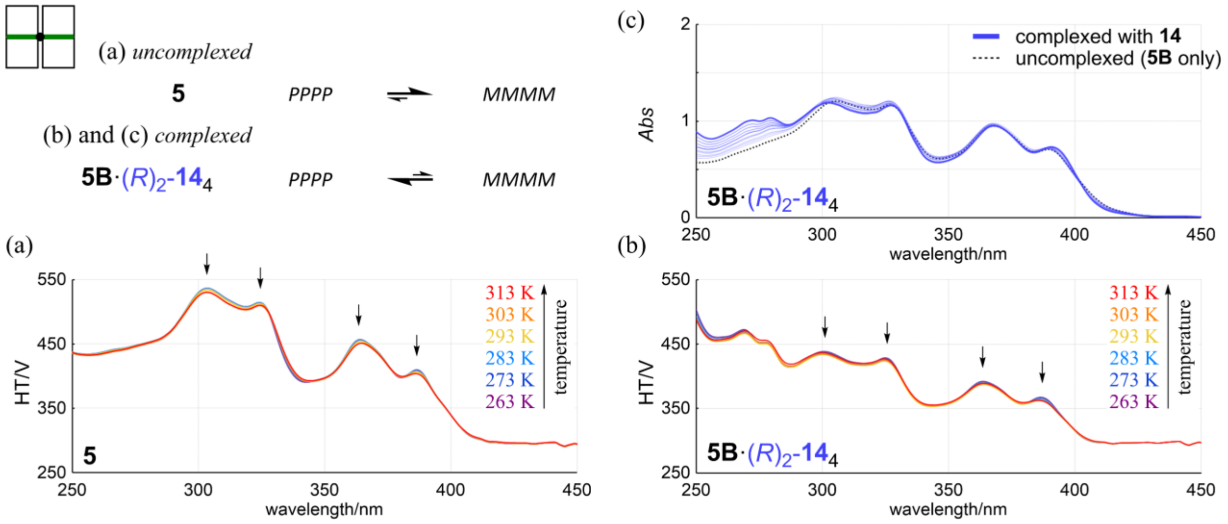

Fig. S23C. Changes of *HT*/V with temperature in a solution of (a) **5** or (b) **5B** (7.4×10^{-5} M) in the presence of $(R)_2$-**14** (16 equiv.) for VT-CD measurements in CH_2Cl_2; (c) UV spectra of **5B** (7.4×10^{-5} M) in the presence of (R)**-14** (0.5, 1, 1.5, 2, 2.5, 3, 3.5, 4, 5, 6 and 7 equiv.), measured in CH_2Cl_2 at room temperature. (a) Cell length = 1 cm. (b) and (c) Cell length = 0.1 cm.

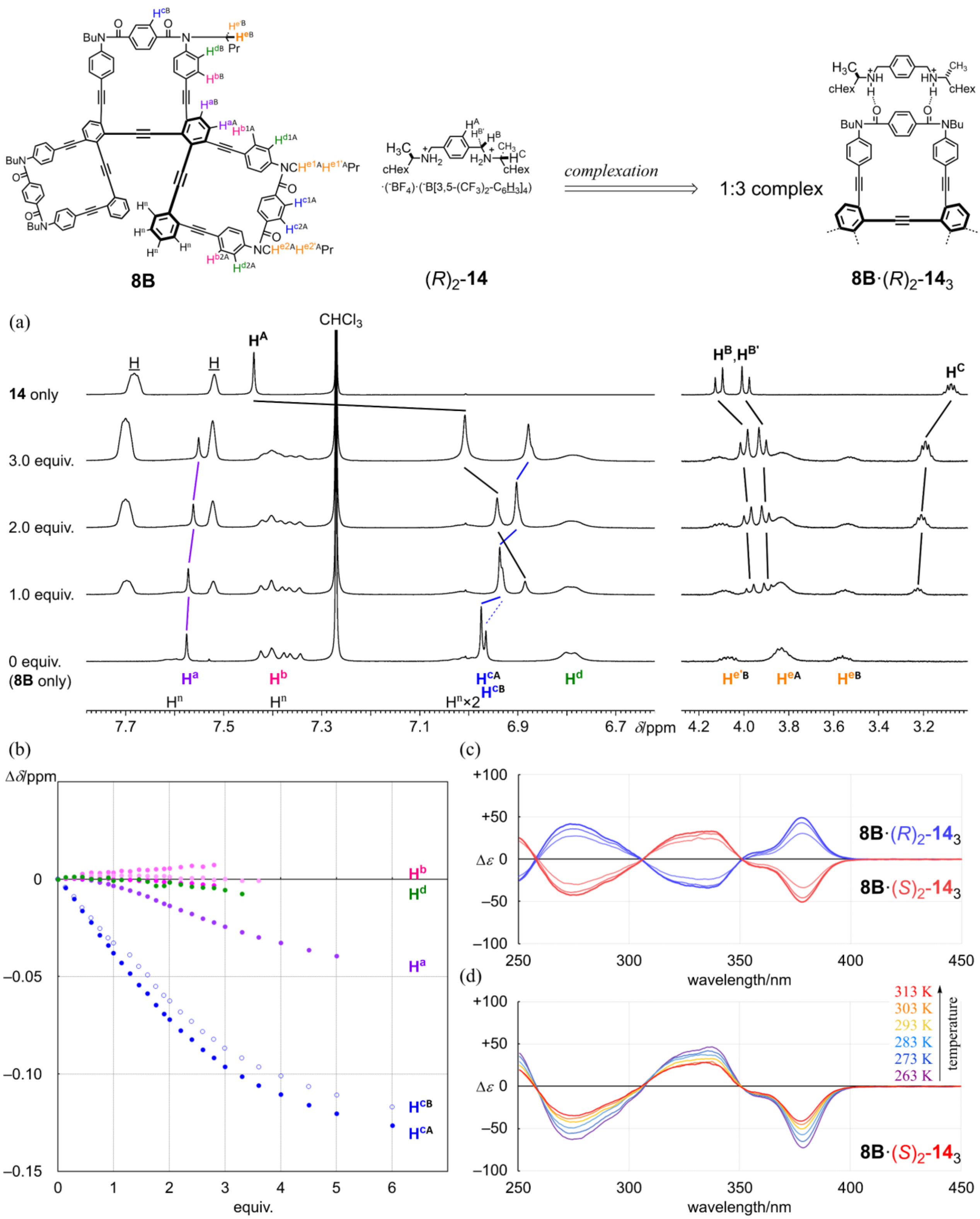

Fig. S24A. (a) ^{1}H NMR spectra of **8B** (0.67 mM) in the presence of (R)$_2$-**14** [0 (**8B** only), 1, 2 and 3 equiv.], and ^{1}H NMR spectrum of (R)$_2$-**14**, measured in $CDCl_3$ containing 1.7 vol% CD_3CN at 303 K ($CHCl_3$ δ 7.27 ppm); (b) plots of complexation-induced changes in the chemical shift ($\Delta\delta$/ppm = $\delta_{8B\cdot14}$–δ_{8B}) versus equivalents of (R)$_2$-**14**; (c) CD spectra of **8B** (1.0×10^{-4} M) in the presence of (R)$_2$-**14** [3, 6 and 9 equiv. (blue lines)] or (S)$_2$-**14** [3, 6 and 9 equiv. (red lines)], measured in CH_2Cl_2 at 293 K; (d) VT-CD spectra of **8B** in the presence of (S)$_2$-**14** (9 equiv.), measured in CH_2Cl_2 at 263-313 K.

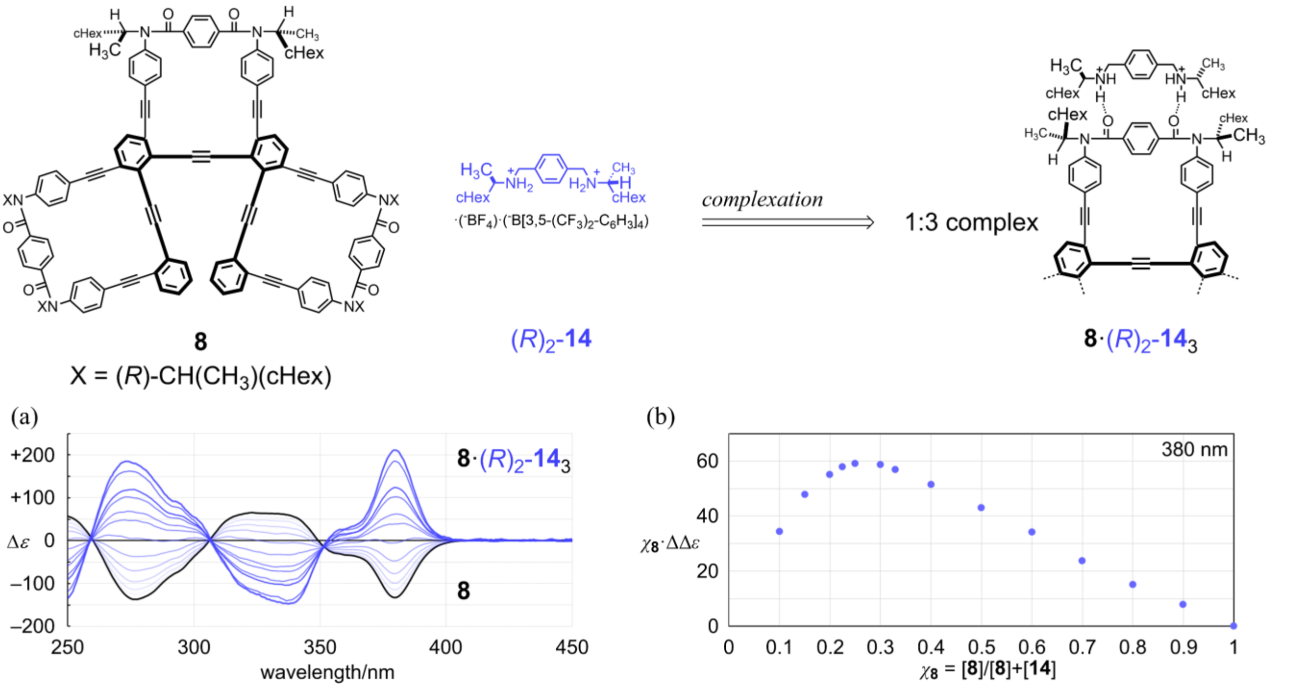

Fig. S24B. (a) CD spectra of **8** in the presence of (R)$_2$-**14** ([**8**] + [**14**] = 1.3 × 10^{-4} M); (b) a Job plot for complexation of **8** with (R)$_2$-**14**, based on changes in the molar CD ($\Delta\Delta\varepsilon = \Delta\varepsilon_{\mathbf{8}\cdot\mathbf{14}} - \Delta\varepsilon_{\mathbf{8}}$) at 380 nm. All spectra were measured in CH_2Cl_2 at 293 K.

UV and CD spectra in the presence of achiral guest (13).

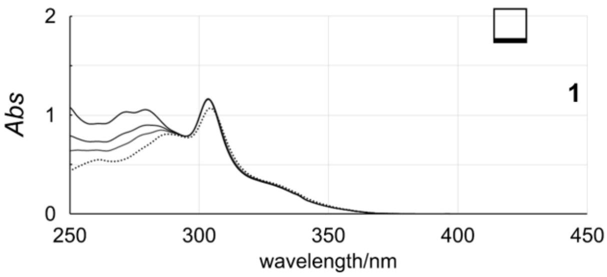

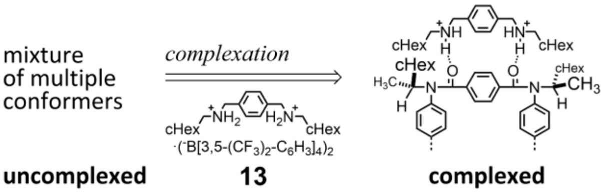

rotational arrangement

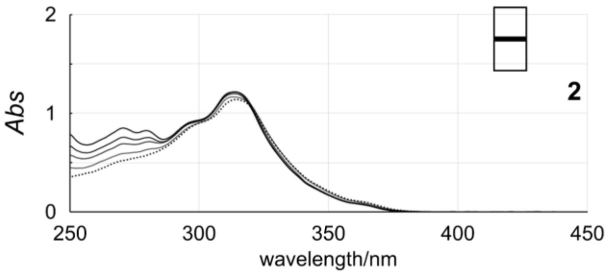

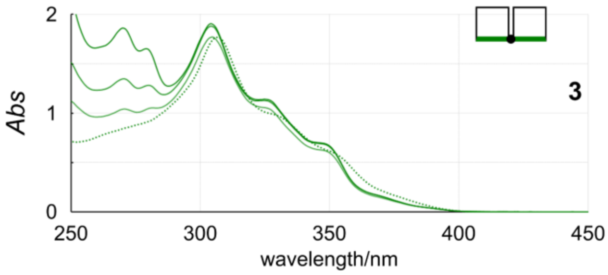

acyclic arrangement

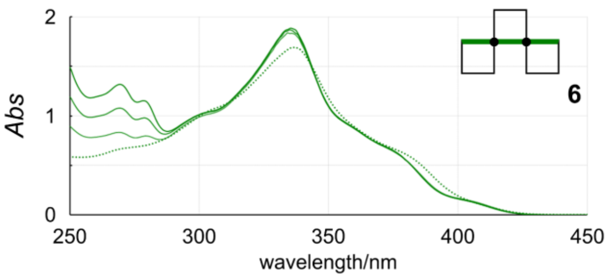

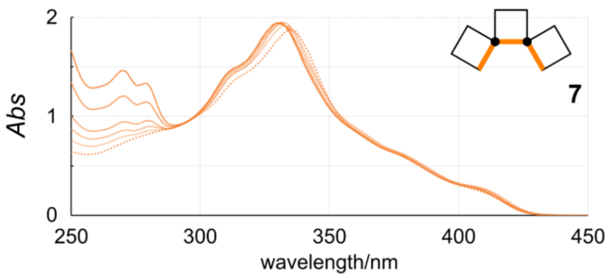

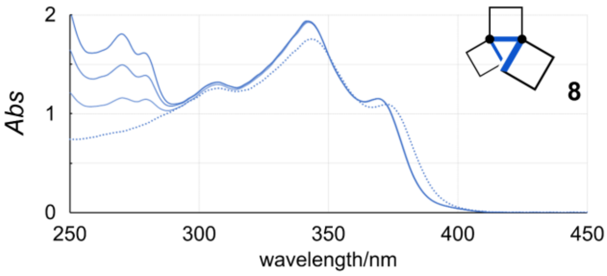

cyclic arrangement

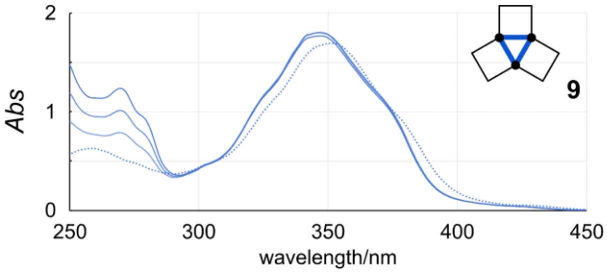

Fig. S25. UV spectra of **1** (1.14×10^{-4} M), **2** (8.37×10^{-5} M), **3** (1.23×10^{-4} M), **6** (8.42×10^{-5} M), **7** (9.22×10^{-5} M), **8** (1.07×10^{-4} M) and **9** (7.78×10^{-5} M) in the presence of **13** [0 equiv. (**1**, **2**, **3**, **6**, **7**, **8**, or **9** only, dashed line); 1, 2 and 4 equiv. for **1**; 1, 2, 3 and 4 equiv. for **2**; 2, 4 and 8 equiv. for **3**; 3, 6 and 9 equiv. for **6**, **8**, and **9**; 1, 2, 3, 6 and 9 equiv. for **7** (solid lines)]. All spectra were measured in CH_2Cl_2 at room temperature. Cell length = 0.1 cm.

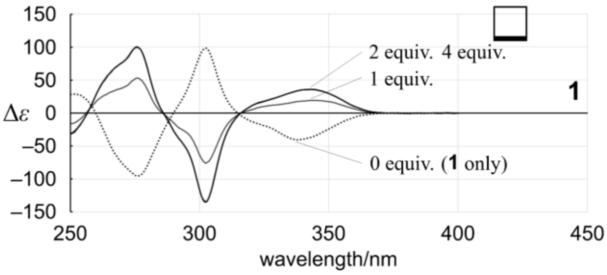

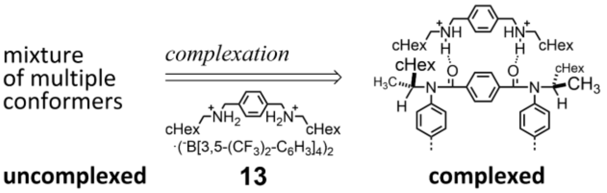

rotational arrangement

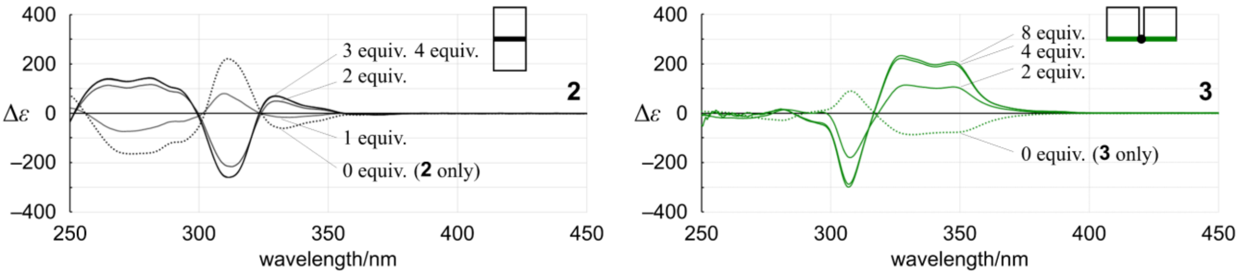

acyclic arrangement

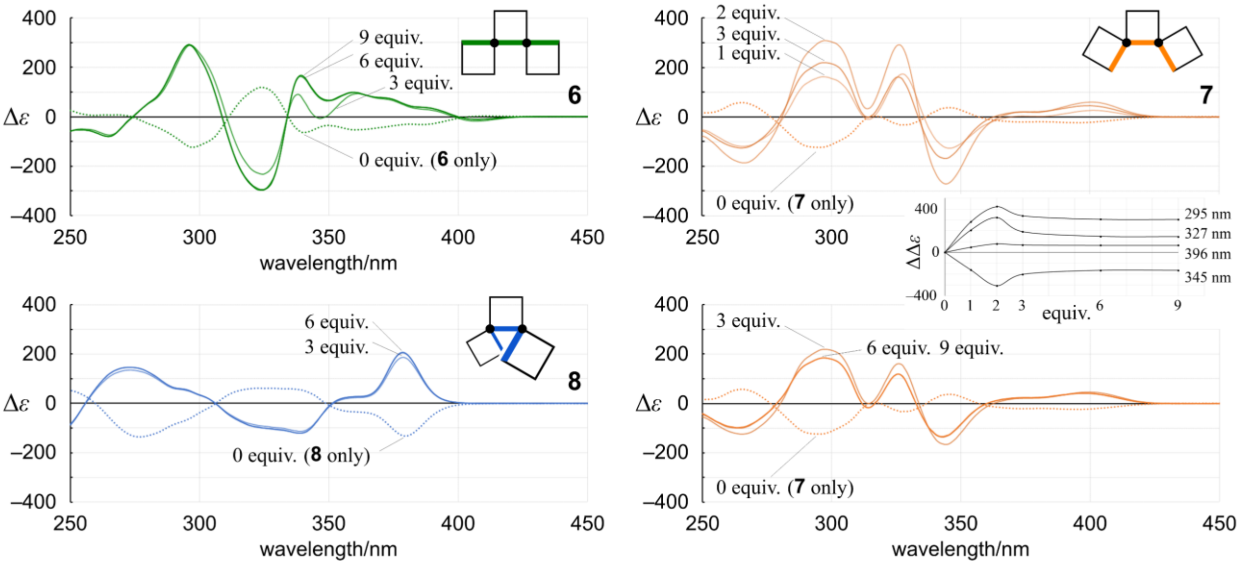

cyclic arrangement

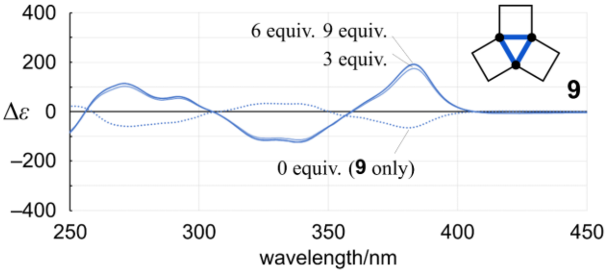

Fig. S26. CD spectra of **1** (1.14×10^{-4} M), **2** (8.37×10^{-5} M), **3** (1.23×10^{-4} M), **6** (8.42×10^{-5} M), **7** (9.22×10^{-5} M), **8** (1.07×10^{-4} M) and **9** (7.78×10^{-5} M) in the presence of **13** [0 equiv. (**1**, **2**, **3**, **6**, **7**, **8**, or **9** only, dashed line); 1, 2 and 4 equiv. for **1**; 1, 2, 3 and 4 equiv. for **2**; 2, 4 and 8 equiv. for **3**; 3, 6 and 9 equiv. for **6**, **8**, and **9**; 1, 2, 3, 6 and 9 equiv. for **7** (solid lines)]. Inset: changes in molar CD ($\Delta\Delta\varepsilon = \Delta\varepsilon_{7\cdot13} - \Delta\varepsilon_{7}$) with equivalents of **13**. All spectra were measured in CH_2Cl_2 at 293 K. $\Delta\varepsilon$ [$L \cdot mol^{-1} \cdot cm^{-1}$].

Plots of Δε/ε before and after complexation (13).

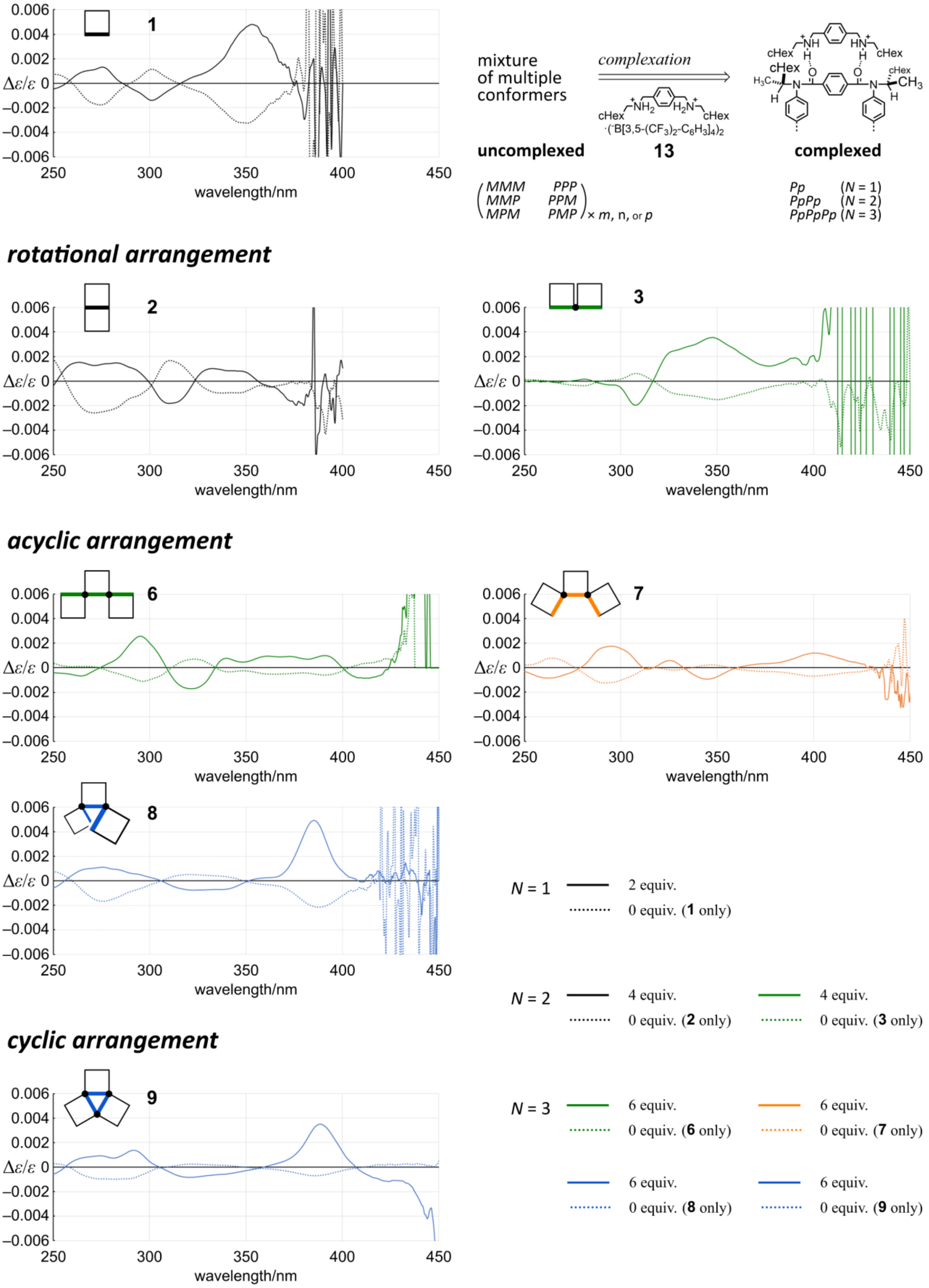

Fig. S27. Plots of Δε/ε for **1**, **2**, **3**, **6**, **7**, **8**, and **9** versus wavelength in the presence (solid line) or absence (dashed line) of **13**.

Experimental

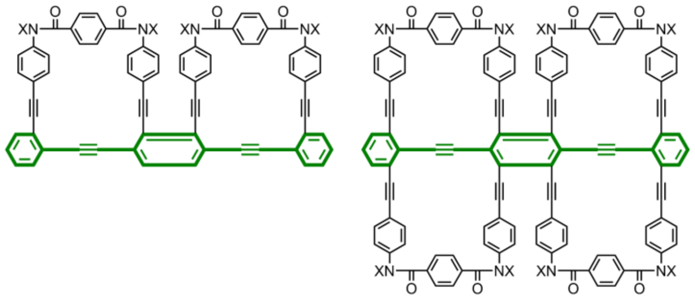

3/3B **5/5B**

X = (*R*)-CH(CH$_3$)(cHex)
B: X = *n*Bu

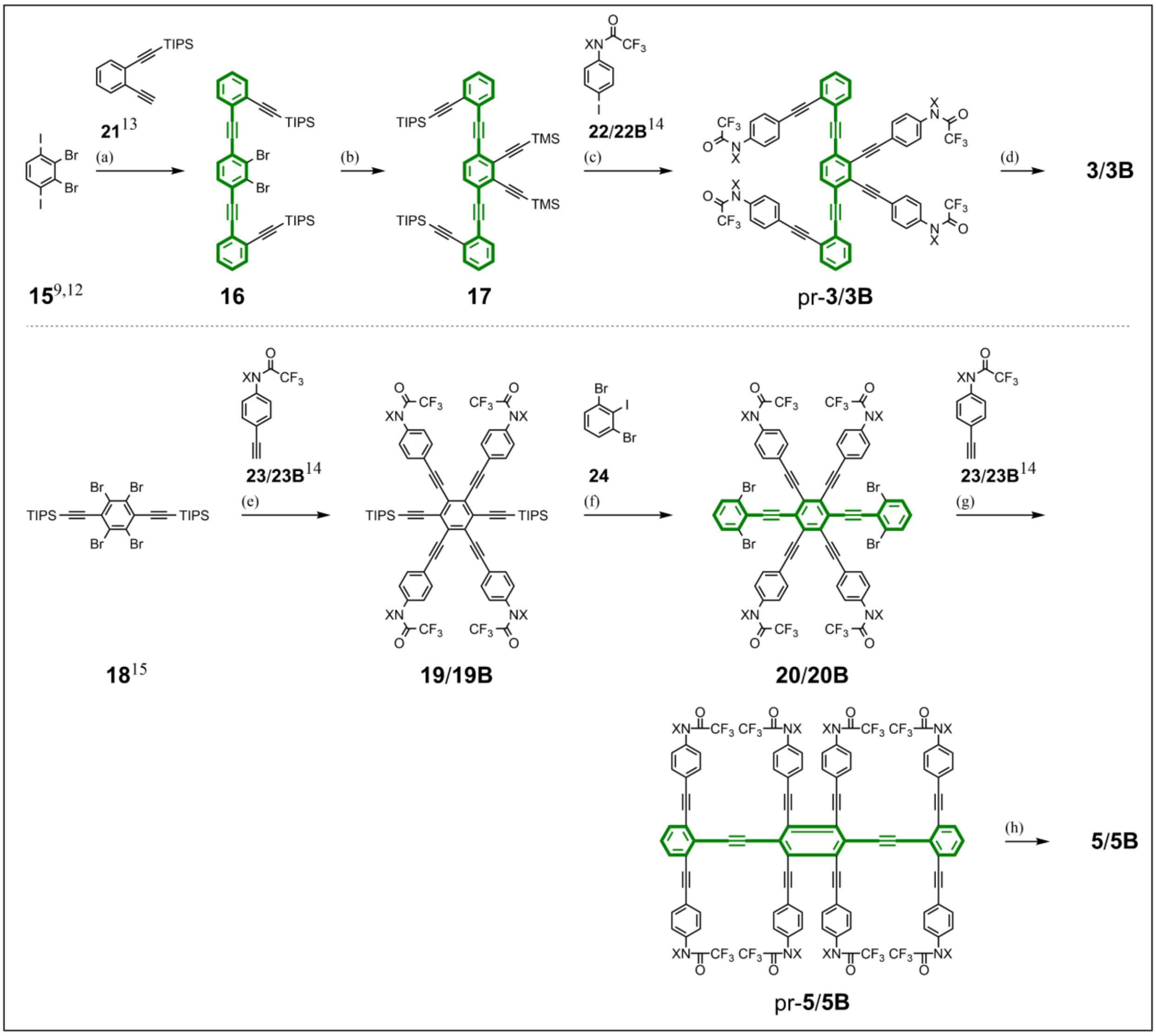

Scheme S1. Synthesis of bis(macrocycle)s **3**/**3B** and tetrakis(macrocycle)s **5**/**5B**. Reagents and yields: (a) **21**, Pd(PPh$_3$)$_4$, CuI, Et$_3$N (**16**: 92%); (b) trimethylsilylacetylene (TMSA), Pd(PPh$_3$)$_4$, CuI, Et$_3$N (**17**: 67%); (c) **22**/**22B**, Pd(PPh$_3$)$_4$, CuI, tetrabutylammonium fluoride (TBAF), Et$_3$N, THF (76% for pr-**3**/73% for pr-**3B**); (d) i) NaH, MeOH, THF (98% for pr-**3'**/96% for pr-**3B'**), ii) terephthaloyl chloride, Et$_3$N, toluene (78% for **3**/65% for **3B**); (e) **23**/**23B**, Pd(PPh$_3$)$_4$, CuI, Et$_3$N (80% for **19**/70% for **19B**); (f) i) TBAF, THF (69% for **19'**/84% for **19B'**), ii) **24**, Pd(PPh$_3$)$_4$, CuI, Et$_3$N, benzene (56% for **20**/51% for **20B**); (g) **23**/**23B**, Pd(PPh$_3$)$_4$, CuI, Et$_3$N, THF (25% for pr-**5**/43% for pr-**5B**); (h) i) NaH, MeOH, THF (77% for pr-**5'**/79% for pr-**5B'**), ii) terephthaloyl chloride, Et$_3$N, toluene (54% for **5**/53% for **5B**).

(a) Preparation of **16**

To a solution of **15**[9,12] (750 mg, 1.54 mmol) and **21**[13] (886 mg, 3.14 mmol) in Et_3N (32 mL) were added $Pd(PPh_3)_4$ (94 mg, 0.081 mmol) and CuI (34 mg, 0.18 mmol) at room temperature under an argon atmosphere, and the mixture was stirred at 50 °C for 2.5 h. After removing a solid by filtration through a Celite pad, the filtrate was concentrated by evaporation, and the residue was purified by column chromatography on SiO_2 (dichloromethane/hexane) to give **16** (1.13 g) as a pale yellow solid in 92% yield. An analytical sample was obtained as colorless crystals by purification through a SiO_2 column (dichloromethane/hexane) and GPC (chloroform; JAIGEL-1H & 2H, Japan Analytical Industry Co., Ltd., Japan), followed by recrystallization from ethanol. **16**: mp 112-114 °C; elemental analyses Found: C, 66.20; H, 6.53%. Calcd for $C_{44}H_{52}Br_2Si_2$: C, 66.32; H, 6.58%; ^{1}H NMR δ_H(400 MHz; $CDCl_3$; Me_4Si)/ppm 7.61-7.56 (2H, m), 7.56-7.52 (2H, m), 7.45 (2H, s), 7.35-7.29 (4H, m), 1.14-1.10 (42H, m); ^{13}C NMR δ_C(100 MHz; $CDCl_3$)/ppm 132.9, 132.3, 131.1, 128.6, 128.6, 128.1, 127.1, 125.9, 125.0, 105.1, 95.6, 94.7, 91.8, 18.7, 11.3; FD-LRMS *m/z* 794.1 (M^+, 46%), 795.1 ($[M+1]^+$, 27), 796.1 ($[M+2]^+$, 100), 797.1 ($[M+3]^+$, 57), 798.1 ($[M+4]^+$, 66), 799.1 ($[M+5]^+$, 33), 800.1 ($[M+6]^+$, 12).

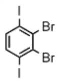

15

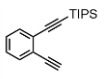

21

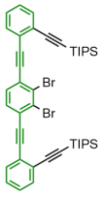

16

(b) Preparation of **17**

To a solution of **16** (601 mg, 0.754 mmol) in Et_3N (15 mL) were added $Pd(PPh_3)_4$ (44 mg, 0.038 mmol) and CuI (17 mg, 0.089 mmol) at room temperature under an argon atmosphere, and the mixture was stirred at 80 °C. To the warmed-up solution were added two portions of TMSA (first 2.2 + additional 0.50 mL, 16 + 3.5 mmol), and the mixture was stirred at that temperature for (first) 15 h and (additional) 9 h. After removing a solid by filtration through a Celite pad, the filtrate was concentrated, and the residue was purified by column chromatography on SiO_2 (dichloromethane/hexane) and recrystallization from methanol to give **17** (342 mg, 55%) as orange-brown crystals. A solid that was recovered from the mother liquor was further purified through a SiO_2 column (dichloromethane/hexane) and HPLC with a standard normal-phase column (dichloromethane/hexane = 5:95; YMC-Pack SIL, SIL-06, YMC Co., Ltd., Japan) to give **17** (79 mg, 13%) as an orange-brown amorphous solid. An analytical sample was obtained as pale yellow crystals by purification through a SiO_2 column (dichloromethane/hexane) and GPC (chloroform; JAIGEL-1H & 2H), followed by recrystallization from methanol. **17**: mp 120-121 °C; elemental analyses Found: C, 77.72; H, 8.56%. Calcd for $C_{54}H_{70}Si_4$: C, 78.00; H, 8.49%; ^{1}H NMR δ_H(400 MHz; $CDCl_3$; Me_4Si)/ppm 7.57-7.52 (4H, m), 7.39 (2H, s), 7.31-7.26 (4H, m), 1.16-1.11 (42H, m), 0.27 (18H, s); ^{13}C NMR δ_C(100 MHz; $CDCl_3$)/ppm 132.8, 132.4, 130.8, 128.4, 128.2, 127.9, 126.1, 125.9, 125.6, 105.2, 103.7, 101.5, 95.3, 94.0, 91.5, 18.7, 11.3, 0.0; FD-LRMS *m/z* 830.4 (M^+, 100%), 831.4 ($[M+1]^+$, 79), 832.4 ($[M+2]^+$, 47), 833.4 ($[M+3]^+$, 20), 834.4 ($[M+4]^+$, 5), 835.4 ($[M+5]^+$, 2).

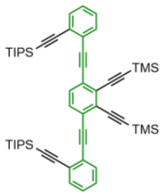

17

---

12 V. Diemer, F. R. Leroux and F. Colobert, *Eur. J. Org. Chem.*, 2011, 327.
13 M. L. Bell, R. C. Chiechi, C. A. Johnson, D. B. Kimball, A. J. Matzger, W. B. Wan, T. J. R. Weakley and M. M. Haley, *Tetrahedron*, 2001, **57**, 3507.

(c) Preparation of pr-**3** (TFA-protected) [X = (*R*)-CH(CH_3)(cHex)]

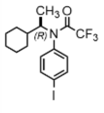

22

To a solution of **17** (362 mg, 0.435 mmol), **22**[14] (1.85 g, 4.36 mmol), $Pd(PPh_3)_4$ (110 mg, 0.0952 mmol) and CuI (17 mg, 0.089 mmol) in Et_3N (58 mL) and THF (23 mL) was added a diluted solution of TBAF (1.9 mmol) in THF (8 mL) via a syringe pump over 2 h at 50 °C under an argon atmosphere. The reaction mixture was diluted with ethyl acetate, washed with 1M HCl and brine, dried over magnesium sulfate, and concentrated. The residue was dissolved in CH_2Cl_2 (14 mL) containing Et_3N (1.2 mL), which was treated with trifluoroacetic anhydride (TFAA) (1.2 mL), diluted with dichloromethane and quenched with satd. aq. $NaHCO_3$. The organic layer was separated and concentrated. The residue was dissolved in ethyl acetate and washed with 1M aq. HCl and brine, dried over magnesium sulfate and concentrated. The residue was purified by column chromatography on SiO_2 (dichloromethane/hexane-dichloromethane) to give pr-**3** (519 mg) as an amorphous solid in 76% yield. An analytical sample was obtained as a white amorphous solid by purification through GPC (chloroform; JAIGEL-1H & 2H) and HPLC (ethyl acetate/hexane = 1:3). pr-**3**: mp 119-121 °C; $[\alpha]_D^{24}$ = –32.7 (*c* = 0.251, chloroform); elemental analyses Found: C, 72.03; H, 5.52; N, 3.52%. Calcd for $C_{94}H_{86}F_{12}N_4O_4$: C, 72.20; H, 5.54; N, 3.58%; ^{1}H NMR δ_H(400 MHz; $CDCl_3$; Me_4Si)/ppm 7.64-7.61 (4H, br.m), 7.56 (2H, s), 7.55-7.46 (8H, br.d×2), 7.43-7.35 (4H, m), 7.16-7.02 (8H, br.d×2), 4.45-4.26 (4H, m), 1.94-1.92 (4H, br.d), 1.85-1.56 (16H, m), 1.48-1.33 (4H, m), 1.28-1.00 (16H, m), 1.08 (6H, d, *J* = 6.8 Hz), 1.06 (6H, d, *J* = 7.2 Hz), 0.99-0.84 (4H, m); ^{13}C NMR δ_C(100 MHz; $CDCl_3$)/ppm 156.7 (C(=O)CF_3), 136.5, 136.0, 132.1 (br.), 132.1, 132.0, 131.4, 130.6 (br.), 129.3 (br.), 128.8, 128.5, 127.9, 126.1, 125.4, 125.4, 124.2, 124.0, 116.5 (CF_3), 97.1, 94.4, 92.6, 91.5, 89.9, 88.6, 60.1, 59.9, 40.3, 30.7, 30.6, 29.8, 29.7, 26.1, 25.9, 25.8, 16.3; FD-LRMS *m/z* 1452.6 ([M–(1-cyclohexylethyl)+1H]$^+$, 2%), 1453.6 ([M+1–(1-cyclohexylethyl)+1H]$^+$, 3), 1562.8 (M^+, 92), 1563.8 ([M+1]$^+$, 100), 1564.8 ([M+2]$^+$, 56), 1565.8 ([M+3]$^+$, 20), 1566.8 ([M+4]$^+$, 7).

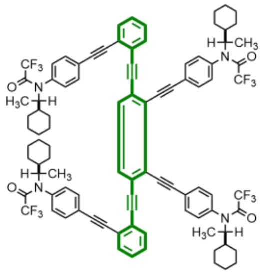

pr-**3**

(c) Preparation of pr-**3B** (TFA-protected) [X = *n*Bu]

To a solution of **17** (444 mg, 0.534 mmol), **22B** (1.19 g, 3.20 mmol), $Pd(PPh_3)_4$ (126 mg, 0.109 mmol) and CuI (41 mg, 0.22 mmol) in Et_3N (72 mL) and THF (30 mL) was added a diluted solution of TBAF (2.7 mmol) in THF (10 mL) via a syringe pump over 2 h at 50 °C under an argon atmosphere. The reaction mixture was diluted with ethyl acetate and washed with 1M aq. HCl and brine were dried over magnesium sulfate and passed through a SiO_2/Celite pad. The filtrate was concentrated, and the residue was dissolved in CH_2Cl_2 (12 mL) containing Et_3N (1.2 mL), which was treated with TFAA (1.1 mL), diluted with dichloromethane, and quenched with satd. aq. $NaHCO_3$. The organic layer was separated and concentrated. The residue was dissolved in ethyl acetate and washed with 1M aq. HCl and brine, dried over magnesium sulfate and concentrated. The residue was purified by column chromatography on SiO_2 (dichloromethane/hexane-dichloromethane) to give pr-**3B** (522 mg) as a brown amorphous solid in 73% yield. An analytical sample was obtained as a

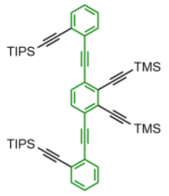

17

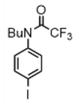

22B

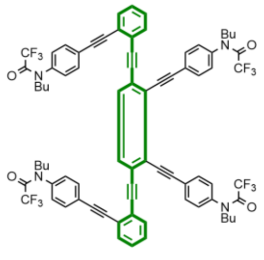

pr-**3B**

---

14 R. Katoono, K. Kusaka, K. Fujiwara and T. Suzuki, *Chem. Asian J.*, 2014, **9**, 3182.

white amorphous solid by purification through GPC (chloroform;JAIGEL-1H & 2H) and HPLC (ethyl acetate/hexane = 1:3). pr-**3B**: mp 76-78 °C; elemental analyses Found: C, 69.37; H, 4.53; N, 4.08%. Calcd for $C_{78}H_{62}F_{12}N_4O_4$: C, 69.53; H, 4.64; N, 4.16%; ^{1}H NMR δ_H(400 MHz; $CDCl_3$; Me_4Si)/ppm 7.65-7.61 (4H, m), 7.56 (2H, s), 7.53 (4H, d, *J* = 8.0 Hz), 7.52 (4H, d, *J* = 8.0 Hz), 7.43-7.35 (4H, m), 7.12 (8H, d×2, *J* = 8.0 Hz), 3.70 (4H, t, *J* = 7.2 Hz), 3.68 (4H, t, *J* = 7.6 Hz), 1.55-1.46 (8H, m), 1.37-1.24 (8H, m), 0.90 (6H, t, *J* = 7.2 Hz), 0.87 (6H, t, *J* = 7.2 Hz); ^{13}C NMR δ_C(100 MHz; $CDCl_3$)/ppm 156.4 ($\underline{C}$(=O)CF_3), 139.2, 138.8, 132.7, 132.6, 132.1, 132.0, 131.4, 128.8, 128.5, 128.3, 128.3, 127.9, 126.1, 125.4, 125.4, 124.0, 123.8, 116.3 ($\underline{C}F_3$), 97.1, 94.4, 92.6, 91.5, 89.8, 88.4, 51.6, 51.6, 28.9, 19.8, 19.8, 13.6, 13.6; FD-LRMS *m/z* 673.3 (M^{2+}, 2%), 673.8 ($[M+1]^{2+}$, 2), 1346.5 (M^+, 100), 1347.5 ($[M+1]^+$, 88), 1348.5 ($[M+2]^+$, 40), 1349.5 ($[M+3]^+$, 12), 1350.5 ($[M+4]^+$, 3).

(d) Preparation of **3** [X = (*R*)-CH(CH_3)(cHex)]

i) To an ice-cooled solution of pr-**3** (602 mg, 0.385 mmol) in THF (42 mL) were added 60% NaH in oil (1.23 g, 30.8 mmol) and MeOH (1.4 mL). The solution was stirred at room temperature for 15 min and diluted with dichloromethane, which was washed with water and brine, dried over magnesium sulfate, and concentrated. The resulting solid was purified by column chromatography on SiO_2 (dichloromethane/hexane) to give pr-**3'** (446 mg) as a yellow amorphous solid in 98% yield.

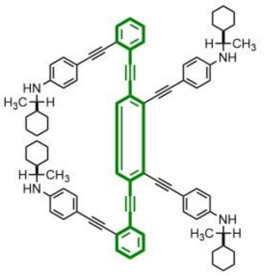

pr-**3'**

ii) To a refluxed solution of pr-**3'** (319 mg, 0.270 mmol) in toluene (108 mL) containing Et_3N (0.2 mL) were added five portions (112, 112, 119, 114, and 116 mg, a total of 2.82 mmol) of terephthaloyl chloride at an interval of 30 min. The reaction mixture was diluted with dichloromethane and quenched with 1M aq. NaOH at room temperature. The organic layer was washed with brine, dried over magnesium sulfate, and concentrated. The residue was purified by column chromatography on Al_2O_3/SiO_2 [Al_2O_3/SiO_2 = 1:2 (v/v); ethyl acetate/dichloromethane (first round) and ethyl acetate/hexane (second round)] to give **3** (304 mg) as a white solid in 78% yield. An analytical sample was obtained as a white solid by purification through GPC (chloroform; JAIGEL-1H & 2H) and recrystallization from ethanol or HPLC (ethanol/ethyl acetate/hexane = 5:600:400). **3**: mp >300 °C; $[\alpha]_D^{24}$ = –809 (*c* = 0.430, chloroform); elemental analyses Found: C, 84.42; H, 6.42; N, 3.90%. Calcd for $C_{102}H_{94}N_4O_4\cdot(C_2H_5OH)_{0.5}$: C, 84.57; H, 6.68; N, 3.83%; ^{1}H NMR δ_H(400 MHz; $CDCl_3$; Me_4Si)/ppm 7.68-7.65 (2H, m), 7.66-7.65 (2H, m), 7.55 (4H, d, *J* = 8.0 Hz), 7.53 (2H, s), 7.47-7.32 (8H, br.m), 7.03 (4H, d, *J* = 8.4 Hz), 7.00 (4H, d, *J* = 8.4 Hz), 7.11-6.48 (8H, br.), 4.62-4.43 (4H, br.m), 2.12 (4H, br.s), 1.86-1.49 (16H, br.m), 1.35-0.8 (36H, br.m); ^{13}C NMR δ_C(100 MHz; $CDCl_3$/ppm; 313 K) 170.4, 170.3, 142.1, 141.1, 138.3, 138.0, 132.8, 132.6, 132.4 (br.), 132.3, 132.1, 131.5-130.1 (br.), 130.1-129.8 (br.), 128.7, 128.4, 127.5, 127.3, 127.2, 126.2, 125.2, 125.1, 121.8, 121.7, 97.1, 93.9, 92.8, 91.2, 89.8, 88.8, 58.5, 57.8, 41.7, 41.5, 30.9, 30.4, 30.3, 26.3, 26.2, 26.1, 16.8, 16.7; FD-LRMS *m/z* 719.4 (M^{2+} , 3%), 719.9 ($[M+1]^{2+}$, 3), 720.4 ($[M+2]^{2+}$, 2), 1218.5 ([M–2(1-cyclohexylethyl)+2H]$^+$, 3), 1219.5 ([M+1–2(1-cyclohexylethyl)+2H]$^+$, 3), 1328.6 ([M–(1-cyclohexylethyl)+H]$^+$, 21), 1329.6 ([M+1–(1-cyclohexylethyl)+H]$^+$, 23), 1330.7 ([M+2–(1-cyclohexylethyl)+H]$^+$, 13), 1331.7 ([M+3–(1-cyclohexylethyl)+H]$^+$, 5), 1344.7 ([M+2–(methyl)–(cyclohexyl)+2H]$^+$, 6), 1345.6 ([M+3–(methyl)–(cyclohexyl)+2H]$^+$, 9), 1355.7 ([M–(cyclohexyl)]$^+$, 3), 1356.7 ([M+1–(cyclohexyl)]$^+$, 3), 1357.7 ([M+2–(cyclohexyl)]$^+$, 3), 1410.8 ([M–2(methyl)+2H]$^+$, 7), 1411.8 ([M+1–

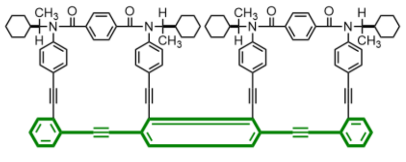
3

2(methyl)+2H]$^+$, 8), 1412.8 ([M+2–2(methyl)+2H]$^+$, 4), 1413.8 ([M+3–2(methyl)+2H]$^+$, 2), 1438.8 (M$^+$, 87), 1439.8 ([M+1]$^+$, 100), 1440.8 ([M+2]$^+$, 59), 1441.8 ([M+3]$^+$, 25), 1442.8 ([M+4]$^+$, 8); FD-HRMS Found: 1438.72606, Calcd for $C_{102}H_{94}N_4O_4$: 1438.72750; UV λ_{max}(CH_2Cl_2)/nm (log ε) 354 (shoulder 4.66), 330 (sh. 4.90), 307 (5.16); CD λ(CH_2Cl_2)/nm ($\Delta\varepsilon$ at 293 K) 347 (–77), 331 (–86), 308 (+92), 281 (–27).

(d) Preparation of **3B** [X = *n*Bu]

i) To an ice-cooled solution of pr-**3B** (237 mg, 0.176 mmol) in THF (19 mL) were added 60% NaH in oil (37 mg, 0.93 mmol) and MeOH (0.65 mL). The solution was stirred at room temperature for 9 min and diluted with dichloromethane, which was washed with water and brine, dried over magnesium sulfate, and concentrated. The resulting solid was purified by column chromatography on Al_2O_3 (hexane-dichloromethane) to give pr-**3B'** (162 mg) as a yellow solid in 96% yield.

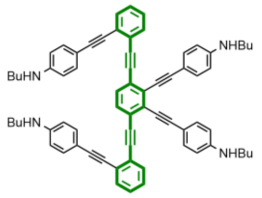

pr-**3B'**

ii) To a refluxed solution of pr-**3B'** (162 mg, 0.168 mmol) in toluene (67 mL) and THF (20 mL) containing Et_3N (0.2 mL) were added two portions (76 and 17 mg, a total of 0.46 mmol) of terephthaloyl chloride at an interval of 50 min. The reaction mixture was diluted with dichloromethane and quenched with 1M aq. NaOH at room temperature. The organic layer was dried over magnesium sulfate and passed through an Al_2O_3 pad. The filtrate was concentrated and the residue was purified by column chromatography on Al_2O_3/SiO_2 [Al_2O_3/SiO_2 = 1:2 (v/v); ethyl acetate/dichloromethane (first round)] and SiO_2 [ethyl acetate/dichloromethane (second and third rounds)], followed by HPLC (ethyl acetate/dichloromethane = 50:50) to give **3B** (134 mg) as a pale yellowish-white solid in 65% yield. An analytical sample was obtained as a white solid by purification through GPC (chloroform; JAIGEL-1H & 2H) and recrystallization from 1-propanol. **3B**: mp >300 °C; elemental analyses Found: C, 84.23; H, 5.69; N, 4.59%. Calcd for $C_{86}H_{70}N_4O_4$: C, 84.42; H, 5.77; N, 4.58%; ^{1}H NMR δ_H(400 MHz; $CDCl_3$; Me_4Si)/ppm 7.66-7.60 (4H, m), 7.56 (4H, d, J = 8.8 Hz), 7.54 (2H, s), 7.42 (4H, d, J = 8.4 Hz), 7.43-7.33 (4H, m), 7.06 (4H, d, J = 8.4 Hz), 7.03 (4H, d, J = 8.4 Hz), 6.88 (4H, br.d), 6.85 (4H, br.d), 4.00-3.73 (8H, br.m), 1.57-1.50 (8H, m), 1.39-1.28 (8H, m), 0.88 (6H, t, J = 7.2 Hz), 0.87 (6H, t, J = 7.2 Hz); ^{13}C NMR δ_C(100 MHz; $CDCl_3$/ppm) 169.9, 169.8, 143.5, 142.8, 137.7, 137.2, 132.8, 132.7, 132.6, 132.6, 132.1, 128.6, 128.6, 128.4, 128.3, 127.4, 127.3, 126.1, 125.0, 124.9, 121.6, 121.4, 96.8, 93.8, 92.6, 91.1, 89.6, 88.4, 49.4, 49.1, 29.8, 20.1, 20.1, 13.7, 13.7; FD-LRMS *m*/*z* 611.3 (M^{2+}, 6%), 611.8 ([M+1]$^{2+}$, 6), 612.3 ([M+2]$^{2+}$, 3), 1222.6 (M$^+$, 100), 1223.6 ([M+1]$^+$, 96), 1224.6 ([M+2]$^+$, 49), 1225.6 ([M+3]$^+$, 17), 1226.6 ([M+4]$^+$, 5); UV λ_{max}(CH_2Cl_2)/nm (log ε) 354 (shoulder 4.65), 330 (sh. 4.90), 307 (5.15).

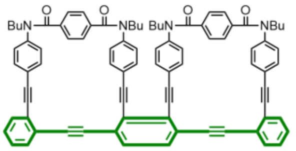

3B

(e) Preparation of **19** [X = (*R*)-CH(CH_3)(cHex)]

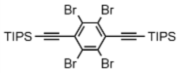
18

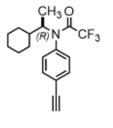
23

To a solution of **18**[15] (1.10 g, 1.46 mmol) and **23**[14] (9.42 g, 29.1 mmol) in Et_3N (62 mL) were added Pd(PPh_3)$_4$ (86 mg, 0.074 mmol) and CuI (64 mg, 0.34 mmol) at 80 °C under an argon atmosphere, and the mixture was stirred at that temperature for 29 h. After removing a solid by filtration through a Celite pad, the filtrate was concentrated, and the residue was purified by column chromatography on SiO_2 (dichloromethane/hexane-dichloromethane), followed by suspending in methanol to give **19** (2.03 g) as an off-white solid in 80% yield. An analytical sample was obtained as a white solid by further purification through a SiO_2 column (dichloromethane/hexane-dichloromethane) and GPC (chloroform; JAIGEL-1H & 2H), followed by refluxing in ethanol. **19**: mp 225-228 °C; $[\alpha]_D^{24} = -40.9$ (c = 0.422, chloroform); elemental analyses Found: C, 69.47; H, 6.87; N, 3.29%. Calcd for $C_{100}H_{118}F_{12}N_4O_4Si_2$: C, 69.66; H, 6.90; N, 3.25%; ^{1}H NMR δ_H(400 MHz; $CDCl_3$; Me_4Si)/ppm 7.56 (8H, d, J = 7.6 Hz), 7.13 (8H, d, J = 7.6 Hz), 4.41 (4H, dq, J = 6.8, 10 Hz), 1.95 (4H, d, J = 10 Hz), 1.85-1.59 (16H, m), 1.47-1.36 (4H, m), 1.27-0.87 (62H, m), 1.11 (12H, d, J = 6.8 Hz); ^{13}C NMR δ_C(100 MHz; $CDCl_3$)/ppm 156.8 (<u>C</u>(=O)CF_3), 136.6, 132.2, 132.1, 130.7, 129.4, 128.3, 127.5, 123.9, 116.5 (<u>C</u>F_3), 103.5, 102.7, 98.0, 88.6, 59.9, 40.4, 30.7, 29.8, 26.1, 26.0, 25.8, 18.7, 16.4, 11.3; FD-LRMS *m/z* 861.5 (M^{2+}, 4%), 862.0 ($[M+1]^{2+}$, 6), 862.5 ($[M+2]^{2+}$, 4), 863.0 ($[M+3]^{2+}$, 2), 1723.0 (M^+, 81), 1724.0 ($[M+1]^+$, 100), 1725.0 ($[M+2]^+$, 68), 1726.0 ($[M+3]^+$, 34), 1727.0 ($[M+4]^+$, 13).

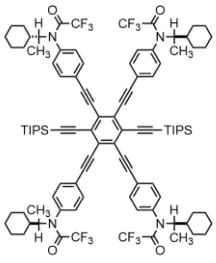
19

(e) Preparation of **19B** [X = *n*Bu]

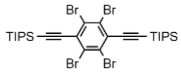
18

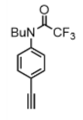
23B

To a solution of **18** (1.22 g, 1.61 mmol) and **23B**[14] (8.67 g, 32.2 mmol) in Et_3N (64 mL) were added Pd(PPh_3)$_4$ (93 mg, 0.081 mmol) and CuI (57 mg, 0.30 mmol) at 80 °C under an argon atmosphere, and the mixture was stirred at that temperature for 23 h. After removing a solid by filtration through a Celite pad, the filtrate was concentrated, and the residue was purified by column chromatography on SiO_2 (dichloromethane/hexane-dichloromethane), followed by suspending in methanol to give **19B** (1.71 g) as a yellow solid in 70% yield. An analytical sample was obtained as pale yellow crystals by purification through a SiO_2 column (dichloromethane/hexane-dichloromethane) and GPC (chloroform; JAIGEL-1H & 2H), followed by recrystallization from ethyl acetate. **19B**: mp 281-284 °C (decomp.); elemental analyses Found: C, 66.89; H, 6.23; N, 3.71%. Calcd for $C_{84}H_{94}F_{12}N_4O_4Si_2$: C, 66.91; H, 6.28; N, 3.72%; ^{1}H NMR δ_H(400 MHz; $CDCl_3$; Me_4Si)/ppm 7.58 (8H, d, J = 8.4 Hz), 7.17 (8H, d, J = 8.4 Hz), 3.74 (8H, t, J = 7.6 Hz), 1.58-1.51 (8H, m), 1.39-1.30 (8H, m), 1.14-1.02 (42H, m), 0.91 (12H, t, J = 7.6 Hz); ^{13}C NMR δ_C(100 MHz; $CDCl_3$)/ppm 156.4 (<u>C</u>(=O)CF_3), 139.4, 132.7, 128.4, 128.3, 127.5, 123.8, 116.4 (<u>C</u>F_3), 103.4, 102.6, 97.9, 88.4, 51.6, 28.9, 19.8, 18.6, 13.6, 11.2; FD-LRMS *m/z* 753.4 (M^{2+}, 15%), 753.9 ($[M+1]^{2+}$, 16), 754.4 ($[M+2]^{2+}$, 10), 754.9 ($[M+3]^{2+}$, 4), 1506.8 (M^+, 96), 1507.8 ($[M+1]^+$, 100), 1508.8 ($[M+2]^+$, 60), 1509.8 ($[M+3]^+$, 26).

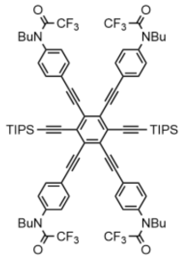
19B

---

14 R. Katoono, K. Kusaka, K. Fujiwara and T. Suzuki, *Chem. Asian J.*, 2014, **9**, 3182.
15 B. VanVeller, K. Miki and T. M. Swager, *Org. Lett.*, 2010, **12**, 1292.

(f) Preparation of **20** [X = (*R*)-CH(CH_3)(cHex)]

i) To a solution of **19** (2.77 g, 1.61 mmol) in THF (80 mL) was added 1M TBAF in THF (4.8 mL, 4.8 mmol) at room temperature, and the mixture was stirred at that temperature for 20 min and diluted with ethyl acetate. The diluted solution was washed with 1M aq. HCl and brine were dried over magnesium sulfate and passed through a SiO_2/Celite pad. The filtrate was concentrated, and the residue was dissolved in CH_2Cl_2 (80 mL) containing Et_3N (9.2 mL), which was treated with TFAA (8.0 mL) in an ice bath, followed by satd. aq. $NaHCO_3$. The organic layer was separated, dried over magnesium sulfate, and passed through a SiO_2 column (dichloromethane/hexane-dichloromethane) to give an oil containing **19'**. The oil was dissolved in ethyl acetate and repeatedly washed with 1M aq. HCl, dried over magnesium sulfate and concentrated. The residue was purified by column chromatography on SiO_2 (dichloromethane/hexane-dichloromethane) to yield **19'** (1.56 g) as a yellow solid in a 69% yield.

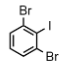

24

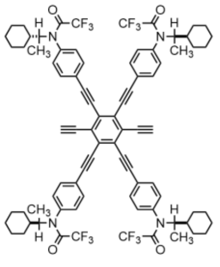

19'

ii) To a solution of **19'** (820 mg, 0.581 mmol) and **24** (2.53 g, 6.99 mmol) in Et_3N (12 mL) and benzene (18 mL) were added $Pd(PPh_3)_4$ (273 mg, 0.236 mmol) and CuI (61 mg, 0.32 mmol) at 90 °C under an argon atmosphere, and the mixture was stirred at that temperature for 9.5 h. After removing a solid by filtration through a Celite pad, the filtrate was concentrated, and the residue was purified by column chromatography on SiO_2 (dichloromethane/hexane) to give **20** (613 mg) as a yellow solid in 56% yield. An analytical sample was obtained as a pale yellow solid by purification through GPC (chloroform; JAIGEL-1H & 2H), HPLC (ethyl acetate/hexane = 1:3) and GPC (chloroform; JAIGEL-2H & 2.5H). **20**: mp 226-228 °C; $[\alpha]_D^{24}$ = –26.4 (*c* = 0.391, chloroform); elemental analyses Found: C, 60.03; H, 4.35; N, 2.92%. Calcd for $C_{94}H_{82}Br_4F_{12}N_4O_4$: C, 60.08; H, 4.40; N, 2.98%; ^{1}H NMR δ_H(400 MHz; $CDCl_3$; Me_4Si)/ppm 7.63 (12H, d, J = 8.0 Hz), 7.14 (8H, d, J = 8.0 Hz), 7.11 (2H, t, J = 8.0 Hz), 4.40 (4H, dq, J = 6.8, 10 Hz), 1.95 (4H, d, J = 10 Hz), 1.86-1.60 (16H, m), 1.50-1.37 (4H, m), 1.27-1.04 (16H), 1.12 (12H, d, J = 6.8 Hz) , 1.04-0.87 (4H, m); ^{13}C NMR δ_C(100 MHz; $CDCl_3$)/ppm 156.7 ($\underline{C}$(=O)CF_3), 136.6, 132.6, 132.4, 131.5, 130.7, 130.5, 129.4, 128.4, 127.0, 126.7, 123.9, 116.4 ($\underline{C}F_3$), 98.8, 96.4, 94.4, 88.6, 60.0, 40.3, 30.7, 29.7, 26.1, 25.9, 25.7, 16.3; FD-LRMS *m/z* 938.1 ($[M+2]^{2+}$, 4%), 938.6 ($[M+3]^{2+}$, 4), 939.1 ($[M+4]^{2+}$, 8), 939.6 ($[M+5]^{2+}$, 7), 940.1($[M+6]^{2+}$, 7), 940.6 ($[M+7]^{2+}$, 5), 941.1 ($[M+8]^{2+}$, 3), 941.6 ($[M+9]^{2+}$, 2), 1874.2 (M^+, 13), 1875.2 ($[M+1]^+$, 14), 1876.2 ($[M+2]^+$, 56), 1877.2 ($[M+3]^+$, 54), 1878.2 ($[M+4]^+$, 100), 1879.2 ($[M+5]^+$, 87), 1880.2 ($[M+6]^+$, 90), 1881.2 ($[M+7]^+$, 65), 1882.2 ($[M+8]^+$, 43), 1883.2 ($[M+9]^+$, 25), 1884.2 ($[M+10]^+$, 10), 1885.2 ($[M+11]^+$, 4).

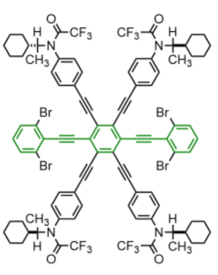

20

(f) Preparation of **20B** [X = *n*Bu]

i) To a solution of **19B** (500 mg, 0.332 mmol) in THF (17 mL) was added 1M TBAF in THF (0.7 mL, 0.7 mmol) at room temperature, and the mixture was stirred at that temperature for 20 min and diluted with ethyl acetate. The diluted solution was washed with water and brine, dried over magnesium sulfate, and passed through a SiO_2/Celite pad. The filtrate was concentrated, and the residue was dissolved in CH_2Cl_2 (16 mL) containing Et_3N (0.4 mL), which was treated with TFAA (1.2 mL) in an ice bath, followed by satd. aq. $NaHCO_3$. The organic layer was separated,

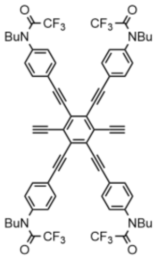

19B'

dried over magnesium sulfate, and concentrated. The residue was purified by column chromatography on SiO_2 (dichloromethane/hexane-dichloromethane) to give **19B'** (331 mg) as a yellow amorphous solid in 84% yield.

ii) To a solution of **19B'** (331 mg, 0.277 mmol) and **24** (1.21 g, 3.33 mmol) in Et_3N (6 mL) and benzene (8 mL) were added $Pd(PPh_3)_4$ (131 mg, 0.113 mmol) and CuI (27 mg, 0.14 mmol) at 90 °C under an argon atmosphere, and the mixture was stirred at that temperature for 16 h. After removing a solid by filtration through a Celite pad, the filtrate was concentrated and the residue was passed through a SiO_2 column (dichloromethane/hexane) to create a mixture containing **20B**. The mixture was dissolved in CH_2Cl_2 (7 mL) containing Et_3N (0.2 mL), which was treated with TFAA (0.1 mL) in an ice bath, followed by satd. aq. $NaHCO_3$. The organic layer was separated, dried over magnesium sulfate, and concentrated. The residue was purified by column chromatography on SiO_2 (dichloromethane) to give **20B** (234 mg) as a yellow solid in 51% yield. An analytical sample was obtained as a yellow solid by further purification through GPC (chloroform; JAIGEL-1H & 2H) and recrystallization from ethyl acetate. **20B**: mp 231-233 °C; elemental analyses Found: C, 55.84; H, 3.31; N, 3.32%. Calcd for $C_{78}H_{58}Br_4F_{12}N_4O_4 \cdot H_2O$: C, 55.73; H, 3.60; N, 3.33%; ^{1}H NMR δ_H(400 MHz; $CDCl_3$; Me_4Si)/ppm 7.65 (8H, d, J = 8.0 Hz), 7.63 (4H, d, J = 8.0 Hz), 7.18 (8H, d, J = 8.0 Hz), 7.11 (2H, t, J = 8.0 Hz), 3.74 (8H, t, J = 7.6 Hz), 1.59-1.51 (8H, m), 1.39-1.29 (8H, m), 0.92 (12H, t, J = 7.2 Hz); ^{13}C NMR δ_C(100 MHz; $CDCl_3$)/ppm 156.4 (C(=O)CF_3), 139.6, 133.1, 131.6, 130.5, 128.4, 128.3, 127.1, 126.8, 126.7, 123.8, 116.4 (CF_3), 98.8, 96.5, 94.4, 88.5, 51.6, 29.0, 19.8, 13.6; FD-LRMS m/z 829.1 (M^{2+}, 1%), 830.1 ($[M+2]^{2+}$, 5), 830.6 ($[M+3]^{2+}$, 4), 831.1 ($[M+4]^{2+}$, 8), 831.6 ($[M+5]^{2+}$, 6), 832.1 ($[M+6]^{2+}$, 7), 832.6 ($[M+7]^{2+}$, 5), 833.1 ($[M+8]^{2+}$, 3), 1658.1 (M^+, 14), 1659.1 ($[M+1]^+$, 12), 1660.1 ($[M+2]^+$, 58), 1661.1 ($[M+3]^+$, 51), 1662.1 ($[M+4]^+$, 100), 1663.1 ($[M+5]^+$, 75), 1664.1 ($[M+6]^+$, 82), 1665.1 ($[M+7]^+$, 54), 1666.1 ($[M+8]^+$, 36), 1667.1 ($[M+9]^+$, 18), 1668.1 ($[M+10]^+$, 7), 1669.1 ($[M+11]^+$, 3).

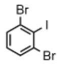

24

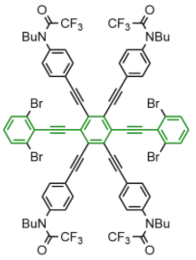

20B

(g) Preparation of pr-**5** (TFA-protected) [X = (R)-CH(CH_3)(cHex)]

To a solution of **20** (428 mg, 0.228 mmol) and **23** (2.21 g, 6.84 mmol) in Et_3N (13 mL) and THF (5 mL) were added $Pd(PPh_3)_4$ (17 mg, 0.015 mmol) and CuI (7.4 mg, 0.039 mmol) at 80 °C under an argon atmosphere, and the mixture was stirred at that temperature for 65 h with maintenance of the volume of solvents. After removing a solid by filtration through a Celite pad, the filtrate was concentrated, and the residue was purified by column chromatography on SiO_2 (dichloromethane-ethyl acetate/dichloromethane) to give pr-**5** (165 mg) as a brown amorphous solid in 25% yield. An analytical sample was obtained as a yellow solid by further purification through a SiO_2 column (dichloromethane-ethyl acetate/dichloromethane), GPC (chloroform; JAIGEL-1H & 3H), and HPLC (ethyl acetate/dichloromethane = 4:96). pr-**5**: mp 125-139 °C (decomp.); $[\alpha]_D^{24} = -72.3$ (c = 0.176, chloroform); elemental analyses Found: C, 69.78; H, 5.64; N, 3.92%. Calcd for $C_{166}H_{158}F_{24}N_8O_8$: C, 69.98; H, 5.59; N, 3.93%; ^{1}H NMR δ_H(400 MHz; $CDCl_3$; Me_4Si)/ppm 7.67 (4H, d, J = 7.6 Hz), 7.48 (2H, t, J = 7.6 Hz), 7.43 (8H, d, J = 8.0 Hz), 7.41 (8H, d, J = 8.0 Hz), 7.01 (16H, d×2, J = 8.0 Hz), 4.36-4.24 (8H, dq×2), 1.92-0.80 (88H, m), 1.05 (12H, d, J = 7.2

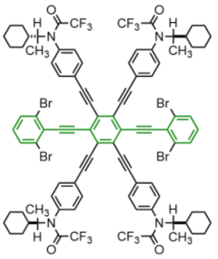

20

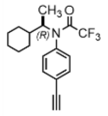

23

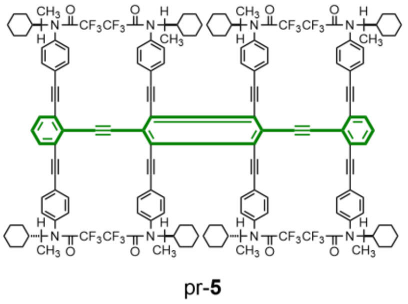

pr-**5**

Hz), 0.97 (12H, d, J = 6.8 Hz); ^{13}C NMR δ_C(100 MHz; $CDCl_3$)/ppm 156.6 (C(=O)CF_3), 156.5 (C(=O)CF_3), 136.8, 136.1, 132.3, 132.2, 132.0, 130.5, 129.1 (br.m), 129.0, 128.3, 127.5, 127.4, 126.2, 123.6, 123.4, 116.4 (CF_3), 116.4 (CF_3), 99.2, 97.2, 94.1, 93.3, 89.3, 88.2, 60.2, 59.8, 40.3, 40.2, 30.6, 30.6, 29.8, 29.7, 26.1, 26.0, 25.9, 25.9, 25.7, 25.7, 16.2, 16.2; FD-LRMS m/z 2847.2 (M^+, 53%), 2848.2 ($[M+1]^+$, 100), 2849.2 ($[M+2]^+$, 94), 2850.2 ($[M+3]^+$, 63), 2851.2 ($[M+4]^+$, 30), 2852.2 ($[M+5]^+$, 13), 2853.2 ($[M+6]^+$, 5).

(g) Preparation of pr-**5B** (TFA-protected) [X = nBu]

To a solution of **20B** (335 mg, 0.201 mmol) and **23B** (1.63 g, 6.05 mmol) in Et_3N (12 mL) and THF (5 mL) were added $Pd(PPh_3)_4$ (16 mg, 0.014 mmol) and CuI (6.3 mg, 0.033 mmol) at 80 °C under an argon atmosphere, and the mixture was stirred at that temperature for 65 h with maintenance of the volume of solvents. The filtrate was concentrated after removing a solid by filtration through a Celite pad. The residue was purified by column chromatography on SiO_2 [dichloromethane-ethyl acetate/dichloromethane (first round) and ethyl acetate/hexane (second round)] to give pr-**5B** (209 mg) as a brownish-yellow amorphous solid in 43% yield. An analytical sample was obtained as a pale yellow solid by purification through a SiO_2 column (ethyl acetate/dichloromethane) and HPLC (ethyl acetate/hexane = 2:3). pr-**5B**: mp 109-121 °C (decomp.); elemental analyses Found: C, 66.07; H, 4.48; N, 4.58%. Calcd for $C_{134}H_{110}F_{24}N_8O_8\cdot H_2O$: C, 66.11; H, 4.64; N, 4.60%; ^{1}H NMR δ_H(400 MHz; $CDCl_3$; Me_4Si)/ppm 7.67 (4H, d, J = 7.6 Hz), 7.48 (2H, t, J = 7.6 Hz), 7.43 (8H, d, J = 8.0 Hz), 7.42 (8H, d, J = 8.0 Hz), 7.04 (8H, d, J = 8.0 Hz), 7.03 (8H, d, J = 8.0 Hz), 3.67 (8H, t, J = 7.6 Hz), 3.60 (8H, t, J = 7.6 Hz), 1.52-1.37 (16H, m), 1.35-1.17 (16H, m), 0.90 (12H, t, J = 7.2 Hz), 0.79 (12H, t, J = 7.2 Hz); ^{13}C NMR δ_C(100 MHz; $CDCl_3$)/ppm 156.3 (C(=O)CF_3), 156.2 (C(=O)CF_3), 139.5, 138.9, 132.8, 132.0, 128.9, 128.2, 128.1, 127.4, 127.3, 126.9 (br.), 126.1, 123.5, 123.2, 116.3 (CF_3), 116.2 (CF_3), 99.1, 97.2, 94.1, 93.3, 89.1, 88.0, 51.6, 51.5, 28.8, 28.8, 19.7, 19.6, 13.6, 13.5; FD-LRMS m/z 1207.4 (M^{2+}, 2%), 1207.9 ($[M+1]^{2+}$, 6), 1208.4 ($[M+2]^{2+}$, 4), 2414.9 (M^+, 65), 2415.9 ($[M+1]^+$, 100), 2416.9 ($[M+2]^+$, 77), 2417.9 ($[M+3]^+$, 43), 2418.9 ($[M+4]^+$, 20), 2419.9 ($[M+5]^+$, 7); FD-HRMS Found: 2414.80547, Calcd for $C_{134}H_{110}F_{24}N_8O_8$: 2414.80634.

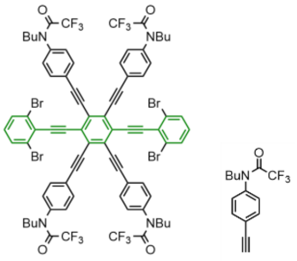

20B **23B**

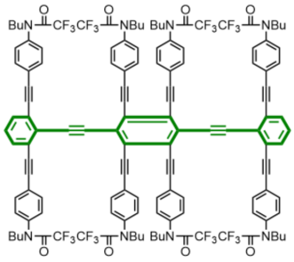

pr-**5B**

(h) Preparation of **5** [X = (R)-CH(CH_3)(cHex)]

i) To an ice-cooled solution of pr-**5** (125 mg, 0.0439 mmol) in THF (69 mL) were added 60% NaH in oil (575 mg, 14.4 mmol) and MeOH (2.3 mL). The solution was stirred at room temperature for 15 min and diluted with dichloromethane, which was washed with satd. aq. $NaHCO_3$, dried over magnesium sulfate and concentrated. The resulting solid was purified by column chromatography on SiO_2 (dichloromethane/hexane) to give pr-**5'** (70 mg) as an orange solid with a 77% yield.

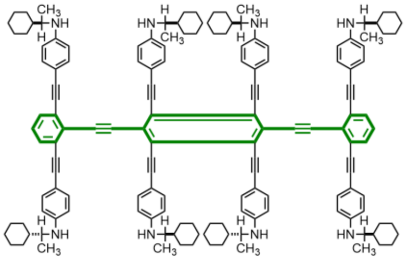

pr-**5'**

ii) To a refluxed solution of pr-**5'** (46 mg, 0.022 mmol) in toluene (9 mL) containing Et_3N (0.8 mL) were added five portions (20, 21, 20, 20, and 20 mg, a total of 0.50 mmol) of terephthaloyl chloride at an interval of 30 min. The reaction mixture was diluted with dichloromethane and quenched with 1M aq. NaOH at room temperature. The organic layer was dried over magnesium sulfate and concentrated. The residue was passed through an Al_2O_3/SiO_2 column [Al_2O_3/SiO_2 = 1:2 (v/v); ethyl acetate/dichloromethane] to give **5** (31 mg) as a yellow solid in 54% yield. An analytical sample was obtained as a yellow solid by purification through HPLC [methanol/dichloromethane = 4:96 (first round) and 6:94 (second round)] and GPC (chloroform; JAIGEL-1H & 3H), followed by recrystallization from 1-propanol. **5**: mp >300 °C; $[\alpha]_D^{24}$ = –1338 (c = 0.344, chloroform); elemental analyses Found: C, 82.64; H, 6.63; N, 4.26%. Calcd for $C_{182}H_{174}N_8O_8{\cdot}(H_2O)_2$: C, 82.88; H, 6.80; N, 4.25%; ^{1}H NMR δ_H(400 MHz; $CDCl_3$; Me_4Si; 323 K)/ppm major conformer 7.66 (4H, d, J = 8.0 Hz), 7.51-7.42 (8H, br.d), 7.40 (2H, t, J = 8.0 Hz), 7.38 (8H, d, J = 8.8 Hz), 7.12 (8H, br.s), 6.99 (16H, s), 6.43 (8H, br.s), 4.64-4.54 (8H, dq×2), 2.14 (4H, d, J = 10.8 Hz), 2.11 (4H, d, J = 10.8 Hz), 1.82-0.85 (80H, m), 1.06 (12H, d, J = 6.8 Hz), 0.94 (12H, d, J = 7.2 Hz); ^{13}C NMR δ_C(100 MHz; $CDCl_3$/ppm; 313 K) major conformer 170.5, 170.2, 142.3, 140.9, 138.4, 138.2, 132.5, 132.4, 131.6, 130.0, 128.9, 128.6, 128.2, 127.2, 127.1, 126.6, 126.4, 121.7, 121.1, 99.2, 96.6, 93.4, 93.1, 89.4, 88.8, 57.5, 56.8, 42.6, 42.1, 30.6, 30.6, 30.5, 30.2, 26.2, 26.1, 26.0, 26.0, 16.6, 16.5; FD-LRMS m/z 104.1 ($C_7H_4O^{\cdot+}$, 28%), 105.1 ($C_7H_5O^+$, 21), 1299.7 (M^{2+} , 5), 1300.3 ($[M+1]^{2+}$, 11), 1300.8 ($[M+2]^{2+}$, 12), 1301.3 ($[M+3]^{2+}$, 10), 1301.8 ($[M+4]^{2+}$, 7), 1302.2 ($[M+5]^{2+}$, 5), 2489.3 ($[M–(1\text{-cyclohexylethyl})+H]^+$, 14), 2490.3 ($[M+1–(1\text{-cyclohexylethyl})+H]^+$, 28), 2491.3 ($[M+2–(1\text{-cyclohexylethyl})+H]^+$, 31), 2492.3 ($[M+3–(1\text{-cyclohexylethyl})+H]^+$, 22), 2493.3 ($[M+4–(1\text{-cyclohexylethyl})+H]^+$, 17), 2516.5 ($[M–(\text{cyclohexyl})]^+$, 6), 2517.3 ($[M+1–(\text{cyclohexyl})]^+$, 13), 2518.3 ($[M+2–(\text{cyclohexyl})]^+$, 14), 2519.3 ($[M+3–(\text{cyclohexyl})]^+$, 10), 2520.3 ($[M+4–(\text{cyclohexyl})]^+$, 11), 2599.4 ($M^+$, 33), 2600.4 ($[M+1]^+$, 78), 2601.4 ($[M+2]^+$, 100), 2602.4 ($[M+3]^+$, 83), 2603.4 ($[M+4]^+$, 53), 2604.4 ($[M+5]^+$, 25), 2605.4 ($[M+6]^+$, 12); FD-HRMS Found: 2599.34691, Calcd for $C_{182}H_{174}N_8O_8$: 2599.34546; UV $\lambda_{max}(CH_2Cl_2)$/nm (log ε) 390 (4.98), 368 (5.10), 327 (5.19), 306 (5.21); CD $\lambda(CH_2Cl_2)$/nm ($\Delta\varepsilon$ at 293 K) 397 (–174), 389 (–149), 372 (–185), 324 (+451), 296 (–247), 280 (–175), 271 (–226).

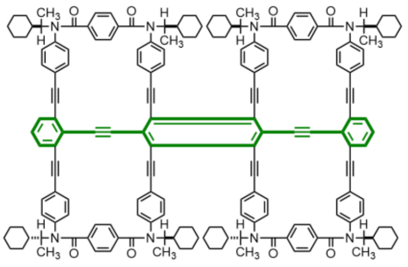

5

(h) Preparation of **5B** [X = nBu]

i) To an ice-cooled solution of pr-**5B** (126 mg, 0.0522 mmol) in THF (518 mL) were added 60% NaH in oil (28 mg, 0.70 mmol) and MeOH (0.2 mL). The solution was stirred at room temperature for 9 min and diluted with dichloromethane, which was washed with satd. aq. $NaHCO_3$, dried over magnesium sulfate and concentrated. The resulting solid was purified by column chromatography on SiO_2 (dichloromethane) to give pr-**5B'** (68 mg) as an orange amorphous solid with a 79% yield.

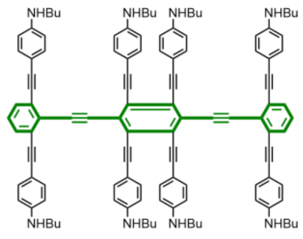

pr-**5B'**

ii) To a refluxed solution of pr-**5B'** (68 mg, 0.041 mmol) in toluene (17 mL) containing Et_3N (0.8 mL) was added terephthaloyl chloride (39 mg, 0.19 mmol), and the mixture was stirred at that temperature for 21 min. The reaction mixture was diluted with dichloromethane and quenched with 1M aq. NaOH at room temperature. The organic layer was dried over magnesium sulfate and concentrated. The residue was passed through an Al_2O_3/SiO_2 column [Al_2O_3/SiO_2 = 1:2 (v/v); ethyl acetate/dichloromethane] to give **5B** (47 mg) as a yellow solid in 53% yield. An analytical sample was obtained as a yellow solid by further purification through HPLC (methanol/dichloromethane = 5:95) and GPC (chloroform; JAIGEL-1H & 3H), followed by refluxing in 1-butanol. **5B**: mp >300 °C; elemental analyses Found: C, 81.15; H, 5.70; N, 5.01%. Calcd for $C_{150}H_{126}N_8O_8\cdot(H_2O)_3$: C, 81.05; H, 5.99; N, 5.04%; ^{1}H NMR δ_H(400 MHz; $CDCl_3$; Me_4Si; 303 K)/ppm 7.66 (4H, d, J = 8.0 Hz), 7.45 (8H, d, J = 8.8 Hz), 7.42 (8H, d, J = 8.8 Hz), 7.41 (2H, t, J = 8.0 Hz), 7.03 (8H, d, J = 8.4 Hz), 7.00 (8H, d, J = 8.4 Hz), 6.84 (16H, br.d), 4.20-4.13 (4H, m), 4.02-3.95 (4H, m), 3.64-3.55 (8H, m), 1.64-1.44 (16H, m), 1.39-1.23 (16H, m), 0.89 (12H, t, J = 7.2 Hz), 0.81 (12H, t, J = 7.2 Hz); ^{13}C NMR δ_C(100 MHz; $CDCl_3$/ppm) 169.9, 169.6, 143.8, 143.0, 137.6, 137.2, 132.6, 132.6, 128.6, 128.5 (br.), 128.0, 127.4, 127.2, 127.2, 126.2, 121.4, 121.0, 98.9, 96.4, 93.4, 93.1, 89.2, 88.4, 49.3, 49.1, 29.8, 29.7, 20.1, 20.0, 13.8, 13.7; FD-LRMS m/z 1083.5 (M^{2+}, 7%), 1084.0 ($[M+1]^{2+}$, 15), 1084.5 ($[M+2]^{2+}$, 16), 1085.0 ($[M+3]^{2+}$, 14), 1085.5 ($[M+4]^{2+}$, 9), 1086.0 ($[M+5]^{2+}$, 5), 1086.5 ($[M+6]^{2+}$, 2), 2167.0 (M^+, 57), 2168.0 ($[M+1]^+$, 100), 2169.0 ($[M+2]^+$, 100), 2170.0 ($[M+3]^+$, 68), 2171.0 ($[M+4]^+$, 31), 2172.1 ($[M+5]^+$, 13), 2173.0 ($[M+6]^+$, 4); FD-HRMS Found: 2166.97183, Calcd for $C_{150}H_{126}N_8O_8$: 2166.96986; UV λ_{max}(CH_2Cl_2)/nm (log ε) 390 (4.98), 368 (5.10), 327 (5.19), 306 (5.20).

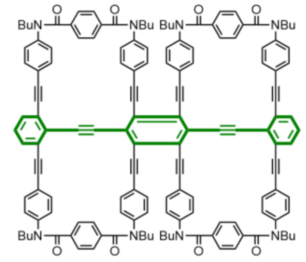

5B

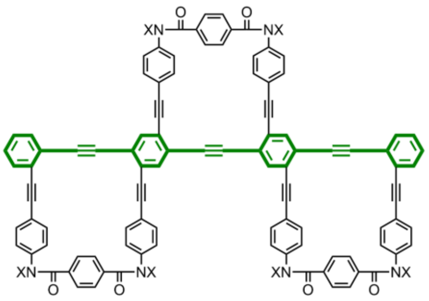

6

X = (*R*)-CH(CH_3)(cHex)

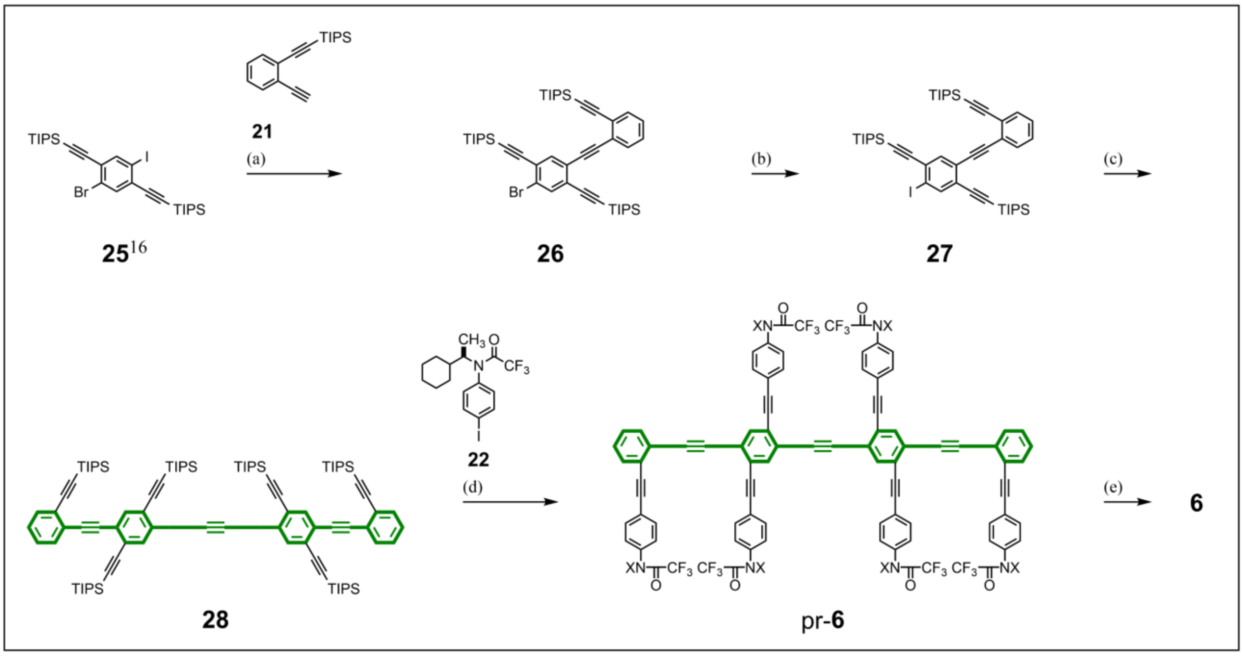

Scheme S2. Synthesis of tris(macrocycle) **6**. Reagents and yields: (a) **21**, Pd(PPh_3)$_4$, CuI, *i*Pr_2NH, THF (**26**: 81%); (b) BuLi, I_2, diethyl ether (**27**: 93%); (c) TMSA, PdCl_2(PPh_3)$_2$, CuI, Et_3N, 1,8-diazabicyclo[5.4.0]-7-undecene (DBU), H_2O, benzene (**28**: 81%); (d) **22**, Pd(PPh_3)$_4$, CuI, TBAF, Et_3N, THF (pr-**6**: 91%); (e) i) NaH, MeOH, THF, ii) terephthaloyl chloride, Et_3N, toluene (**6**: 62%).

(a) Preparation of **26**

To a solution of **25**[16] (5.88 g, 9.14 mmol) and **21** (2.15 g, 7.60 mmol) in *i*Pr_2NH (38 mL) and THF (38 mL) were added Pd(PPh_3)$_4$ (263 mg, 0.228 mmol) and CuI (89 mg, 0.47 mmol) at room temperature under an argon atmosphere, and the mixture was stirred at 47 °C for 12 h. After removing a solid by filtration through a Celite pad, the filtrate was concentrated, and the residue was purified by column chromatography on SiO_2 (hexane-dichloromethane/hexane) to give **26** (4.92 g) as a pale yellow oil in 81% yield. An analytical sample was obtained as a white solid by purification through GPC (chloroform; JAIGEL-1H & 2H). **26**: mp 58-59 °C; ^{1}H NMR δ_H(400 MHz; $CDCl_3$; Me_4Si)/ppm 7.70 (1H, s), 7.62 (1H, s), 7.52-7.48 (2H, m), 7.30-7.23 (2H, m), 1.17-1.12 (21H, m), 1.12-1.04 (42H, m); ^{13}C NMR δ_C(100 MHz; $CDCl_3$)/ppm 136.6, 135.9, 132.6, 132.2, 128.2, 127.7, 126.6, 126.0, 125.5, 125.3, 124.9, 124.7, 105.2, 103.8, 103.7, 98.6, 98.6, 95.4, 93.1, 90.2, 18.7, 18.6, 11.3, 11.3; FD-LRMS *m*/*z* 796.4 (M^+, 78%), 797.4 ([M+1]$^+$, 53), 798.4 ([M+2]$^+$, 100), 799.4 ([M+3]$^+$, 59), 800.4 ([M+4]$^+$, 26), 801.4 ([M+5]$^+$, 9), 802.4 ([M+6]$^+$, 3); FD-HRMS Found: 796.38961, Calcd for $C_{47}H_{69}BrSi_3$: 796.38904.

----

16 A. A. Ouahabi, P. N. W. Baxter, J.-P. Gisselbrecht, A. De Cian, L. Brelot and N. Kyritsakas-Gruber, *J. Org. Chem.*, 2009, **74**, 4675.

(b) Preparation of **27**

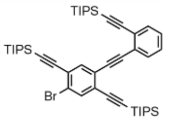

26

To a solution of **26** (4.92 g, 6.17 mmol) in diethyl ether (170 mL) was added *n*BuLi (1.6 M in hexane, 5.5 mL, 8.6 mmol) at below –80 °C under an argon atmosphere, and the mixture was stirred at that temperature for 0.5 h. A solution of I_2 (3.0 g, 12 mmol) in diethyl ether (30 mL) was added to the mixture at that temperature. After addition of 10% aq. $Na_2S_2O_3$, the organic layer was separated, dried over magnesium sulfate, and concentrated. The residue was purified by column chromatography on SiO_2 (hexane-dichloromethane/hexane) to yield **27** (4.87 g) as a white solid with 93% yield. An analytical sample was obtained as a colorless oil by purification through GPC (chloroform; JAIGEL-1H & 2H). **27**: mp 75-76 °C; ^{1}H NMR δ_H(400 MHz; $CDCl_3$; Me_4Si)/ppm 7.95 (1H, s), 7.57 (1H, s), 7.51-7.49 (2H, m), 7.30-7.23 (2H, m), 1.17-1.13 (21H, m), 1.11-1.05 (42H, m); ^{13}C NMR δ_C(100 MHz; $CDCl_3$)/ppm 142.2, 135.7, 132.6, 132.3, 129.7, 128.2, 127.7, 126.5, 126.0, 125.7, 125.5, 107.0, 105.1, 103.5, 99.5, 98.5, 97.8, 95.4, 93.4, 90.4, 18.7, 18.7, 18.6, 11.3, 11.3, 11.3; FD-LRMS *m/z* 844.4 (M^+, 100%), 845.4 ($[M+1]^+$, 71), 846.4 ($[M+2]^+$, 32), 847.4 ($[M+3]^+$, 11), 848.4 ($[M+4]^+$, 3); FD-HRMS Found: 844.37657, Calcd for $C_{47}H_{69}ISi_3$: 844.37517.

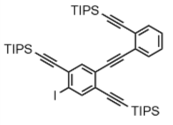

27

(c) Preparation of **28**

To a solution of **27** (4.34 g, 5.13 mmol) in Et_3N (4.3 mL) and benzene (26 mL) were added $PdCl_2(PPh_3)_2$ (216 mg, 0.308 mmol), CuI (98 mg, 0.51 mmol), TMSA (0.36 mL, 2.5 mmol), DBU (4.3 mL) and H_2O (38 µL) at room temperature under an argon atmosphere, and the mixture was stirred at 60 °C for 62.5 h. The reaction mixture was diluted with benzene and washed with 1M aq. HCl and brine, dried over magnesium sulfate and concentrated. The residue was purified by column chromatography on SiO_2 (dichloromethane/hexane) to give **28** (3.02 g) as a pale yellow amorphous solid in 81% yield. An analytical sample was obtained as a pale-yellowish-white solid by purification through GPC (chloroform; JAIGEL-2H & 2.5H). **28**: mp 219-220 °C; ^{1}H NMR δ_H(400 MHz; $CDCl_3$; Me_4Si)/ppm 7.64 (4H, s), 7.53-7.49 (4H, m), 7.30-7.25 (4H, m), 1.17-1.06 (126H, m); ^{13}C NMR δ_C(100 MHz; $CDCl_3$)/ppm 136.1, 132.5, 132.2, 128.1, 127.7, 126.1, 125.7, 125.6, 125.3, 125.3, 124.9, 105.2, 104.1, 104.1, 97.7, 97.5, 95.4, 93.9, 92.6, 90.9, 18.7, 18.7, 11.3; FD-LRMS *m/z* 1458.9 (M^+, 75%), 1459.9 ($[M+1]^+$, 100), 1460.9 ($[M+2]^+$, 84), 1461.9 ($[M+3]^+$, 50), 1462.9 ($[M+4]^+$, 25), 1463.9 ($[M+5]^+$, 10), 1464.9 ($[M+6]^+$, 4); FD-HRMS Found: 1458.93969, Calcd for $C_{96}H_{138}Si_6$: 1458.94141.

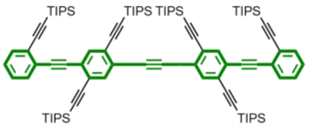

28

(d) Preparation of pr-**6** (TFA-protected)

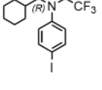

22

To a solution of **28** (1.01 g, 0.689 mmol), **22** (2.71 g, 6.37 mmol), $Pd(PPh_3)_4$ (239 mg, 0.207 mmol) and CuI (41 mg, 0.22 mmol) in Et_3N (60 mL) and THF (24 mL) was added a diluted solution of TBAF (4.3 mmol) in THF (10 mL) at 50 °C via a syringe pump over 2 h under an argon atmosphere. The filtrate was concentrated after removing a solid by filtration through a Celite pad. The residue was dissolved in ethyl acetate and washed with 1M aq. HCl and satd. aq. $NaHCO_3$, dried over magnesium sulfate and concentrated. The residue was dissolved in CH_2Cl_2 (63 mL) containing Et_3N (1 mL), which was treated with TFAA (0.8 mL),

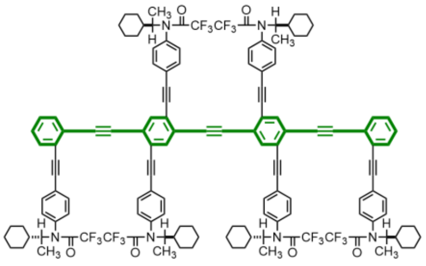

pr-**6**

followed by satd. aq. $NaHCO_3$. The organic layer was separated, dried over magnesium sulfate, and concentrated. The residue was purified by column chromatography on SiO_2 (dichloromethane-ethyl acetate/dichloromethane) to give pr-**6** (1.44 g) as a reddish-yellow amorphous solid in 91% yield. An analytic sample was obtained as a pale yellow solid by purification through GPC (chloroform; JAIGEL-2H & 2.5H), followed by suspension in methanol. pr-**6**: mp 133-135 °C; $[\alpha]_D^{24} = -12.6$ (c = 0.340, chloroform); ^{1}H NMR δ_H(400 MHz; $CDCl_3$; Me_4Si)/ppm 7.88 (2H, s), 7.87 (2H, s), 7.63 (4H, br.d), 7.52-7.44 (4H, br.m), 7.51 (4H, br.d), 7.47 (4H, br.d), 7.44-7.36 (4H, m), 7.08 (12H, br.d), 4.40-4.28 (6H, m), 1.95-1.89 (6H, br.m), 1.82-1.59 (24H, m), 1.47-1.37 (6H, m), 1.32-1.04 (24H, m), 1.08 (6H, d, J = 6.8 Hz), 1.05 (12H, d, J = 6.8 Hz), 0.99-0.84 (6H, m); ^{13}C NMR δ_C(100 MHz; $CDCl_3$; 303 K)/ppm 156.7 ($\underline{C}$(=O)CF_3), 156.7 ($\underline{C}$(=O)CF_3), 156.6 ($\underline{C}$(=O)CF_3), 136.5, 136.5, 136.0, 135.2, 135.1, 132.2 (br.), 132.2, 132.1, 130.6 (br.), 129.3 (br.), 128.9, 128.5, 126.3, 125.4, 125.4, 125.2, 125.2, 124.1, 123.6, 123.5, 116.4 ($\underline{C}F_3$), 116.4 ($\underline{C}F_3$), 94.9, 94.8, 93.1, 92.7, 90.9, 89.9, 88.8, 88.8, 60.1, 59.9, 40.3, 40.2, 30.6, 29.8, 29.7, 29.7, 26.1, 26.0, 25.9, 25.9, 25.7, 25.7, 16.3, 16.2, 16.2; FD-LRMS m/z 2304.9 (M^+, 62%), 2305.9 ($[M+1]^+$, 100), 2306.9 ($[M+2]^+$, 78), 2307.9 ($[M+3]^+$, 44), 2308.9 ($[M+4]^+$, 18), 2309.9 ($[M+5]^+$, 7); FD-HRMS Found: 2304.94726, Calcd for $C_{138}H_{126}F_{18}N_6O_6$: 2304.94514.

(e) Preparation of **6**

i) To a solution of pr-**6** (668 mg, 0.290 mmol) in THF (140 mL) were added 60% NaH in oil (1.39 g, 34.7 mmol) and MeOH (7 mL) at room temperature. The solution was stirred at that temperature for 30 min, cooled in an ice bath, diluted with dichloromethane, and quenched with water. The organic layer was separated, dried over magnesium sulfate, and concentrated. The residue was dissolved in dichloromethane, put on a SiO_2 column, and eluted with dichloromethane/hexane to give pr-**6'** as a reddish-yellow solid.

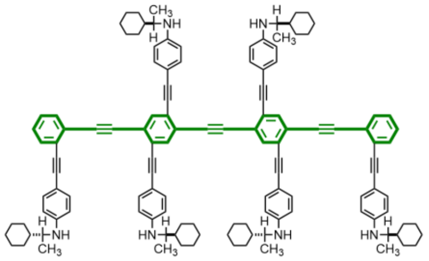

pr-**6'**

ii) To a refluxed solution of pr-**6'** in toluene (200 mL) containing Et_3N (0.3 mL) were added seven portions (199, 60, 61, 74, 76, 67, and 94 mg, a total of 3.11 mmol) of terephthaloyl chloride at an interval of 20 or 40 min. After removing the solvent by evaporation, the residue was dissolved in dichloromethane, which was washed with 1M aq. NaOH, separated and dried over magnesium sulfate. Ethyl acetate (ethyl acetate: dichloromethane = 1:9) and Al_2O_3 (30 g) were added to the mixture and filtrated through a Celite pad. The filtrate was concentrated, and the residue was purified by column chromatography on Al_2O_3/SiO_2 [1:3 (v/v), dichloromethane-tetrahydrofuran/dichloromethane] to give **6** (380 mg) as a yellow solid in 62% yield. An analytic sample was obtained as a pale yellow solid by purification through GPC (chloroform JAIGEL-2H & 2.5H), followed by suspension in ethyl acetate. **6**: mp >275 °C (decomp.); $[\alpha]_D^{23} = -437$ (c = 0.305, chloroform); ^{1}H NMR δ_H(400 MHz; $CDCl_3$; Me_4Si; 303 K)/ppm 7.88 (2H, s), 7.85 (2H, s), 7.68-7.59 (4H, m), 7.48-7.34 (16H, br.m), 6.95 (8H, s), 6.93 (4H, s), 7.1-6.4 (12H, br.m), 4.51 (6H, br.s), 2.17-2.00 (6H, br.m), 1.86-1.44 (30H, m), 1.33-0.90 (48H, m); ^{13}C NMR δ_C(100 MHz; $CDCl_3$; 303 K)/ppm 170.5, 170.4, 170.4, 141.5, 141.5, 141.0, 138.0, 138.0, 138.0, 136.3, 136.1, 132.6-132.3 (br.m), 130.9 (br.), 129.3 (br.), 128.8, 128.4, 127.1, 125.7, 125.3, 125.0, 124.9, 124.7, 121.7, 121.2, 94.9, 94.0, 92.7, 92.5, 90.4, 89.7, 88.8, 88.8, 57.8 (br.), 41.4 (br.), 30.9, 30.2, 26.2, 26.0, 16.7; FD-LRMS m/z 2119.1 (M^+,

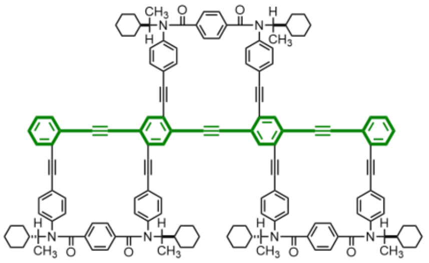

6

27%), 2120.1 ([M+1]$^+$, 78), 2121.1 ([M+2]$^+$, 100), 2122.1 ([M+3]$^+$, 83), 2123.1 ([M+4]$^+$, 43); FD-HRMS Found: 2119.06909, Calcd for $C_{150}H_{138}N_6O_6$: 2119.06778; UV λ_{max}(CH_2Cl_2)/nm (log ε) 377 (shoulder 4.88), 336 (5.30), 306 (sh. 5.11); CD λ(CH_2Cl_2)/nm ($\Delta\varepsilon$ at 293 K) 410 (+4), 385 (–31), 360 (–52), 350 (–34), 340 (–63), 324 (+120), 297 (–123), 268 (+8), 259 (+6).

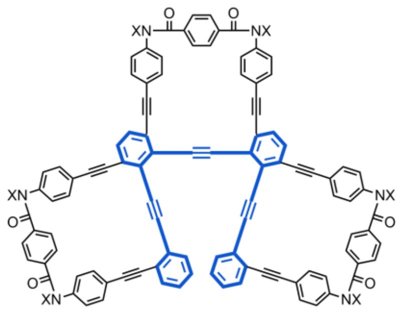

8/8B

X = (*R*)-CH(CH$_3$)(cHex)
B: X = *n*Bu

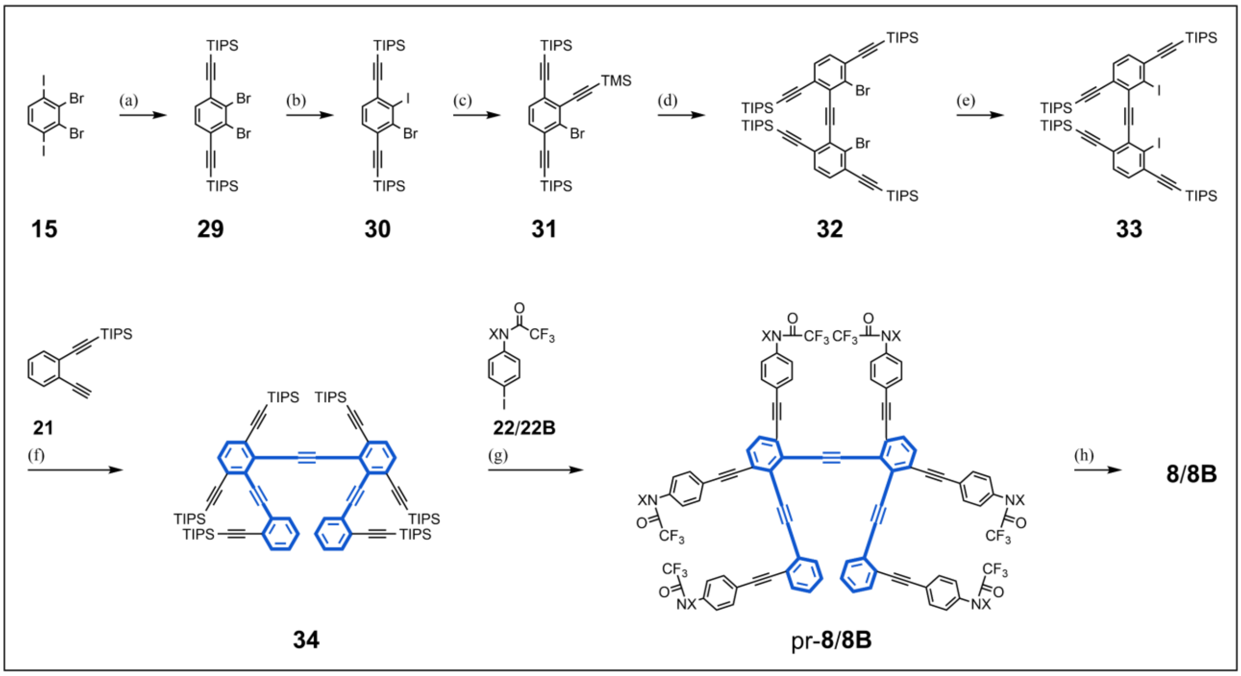

Scheme S3. Synthesis of tris(macrocycle)s **8**/**8B**. Reagents and yields: (a) triisopropylsilylacetylene (TIPSA), Pd(PPh$_3$)$_4$, CuI, Et$_3$N (**29**: 91%); (b) BuLi, I$_2$, THF (**30**: 72%); (c) TMSA, Pd(PPh$_3$)$_4$, CuI, Et$_3$N (**31**: 55%); (d) i) K$_2$CO$_3$, MeOH, THF (**31'**: 98%), ii) **30** (cont. **30H**), Pd(PPh$_3$)$_4$, CuI, Et$_3$N (**32**: 61%); (e) BuLi, I$_2$, THF (**33**: 79%); (f) **21**, Pd(PPh$_3$)$_4$, CuI, Et$_3$N (**34**: 51%); (g) **22**/**22B**, Pd(PPh$_3$)$_4$, CuI, TBAF, Et$_3$N, THF (79% for pr-**8**/61% for pr-**8B**); (h) i) NaH, MeOH, THF (79% for pr-**8'**/96% for pr-**8B'**), ii) terephthaloyl chloride, Et$_3$N, toluene (68% for **8**/59% for **8B**).

(a) Preparation of **29**

To a solution of **15** (30.0 g, 61.5 mmol) and TIPSA (34.5 mL, 155 mmol) in Et$_3$N (615 mL) were added Pd(PPh$_3$)$_4$ (3.56 g, 3.08 mmol) and CuI (1.18 g, 6.17 mmol) at 80 °C under an argon atmosphere, and the solution was stirred at that temperature for 4.5 h. The reaction mixture was passed through a Celite pad, and the filtrate was concentrated. The residue was purified by column chromatography on SiO$_2$ (hexane) and recrystallization from ethanol to give **29** (33.4 g) as colorless crystals in 91% yield. **29**: mp 126-127 °C; elemental analyses Found: C, 56.31; H, 7.42%. Calcd for C$_{28}$H$_{44}$Br$_2$Si$_2$: C, 56.37; H, 7.43%; ^{1}H NMR δ_H(400 MHz; CDCl$_3$; Me$_4$Si)/ppm 7.36 (2H, s), 1.18-1.11 (42H, m); ^{13}C NMR δ_C(100 MHz; CDCl$_3$)/ppm 131.5, 128.8, 127.2, 104.9, 99.0, 18.6, 11.3, FD-LRMS *m*/*z* 594.1 (M$^+$, 53%), 595.1 ([M+1]$^+$, 23), 596.1 ([M+2]$^+$, 100), 597.1 ([M+3]$^+$, 45), 598.1 ([M+4]$^+$, 65), 599.1 ([M+5]$^+$, 25), 600.1 ([M+6]$^+$, 8).

(b) Preparation of **30**

To a cooled solution of **29** (8.00 g, 13.4 mmol) in THF (268 mL) at –85 °C was added a solution of BuLi in hexane (1.57 M, 10.3 mL, 16.1 mmol) via a syringe and then immediately a solution of I_2 (5.11 g, 20.1 mmol) in THF (30 mL) via an additional funnel. The reaction mixture was stirred below –80 °C for 20 min and quenched with 10% aq. $Na_2S_2O_3$. The mixture was diluted with hexane and separated. The organic layer was washed with brine, dried over magnesium sulfate, and concentrated. The residue was purified by column chromatography on SiO_2 (hexane) and recrystallization from ethanol to give colorless crystals (7.02 g, **30**/**30H**[17] = 6.91) containing **30** (6.23 g, 72% yield) and **30H** (0.79 g, 10% yield), which were inseparable and subjected to the next reaction. **30**: ^{1}H NMR δ_H(400 MHz; $CDCl_3$; Me_4Si)/ppm 7.39 (1H, d, J = 8.0 Hz), 7.32 (1H, d, J = 8.0 Hz), 1.20-1.10 (42H, m); FD-LRMS *m/z* 642.1 (M^+, 89%), 643.1 ($[M+1]^+$, 37), 644.1 ($[M+2]^+$, 100), 645.1 ($[M+3]^+$, 39), 646.1 ($[M+4]^+$, 14).

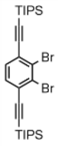

29

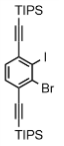

30

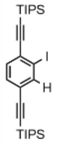

30H

(c) Preparation of **31**

To a solution of **30**+**30H** (4.00 g; 5.52 mmol + 0.799 mmol), $Pd(PPh_3)_4$ (216 mg, 0.187 mmol) and CuI (71 mg, 0.37 mmol) in Et_3N (62 mL) was added TMSA (3.5 mL, 24.8 mmol) at room temperature under an argon atmosphere. The solution was stirred at that temperature for 23 h. The filtrate was concentrated after removing a solid by filtration through a Celite pad. The residue was purified by column chromatography on SiO_2 (hexane) to give a yellow oil (1.89 g, **31**/**31H**[17] = 58.6) containing **31** (1.86 g, 55% yield based on **30**) and **31H** (28 mg, 7% yield based on **30H**), which were inseparable and subjected to the next reaction. **31**: ^{1}H NMR δ_H(400 MHz; $CDCl_3$; Me_4Si)/ppm 7.33 (1H, d, J = 8.0 Hz), 7.31 (1H, d, J = 8.0 Hz), 1.19-1.11 (42H, m), 0.26 (9H, s); FD-LRMS *m/z* 612.2 (M^+, 84%), 613.2 ($[M+1]^+$, 44), 614.2 ($[M+2]^+$, 100), 615.2 ($[M+3]^+$, 48), 616.2 ($[M+4]^+$, 22).

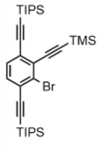

31

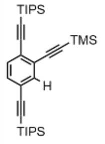

31H

(d) Preparation of **32**

i) To a solution of **31**+**31H** (1.89 g; 3.03 mmol + 0.052 mmol) in MeOH (15 mL) and THF (15 mL) was added K_2CO_3 (440 mg, 3.19 mmol) at room temperature. The mixture was stirred at that temperature for 25 min and concentrated. The residue was dissolved in dichloromethane, washed with water and brine, dried over magnesium sulfate, and concentrated. The resulting solid was purified by column chromatography on SiO_2 (hexane) to give **31'** (1.61 g) as a yellowish-white solid in 98% yield. **31'**: ^{1}H NMR δ_H(400 MHz; $CDCl_3$; Me_4Si)/ppm 7.38 (1H, d, J = 8.0 Hz), 7.35 (1H, d, J = 8.0 Hz), 3.57 (1H, s), 1.19-1.10 (42H, m); FD-LRMS *m/z* 540.2 (M^+, 90%), 541.2 ($[M+1]^+$, 38), 542.2 ($[M+2]^+$, 100), 543.2 ($[M+3]^+$, 41), 544.2 ($[M+4]^+$, 15).

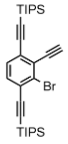

31'

ii) To a solution of **30**+**30H** (2.98 g; 4.11 mmol + 0.595 mmol), $Pd(PPh_3)_4$ (172 mg, 0.149 mmol) and CuI (56 mg, 0.29 mmol) in Et_3N (20 mL) was added a solution of **31'** (1.61 g, 2.96 mmol) in Et_3N (10 mL) at 83 °C under an argon atmosphere. The solution was stirred at that temperature for 18.5 h. After removing a solid by filtration through a Celite pad, the filtrate was concentrated, and the residue was purified through a SiO_2 column (hexane)

---

17 The ratio of products in a mixture was estimated based on peak intensities obtained using MS spectrometry.

and GPC (chloroform; JAIGEL-1H & 2H) to give a yellow amorphous solid (2.21 g, **32**/**32H**[17] = 6.09) containing **32** (1.92 g, 61% yield based on **31'**) and **32H** (291 mg, 50% yield based on **30H**), which were inseparable and subjected to the next reaction. **32**: ^{1}H NMR δ_H(400 MHz; $CDCl_3$; Me_4Si)/ppm 7.36 (4H, s), 1.18-1.10 (42H, m), 1.03-0.97 (42H, m); FD-LRMS *m/z* 1054.4 (M^+, 42%), 1055.4 ($[M+1]^+$, 35), 1056.4 ($[M+2]^+$, 100), 1057.4 ($[M+3]^+$, 78), 1058.4 ($[M+4]^+$, 83), 1059.4 ($[M+5]^+$, 53), 1060.4 ($[M+6]^+$, 27).

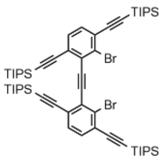

32

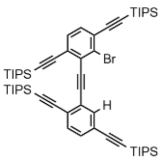

32H

(e) Preparation of **33**

To a cooled solution of **32**+**32H** (2.52 g; 2.07 mmol + 0.340 mmol) in THF (47 mL) at –85 °C were added a solution of BuLi in hexane (1.57 M, 5.0 mL, 7.9 mmol) via a syringe and then immediately a solution of I_2 (2.13 g, 8.38 mmol) in THF (12 mL) via an additional funnel, the reaction mixture was stirred at below –80 °C for 25 min and quenched with 10% aq. $Na_2S_2O_3$. The mixture was diluted with hexane and separated. The organic layer was washed with brine, dried over magnesium sulfate, and concentrated. The residue was purified by column chromatography on SiO_2 (hexane) to give a yellowish-white solid (1.99 g, **33**/**33H**[17] = 16.9) containing **33** (1.89 g, 79% yield based on **32**), **33H** (100 mg, 29% yield based on **32H**) and **33HH** (trace), which were inseparable and subjected to the next reaction. **33**: ^{1}H NMR δ_H(400 MHz; $CDCl_3$; Me_4Si)/ppm 7.40 (2H, d, J = 8.0 Hz), 7.32 (2H, d, J = 8.0 Hz), 1.20-1.11 (42H, m), 1.07-0.95 (42H, m); FD-LRMS *m/z* 1150.4 (M^+, 100%), 1151.4 ($[M+1]^+$, 86), 1152.4 ($[M+2]^+$, 48), 1153.4 ($[M+3]^+$, 22), 1154.4 ($[M+4]^+$, 8).

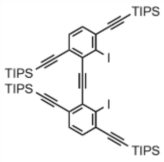

33

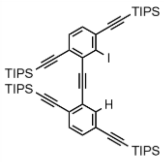

33H

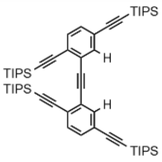

33HH

(f) Preparation of **34**

To a solution of **33**+**33H** (1.01 g; 0.830 mmol + 0.049 mmol) and **21** (1.11 g, 3.93 mmol) in Et_3N (17 mL) were added $Pd(PPh_3)_4$ (62 mg, 0.054 mmol) and CuI (24 mg, 0.13 mmol) at room temperature under an argon atmosphere. The solution was stirred at 80 °C for 4 h. After removing a solid by filtration through a Celite pad, the filtrate was concentrated and the residue was purified through a SiO_2 column (hexane-dichloromethane/hexane) and GPC (chloroform; JAIGEL-1H & 2H) to give a brown amorphous solid (890 mg, **34**/**34·21**/**34H**[17] = 76/17/7) containing **34** (615 mg, 51% yield based on **33**), **34·21** 1:1 adduct (164 mg, 11% yield based on **33**) and **34H** (111 mg, 193% yield based on **33H**), which were inseparable and subjected to the next reaction. **34**: ^{1}H NMR δ_H(400 MHz; $CDCl_3$; Me_4Si)/ppm 7.40-7.36 (4H, m), 7.33 (2H, d, J = 8.0 Hz), 7.30 (2H, d, J = 8.0 Hz), 7.19-7.13 (4H, m), 1.14-0.75 (126H, m); FD-LRMS *m/z* 1302.8 ($[M–(TIPS)]^+$, 78%), 1303.8 ($[M+1–(TIPS)]^+$, 74), 1304.8 ($[M+2–(TIPS)]^+$, 44), 1305.8 ($[M+3–(TIPS)]^+$, 24), 1306.8 ($[M+4–(TIPS)]^+$, 9), 1458.9 (M^+, 74%), 1459.9 ($[M+1]^+$, 100), 1460.9 ($[M+2]^+$, 86), 1461.9 ($[M+3]^+$, 58), 1462.9 ($[M+4]^+$, 31).

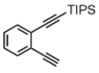

21

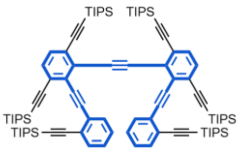

34

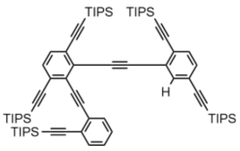

34H

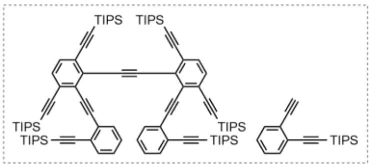

34·21 1:1 adduct

(g) Preparation of pr-**8** (TFA-protected) [X = (*R*)-CH(CH_3)(cHex)]

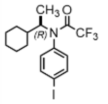

22

To a solution of **34**+**34**·**21**+**34H** (890 mg; 0.421 mmol + 0.0942 mmol + 0.0941 mmol), **22** (2.33 g, 5.49 mmol), Pd(PPh_3)$_4$ (210 mg, 0.182 mmol) and CuI (35 mg, 0.18 mmol) in Et_3N (54 mL) and THF (27 mL) was added a diluted solution of TBAF (3.8 mmol) in THF (8.8 mL) via a syringe pump over 80 min at 50 °C under an argon atmosphere. After removing a solid by filtration through a SiO_2/Celite pad, the filtrate was concentrated, and the residue was dissolved in ethyl acetate, which was washed with 1 M aq. HCl and brine, dried over magnesium sulfate and concentrated. The resulting solid was dissolved in CH_2Cl_2 (3 mL) containing Et_3N (0.7 mL), which was treated with TFAA (0.6 mL), followed by satd. aq. $NaHCO_3$. The organic layer was separated, washed with brine, dried over magnesium sulfate, and concentrated. The residue was purified by column chromatography on SiO_2 (dichloromethane-ethyl acetate/dichloromethane). A part of the product was further purified by GPC (chloroform; JAIGEL-2H & 2.5H) to give pr-**8** (769 mg) as a yellow amorphous solid in 79% yield. pr-**8**: mp >137 °C (decomp.); $[\alpha]_D^{25}$ = +3.7 (c = 0.211, chloroform); ^{1}H NMR δ_H(400 MHz; $CDCl_3$; Me_4Si)/ppm 7.53 (2H, d, J = 8.4 Hz), 7.51 (2H, d, J = 8.4 Hz), 7.46-7.28 (2H, br.m), 7.41-7.30 (12H, br.m), 7.18 (2H, br.t), 7.07-6.84 (12H, br.m), 4.40-4.30 (4H, m), 4.28-4.21 (2H, m), 2.0-0.8 (66H, m); ^{13}C NMR δ_C(100 MHz; $CDCl_3$)/ppm 156.7 (C(=O)CF_3), 136.2 (br.m), 135.7 (br.), 132.4 (br.m), 132.1 (br.m), 131.9, 131.5, 131.2, 130.4 (br.m), 129.0 (br.m), 128.9, 128.3, 128.0, 125.9, 125.4, 125.1, 124.1, 123.7, 123.7, 116.4 (CF_3), 116.4 (CF_3), 97.4, 95.1, 94.7, 94.6, 92.4, 90.2, 90.2, 89.6, 89.5, 60.0, 59.9, 40.2, 40.2, 30.6, 30.5, 29.8, 26.1, 25.9, 25.8, 25.8, 25.6, 16.3, 16.3, 16.1; FD-LRMS m/z 2304.9 (M^+, 64%), 2306.1 ($[M+1]^+$, 100), 2307.0 ($[M+2]^+$, 82), 2308.0 ($[M+3]^+$, 46), 2309.0 ($[M+4]^+$, 31); FD-HRMS Found: 2304.94334, Calcd for $C_{138}H_{126}F_{18}N_6O_6$: 2304.94514.

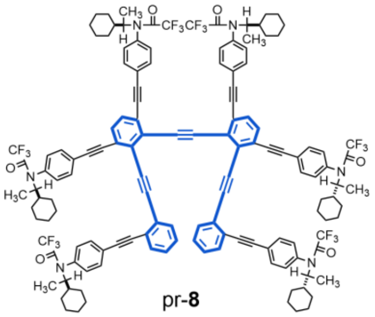

pr-**8**

(g) Preparation of pr-**8B** (TFA-protected) [X = nBu]

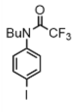

22B

To a solution of **34**+**34**·**21**+**34H** (717 mg; 0.447 mmol + 0.026 mmol + 0.016 mmol), **22B** (1.64 g, 4.42 mmol), Pd(PPh_3)$_4$ (170 mg, 0.147 mmol) and CuI (32 mg, 0.17 mmol) in Et_3N (44 mL) and THF (22 mL) was added a diluted solution of TBAF (3.1 mmol) in THF (5.1 mL) via a syringe pump over 80 min at 50 °C under an argon atmosphere. After removing a solid by filtration through a SiO_2/Celite pad, the filtrate was concentrated, and the residue was dissolved in ethyl acetate, which was washed with 1 M aq. HCl and brine, dried over magnesium sulfate and concentrated. The resulting solid was dissolved in CH_2Cl_2 (2.5 mL) containing Et_3N (0.6 mL), which was treated with TFAA (0.5 mL), followed by satd. aq. $NaHCO_3$. The organic layer was separated, washed with brine, dried over magnesium sulfate, and concentrated. The residue was purified by column chromatography on SiO_2 (dichloromethane-ethyl acetate/dichloromethane) and GPC (chloroform; JAIGEL-2H & 2.5H) to give pr-**8B** (542 mg) as a yellow amorphous solid in 61% yield. pr-**8B**: mp >110-128 °C (decomp.); ^{1}H NMR δ_H(400 MHz; $CDCl_3$)/ppm 7.53 (2H, d, J = 8.0 Hz), 7.50 (2H, d, J = 8.0 Hz), 7.43 (4H, d, J = 8.4 Hz), 7.40-7.31 (14H, br.m), 7.18 (2H, br.t), 7.06 (4H, d, J = 8.0 Hz), 7.02 (4H, d, J = 8.4 Hz), 6.98 (4H, d, J = 8.4 Hz), 3.68 (4H, t, J = 7.6 Hz), 3.65 (4H, t, J = 7.6 Hz), 3.59 (4H, t, J = 7.6 Hz), 0.90 (6H, t, J = 7.2 Hz), 0.89 (6H, t, J = 7.6 Hz), 0.84 (6H, t, J = 7.2 Hz); ^{13}C NMR

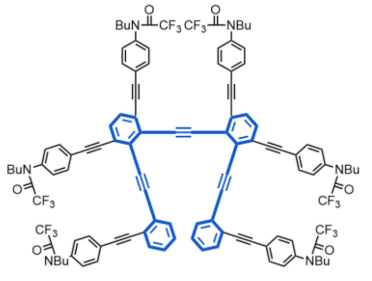

pr-**8B**

δ_C(100 MHz; $CDCl_3$)/ppm 156.3 (C(=O)CF_3), 139.1, 139.0, 138.5, 132.8, 132.8, 132.6, 132.4, 131.9, 131.4, 131.2, 128.8, 128.3, 128.2, 128.0, 128.0, 127.8, 125.8, 125.8, 125.3, 125.0, 123.9, 123.5 (br.m), 116.3 (CF_3), 97.4, 95.0, 94.7, 94.6, 92.3, 90.2, 90.1, 89.4 (br.m), 51.6, 51.5, 51.5, 29.8, 19.8, 19.7, 13.6, 13.6; FD-LRMS *m*/*z* 1980.7 (M^+, 78%), 1981.7 ($[M+1]^+$, 100), 1982.7 ($[M+2]^+$, 70), 1983.7 ($[M+3]^+$, 32), 1984.7 ($[M+4]^+$, 18); FD-HRMS Found: 1980.66442, Calcd for $C_{114}H_{90}F_{18}N_6O_6$: 1980.66344.

(h) Preparation of **8** [X = (*R*)-CH(CH_3)(cHex)]

i) To an ice-cooled solution of pr-**8** (769 mg, 0.333 mmol) in THF (40 mL) were added 60% NaH in oil (1.61 g, 40.1 mmol) and MeOH (2.6 mL). The solution was stirred at room temperature for 20 min and diluted with dichloromethane, which was washed with water and satd. aq. $NaHCO_3$. The organic layer was dried over magnesium sulfate and passed through an Al_2O_3/SiO_2 pad (Al_2O_3 was packed on SiO_2). The filtrate was concentrated, and the residue was purified by column chromatography on SiO_2 (hexane-dichloromethane) to give pr-**8'** (457 mg) as a brownish-yellow solid in 79% yield.

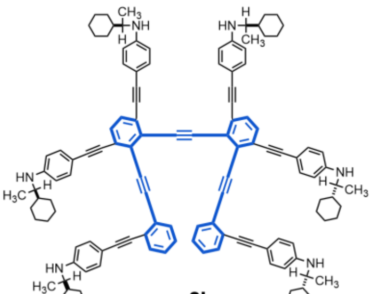

pr-**8'**

ii) To a refluxed solution of pr-**8'** (457 mg, 0.264 mmol) in toluene (106 mL) containing Et_3N (0.3 mL) were added four portions (176, 176, 174 and 176 mg, a total of 3.46 mmol) of terephthaloyl chloride at an interval of 15 min. The reaction mixture was diluted with dichloromethane and quenched with 1M aq. NaOH at room temperature. The organic layer was dried over magnesium sulfate and passed through an Al_2O_3 pad. The filtrate was concentrated, and the residue was purified by column chromatography on Al_2O_3/SiO_2 [Al_2O_3/SiO_2 = 1:1 (v/v); dichloromethane-tetrahydrofuran/dichloromethane = 1:4] to give **8** (383 mg) as a pale yellowish-white solid in 68% yield. An analytical sample was obtained as a white solid by purification through GPC (chloroform; JAIGEL-2H & 2.5H) and HPLC (methanol/dichloromethane = 7:93). **8**: mp >280 °C (decomp.); $[\alpha]_D^{25}$ = –485 (*c* = 0.251, chloroform); ^{1}H NMR δ_H(400 MHz; $CDCl_3$; Me_4Si)/ppm not assigned too broadened; ^{13}C NMR δ_C(100 MHz; $CDCl_3$/ppm; two diastereomeric conformers $(M)_B$-**8** and $(P)_B$-**8**) 170.5, 170.4, 170.3, 170.1, 141.6, 141.2, 141.1, 140.7, 140.7, 138.0, 138.0, 137.9, 137.9, 137.9, 133.1, 132.9, 132.5, 132.3, 132.2, 132.0, 131.5, 128.3, 128.2, 128.1, 127.1, 125.4, 125.3, 125.3, 125.2, 125.1, 125.1, 124.7, 122.1, 121.3, 121.3, 121.3, 121.2, 94.7, 94.7, 94.5, 94.5, 92.3, 92.2, 90.2, 90.1, 89.4, 89.3, 57.6 (br.m), 57.0, 42.2, 42.1, 41.4 (br.m), 39.9, 31.2, 30.8, 30.6, 30.2, 30.1, 30.0, 26.2, 26.0, 25.9, 17.1, 16.8, 16.7, 16.5; FD-LRMS *m*/*z* 2008.0 ($[M–(1\text{-cyclohexylethyl})]^+$, 18%), 2009.0 ($[M+1–(1\text{-cyclohexylethyl})]^+$, 48), 2010.0 ($[M+2–(1\text{-cyclohexylethyl})]^+$, 57), 2011.0 ($[M+3–(1\text{-cyclohexylethyl})]^+$, 42), 2012.0 ($[M+4–(1\text{-cyclohexylethyl})]^+$, 20), 2013.1 ($[M+5–(1\text{-cyclohexylethyl})]^+$, 12), 2119.2 ($M^+$, 43), 2120.2 ($[M+1]^+$, 93), 2121.2 ($[M+2]^+$, 100), 2122.2 ($[M+3]^+$, 71), 2123.2 ($[M+4]^+$, 37), 2124.2 ($[M+5]^+$, 25); FD-HRMS Found: 2119.06867, Calcd for $C_{150}H_{138}N_6O_6$: 2119.06778; UV λ_{max}(CH_2Cl_2)/nm (log ε) 373 (5.00), 343 (5.22), 308 (5.07), 286 (shoulder 4.96); CD λ(CH_2Cl_2)/nm ($\Delta\varepsilon$ at 293 K) 379 (–130), 324 (+65), 277 (–134).

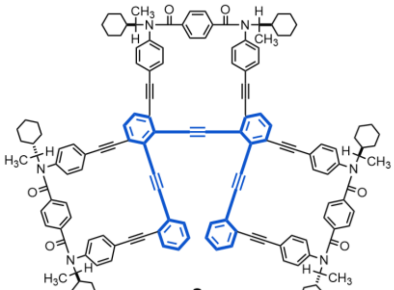

8

(h) Preparation of **8B** [X = *n*Bu]

i) To an ice-cooled solution of pr-**8B** (542 mg, 0.273 mmol) in THF (33 mL) were added 60% NaH in oil (176 mg, 4.40 mmol) and MeOH (1.1 mL). The solution was stirred at room temperature for 15 min, cooled in an ice bath, and diluted with dichloromethane, which was washed with water and satd. aq. $NaHCO_3$. The organic layer was dried over magnesium sulfate and concentrated. The residue was purified by column chromatography on Al_2O_3/SiO_2 [1:1 (v/v); dichloromethane] to give pr-**8B'** (367 mg) as a yellow solid in 96% yield.

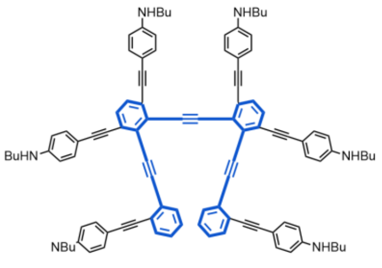

pr-**8B'**

ii) To a solution of pr-**8B'** (367 mg, 0.261 mmol) in toluene (104 mL) containing Et_3N (0.3 mL) was added terephthaloyl chloride (174 mg, 0.857 mmol) at 100 °C. The mixture was stirred at that temperature for 30 min, diluted with dichloromethane, and quenched with 1M aq. NaOH at room temperature. The organic layer was dried over magnesium sulfate and passed through an Al_2O_3 pad. The filtrate was concentrated, and the residue was purified by column chromatography on Al_2O_3/SiO_2 [Al_2O_3/SiO_2 = 1:1 (v/v); dichloromethane-tetrahydrofuran/dichloromethane = 1:4] to give **8B** (279 mg) as a pale yellowish-white solid in 59% yield. An analytical sample was obtained as a white solid by purification through GPC (chloroform; JAIGEL-2H & 2.5H) and HPLC (methanol/dichloromethane = 7:93). **8B**: mp >270 °C (decomp.); ^{1}H NMR δ_H(400 MHz; $CDCl_3$; Me_4Si)/ppm 7.69-7.56 (2H, br.m), 7.59 (2H, d, J = 8.4 Hz), 7.57 (2H, d, J = 8.4 Hz), 7.51-7.30 (2H, br.m), 7.42 (2H, d, J = 8.8 Hz), 7.39 (2H, d, J = 8.8 Hz), 7.36 (2H, d, J = 8.8 Hz), 7.14-6.92 (4H, br.m), 6.98 (8H, s), 6.97 (4H, s), 6.79 (12H, br.s), 4.12-4.05 (4H, m), 3.94-3.70 (8H, br.m), 3.59-3.52 (4H, m), 1.58-1.40 (12H, m), 1.40-1.28 (12H, m), 0.87 (6H, t, J = 7.2 Hz), 0.86 (6H, t, J = 7.6 Hz), 0.84 (6H, t, J = 7.6 Hz); ^{13}C NMR δ_C(100 MHz; $CDCl_3$)/ppm 170.0, 169.9, 143.2, 142.7, 137.4, 137.4, 137.3, 133.1, 132.8, 132.7, 132.6, 132.3, 132.1, 131.5, 128.5, 128.3, 128.3, 128.2, 127.3, 125.4, 125.3, 125.0, 124.8, 121.8, 121.2, 121.1, 97.1, 94.7, 94.7, 94.5, 92.2, 90.1, 89.9, 89.2, 49.1, 49.1, 29.8, 29.7, 20.1 (br.m), 13.8, 13.7, 13.7; FD-LRMS *m*/*z* 1794.9 (M^+, 67%), 1795.9 ($[M+1]^+$, 100), 1796.9 ($[M+2]^+$, 78), 1797.9 ($[M+3]^+$, 41), 1798.9 ($[M+4]^+$, 17); FD-HRMS Found: 1794.78452, Calcd for $C_{126}H_{102}N_6O_6$: 1794.78608; UV λ_{max}(CH_2Cl_2)/nm (log ε) 371 (4.99), 343 (5.21), 308 (5.07), 286 (shoulder 4.96).

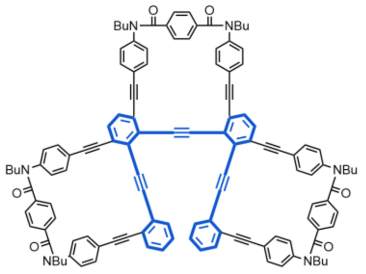

8B
