# Supplementary material for: Study of optical rotation based on the molecular structure in fused oligomers of macrocycles
Source: RSC Adv. 2024 Jul 1;14(29):20735–42. doi: 10.1039/d4ra03709j (PMC11215750; doi:10.1039/d4ra03709j)
Supplement: RA-014-D4RA03709J-s002 [file RA-014-D4RA03709J-s002.pdf]

Electronic Supplementary Material (ESI) for RSC Advances.
This journal is © The Royal Society of Chemistry 2024

Electronic Supplementary Information_2

Consideration of optical rotation based on the molecular structure in fused oligomers of macrocycles

Ryo Katoono*, Yudai Obara, Kazuki Sakamoto and Rei Miyashita
Department of Chemistry, Faculty of Science, Hokkaido University

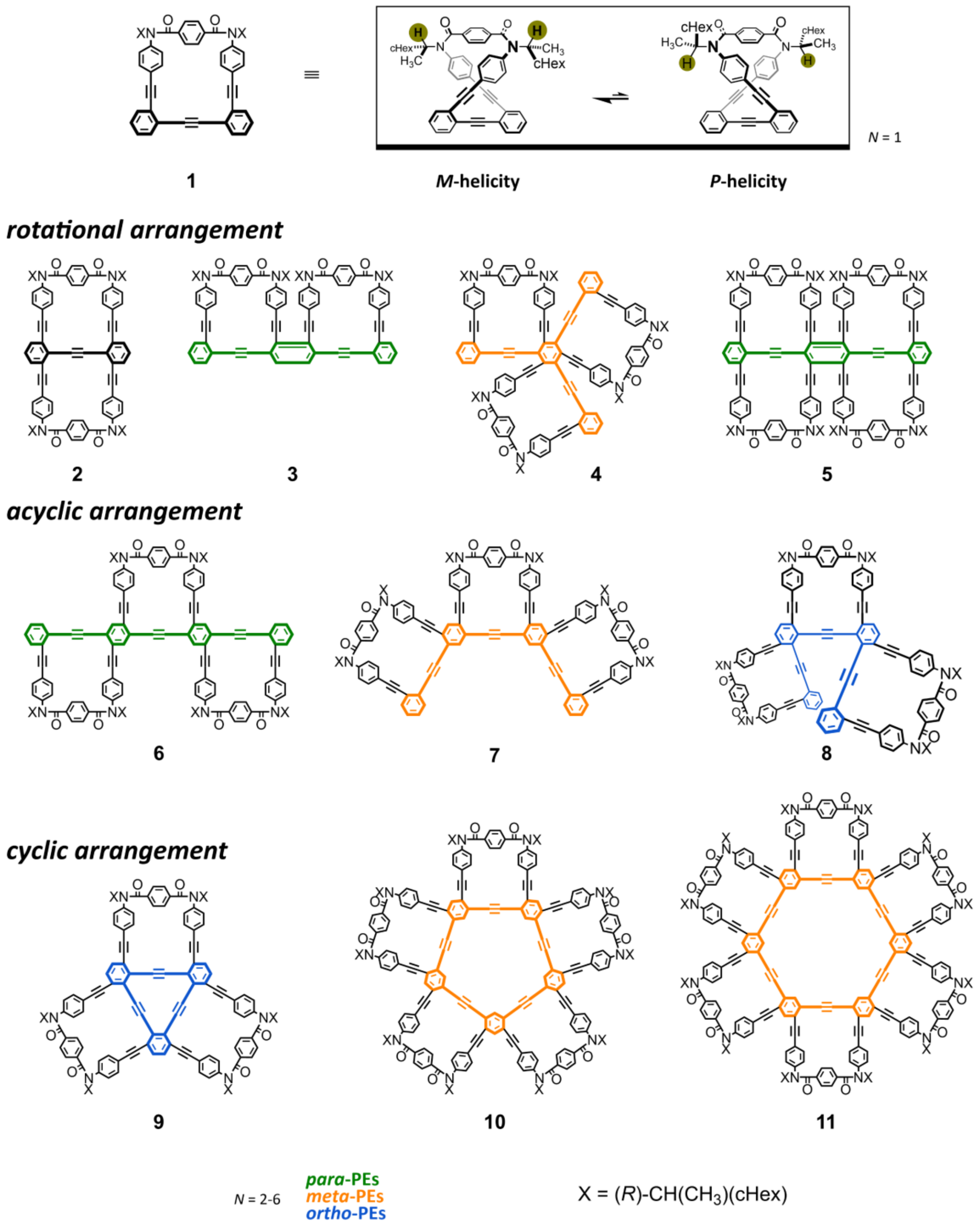

Contents (ESI_1)

Supplementary Figures (**Figures S1-S27**) page

Conformational searches using MacroModel software. S3-S9

^{1}H NMR spectra of **1-11**. S10-S16

Quantitative estimation of the diastereomeric ratio induced in **1**, **2**, **4**, and **5**. S17-S22

VT-CD spectra of **3**, **6**, **7**, **8**, and **9**. S23-S24

UV spectra of **1-11**. S25

Complexation experiment [X = butyl] with a chiral guest (**14**). S26-S35

UV and CD spectra of **3**, **6**, **7**, **8**, and **9** in the presence of an achiral guest (**13**). S36-S37

Plots of $\Delta\varepsilon/\varepsilon$ for **3**, **6**, **7**, **8**, and **9** before and after complexation (**13**). S38

Experimental (**Schemes S1-S3**) S39-S59

Contents (ESI_2)

Copies of ^{1}H/^{13}C NMR spectra and LRMS of new compounds

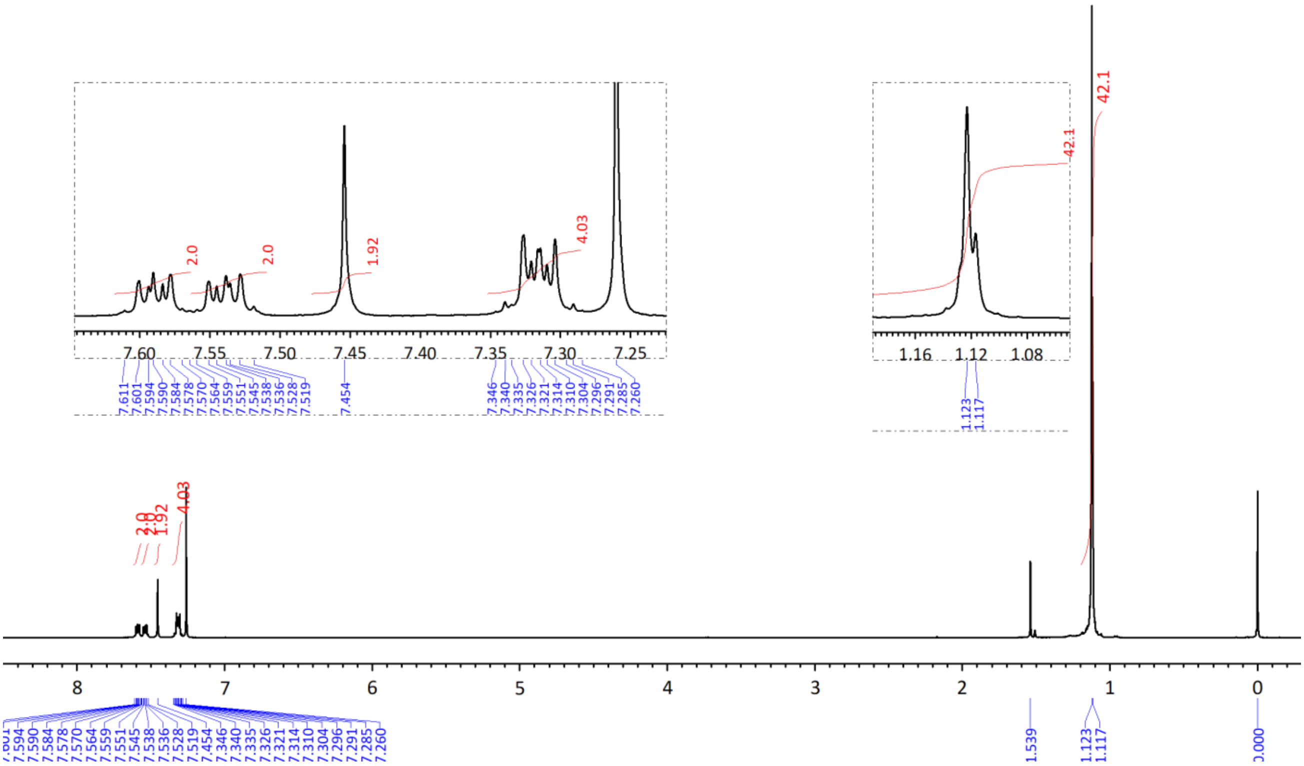

^{1}H NMR spectrum (400 MHz) of **16**, measured in $CDCl_3$ at room temperature.

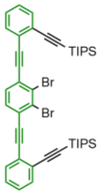

16

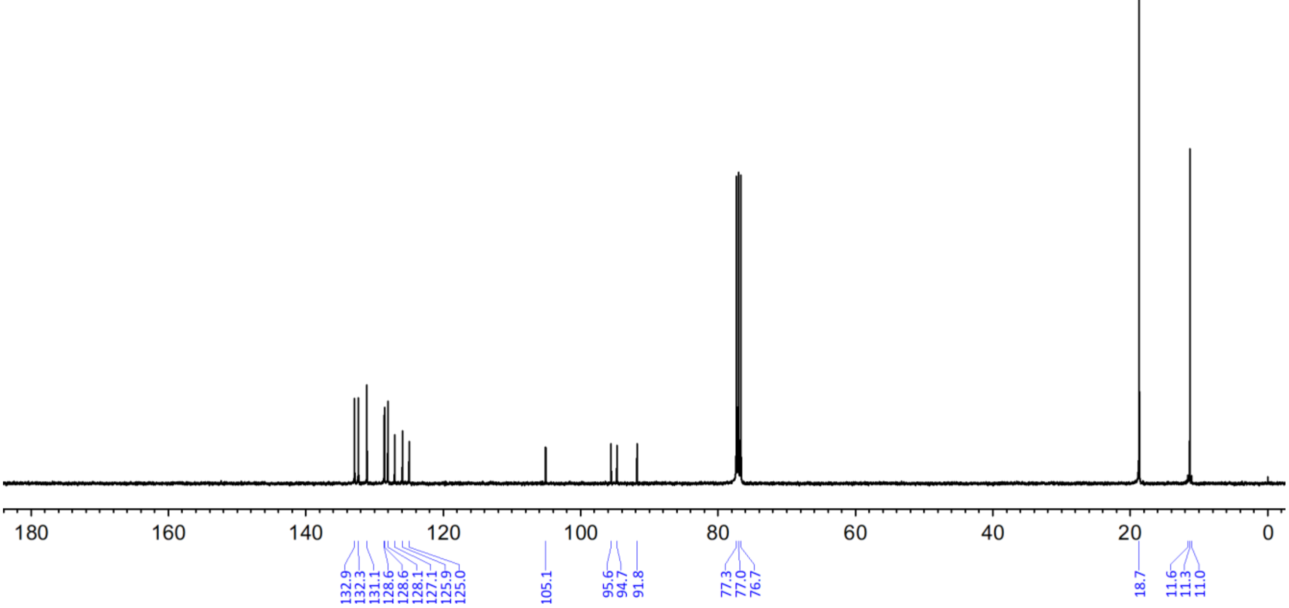

^{13}C NMR spectrum (100 MHz) of **16**, measured in $CDCl_3$ at room temperature.

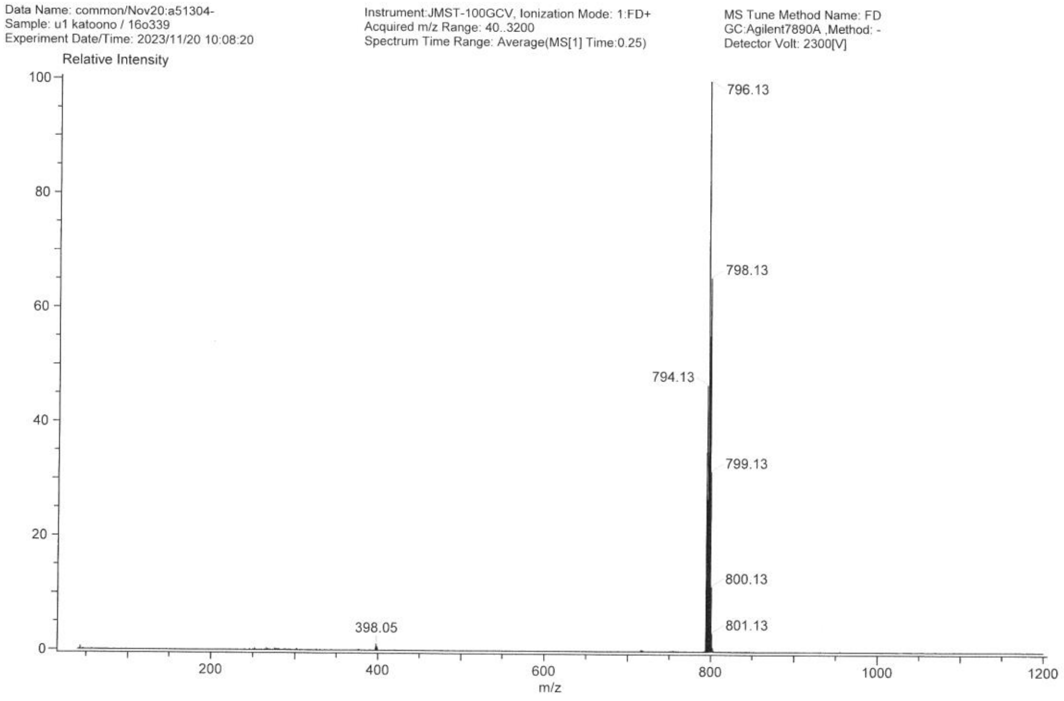
Data Name: common/Nov20:a51304-
Sample: u1 katoono / 16o339
Experiment Date/Time: 2023/11/20 10:08:20
Instrument:JMST-100GCV, Ionization Mode: 1:FD+
Acquired m/z Range: 40..3200
Spectrum Time Range: Average(MS[1] Time:0.25)
MS Tune Method Name: FD
GC:Agilent7890A ,Method: -
Detector Volt: 2300[V]
Relative Intensity
796.13
798.13
794.13
799.13
800.13
801.13
398.05
m/z

LRMS (FD) of **16**.

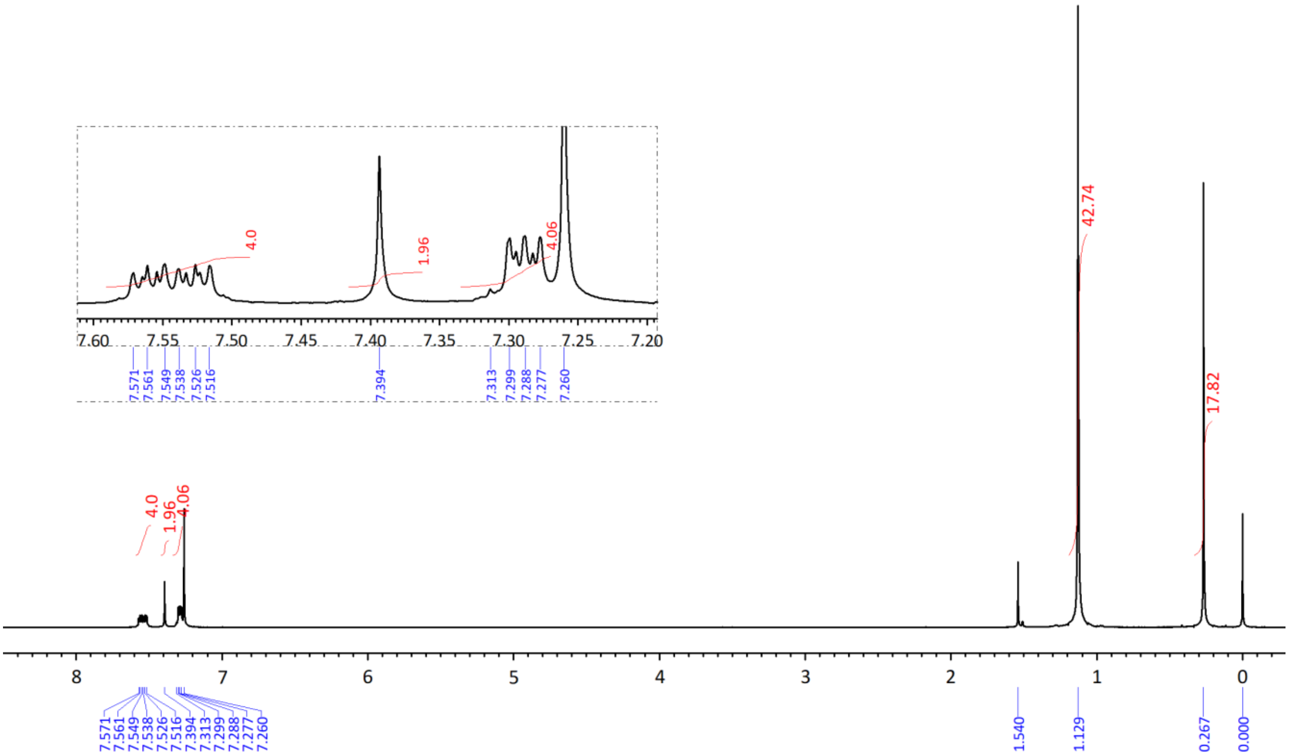

^{1}H NMR spectrum (400 MHz) of **17**, measured in $CDCl_3$ at room temperature.

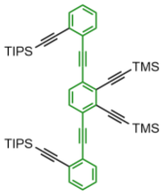

17

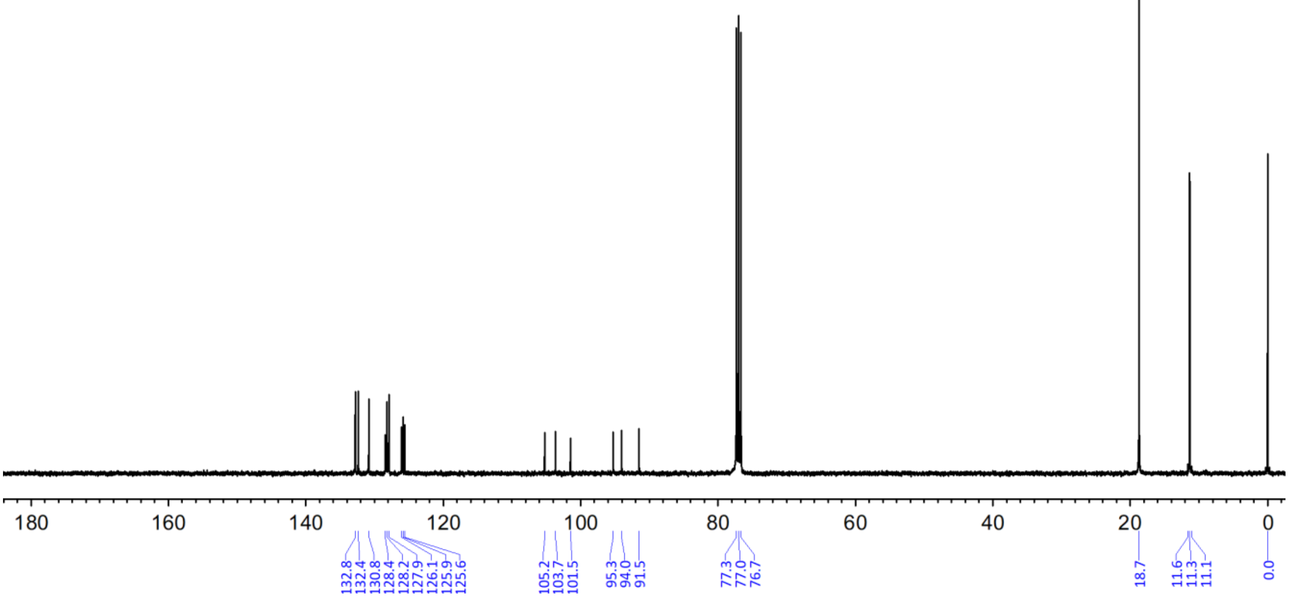

^{13}C NMR spectrum (100 MHz) of **17**, measured in $CDCl_3$ at room temperature.

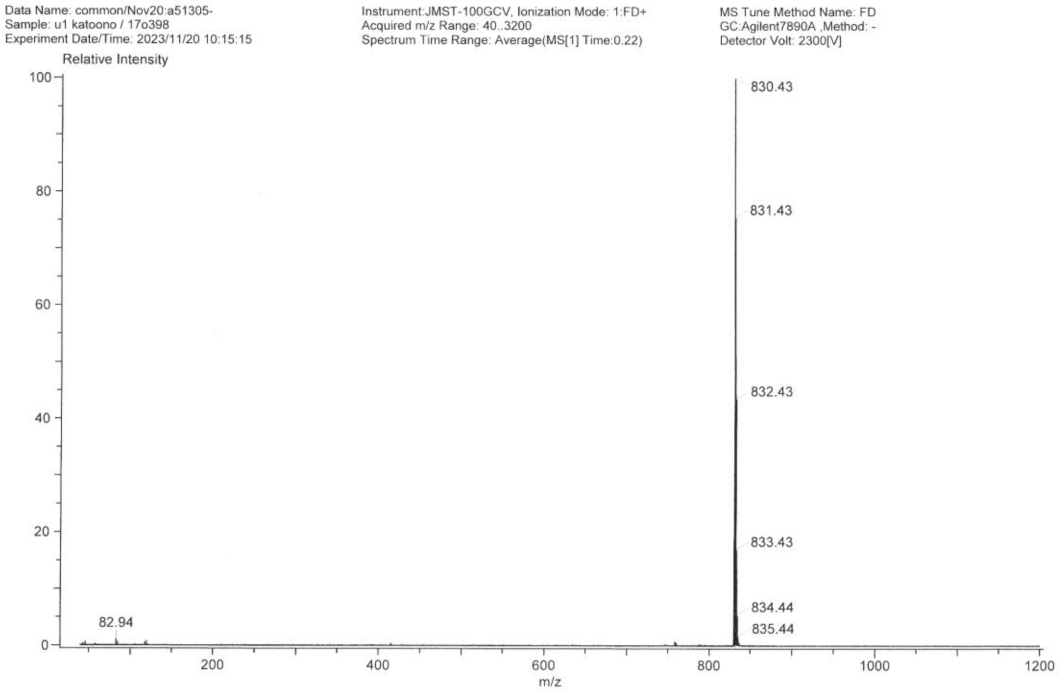
Data Name: common/Nov20:a51305-
Sample: u1 katoono / 17o398
Experiment Date/Time: 2023/11/20 10:15:15
Instrument:JMST-100GCV, Ionization Mode: 1:FD+
Acquired m/z Range: 40..3200
Spectrum Time Range: Average(MS[1] Time:0.22)
MS Tune Method Name: FD
GC:Agilent7890A ,Method: -
Detector Volt: 2300[V]
Relative Intensity
830.43
831.43
832.43
833.43
834.44
835.44
82.94
m/z

LRMS (FD) of **17**.

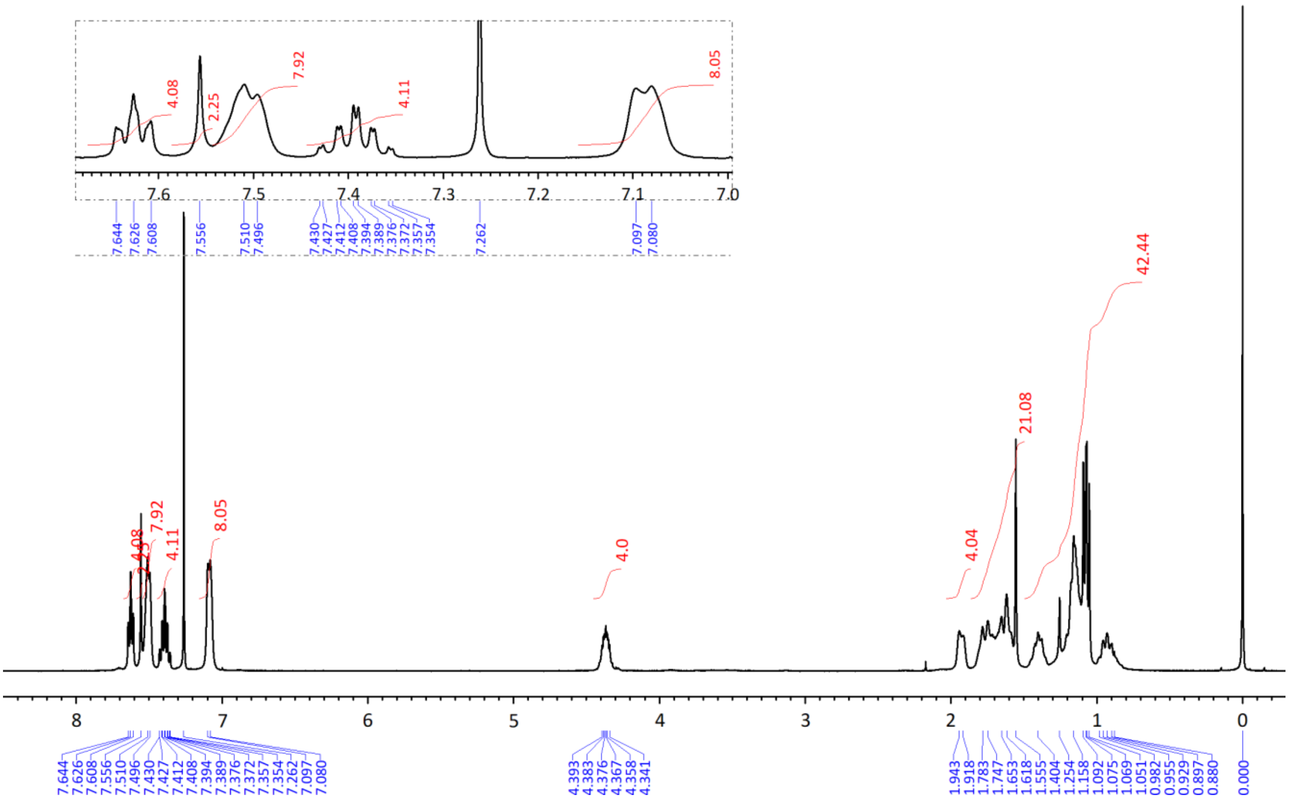

^{1}H NMR spectrum (400 MHz) of pr-**3**, measured in $CDCl_3$ at room temperature.

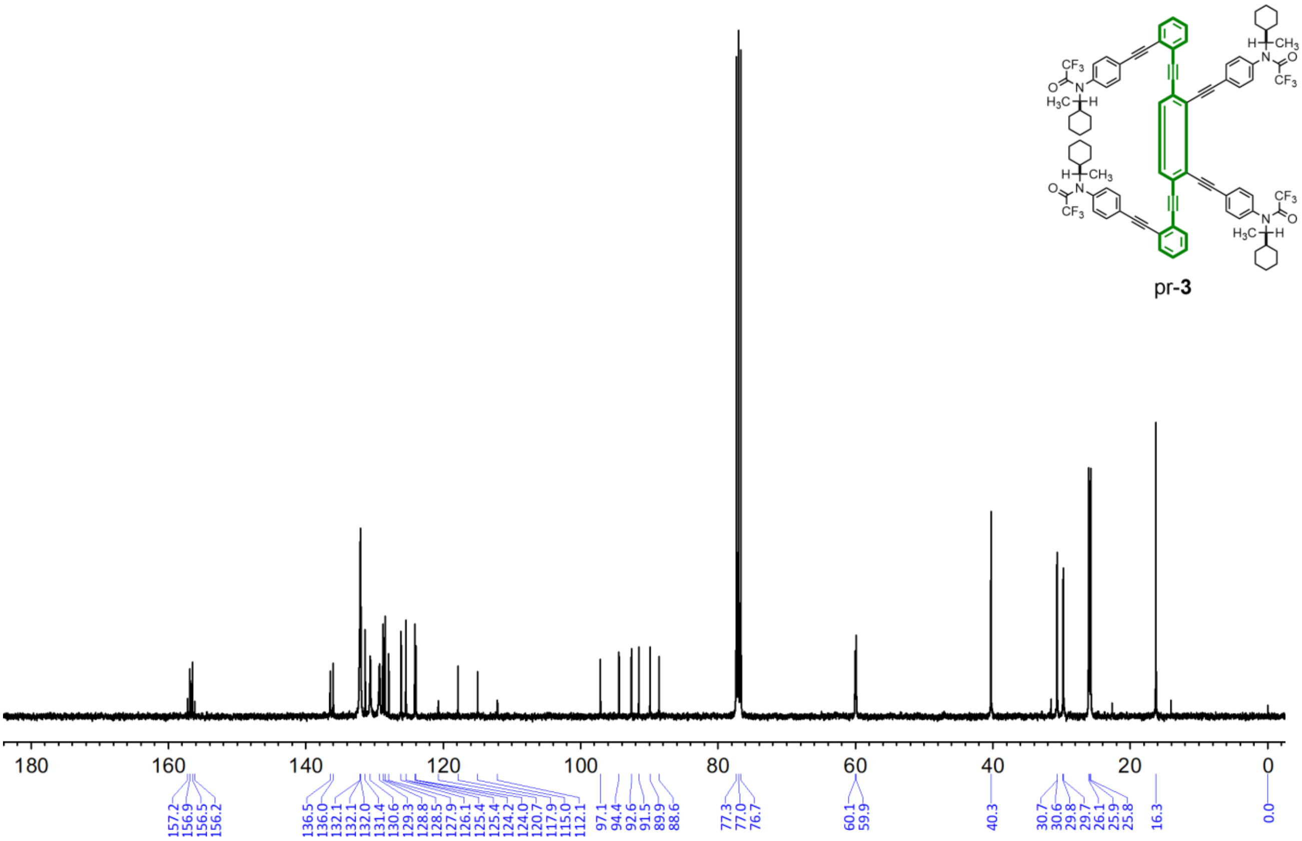

^{13}C NMR spectrum (100 MHz) of pr-**3**, measured in $CDCl_3$ at room temperature.

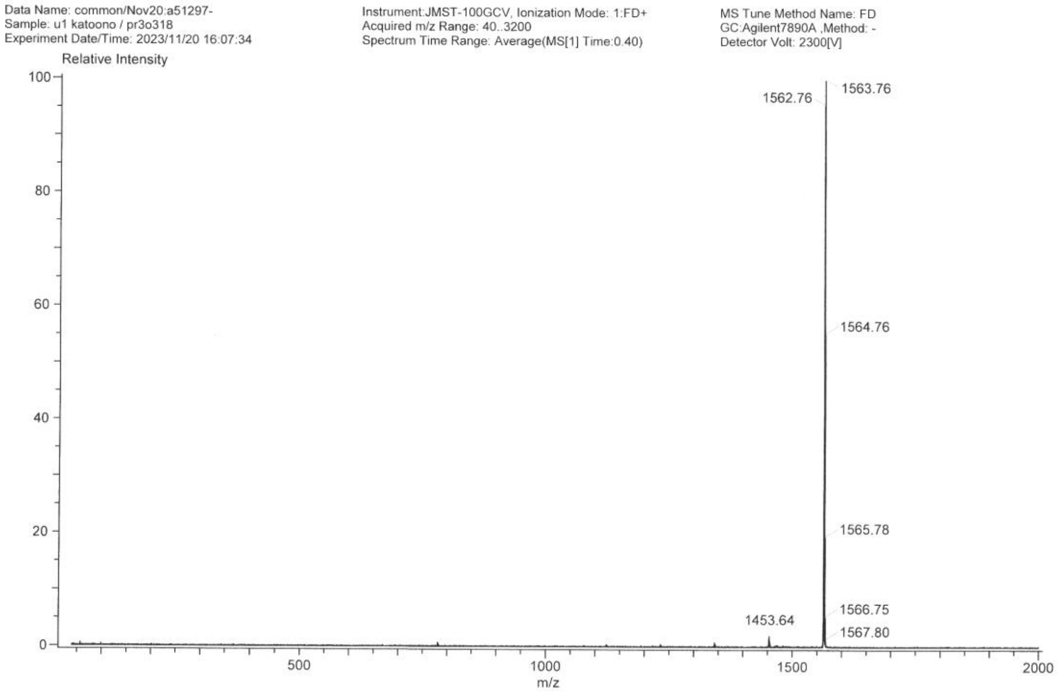

LRMS (FD) of pr-**3**.

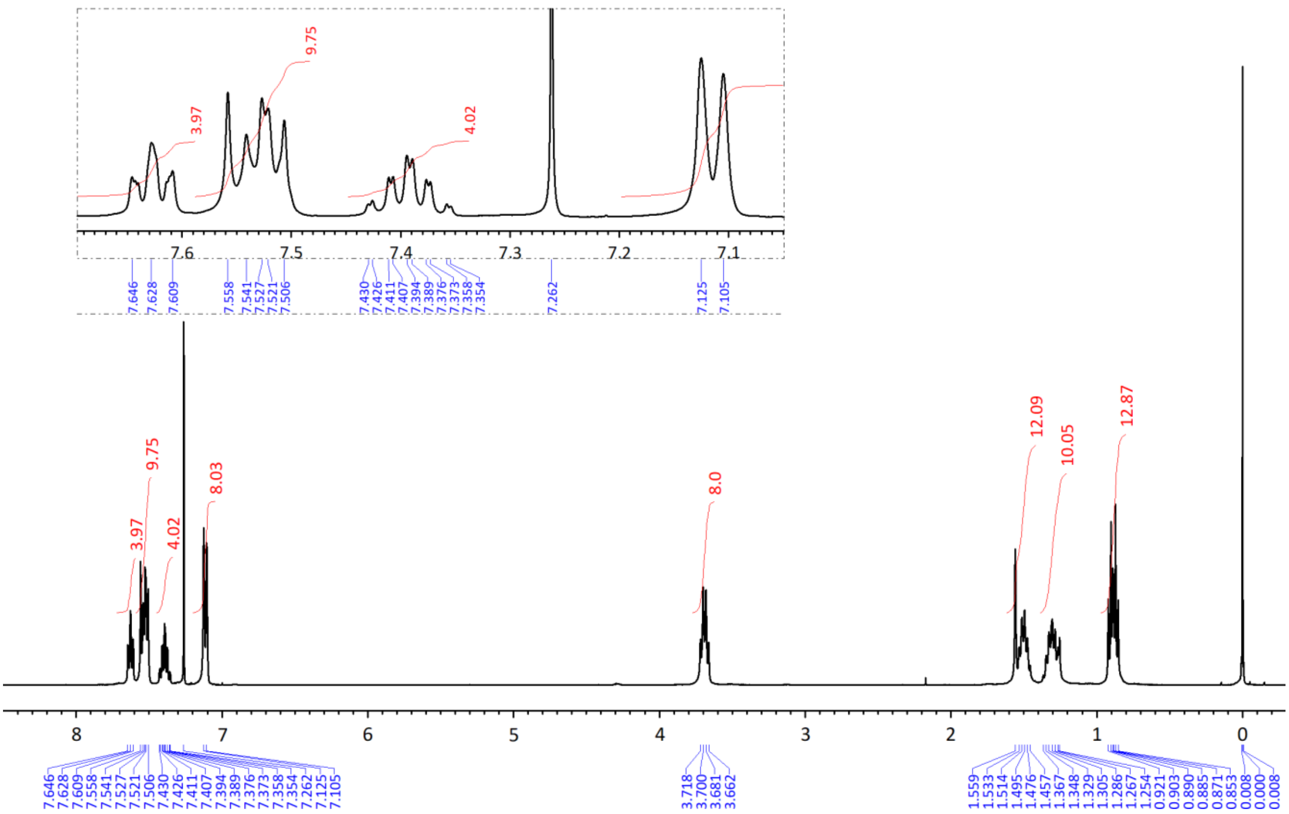

^{1}H NMR spectrum (400 MHz) of pr-**3B**, measured in $CDCl_3$ at room temperature.

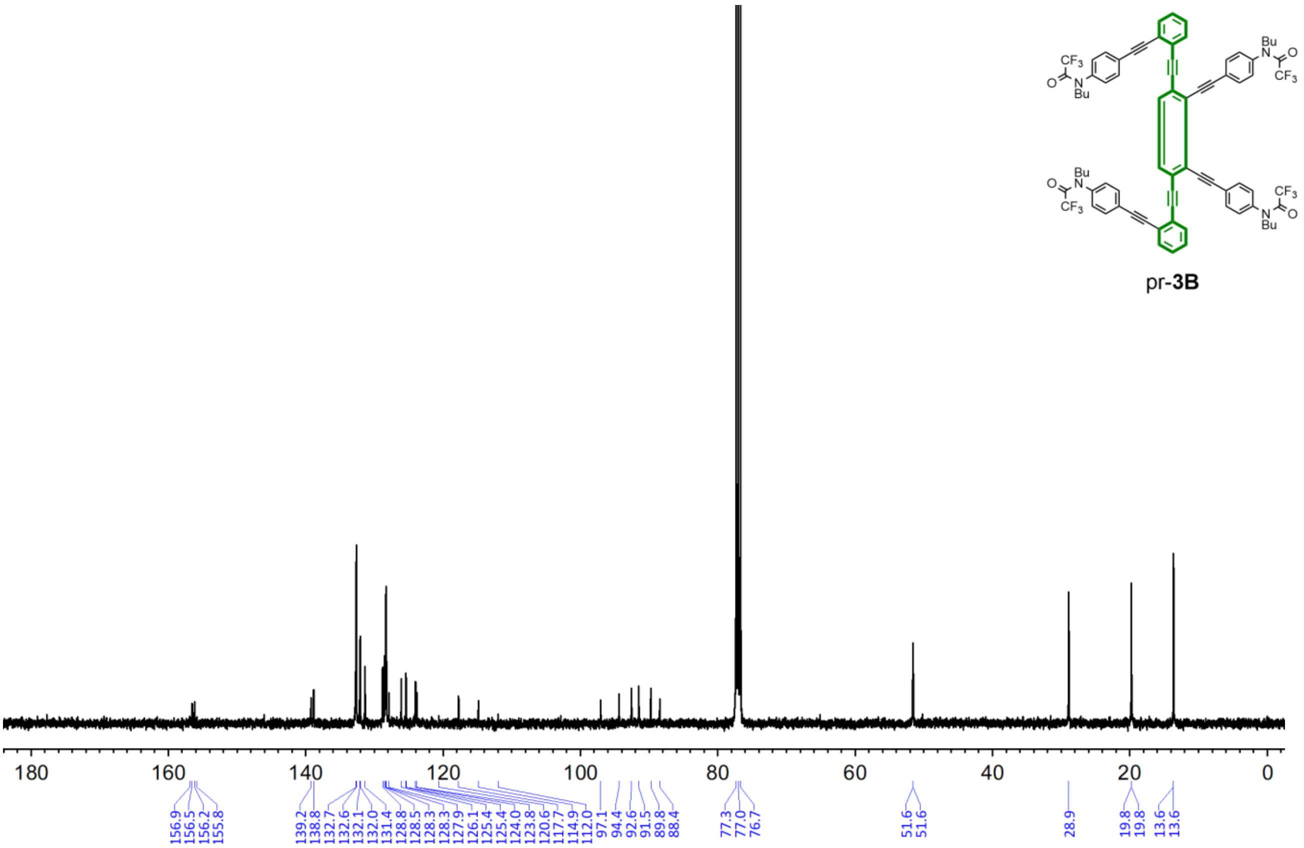

^{13}C NMR spectrum (100 MHz) of pr-**3B**, measured in $CDCl_3$ at room temperature.

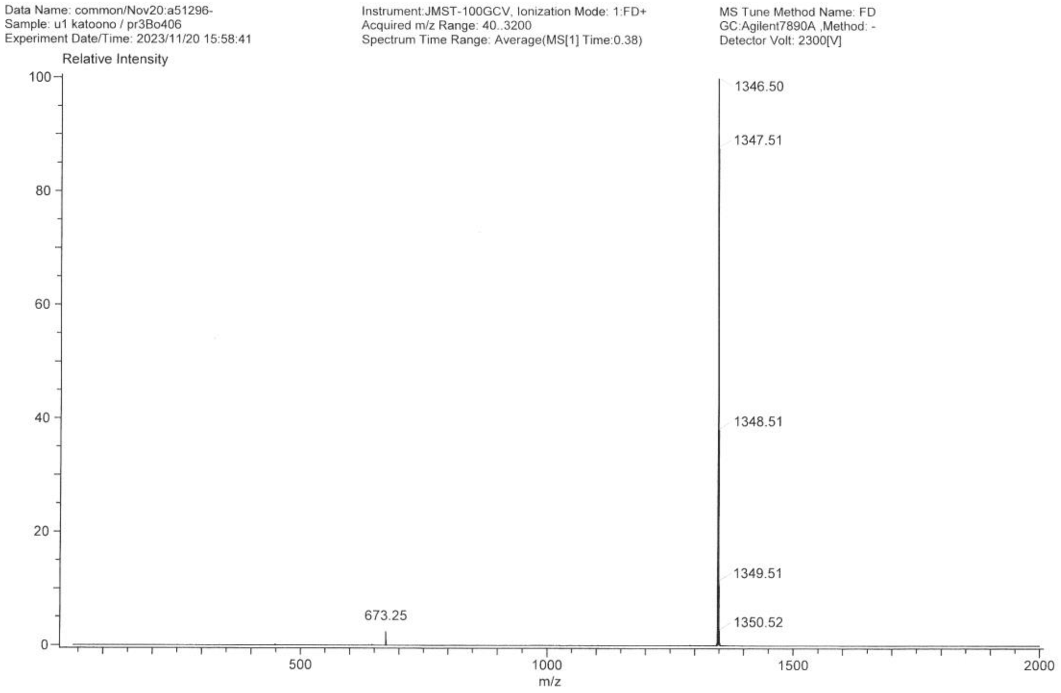

LRMS (FD) of pr-**3B**.

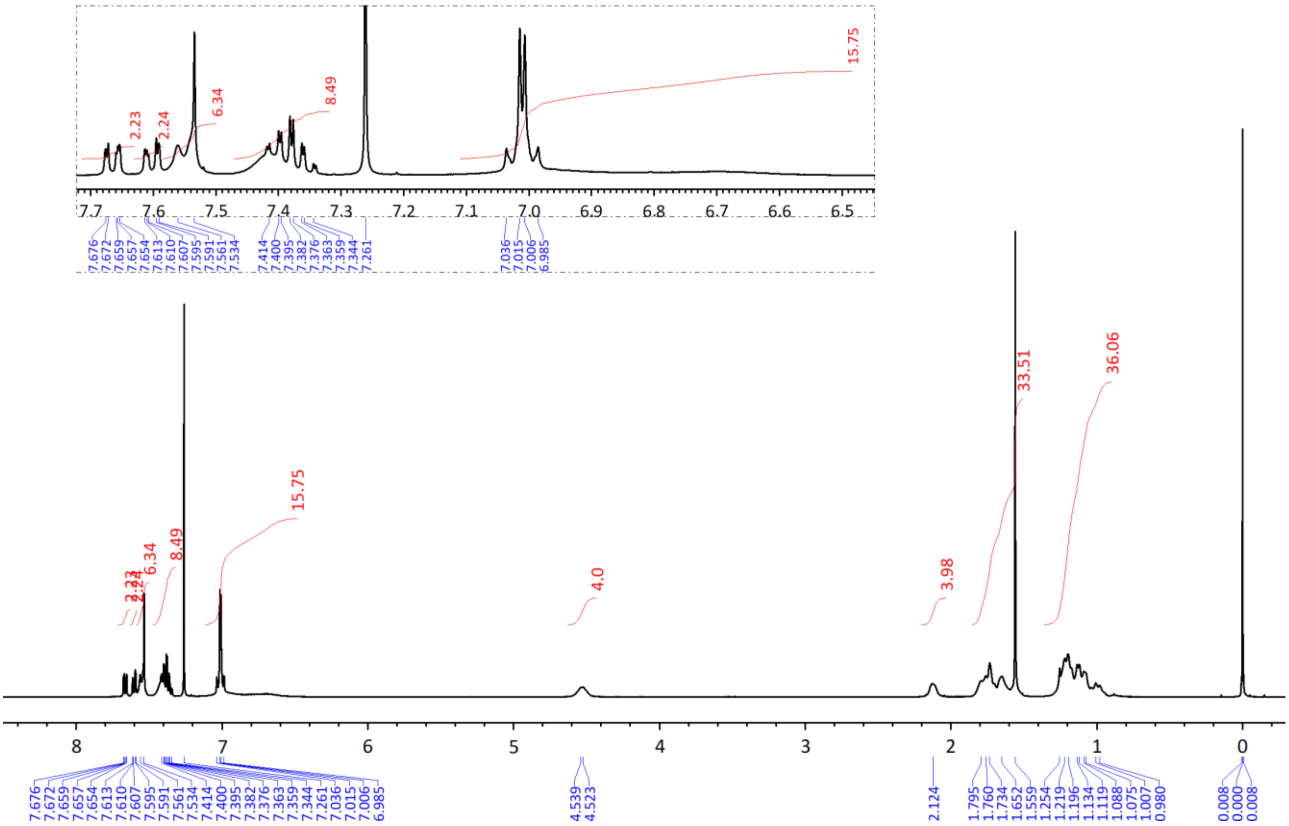

^{1}H NMR spectrum (400 MHz) of **3**, measured in $CDCl_3$ at room temperature.

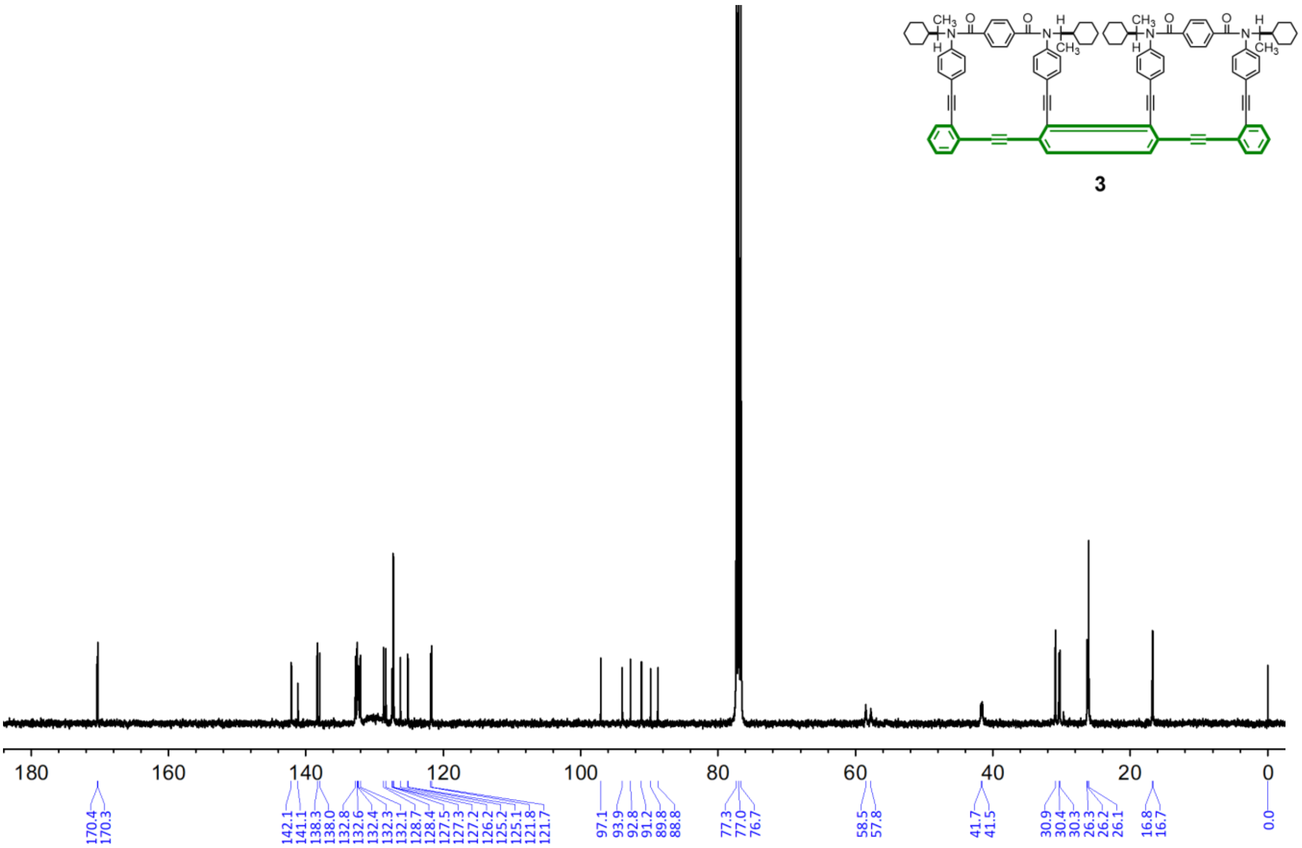

^{13}C NMR spectrum (100 MHz) of **3**, measured in $CDCl_3$ at 313 K.

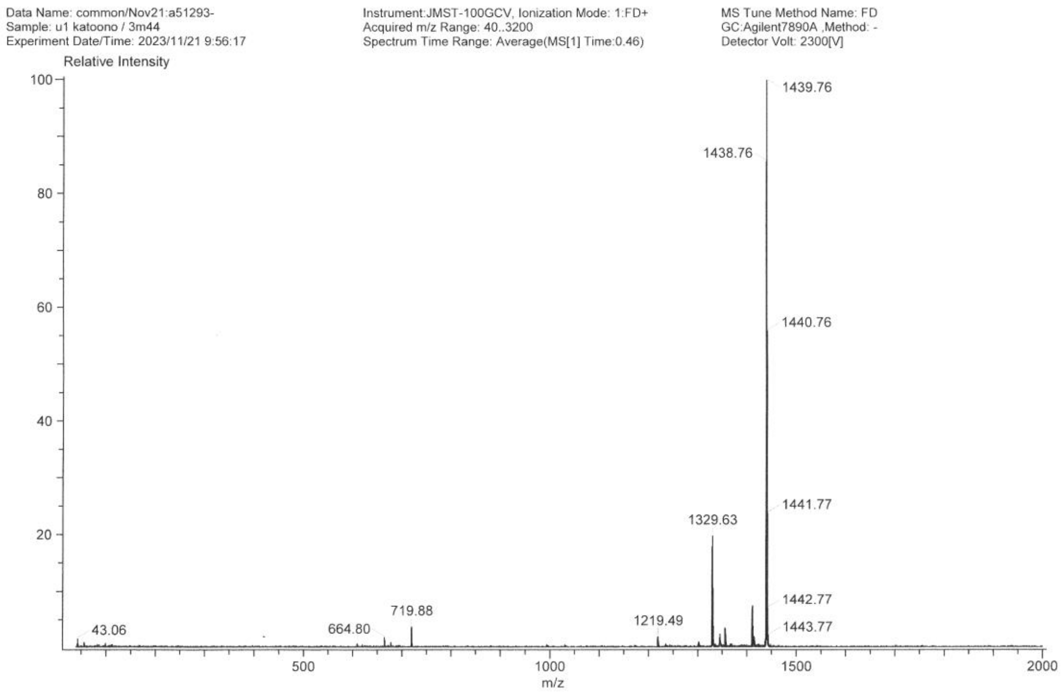
Data Name: common/Nov21:a51293-
Sample: u1 katoono / 3m44
Experiment Date/Time: 2023/11/21 9:56:17
Instrument:JMST-100GCV, Ionization Mode: 1:FD+
Acquired m/z Range: 40..3200
Spectrum Time Range: Average(MS[1] Time:0.46)
MS Tune Method Name: FD
GC:Agilent7890A ,Method: -
Detector Volt: 2300[V]
Relative Intensity
1439.76
1438.76
1440.76
1441.77
1329.63
1442.77
1443.77
1219.49
719.88
664.80
43.06
m/z

LRMS (FD) of **3**.

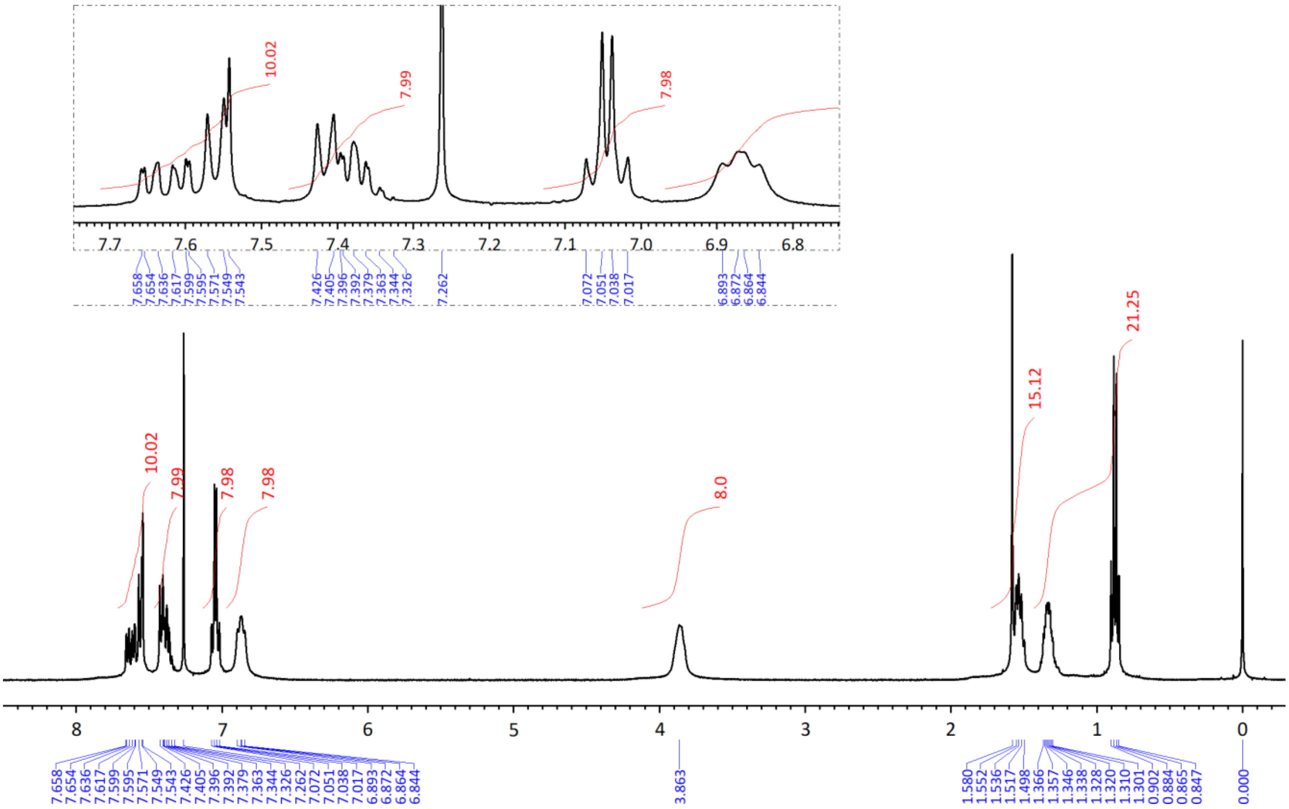

^{1}H NMR spectrum (400 MHz) of **3B**, measured in $CDCl_3$ at room temperature.

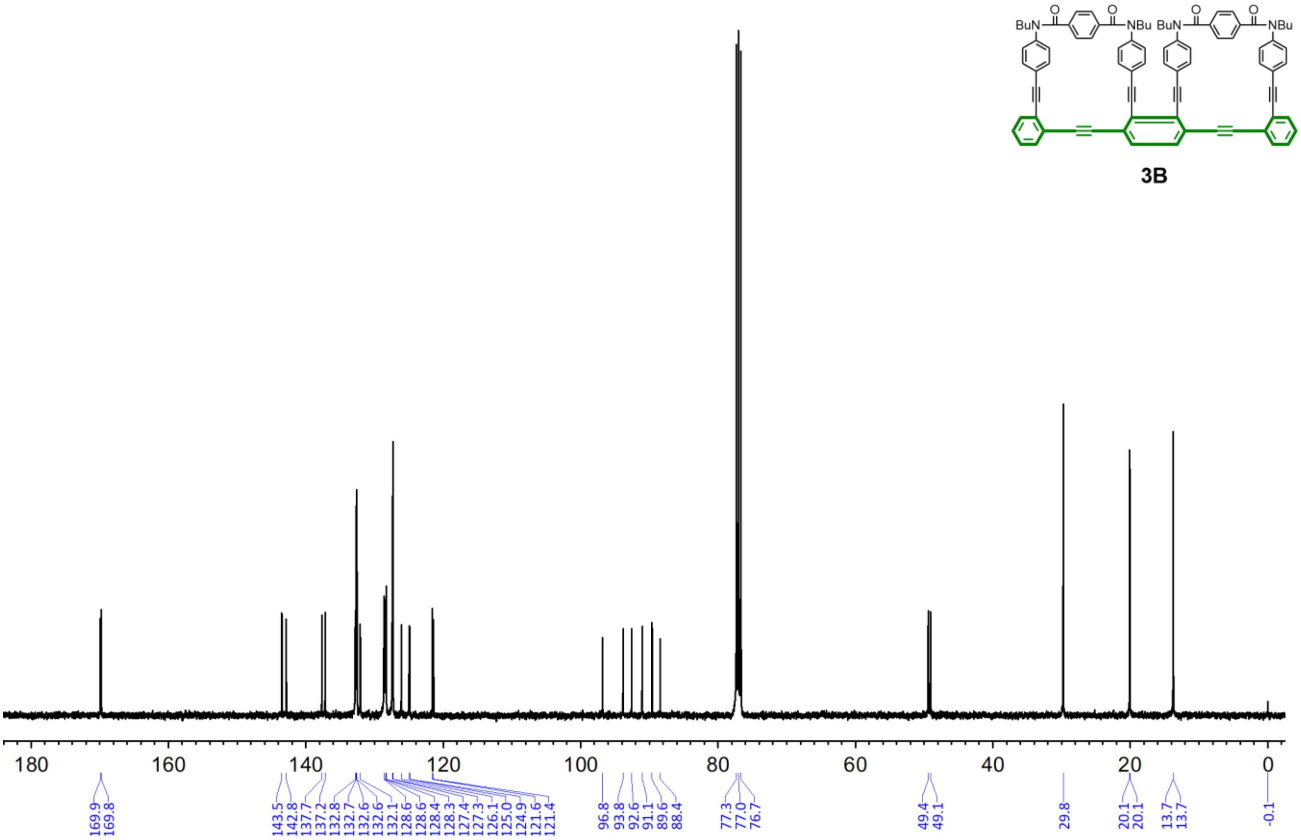

^{13}C NMR spectrum (100 MHz) of **3**, measured in $CDCl_3$ at room temperature.

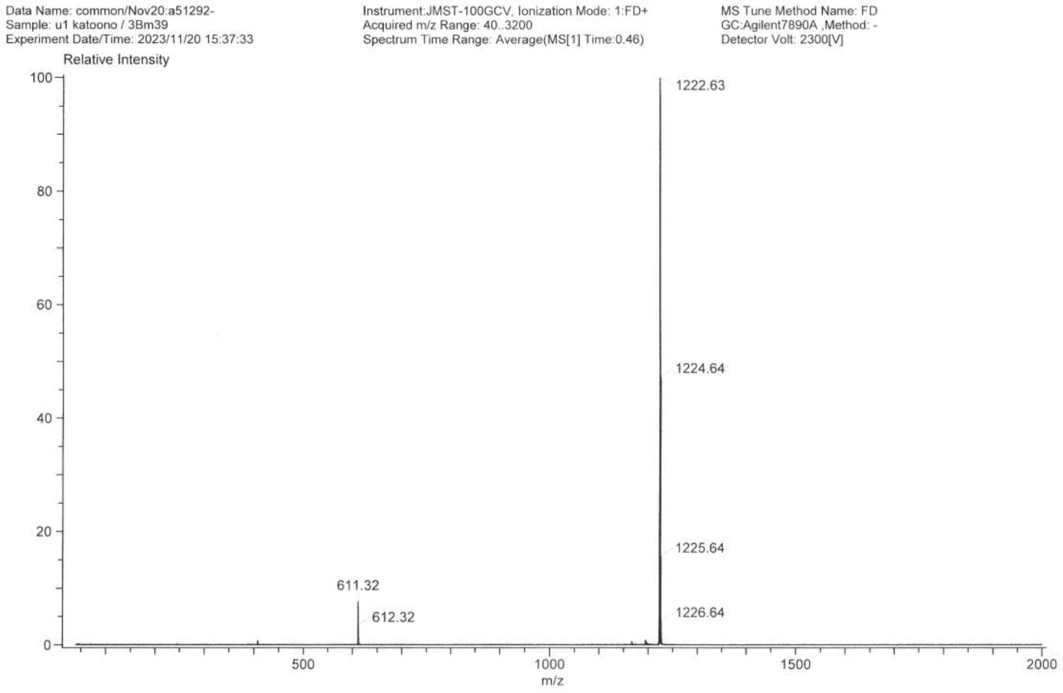
Data Name: common/Nov20:a51292-
Sample: u1 katoono / 3Bm39
Experiment Date/Time: 2023/11/20 15:37:33
Instrument:JMST-100GCV, Ionization Mode: 1:FD+
Acquired m/z Range: 40..3200
Spectrum Time Range: Average(MS[1] Time:0.46)
MS Tune Method Name: FD
GC:Agilent7890A ,Method: -
Detector Volt: 2300[V]
Relative Intensity
1222.63
1224.64
1225.64
1226.64
611.32
612.32
m/z

LRMS (FD) of **3B**.

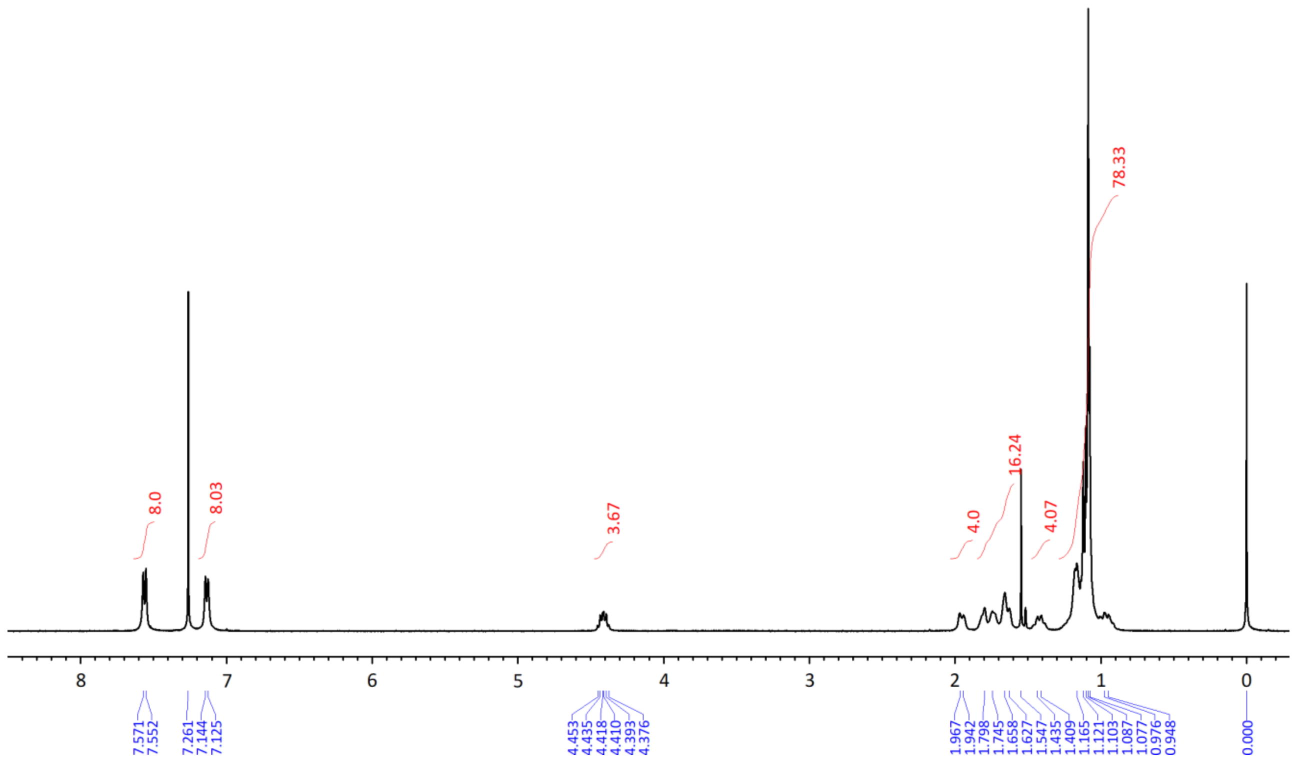

^{1}H NMR spectrum (400 MHz) of **19**, measured in $CDCl_3$ at room temperature.

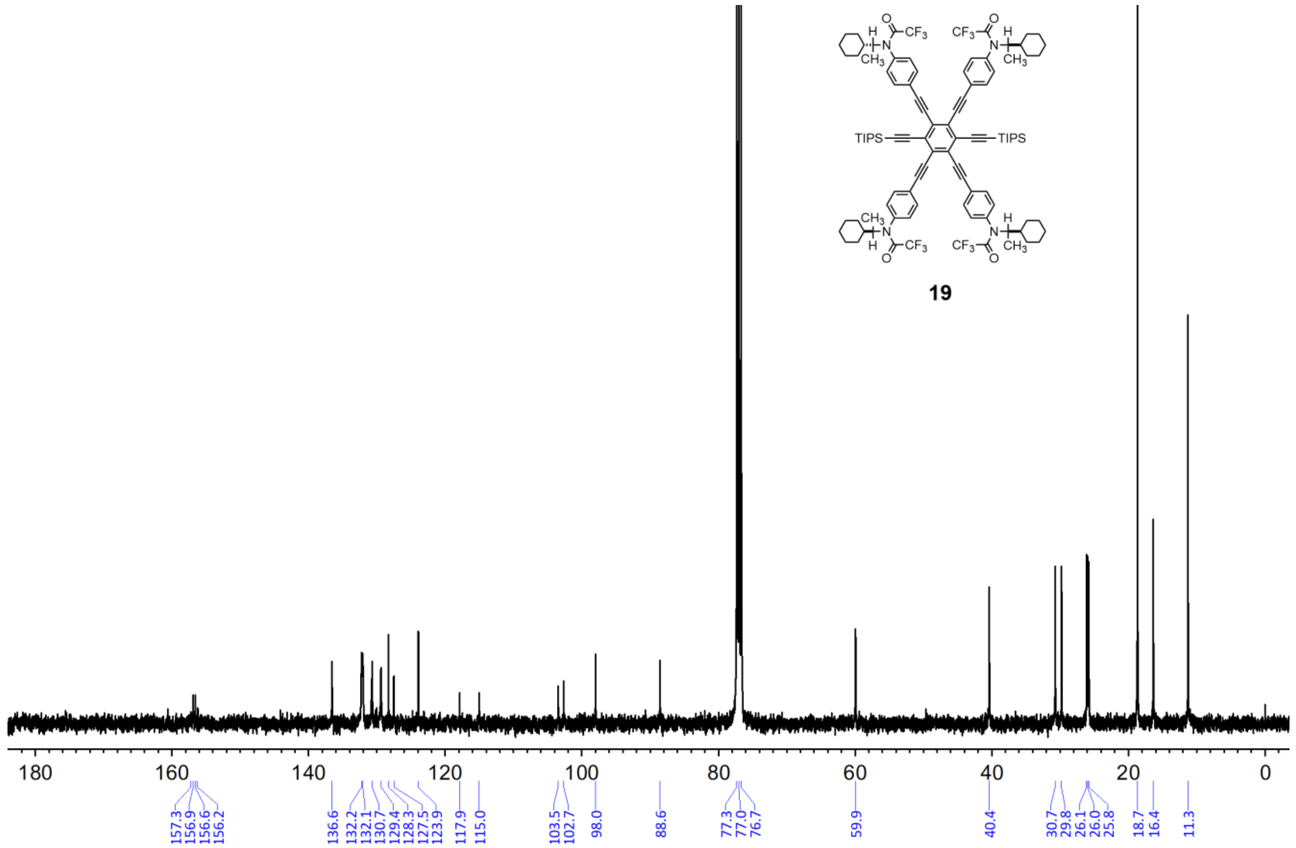

^{13}C NMR spectrum (100 MHz) of **19**, measured in $CDCl_3$ at room temperature.

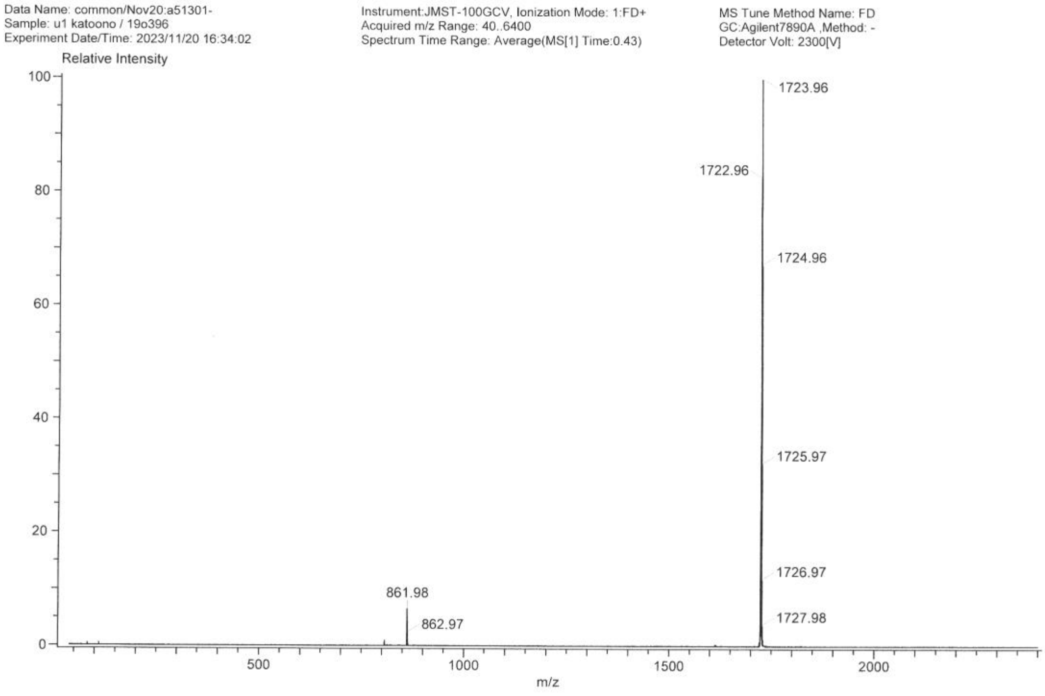

Data Name: common/Nov20:a51301-
Sample: u1 katoono / 19o396
Experiment Date/Time: 2023/11/20 16:34:02
Instrument:JMST-100GCV, Ionization Mode: 1:FD+
Acquired m/z Range: 40..6400
Spectrum Time Range: Average(MS[1] Time:0.43)
MS Tune Method Name: FD
GC:Agilent7890A ,Method: -
Detector Volt: 2300[V]
Relative Intensity
1723.96
1722.96
1724.96
1725.97
1726.97
1727.98
861.98
862.97
m/z

LRMS (FD) of **19**.

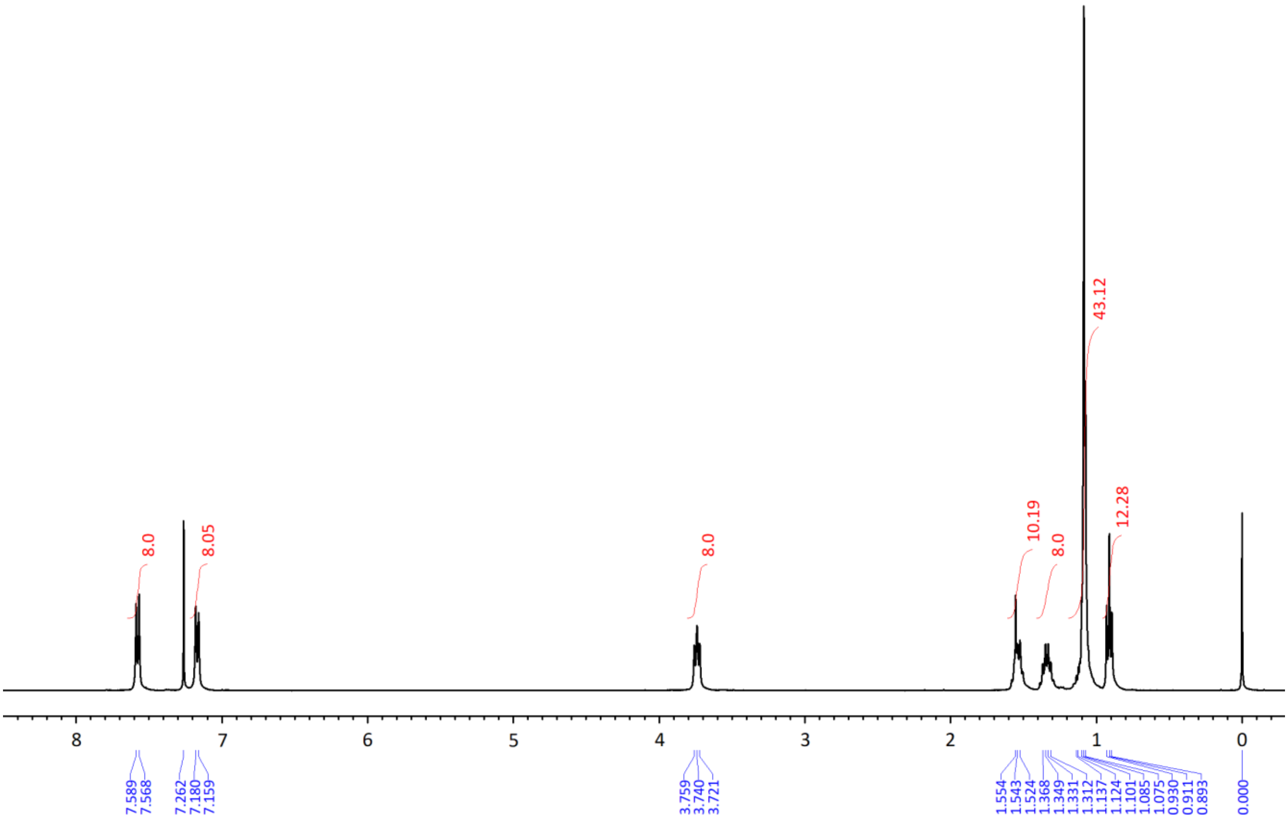

^{1}H NMR spectrum (400 MHz) of **19B**, measured in $CDCl_3$ at room temperature.

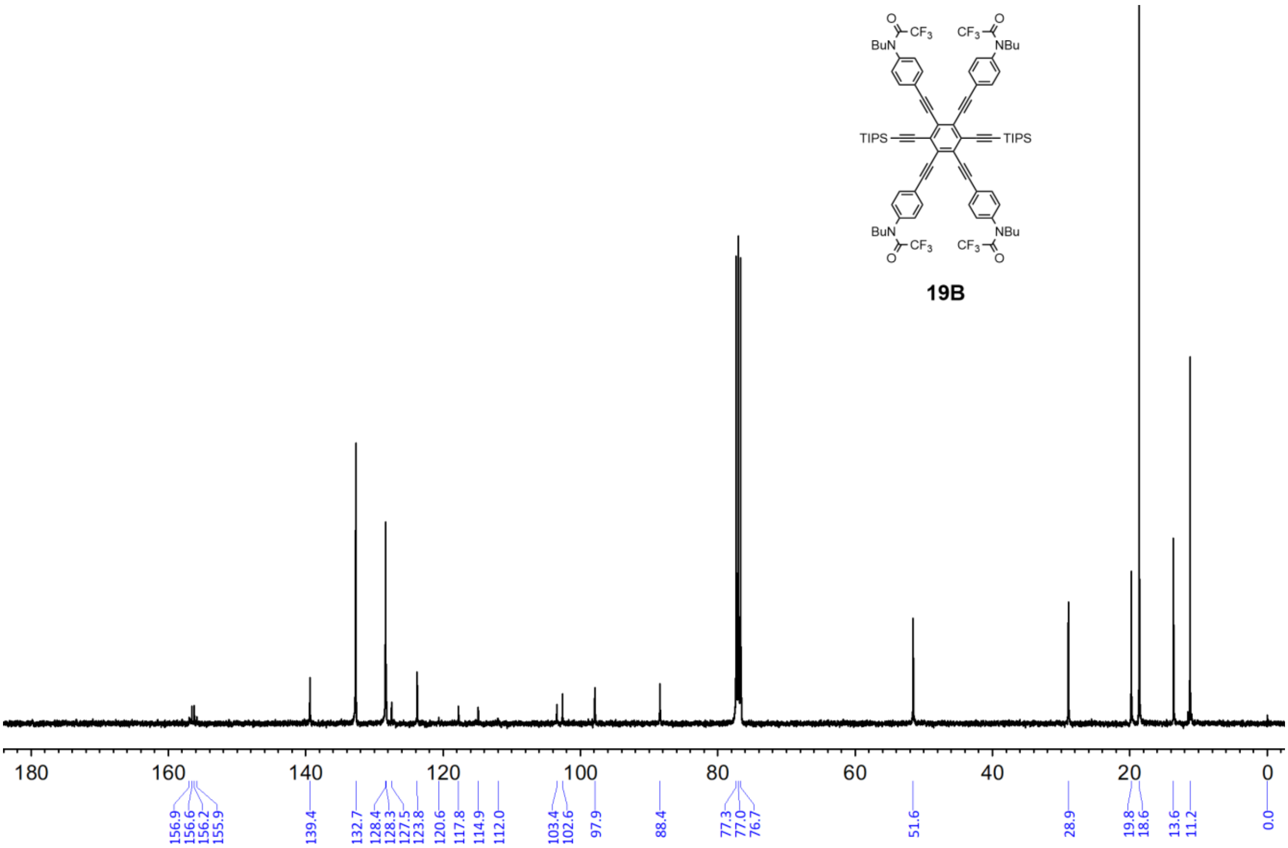

^{13}C NMR spectrum (100 MHz) of **19B**, measured in $CDCl_3$ at room temperature.

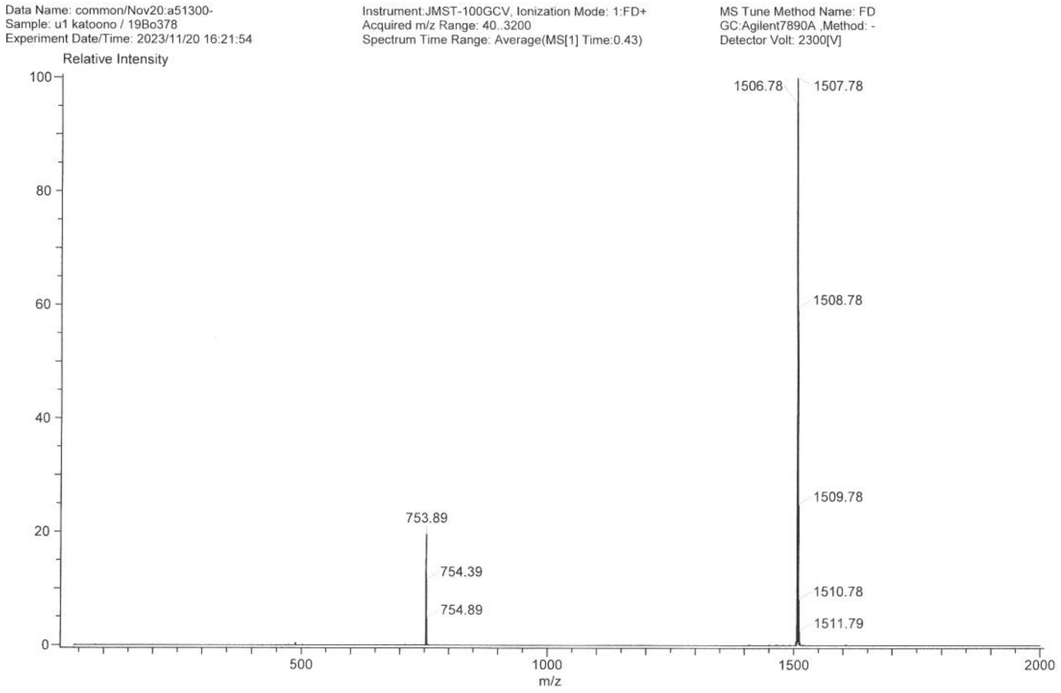

LRMS (FD) of **19B**.

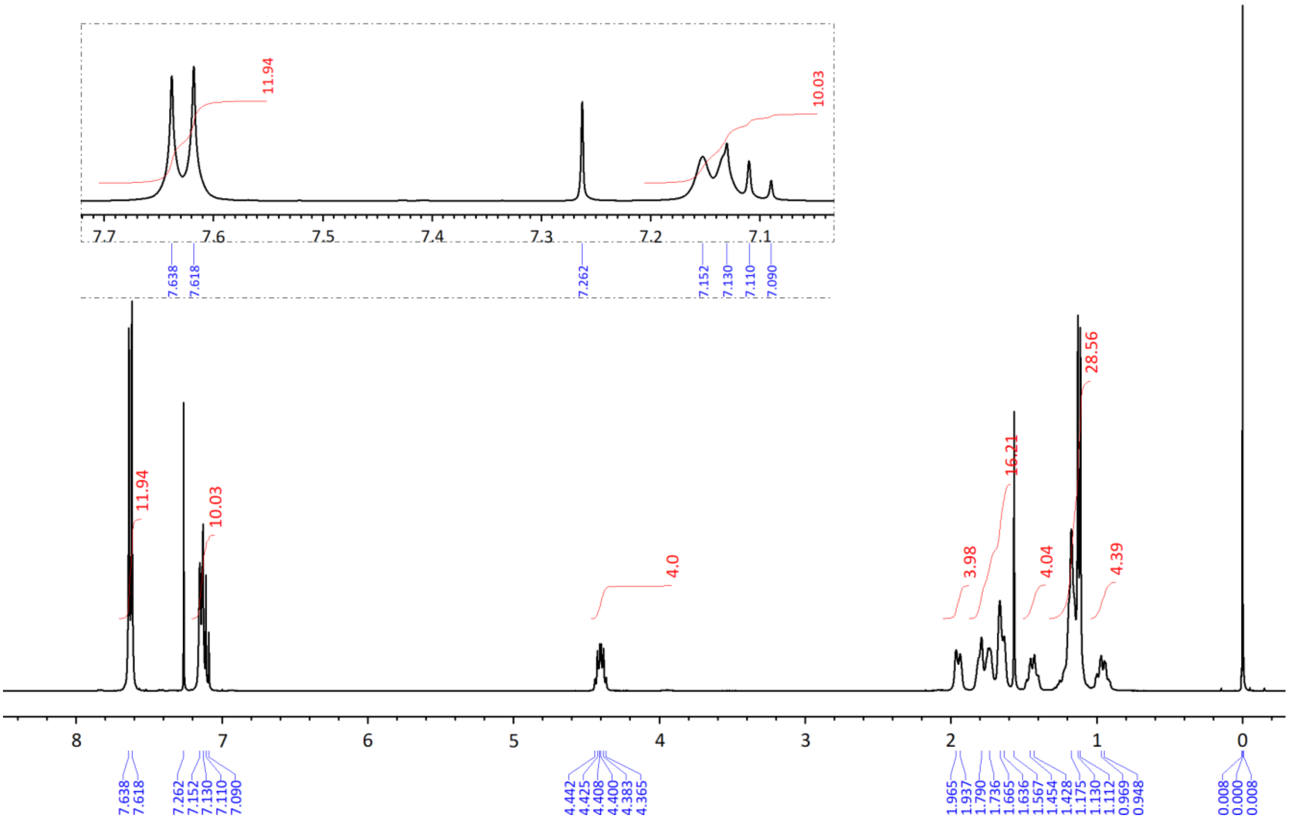

^{1}H NMR spectrum (400 MHz) of **20**, measured in $CDCl_3$ at room temperature.

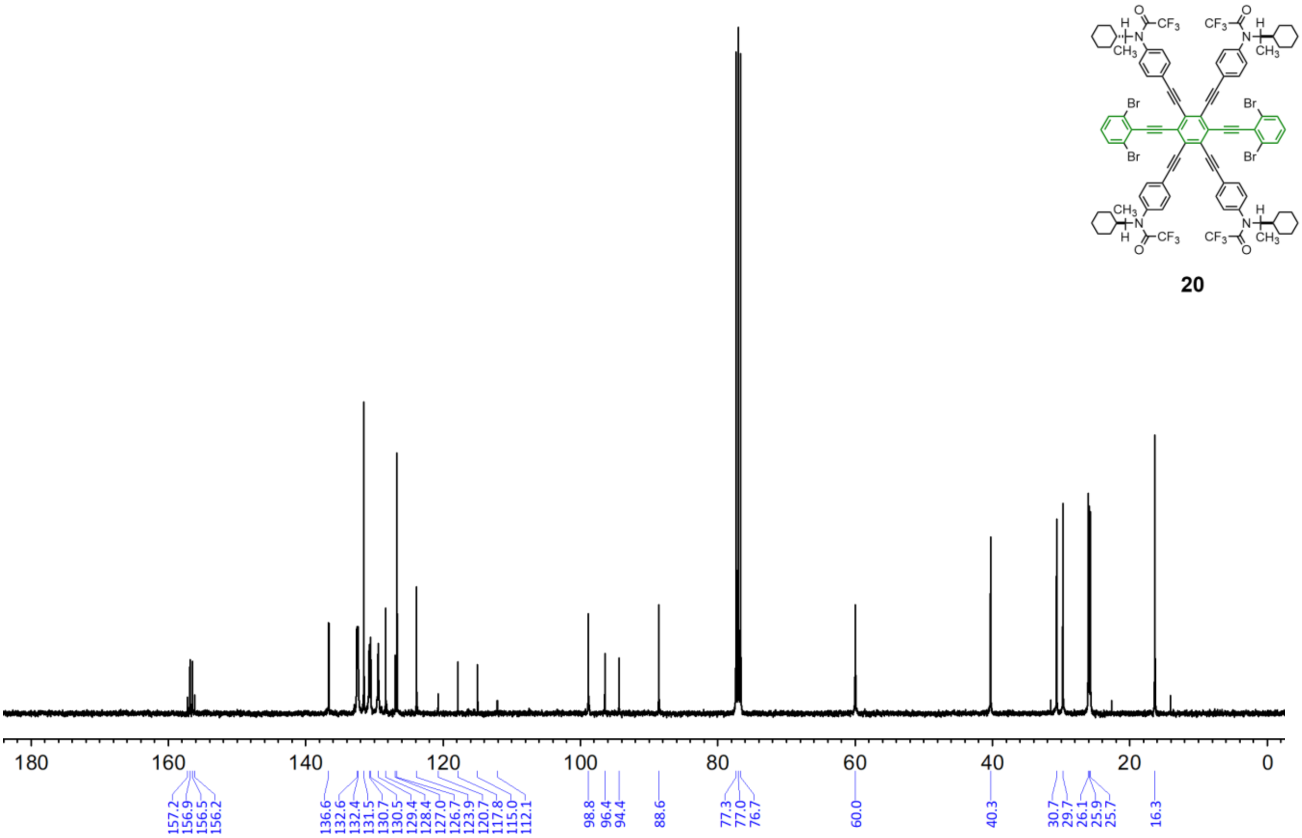

^{13}C NMR spectrum (100 MHz) of **20**, measured in $CDCl_3$ at room temperature.

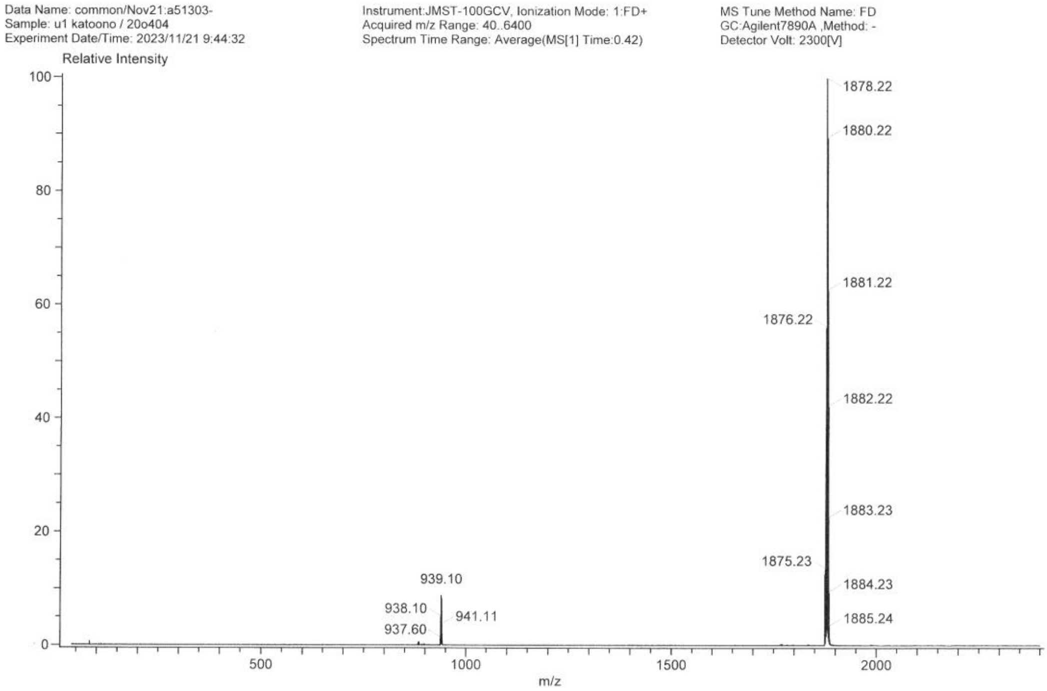
Data Name: common/Nov21:a51303-
Sample: u1 katoono / 20o404
Experiment Date/Time: 2023/11/21 9:44:32
Instrument:JMST-100GCV, Ionization Mode: 1:FD+
Acquired m/z Range: 40..6400
Spectrum Time Range: Average(MS[1] Time:0.42)
MS Tune Method Name: FD
GC:Agilent7890A ,Method: -
Detector Volt: 2300[V]
Relative Intensity
1878.22
1880.22
1881.22
1876.22
1882.22
1883.23
1875.23
1884.23
1885.24
939.10
938.10
941.11
937.60
m/z

LRMS (FD) of **20**.

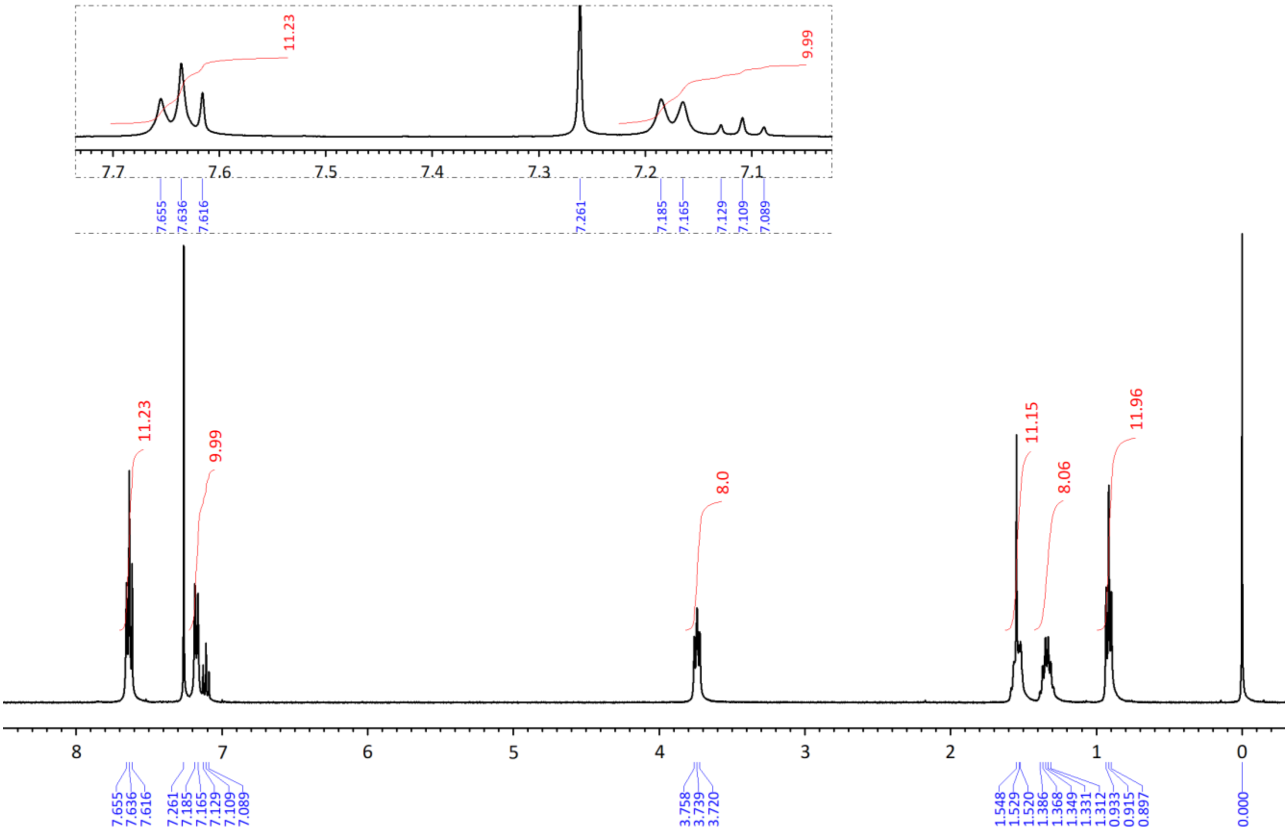

^{1}H NMR spectrum (400 MHz) of **20B**, measured in $CDCl_3$ at room temperature.

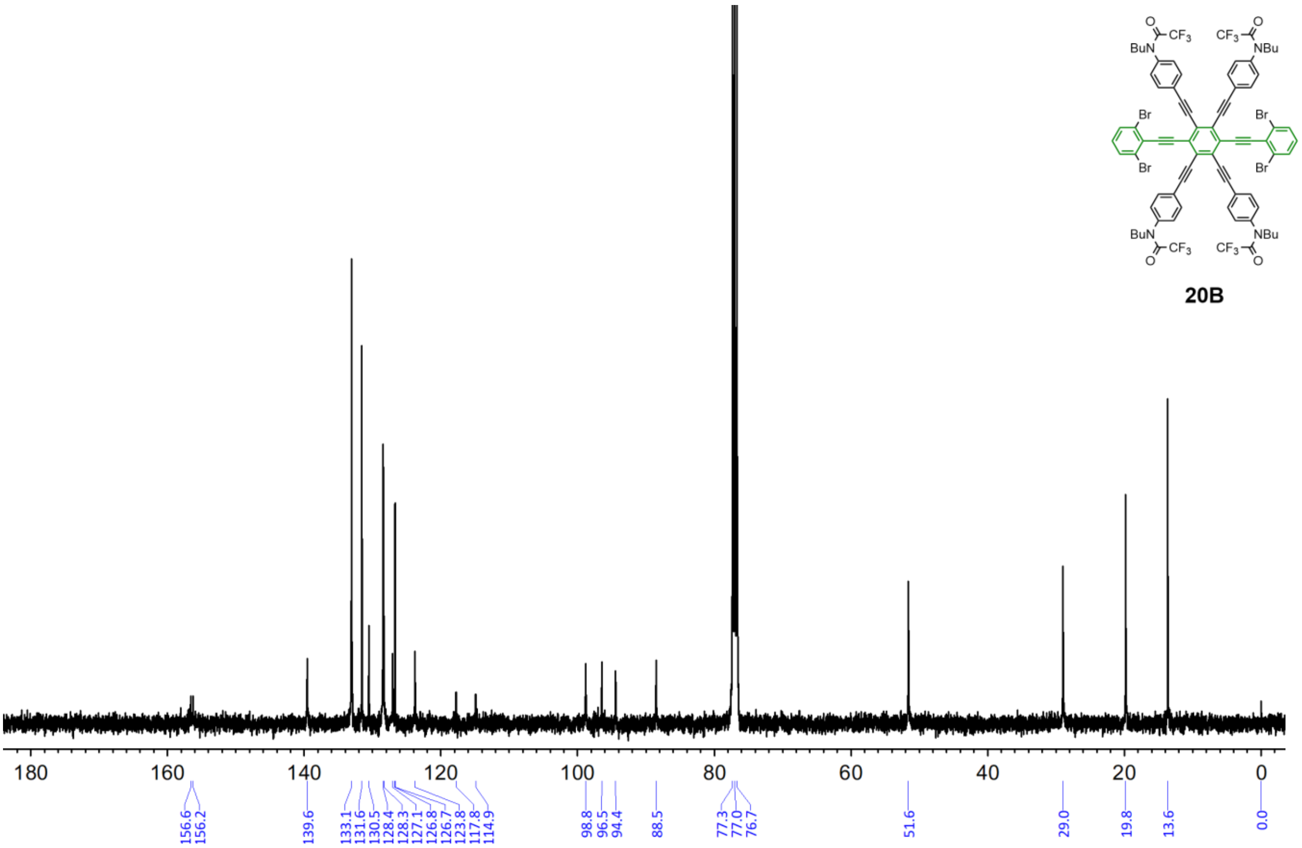

^{13}C NMR spectrum (100 MHz) of **20B**, measured in $CDCl_3$ at room temperature.

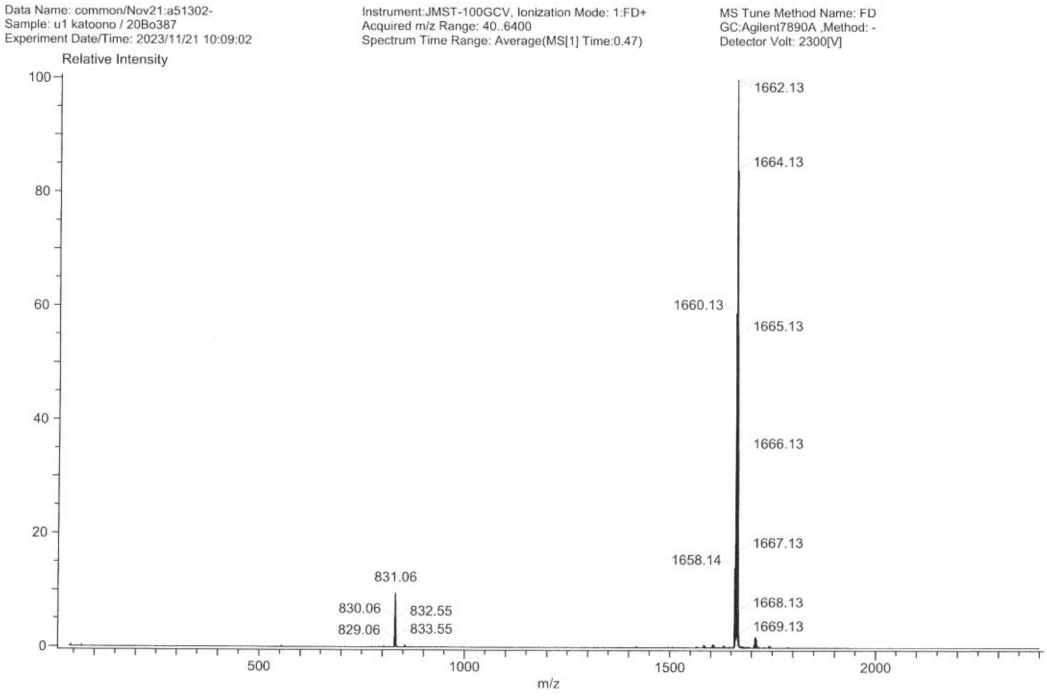

LRMS (FD) of **20B**.

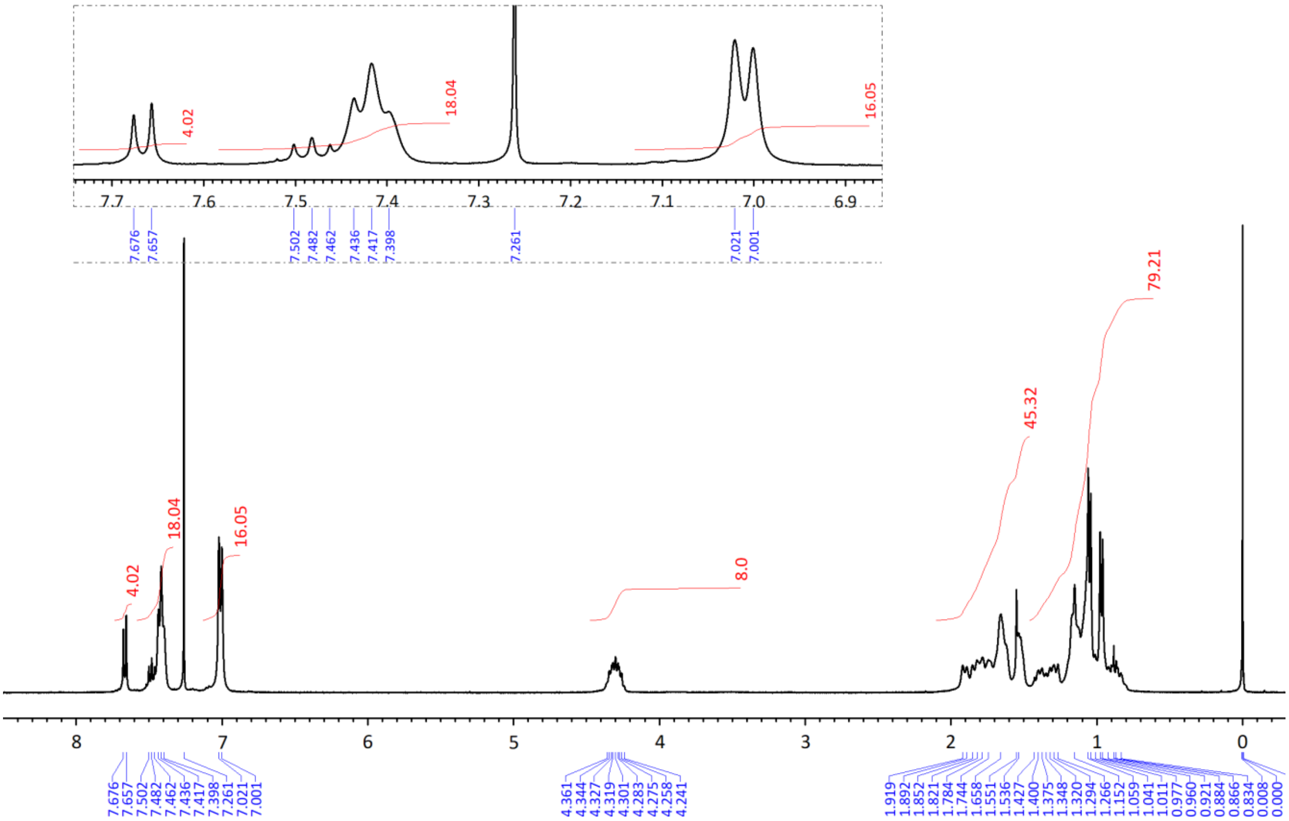

^{1}H NMR spectrum (400 MHz) of pr-**5**, measured in $CDCl_3$ at room temperature.

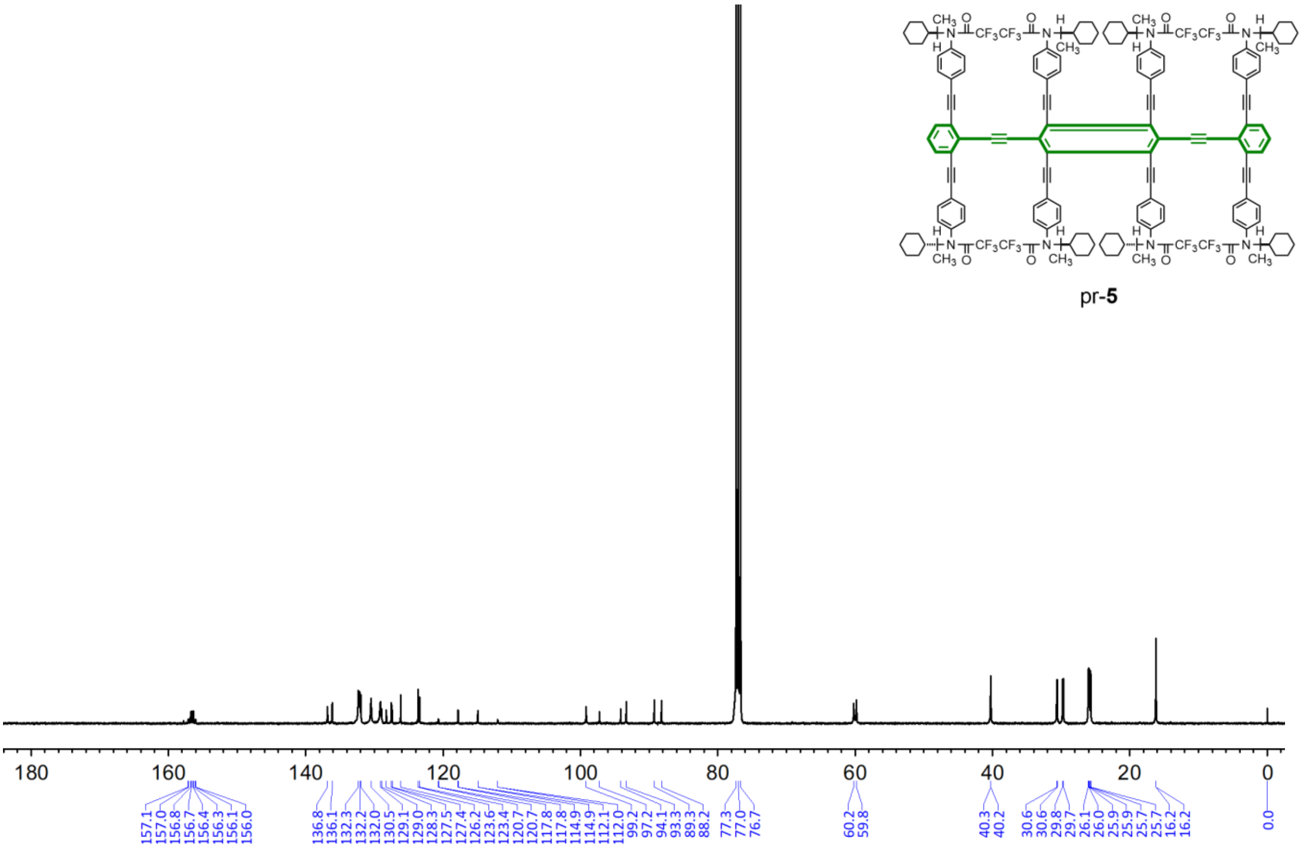

^{13}C NMR spectrum (100 MHz) of pr-**5**, measured in $CDCl_3$ at room temperature.

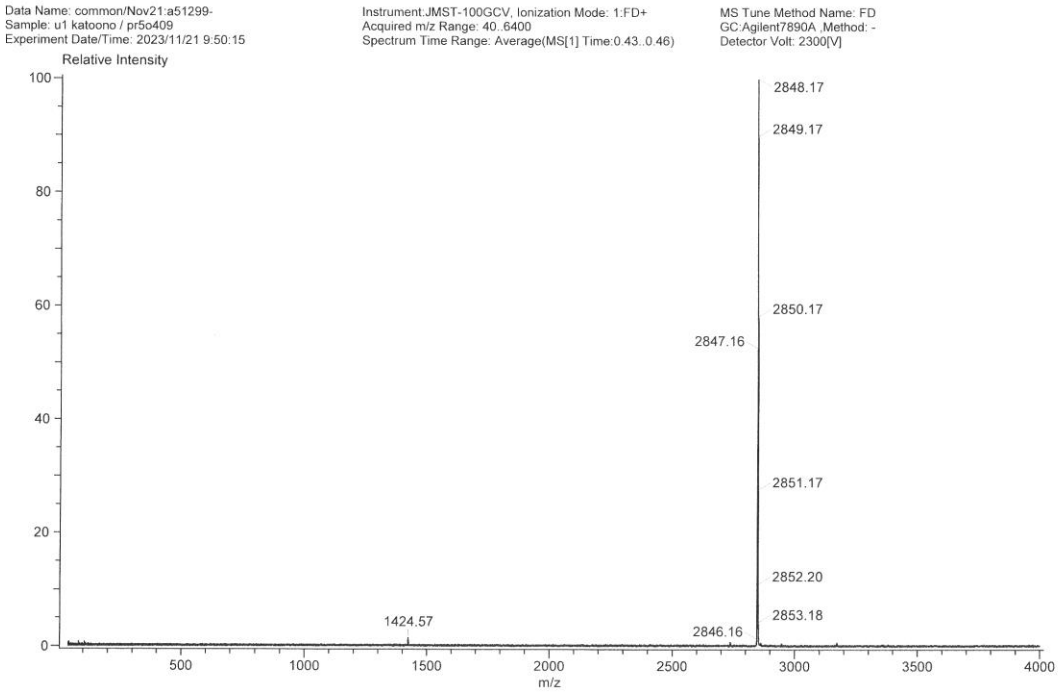

LRMS (FD) of pr-**5**.

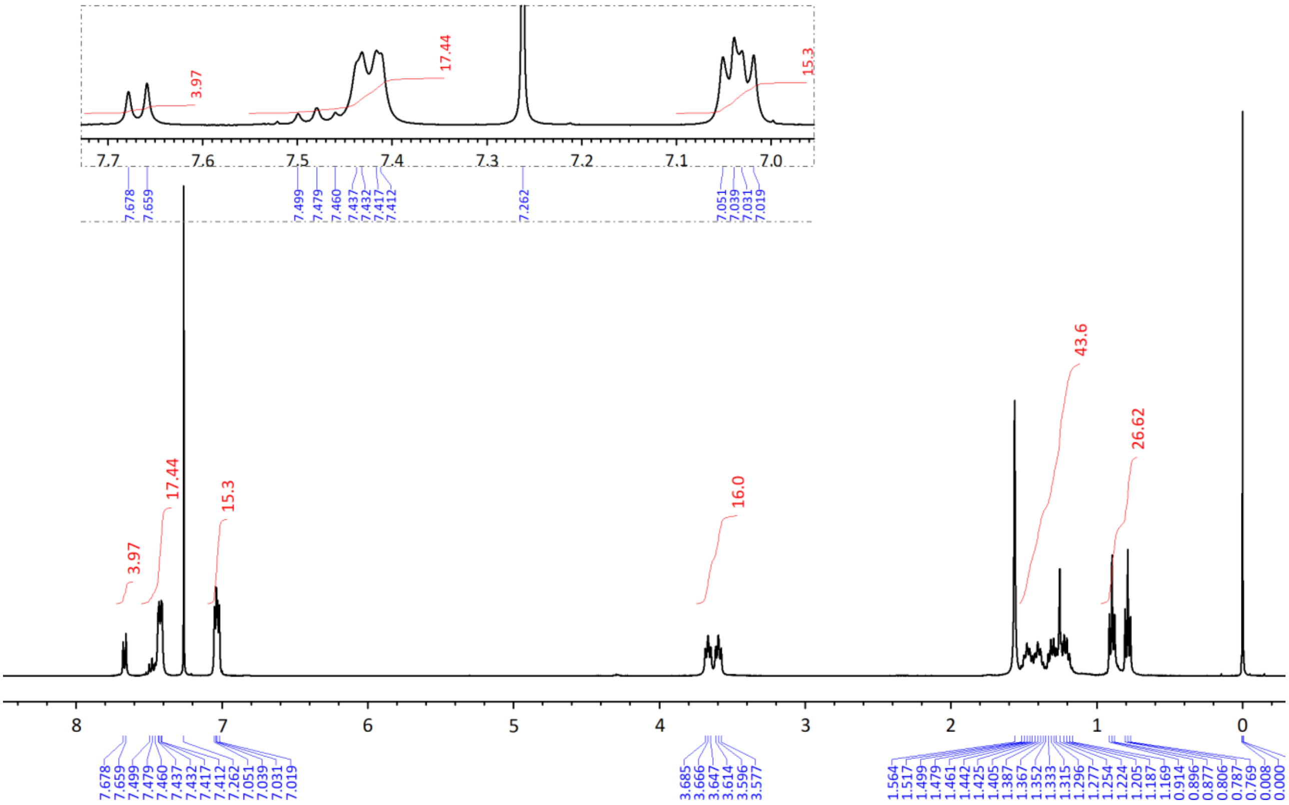

^{1}H NMR spectrum (400 MHz) of pr-**5B**, measured in $CDCl_3$ at room temperature.

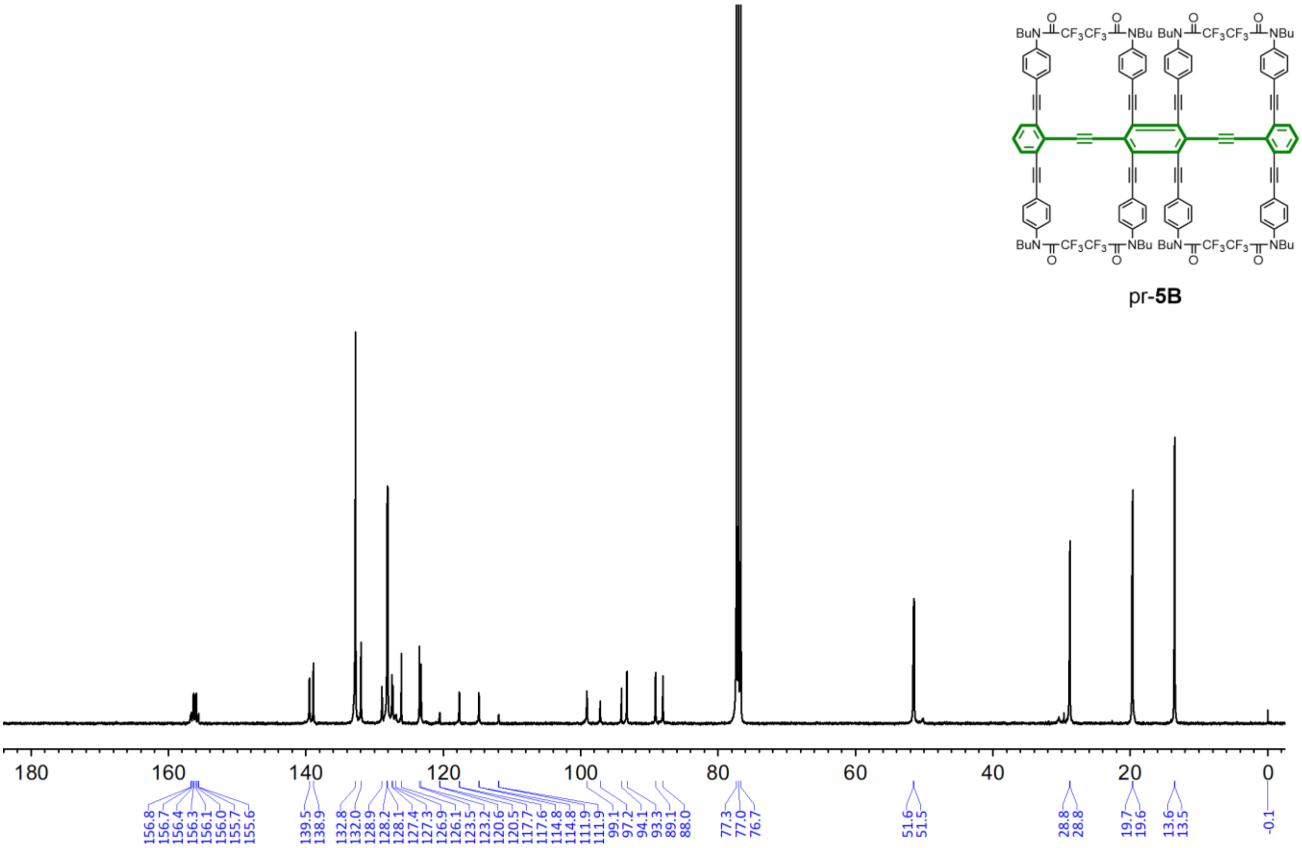

^{13}C NMR spectrum (100 MHz) of pr-**5B**, measured in $CDCl_3$ at room temperature.

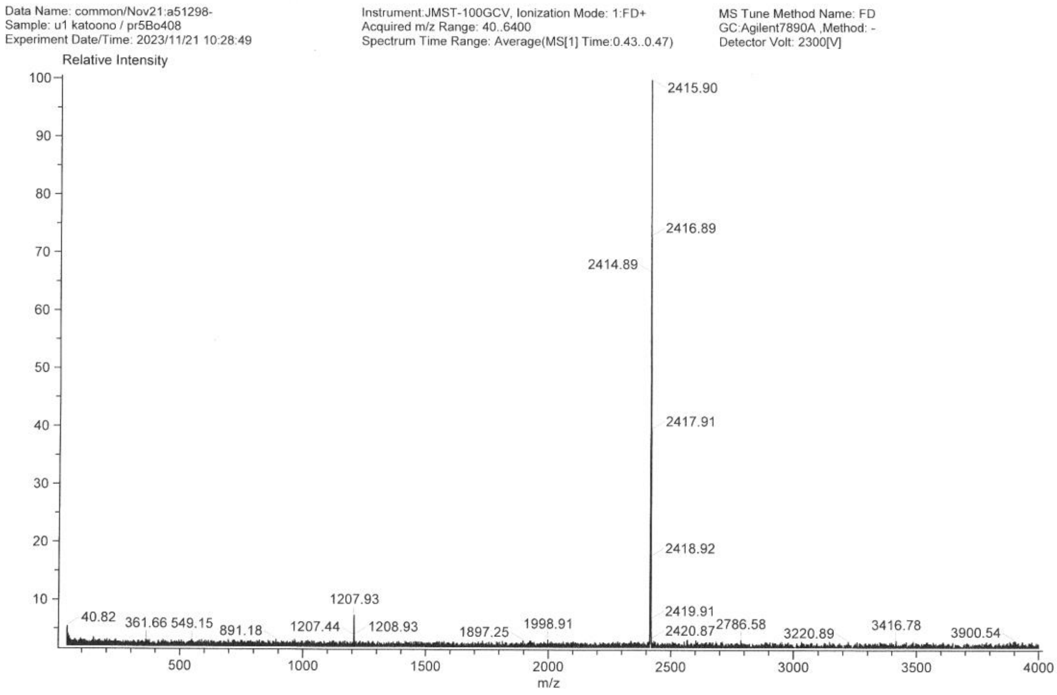

LRMS (FD) of pr-**5B**.

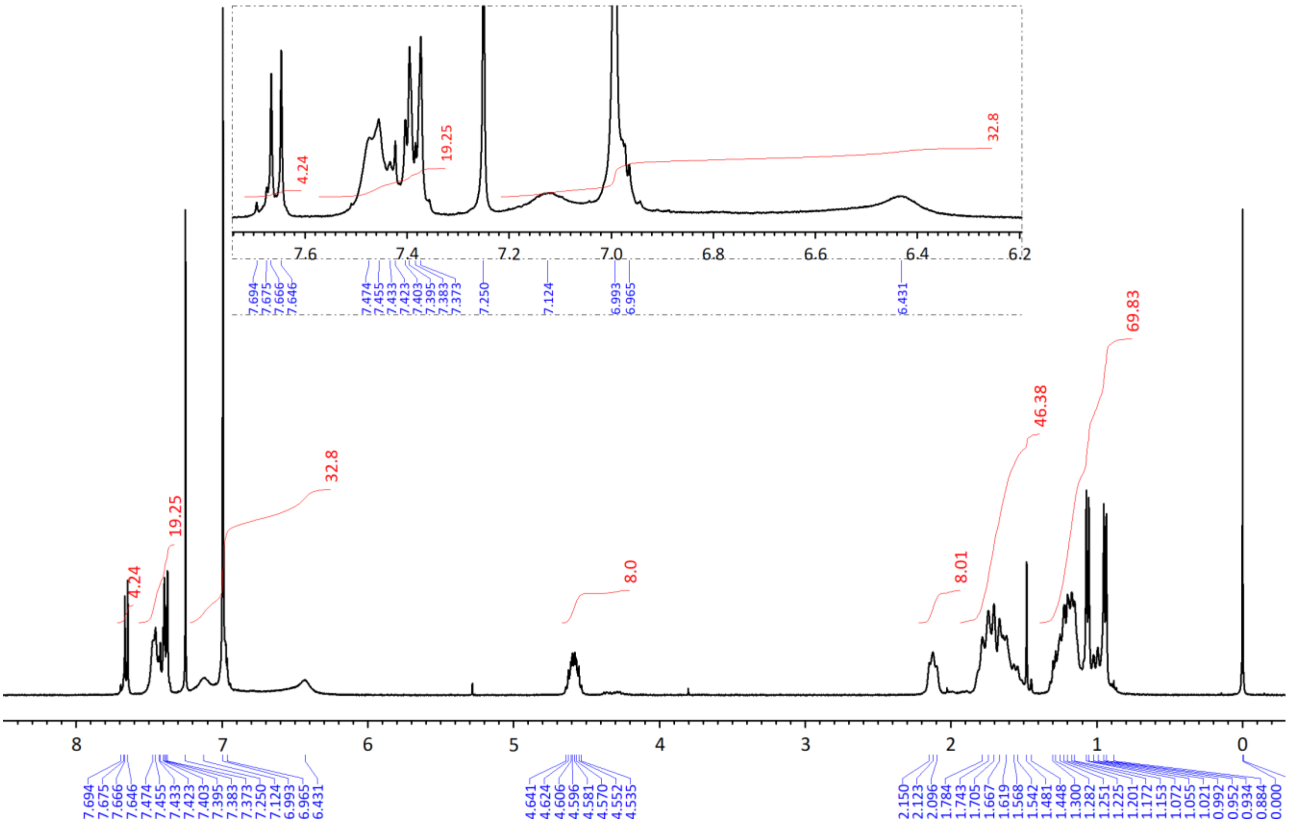

^{1}H NMR spectrum (400 MHz) of **5**, measured in $CDCl_3$ at 323 K.

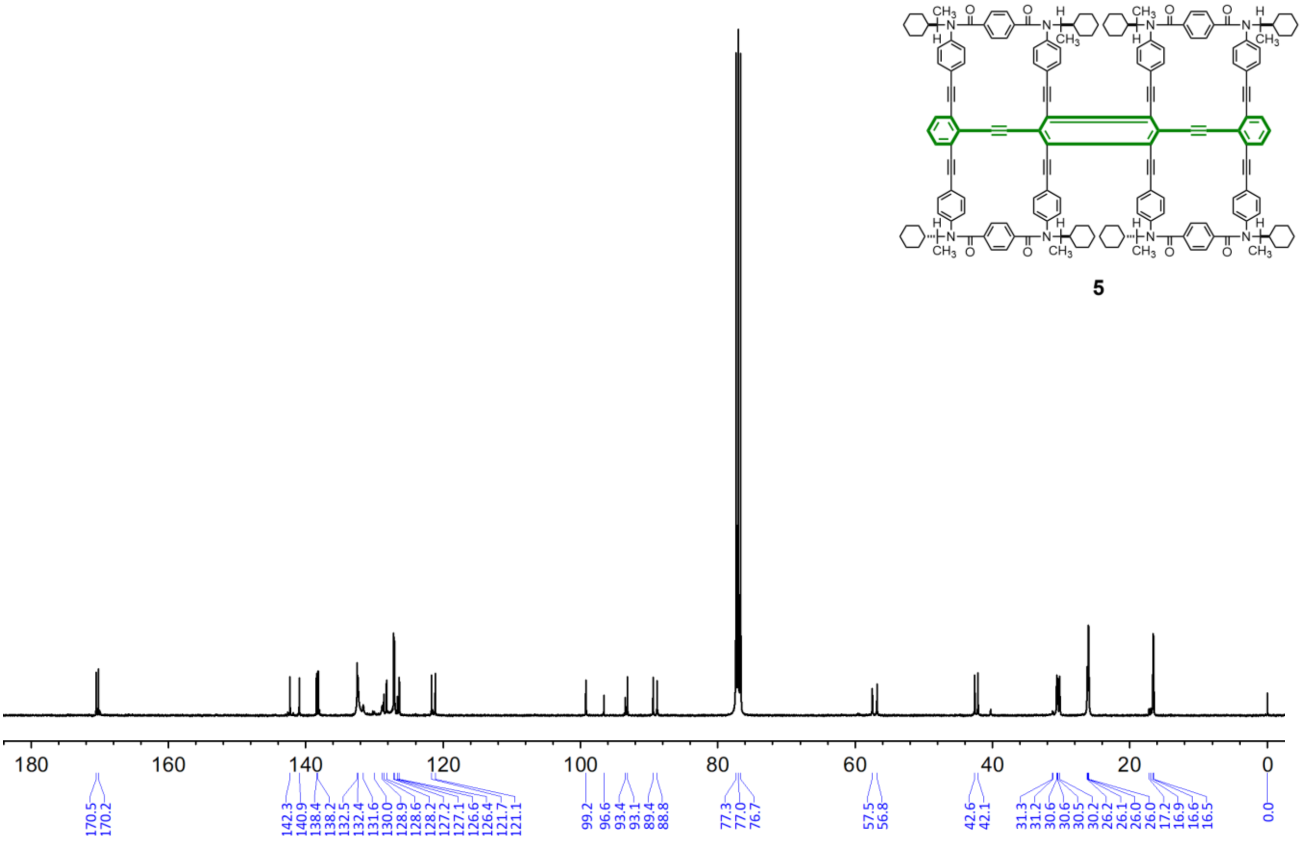

^{13}C NMR spectrum (100 MHz) of **5**, measured in $CDCl_3$ at 313 K.

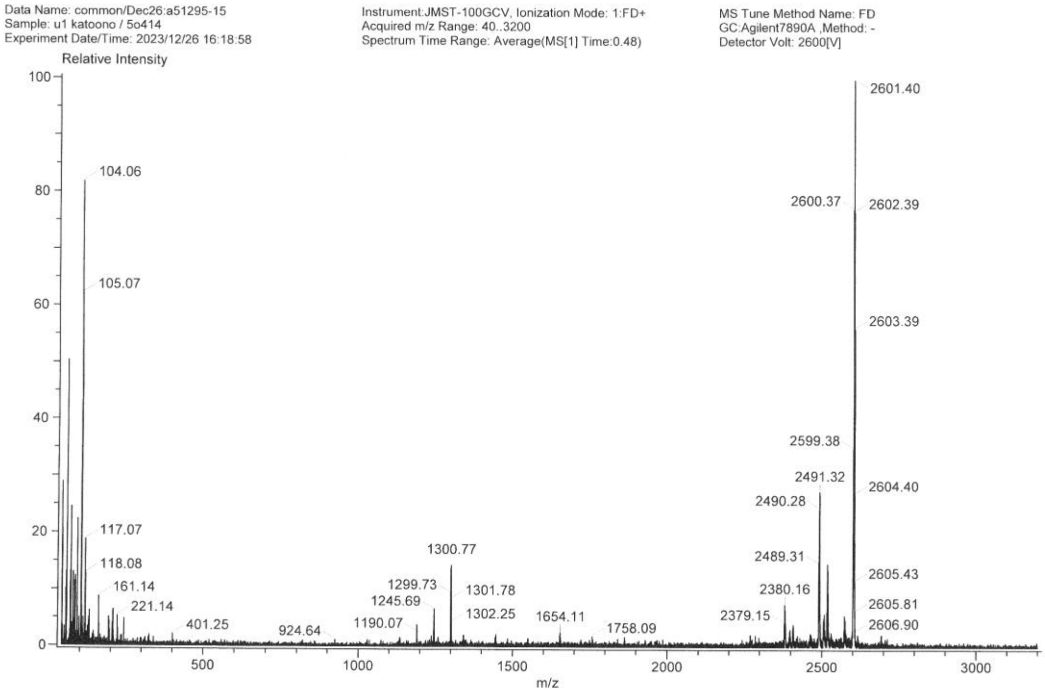
Data Name: common/Dec26:a51295-15
Sample: u1 katoono / 5o414
Experiment Date/Time: 2023/12/26 16:18:58
Instrument:JMST-100GCV, Ionization Mode: 1:FD+
Acquired m/z Range: 40..3200
Spectrum Time Range: Average(MS[1] Time:0.48)
MS Tune Method Name: FD
GC:Agilent7890A ,Method: -
Detector Volt: 2600[V]
Relative Intensity
m/z
2601.40
2600.37
2602.39
2603.39
2599.38
2491.32
2490.28
2604.40
2489.31
2605.43
2380.16
2605.81
2379.15
2606.90
1300.77
1299.73
1301.78
1245.69
1302.25
1190.07
1654.11
1758.09
924.64
401.25
104.06
105.07
117.07
118.08
161.14
221.14

LRMS (FD) of **5**.

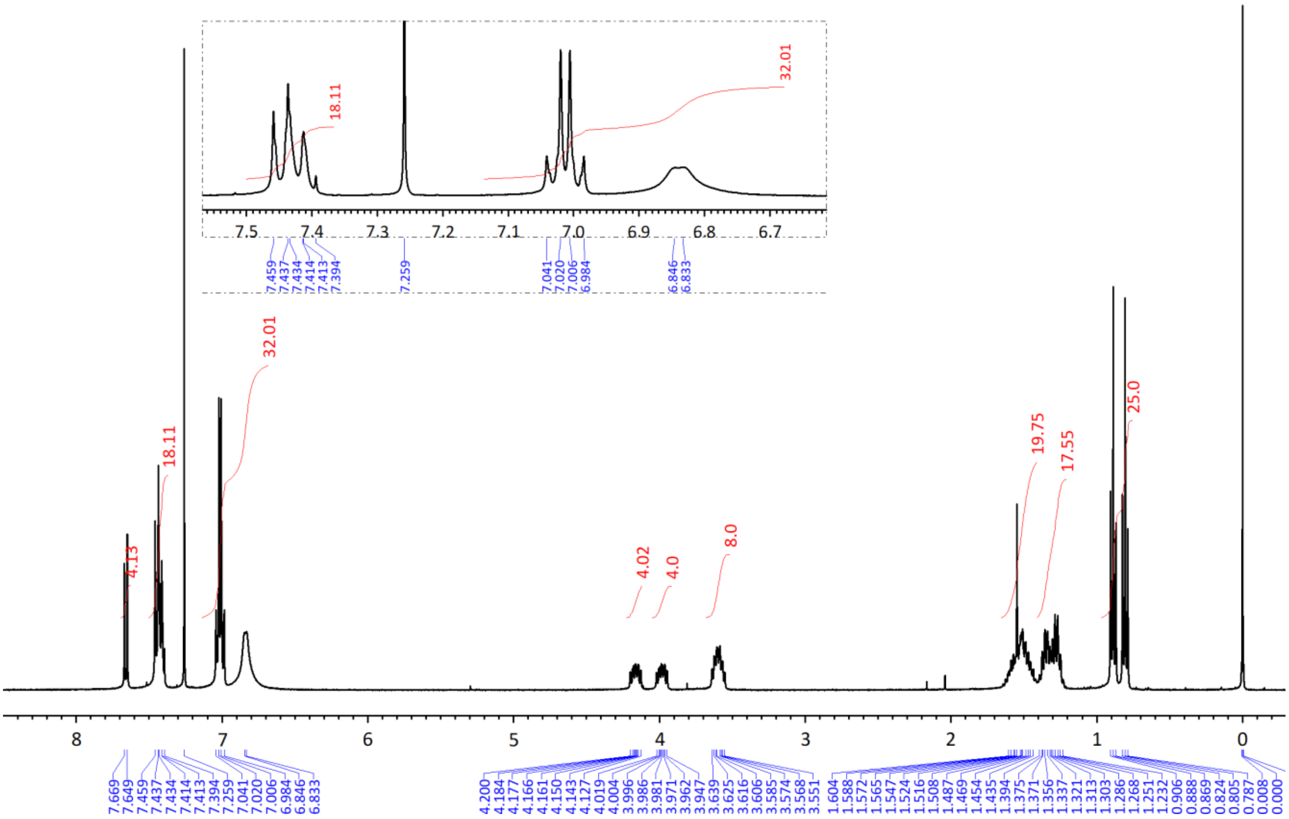

^{1}H NMR spectrum (400 MHz) of **5B**, measured in $CDCl_3$ at 303 K.

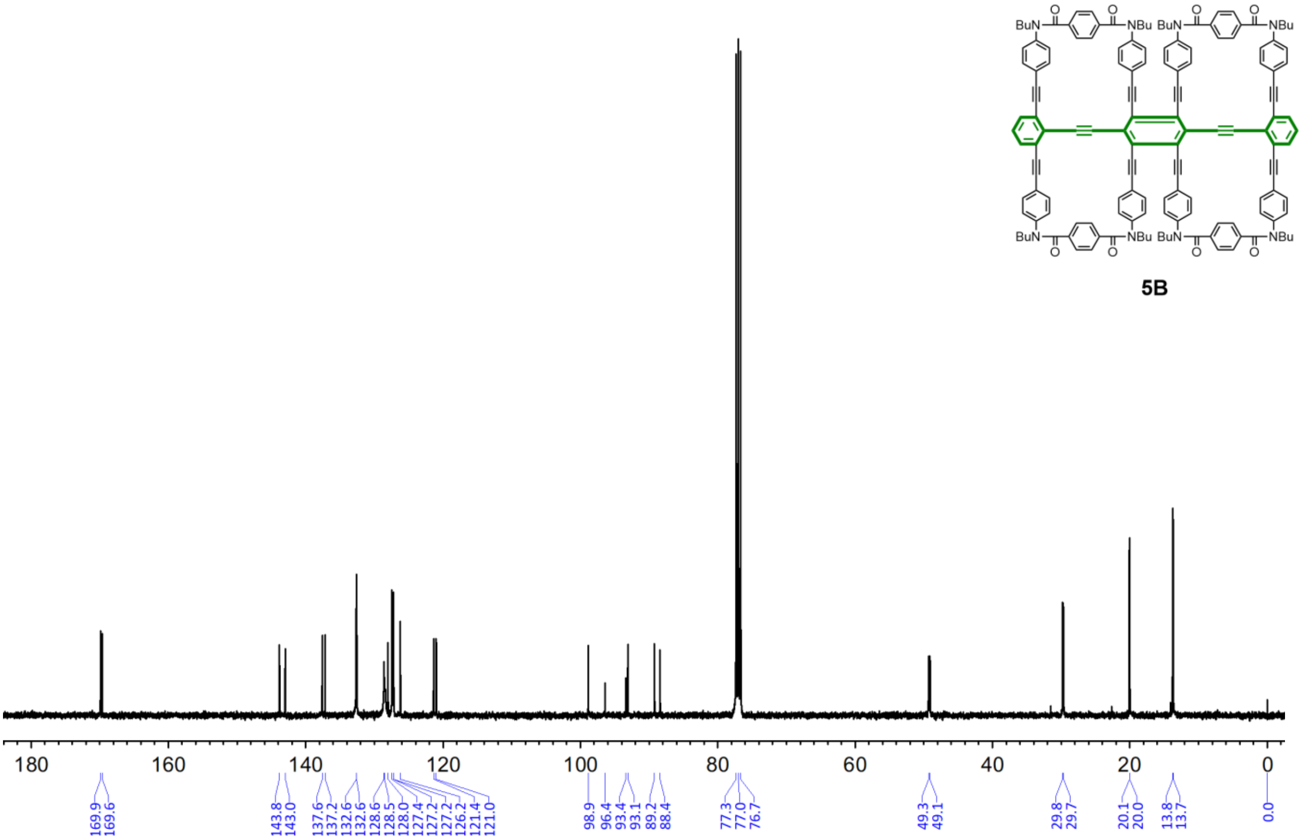

^{13}C NMR spectrum (100 MHz) of **5B**, measured in $CDCl_3$ at room temperature.

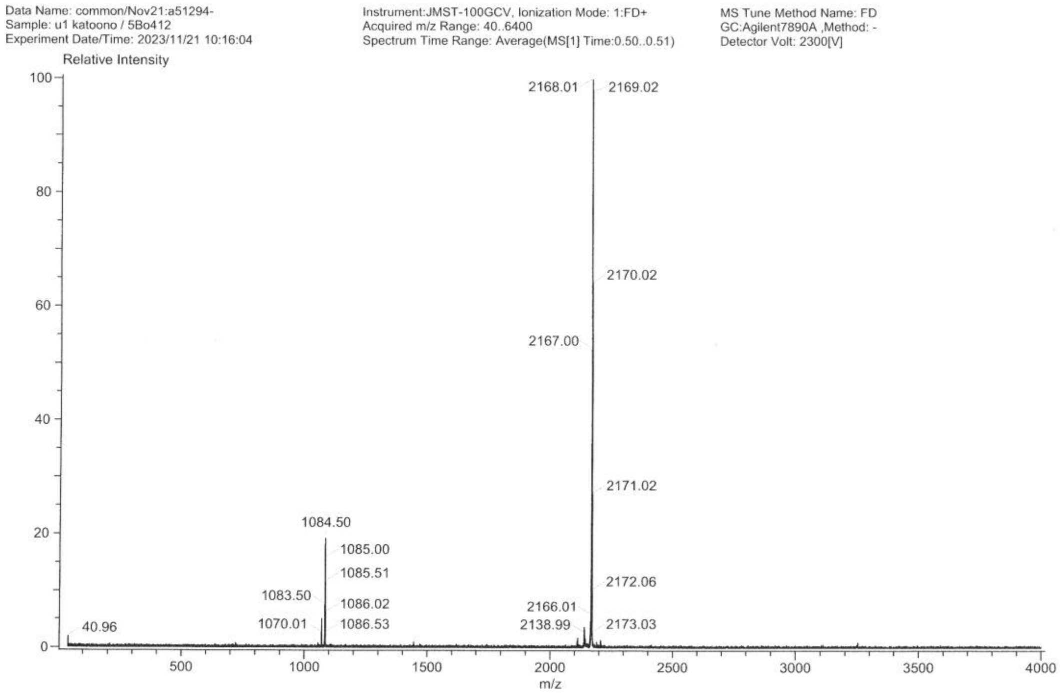

Data Name: common/Nov21:a51294-
Sample: u1 katoono / 5Bo412
Experiment Date/Time: 2023/11/21 10:16:04
Instrument:JMST-100GCV, Ionization Mode: 1:FD+
Acquired m/z Range: 40..6400
Spectrum Time Range: Average(MS[1] Time:0.50..0.51)
MS Tune Method Name: FD
GC:Agilent7890A ,Method: -
Detector Volt: 2300[V]
Relative Intensity
2168.01
2169.02
2170.02
2167.00
2171.02
2172.06
2166.01
2138.99
2173.03
1084.50
1085.00
1085.51
1083.50
1086.02
1070.01
1086.53
40.96
m/z

LRMS (FD) of **5B**.

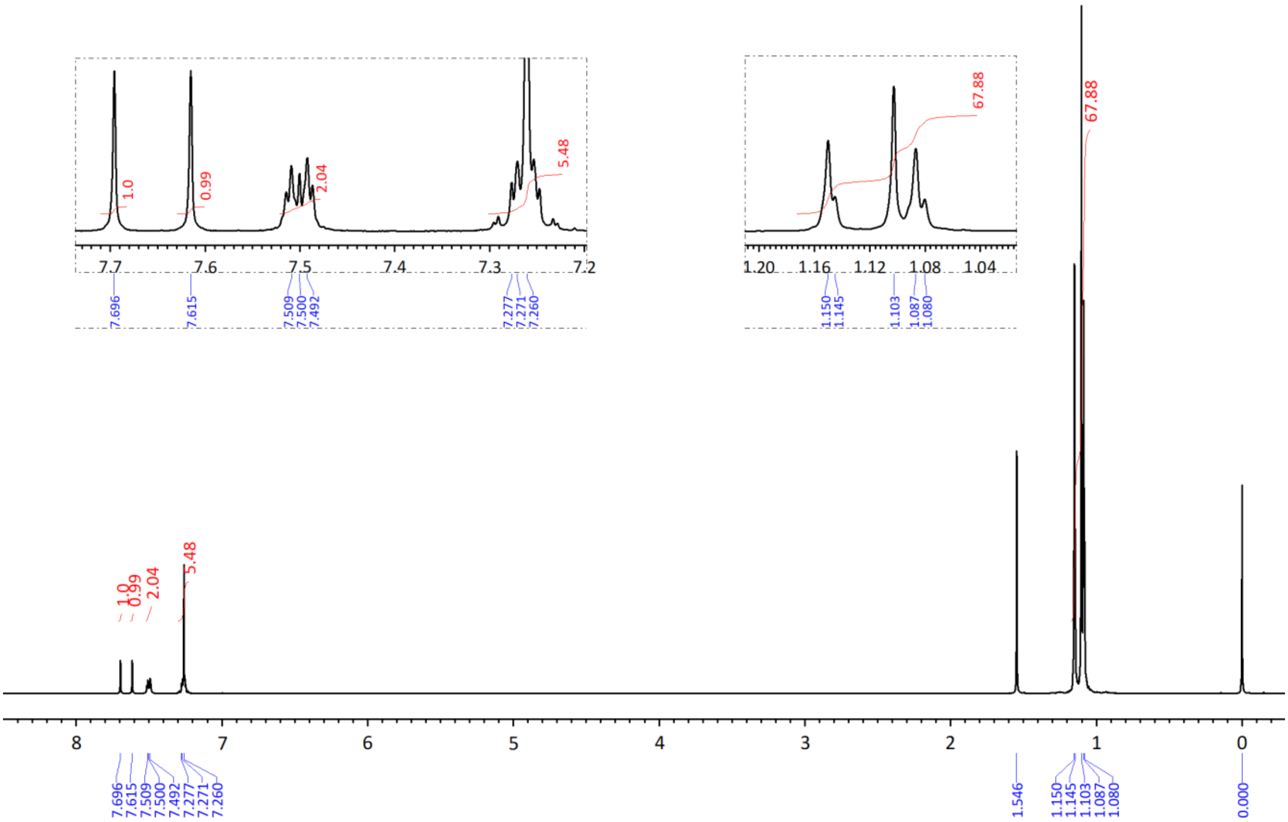

^{1}H NMR spectrum (400 MHz) of **26**, measured in $CDCl_3$ at room temperature.

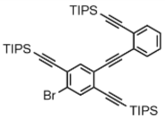

26

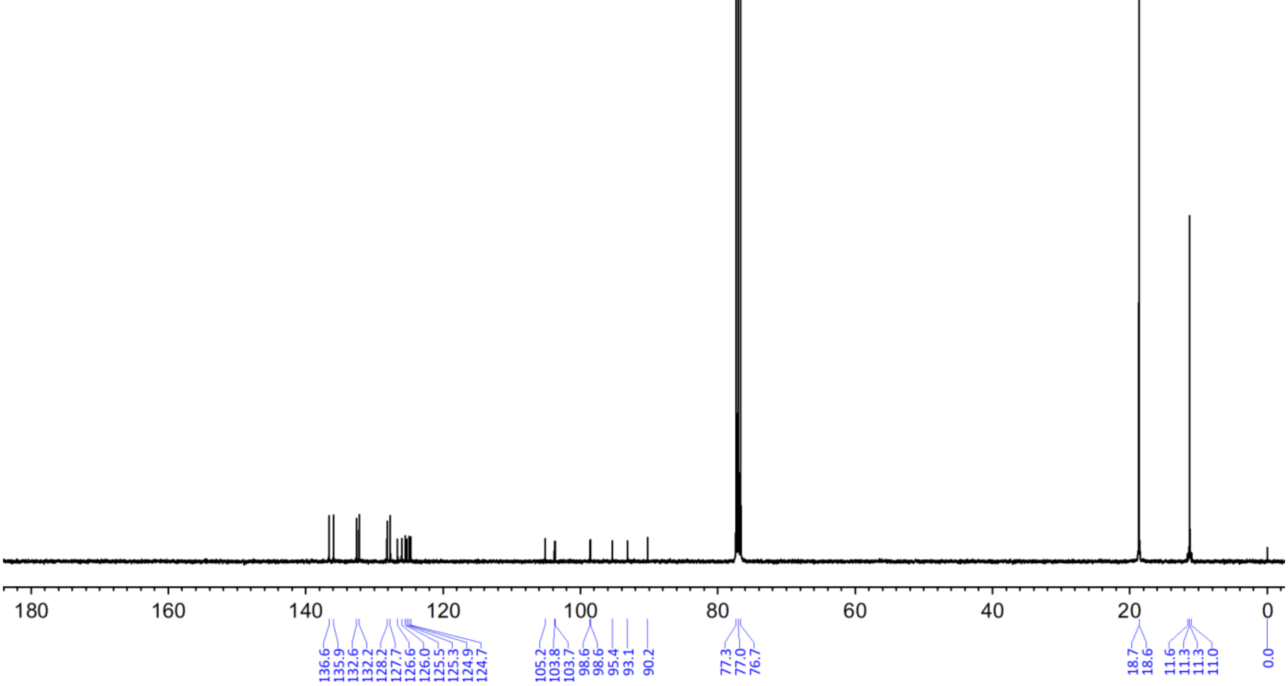

^{13}C NMR spectrum (100 MHz) of **26**, measured in $CDCl_3$ at room temperature.

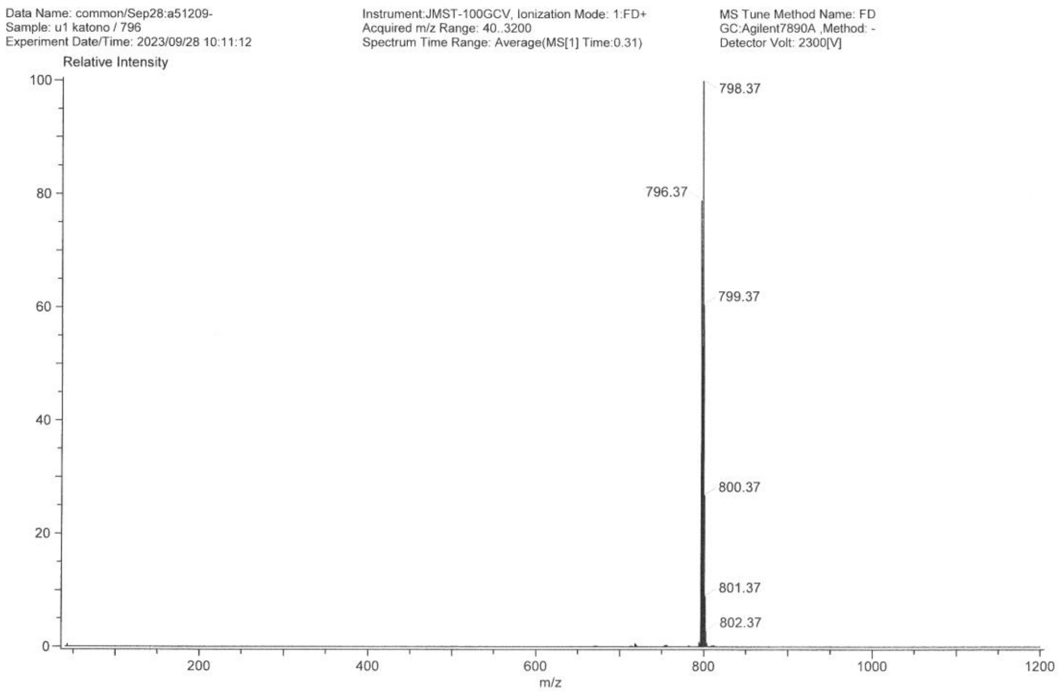
Data Name: common/Sep28:a51209-
Sample: u1 katono / 796
Experiment Date/Time: 2023/09/28 10:11:12
Instrument:JMST-100GCV, Ionization Mode: 1:FD+
Acquired m/z Range: 40..3200
Spectrum Time Range: Average(MS[1] Time:0.31)
MS Tune Method Name: FD
GC:Agilent7890A ,Method: -
Detector Volt: 2300[V]
Relative Intensity
798.37
796.37
799.37
800.37
801.37
802.37
m/z

LRMS (FD) of **26**.

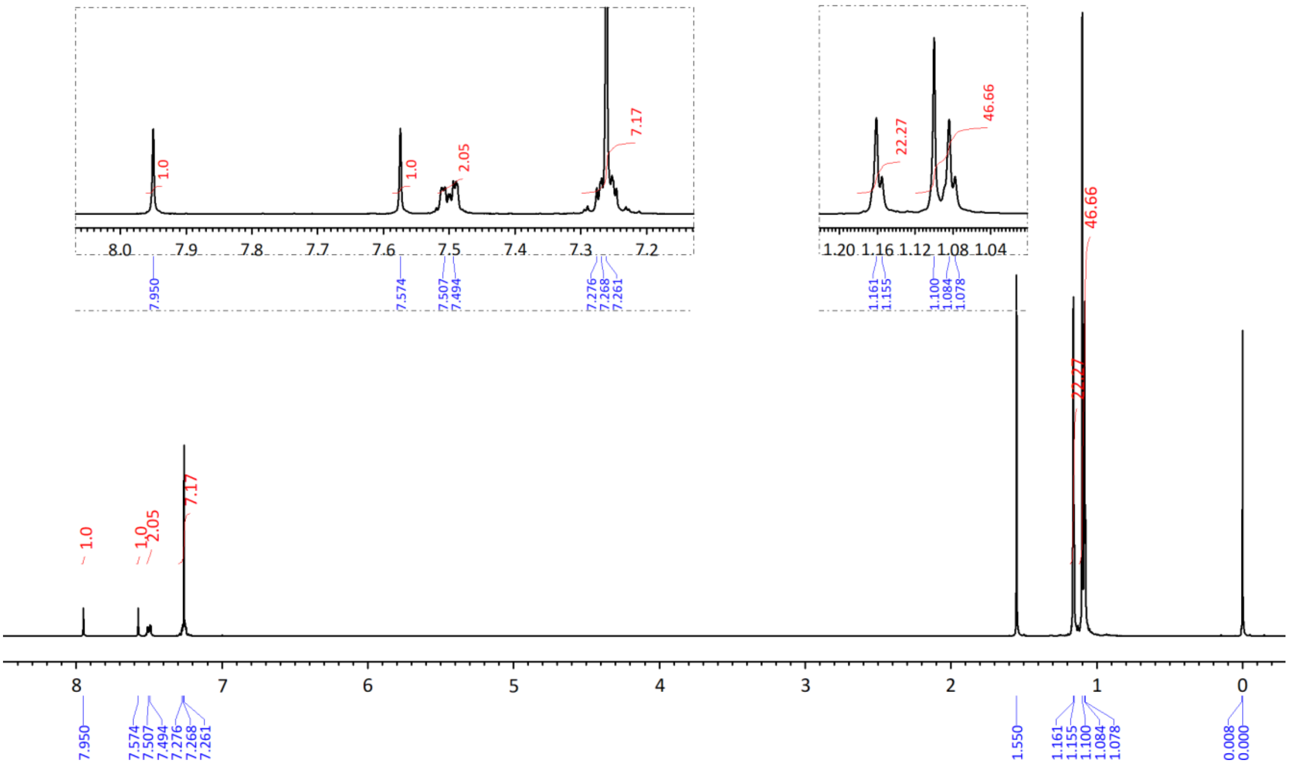

^{1}H NMR spectrum (400 MHz) of **27**, measured in $CDCl_3$ at room temperature.

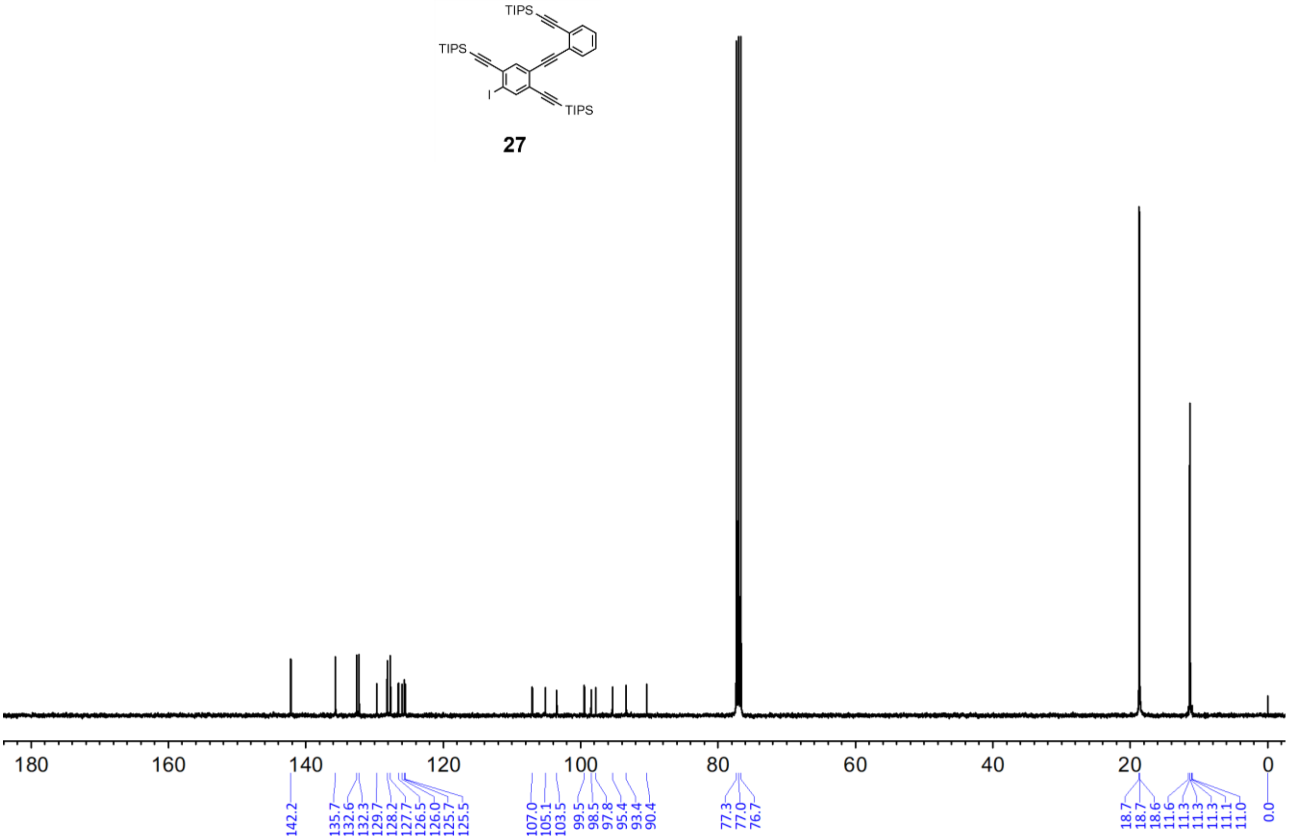

^{13}C NMR spectrum (100 MHz) of **27**, measured in $CDCl_3$ at room temperature.

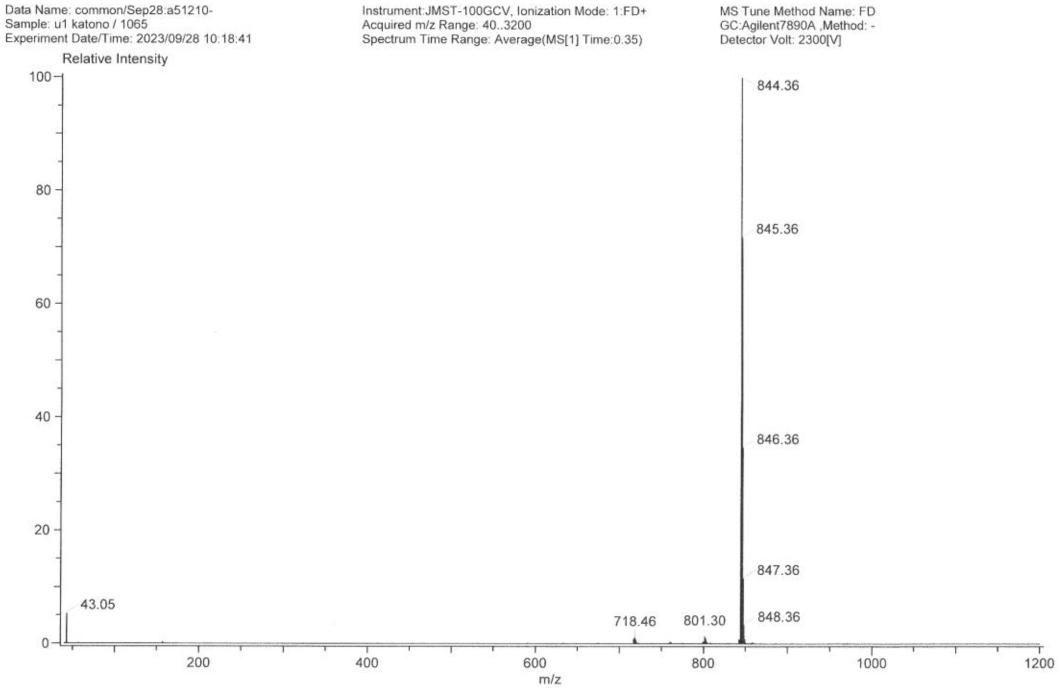
Data Name: common/Sep28:a51210-
Sample: u1 katono / 1065
Experiment Date/Time: 2023/09/28 10:18:41
Instrument:JMST-100GCV, Ionization Mode: 1:FD+
Acquired m/z Range: 40..3200
Spectrum Time Range: Average(MS[1] Time:0.35)
MS Tune Method Name: FD
GC:Agilent7890A ,Method: -
Detector Volt: 2300[V]
Relative Intensity
100
80
60
40
20
0
844.36
845.36
846.36
847.36
848.36
43.05
718.46
801.30
200
400
600
800
1000
1200
m/z

LRMS (FD) of **27**.

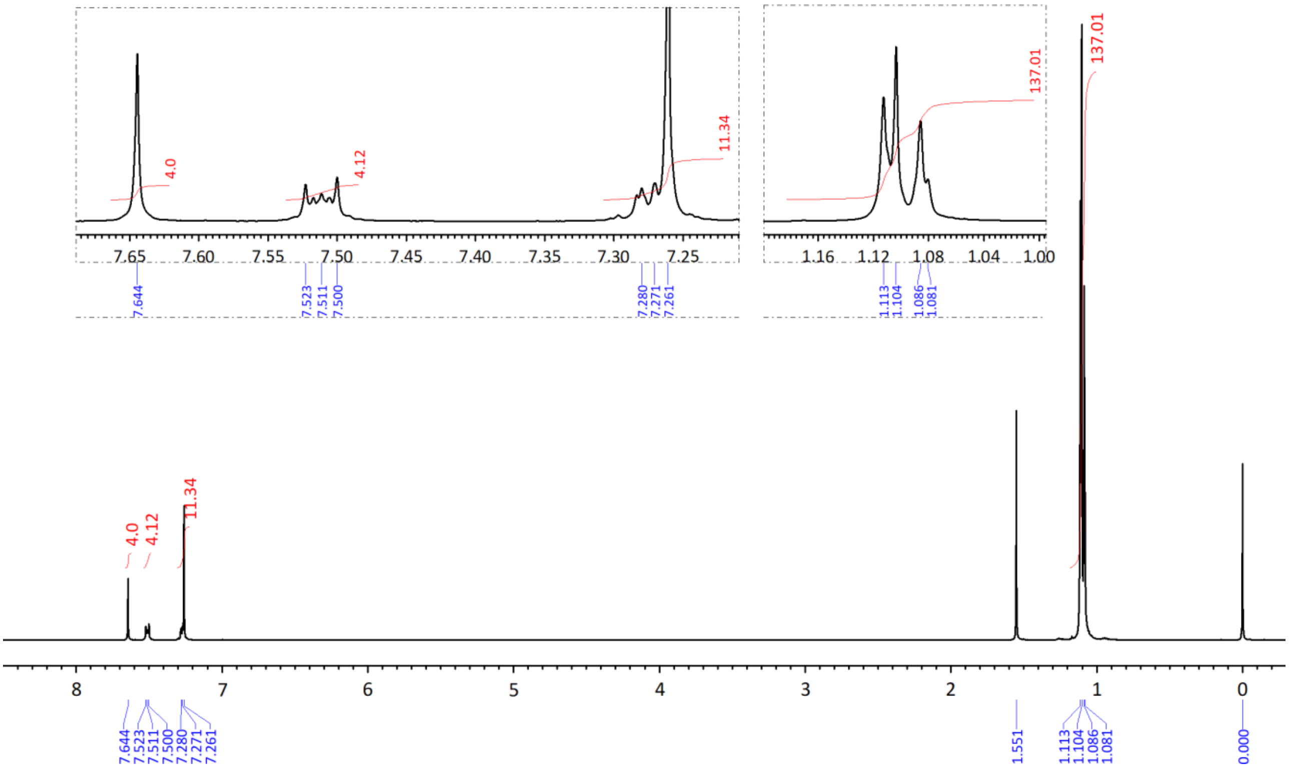

^{1}H NMR spectrum (400 MHz) of **28**, measured in $CDCl_3$ at room temperature.

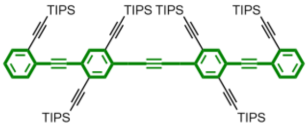

28

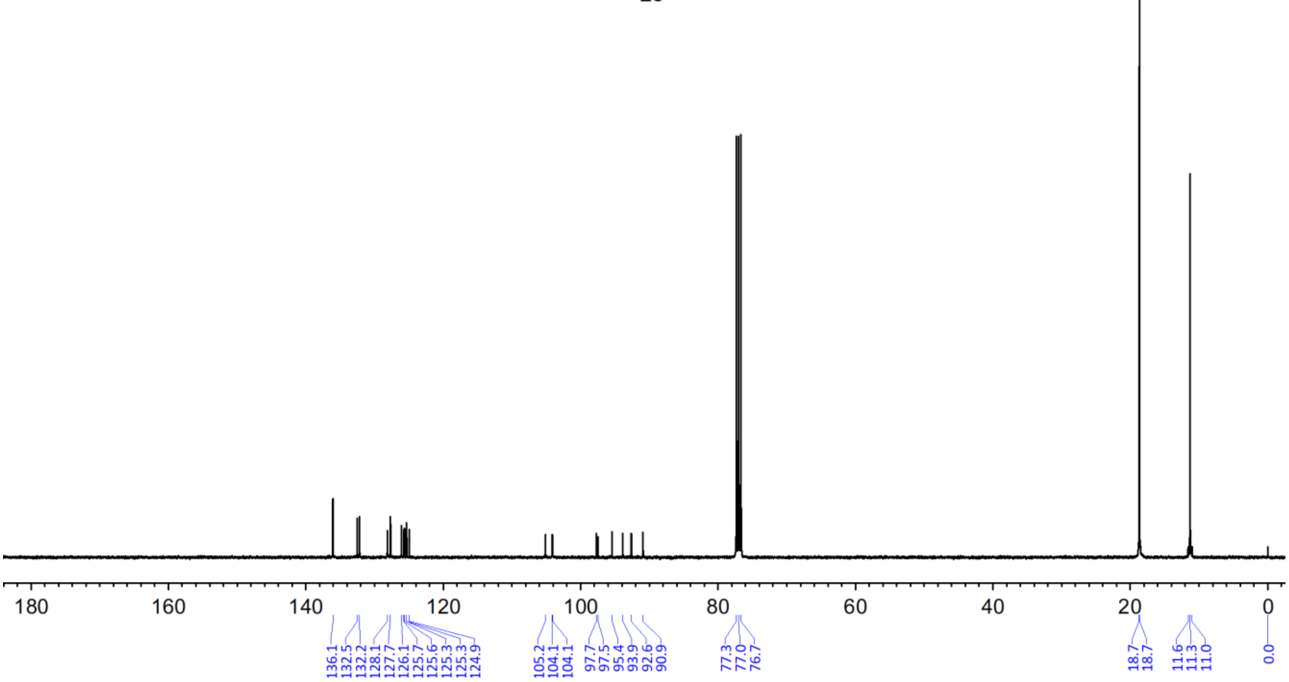

^{13}C NMR spectrum (100 MHz) of **28**, measured in $CDCl_3$ at room temperature.

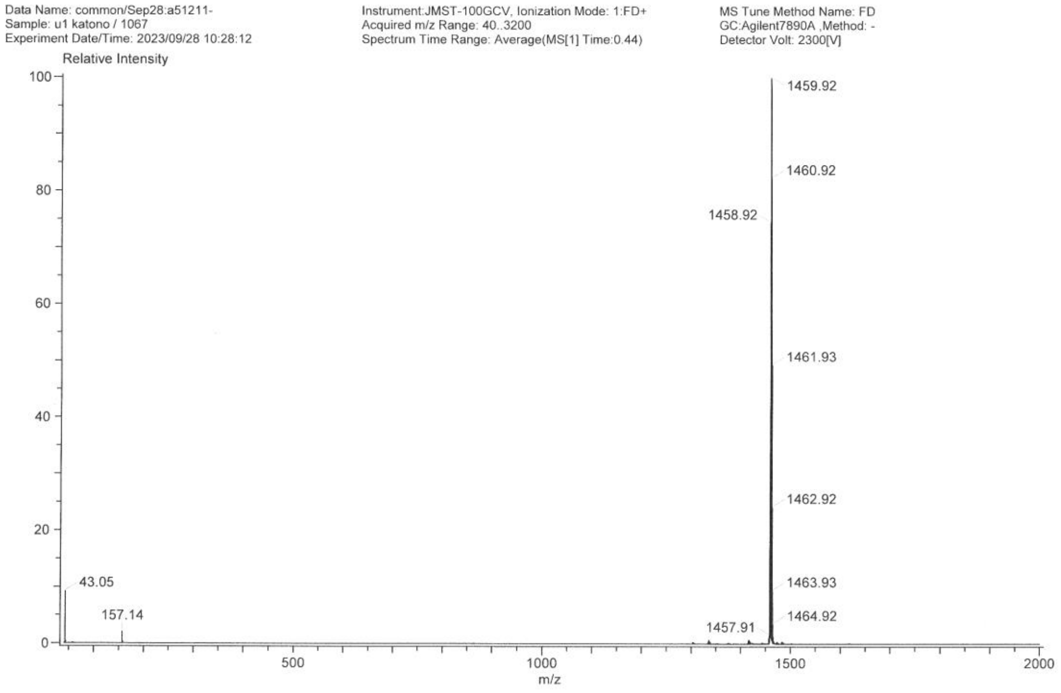
Data Name: common/Sep28:a51211-
Sample: u1 katono / 1067
Experiment Date/Time: 2023/09/28 10:28:12
Instrument:JMST-100GCV, Ionization Mode: 1:FD+
Acquired m/z Range: 40..3200
Spectrum Time Range: Average(MS[1] Time:0.44)
MS Tune Method Name: FD
GC:Agilent7890A ,Method: -
Detector Volt: 2300[V]
Relative Intensity
1459.92
1460.92
1458.92
1461.93
1462.92
1463.93
1464.92
1457.91
43.05
157.14
m/z

LRMS (FD) of **28**.

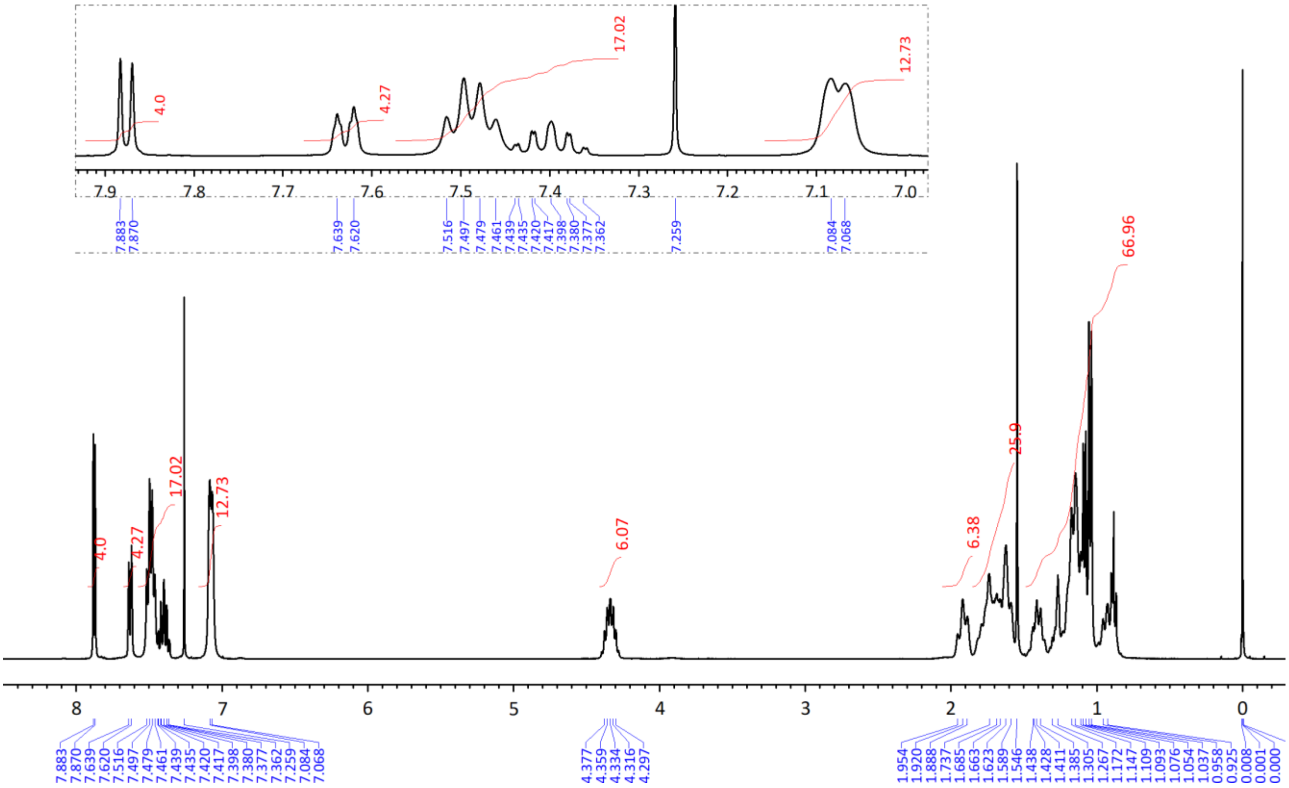

^{1}H NMR spectrum (400 MHz) of pr-**6**, measured in $CDCl_3$ at room temperature.

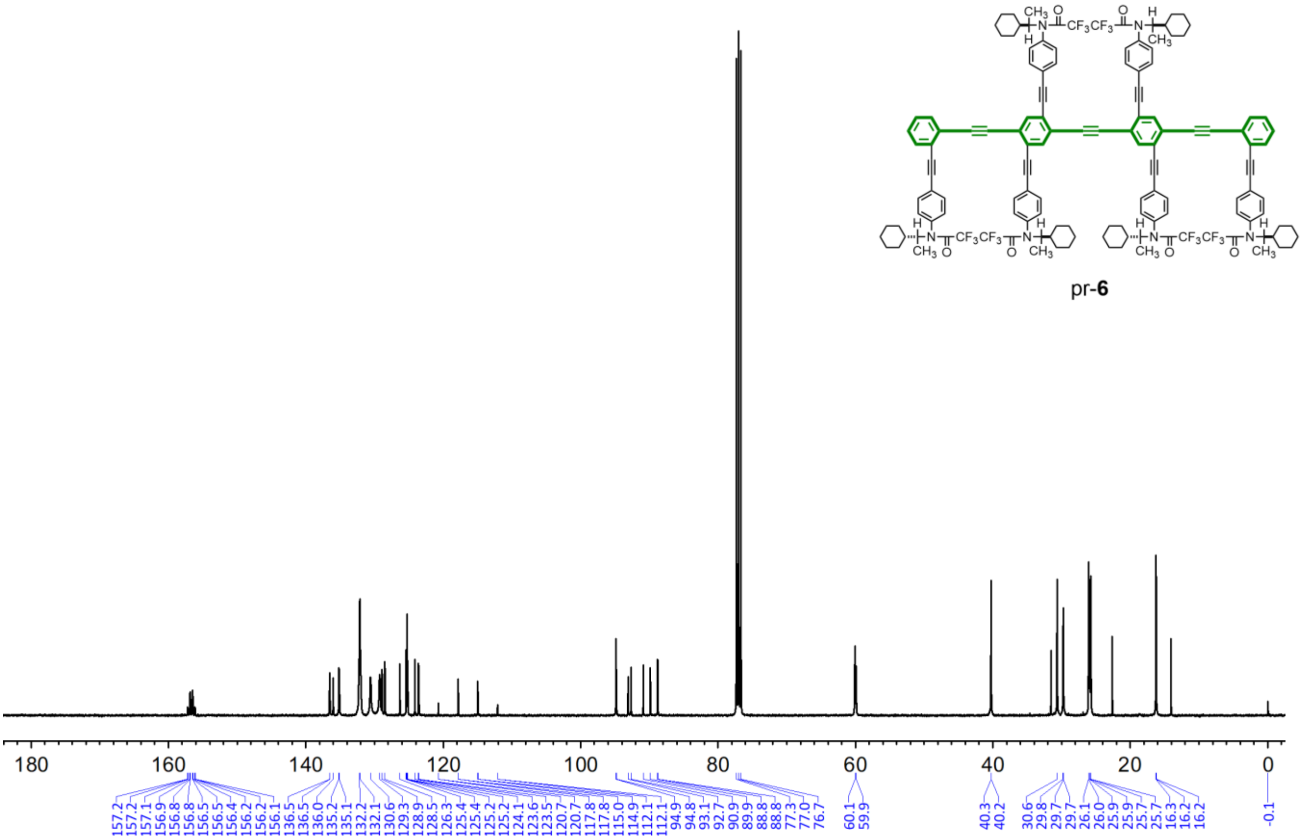

pr-**6**

^{13}C NMR spectrum (100 MHz) of pr-**6**, measured in $CDCl_3$ at 303 K.

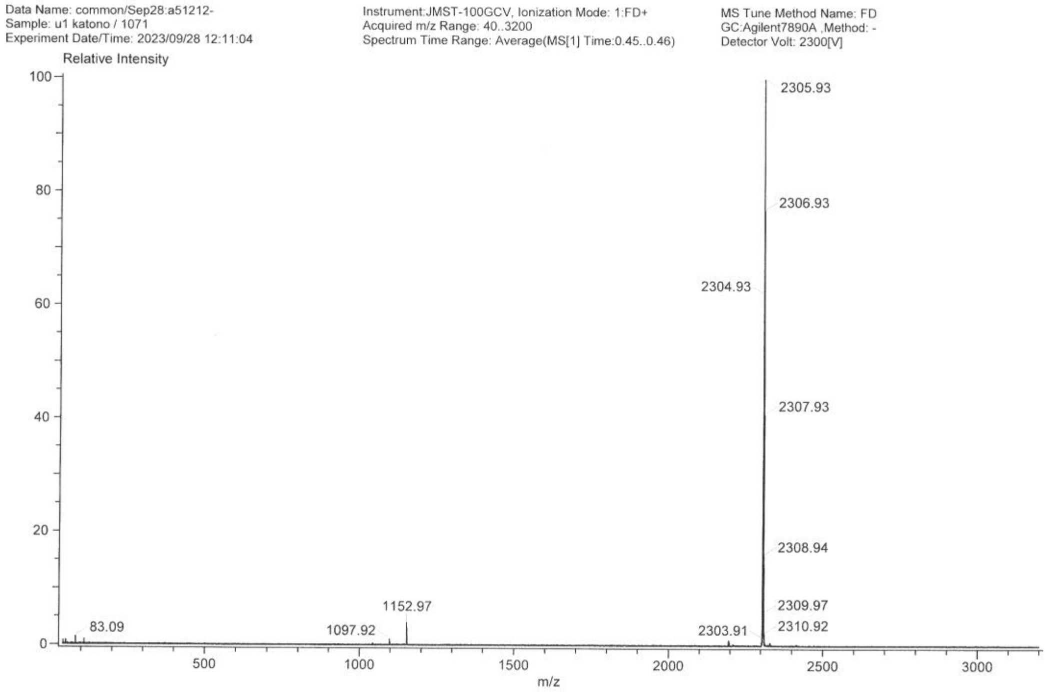

LRMS (FD) of pr-**6**.

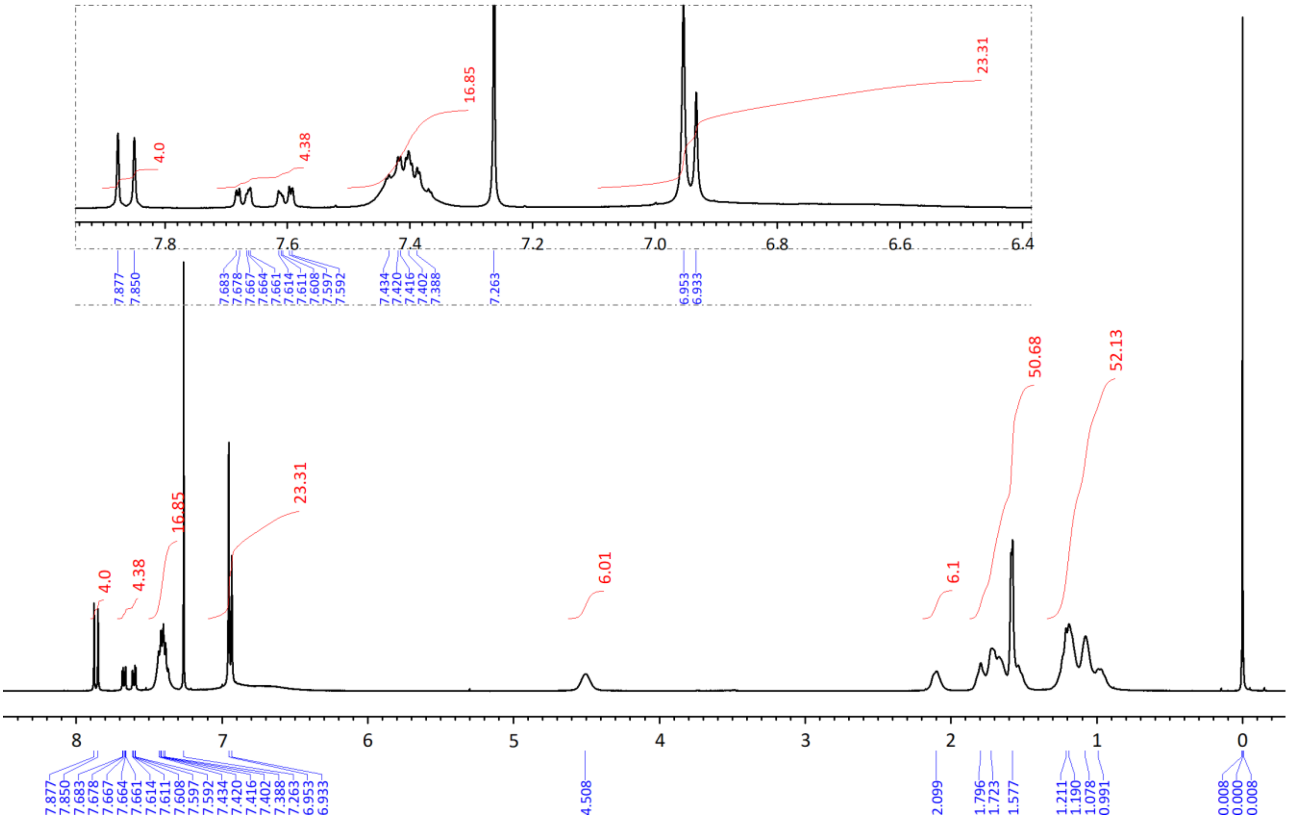

^{1}H NMR spectrum (400 MHz) of **6**, measured in $CDCl_3$ at 303 K.

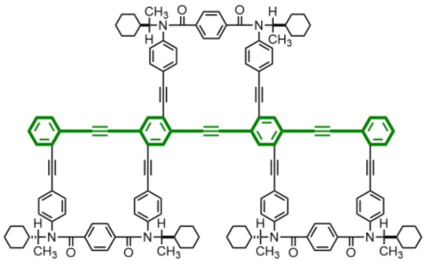

6

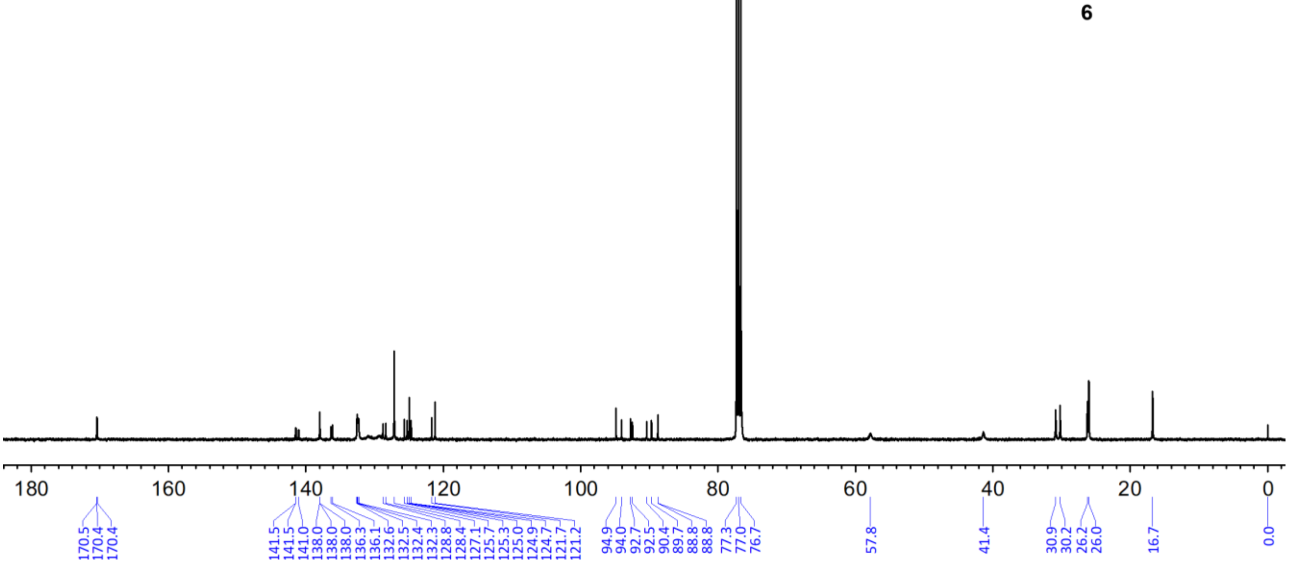

^{13}C NMR spectrum (100 MHz) of **6**, measured in $CDCl_3$ at 303 K.

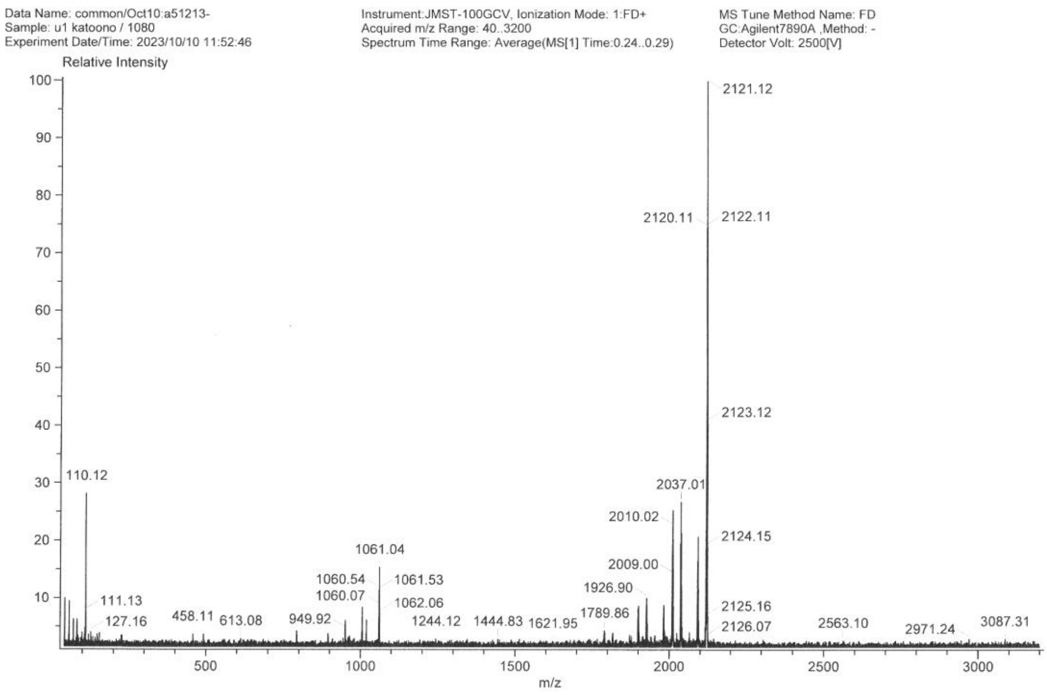
Data Name: common/Oct10:a51213-
Sample: u1 katoono / 1080
Experiment Date/Time: 2023/10/10 11:52:46
Instrument:JMST-100GCV, Ionization Mode: 1:FD+
Acquired m/z Range: 40..3200
Spectrum Time Range: Average(MS[1] Time:0.24..0.29)
MS Tune Method Name: FD
GC:Agilent7890A ,Method: -
Detector Volt: 2500[V]
Relative Intensity
2121.12
2120.11
2122.11
2123.12
110.12
2037.01
2010.02
2124.15
1061.04
2009.00
1060.54
1061.53
1926.90
1060.07
1062.06
111.13
2125.16
127.16
458.11
613.08
949.92
1244.12
1444.83
1621.95
1789.86
2126.07
2563.10
2971.24
3087.31
m/z

LRMS (FD) of **6**.

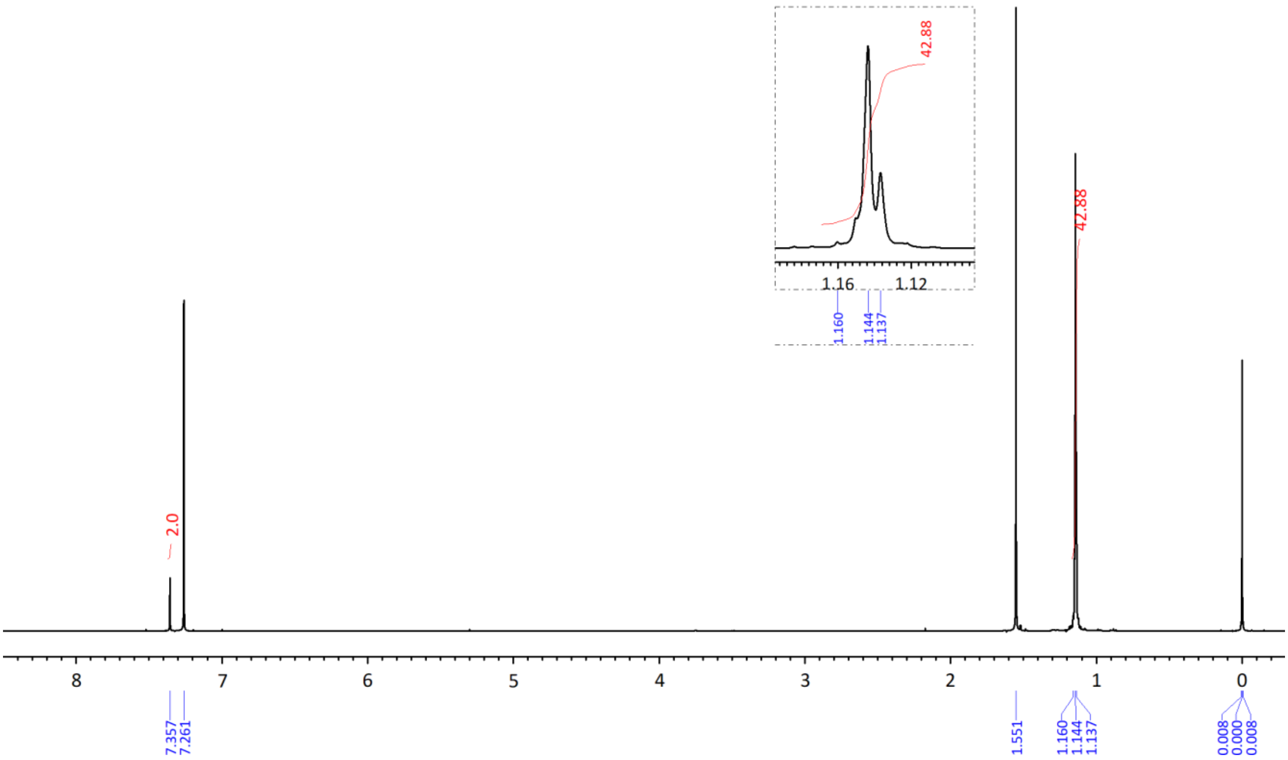

^{1}H NMR spectrum (400 MHz) of **29**, measured in $CDCl_3$ at room temperature.

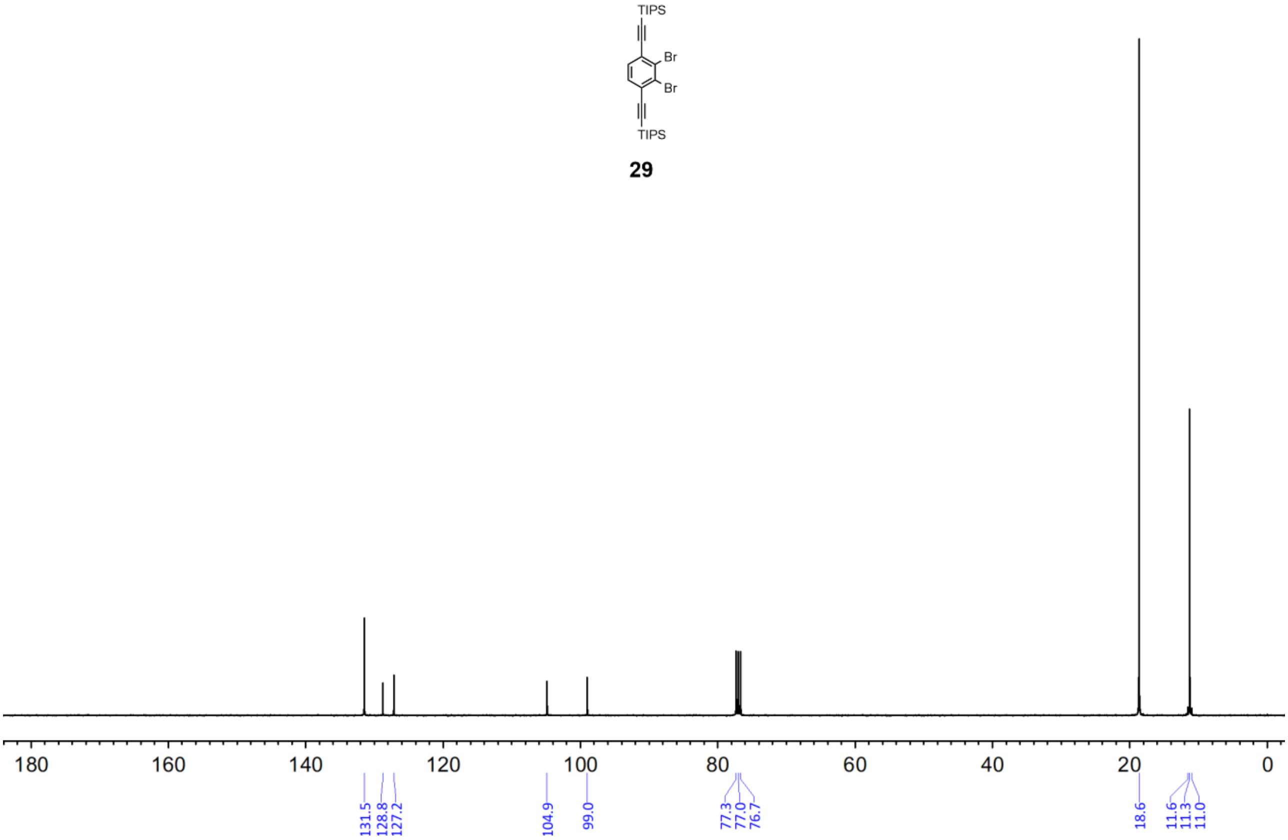

^{13}C NMR spectrum (100 MHz) of **29**, measured in $CDCl_3$ at room temperature.

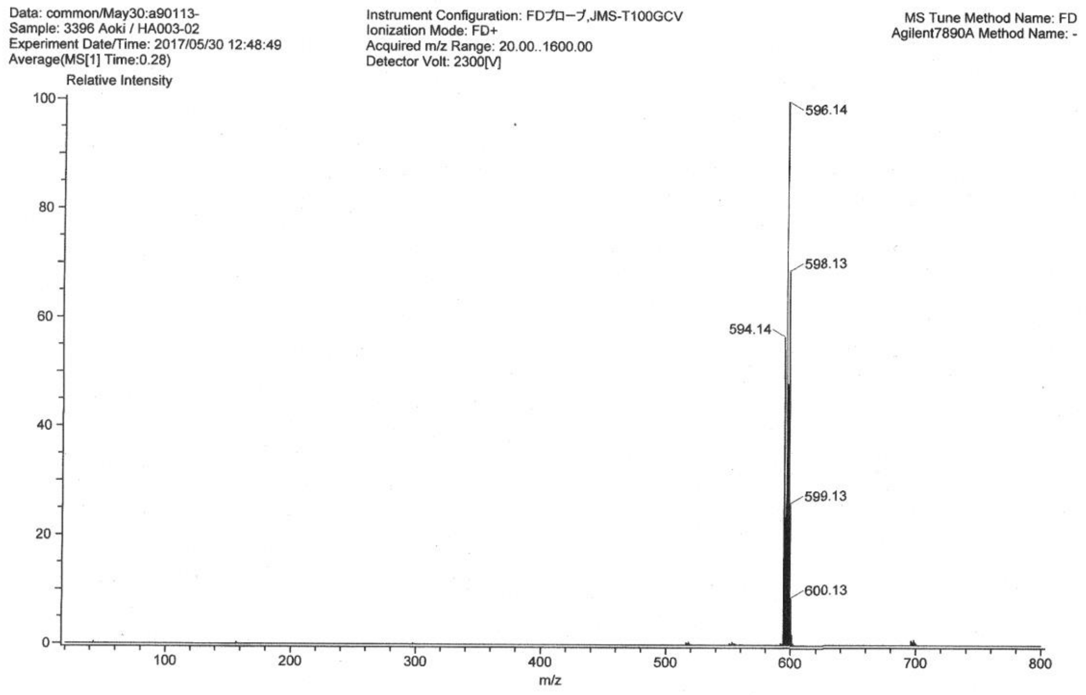

Data: common/May30:a90113-
Sample: 3396 Aoki / HA003-02
Experiment Date/Time: 2017/05/30 12:48:49
Average(MS[1] Time:0.28)
Instrument Configuration: FDプローブ,JMS-T100GCV
Ionization Mode: FD+
Acquired m/z Range: 20.00..1600.00
Detector Volt: 2300[V]
MS Tune Method Name: FD
Agilent7890A Method Name: -
Relative Intensity
596.14
598.13
594.14
599.13
600.13
m/z

LRMS (FD) of **29**.

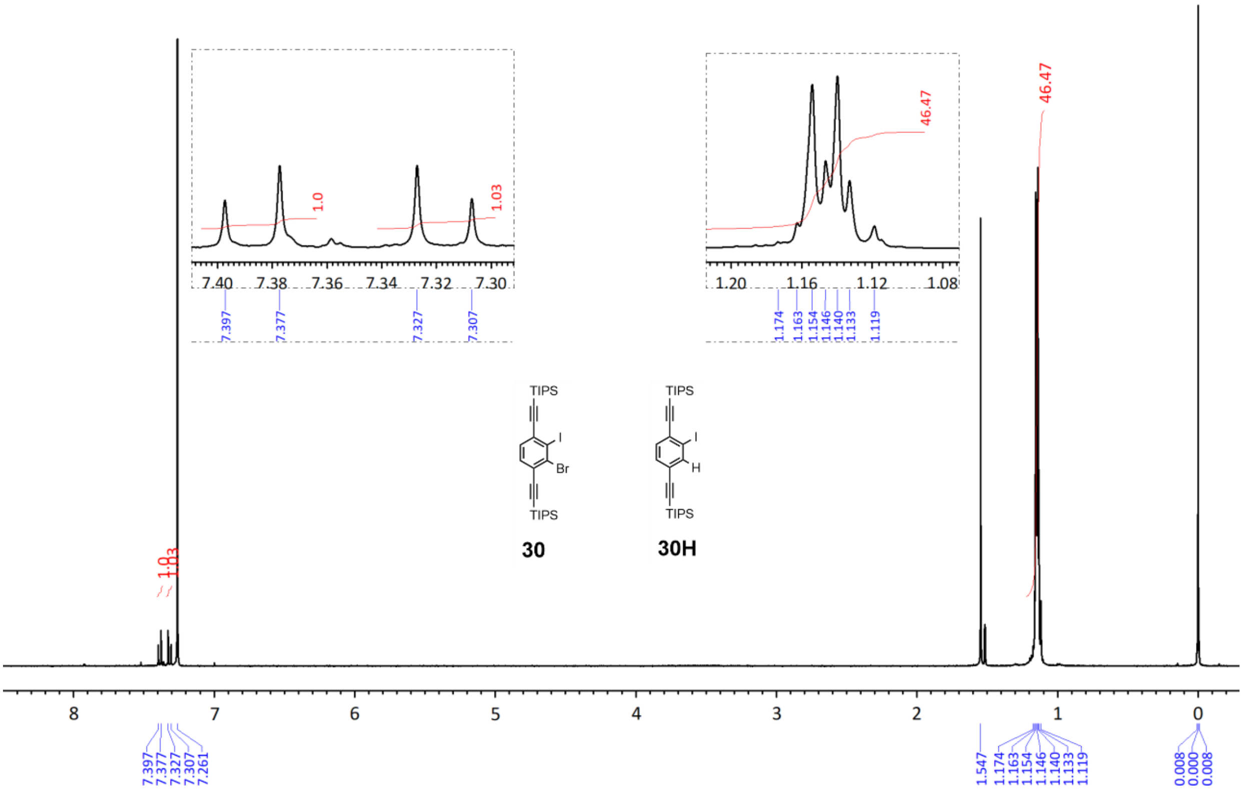

^{1}H NMR spectrum (400 MHz) of **30**/**30H**, measured in $CDCl_3$ at room temperature.

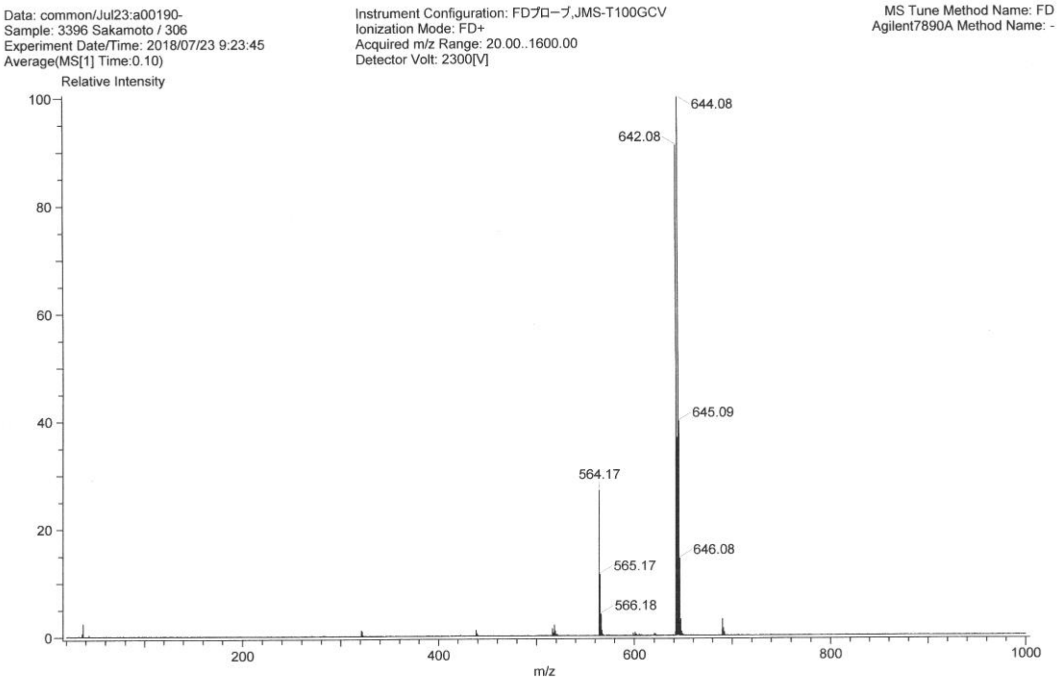

LRMS (FD) of **30**/**30H**.

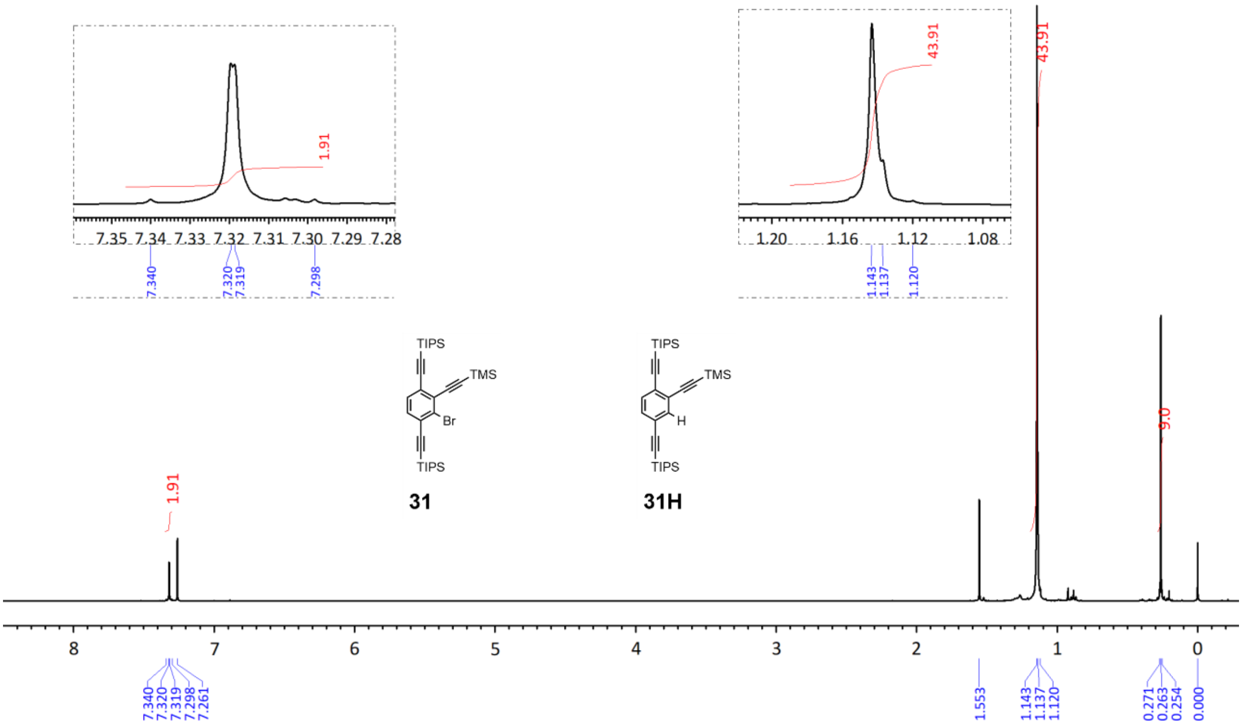

^{1}H NMR spectrum (400 MHz) of **31**/**31H**, measured in $CDCl_3$ at room temperature.

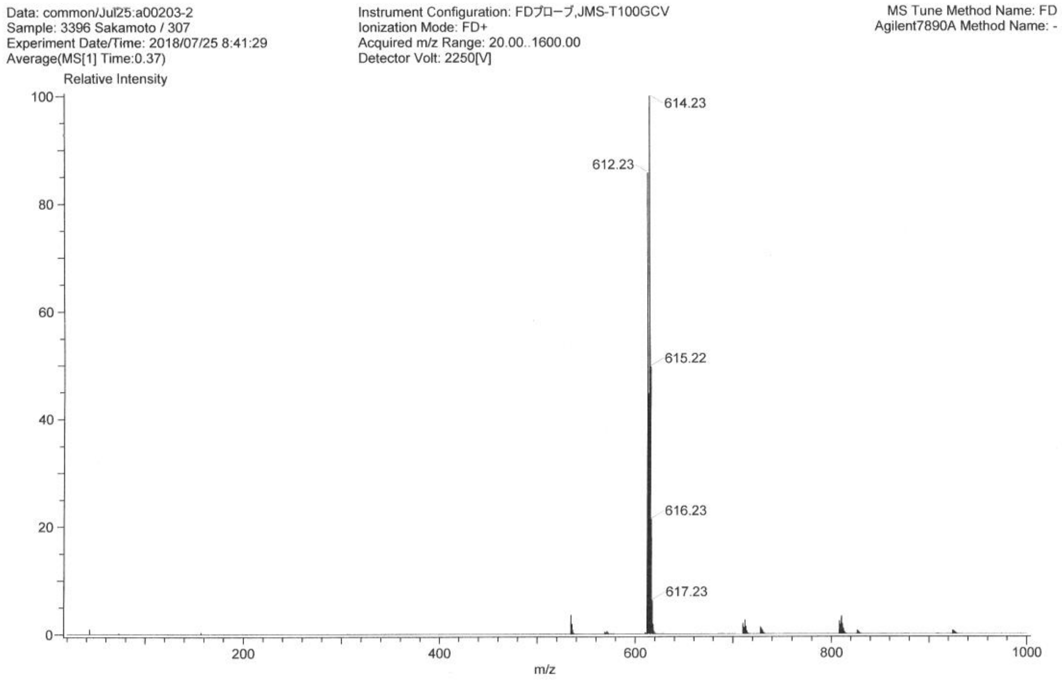

LRMS (FD) of **31**/**31H**.

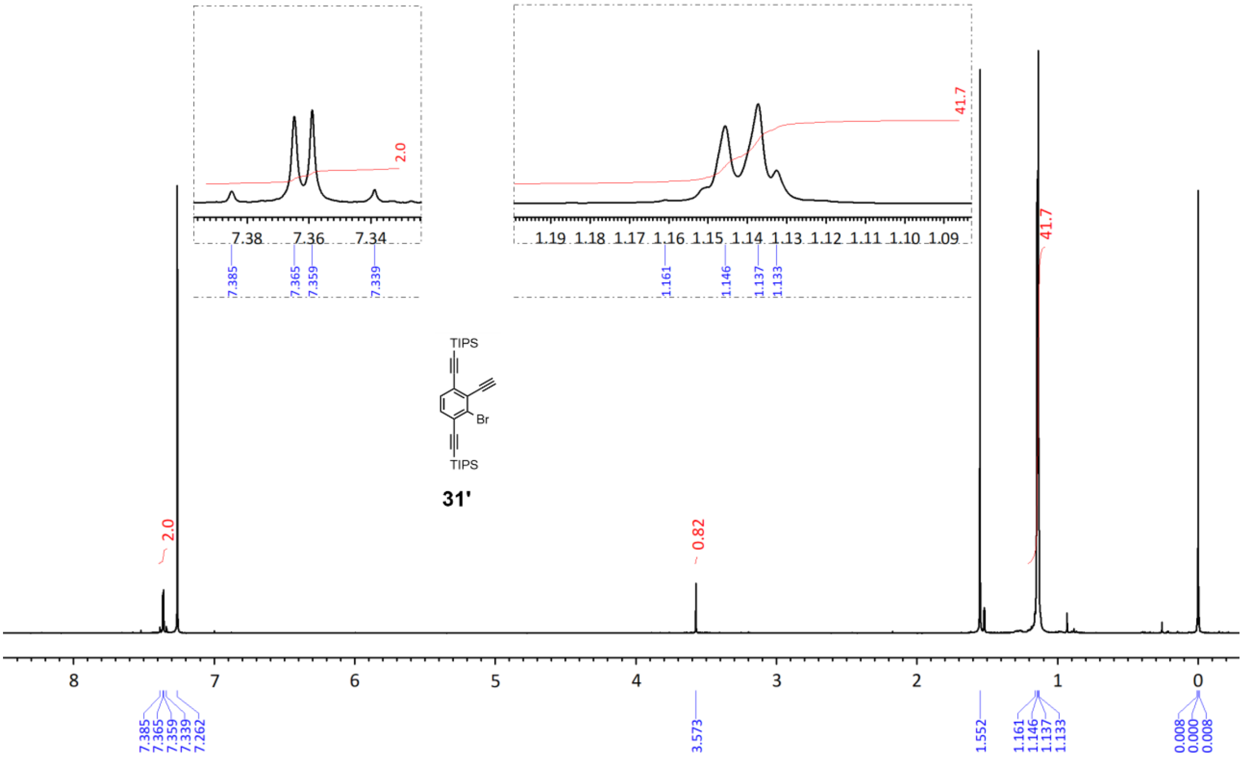

^{1}H NMR spectrum (400 MHz) of **31'**, measured in $CDCl_3$ at room temperature.

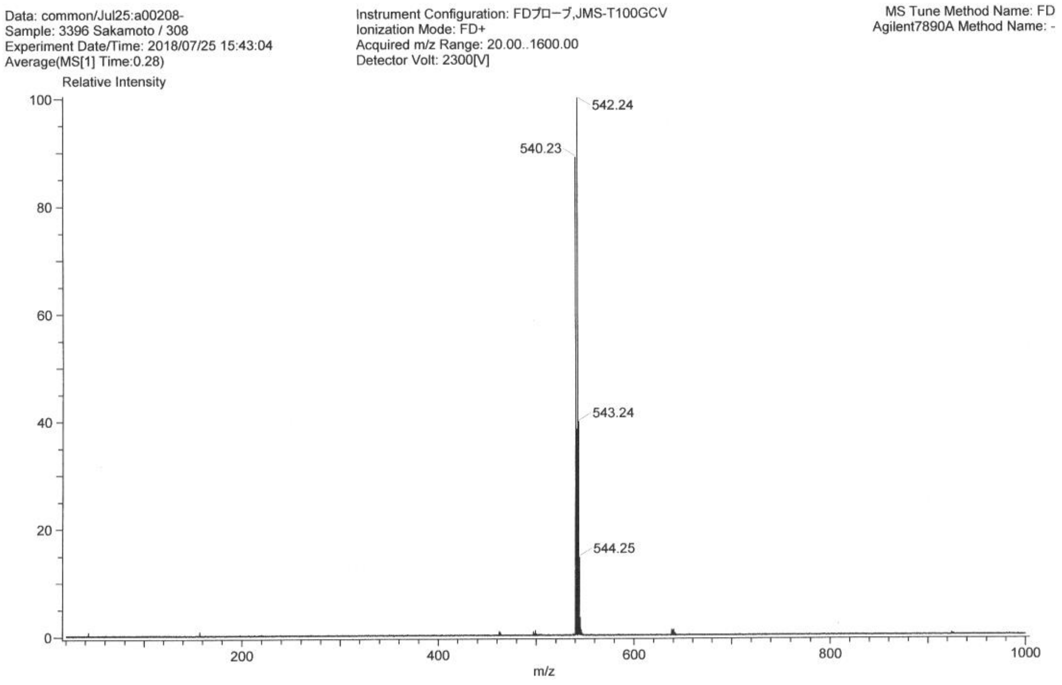

LRMS (FD) of **31'**.

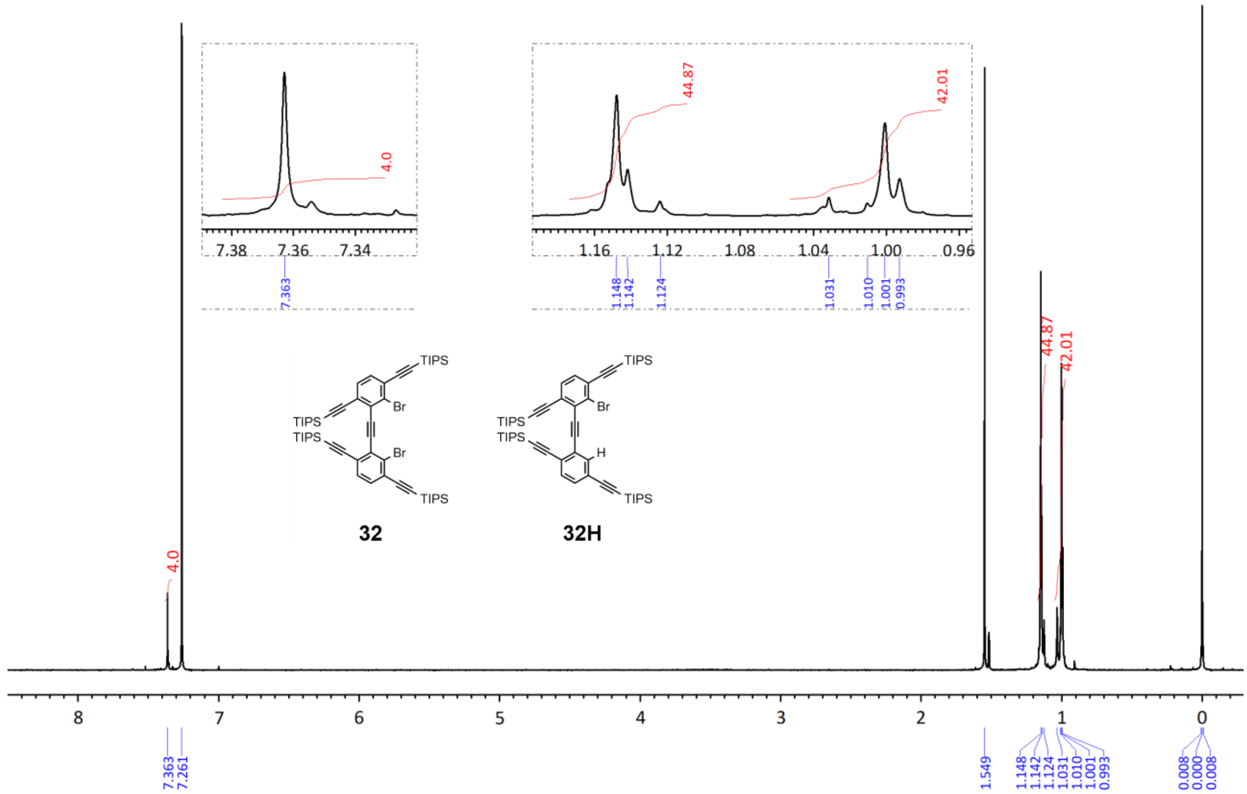

^{1}H NMR spectrum (400 MHz) of **32**/**32H**, measured in $CDCl_3$ at room temperature.

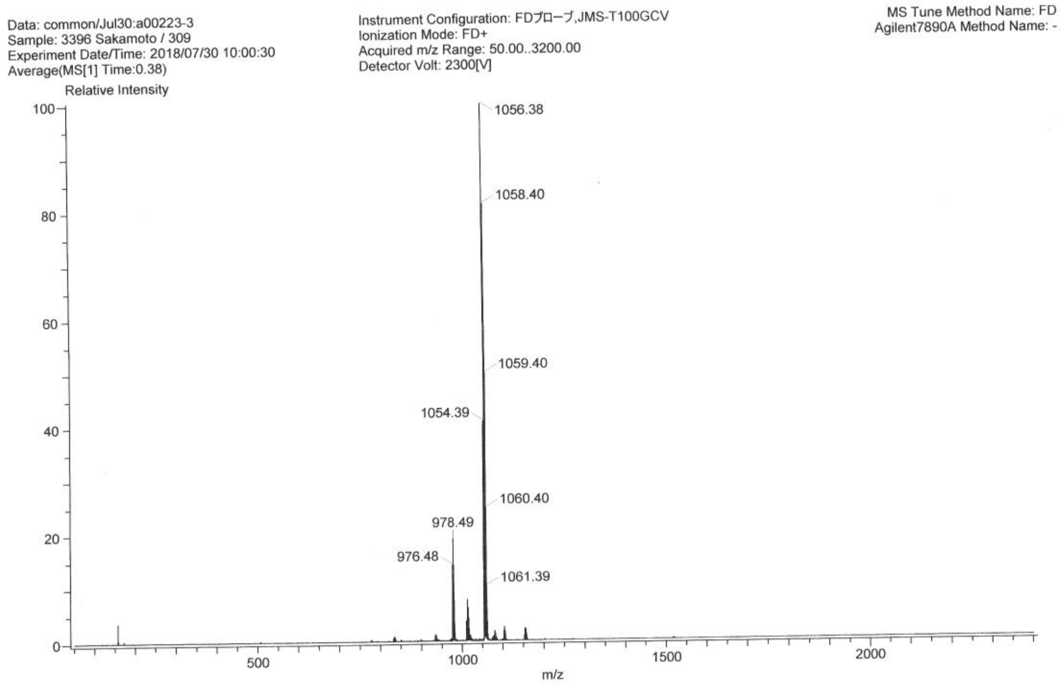

LRMS (FD) of **32**/**32H**.

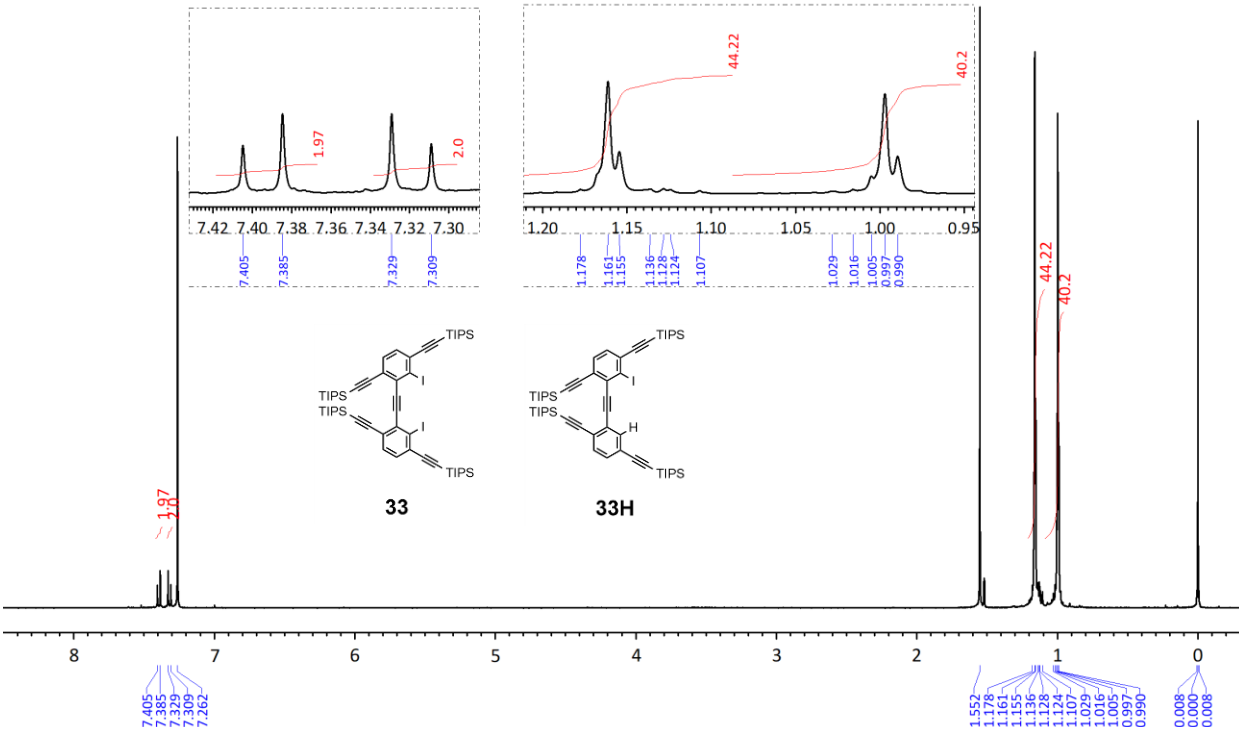

^{1}H NMR spectrum (400 MHz) of **33**/**33H**, measured in $CDCl_3$ at room temperature.

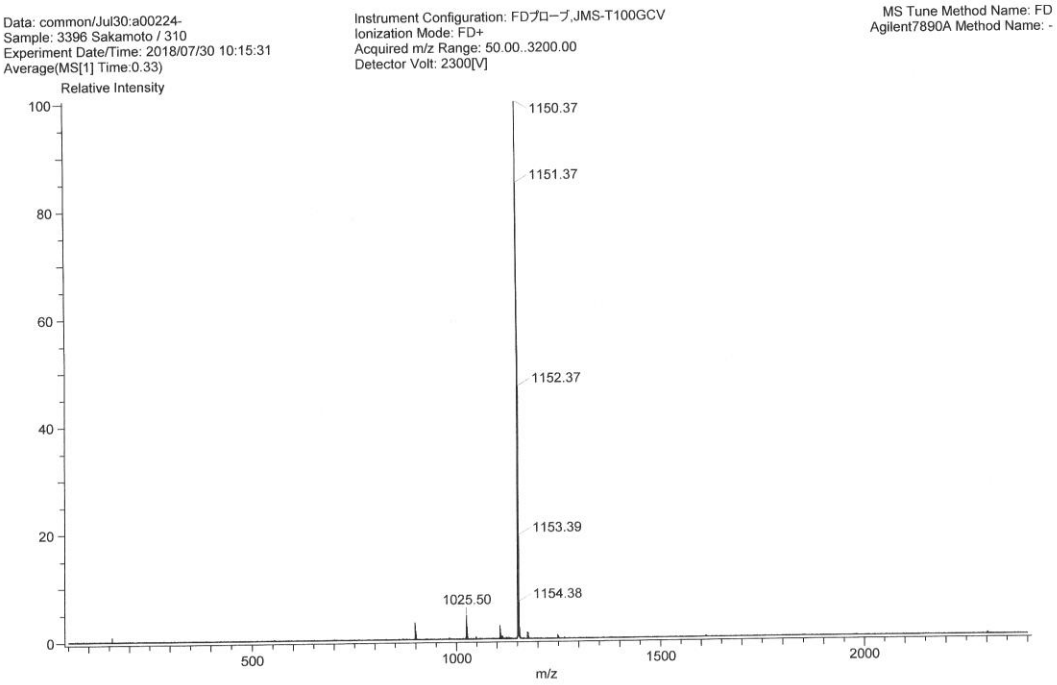

LRMS (FD) of **33**/**33H**.

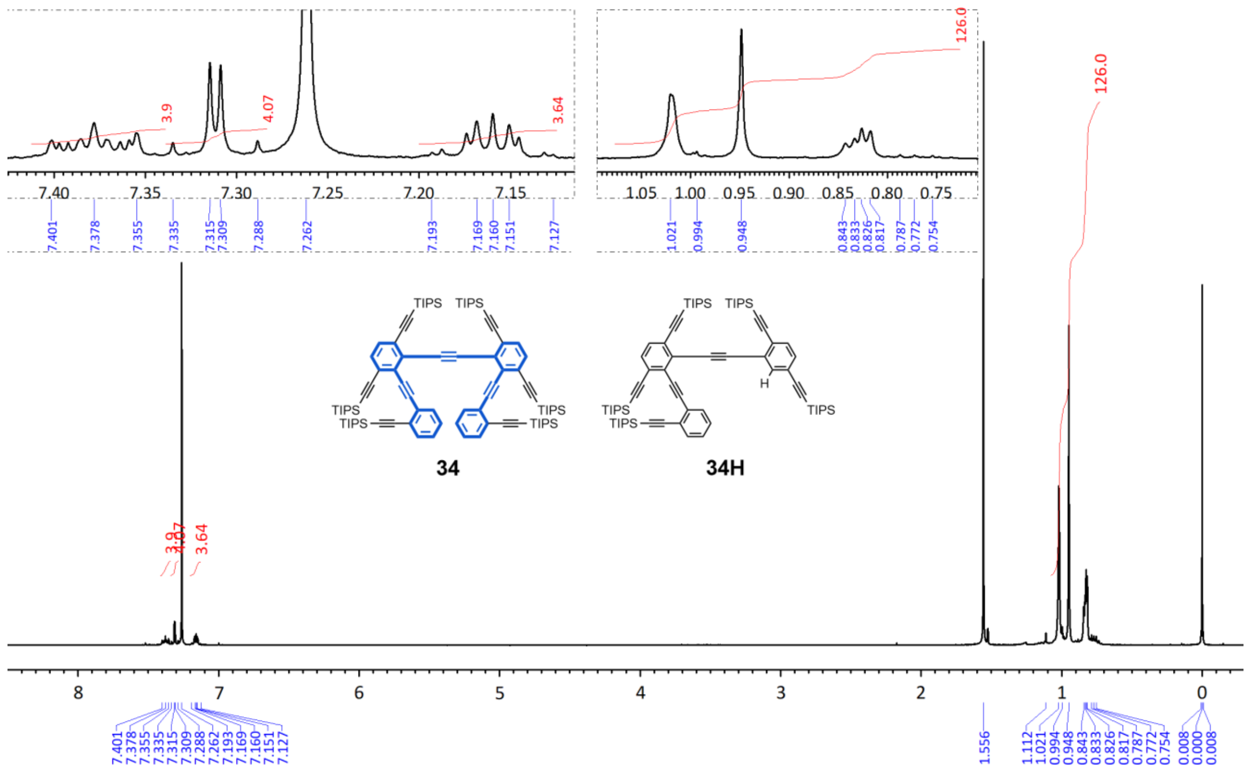

^{1}H NMR spectrum (400 MHz) of **34**/**34H**, measured in $CDCl_3$ at room temperature.

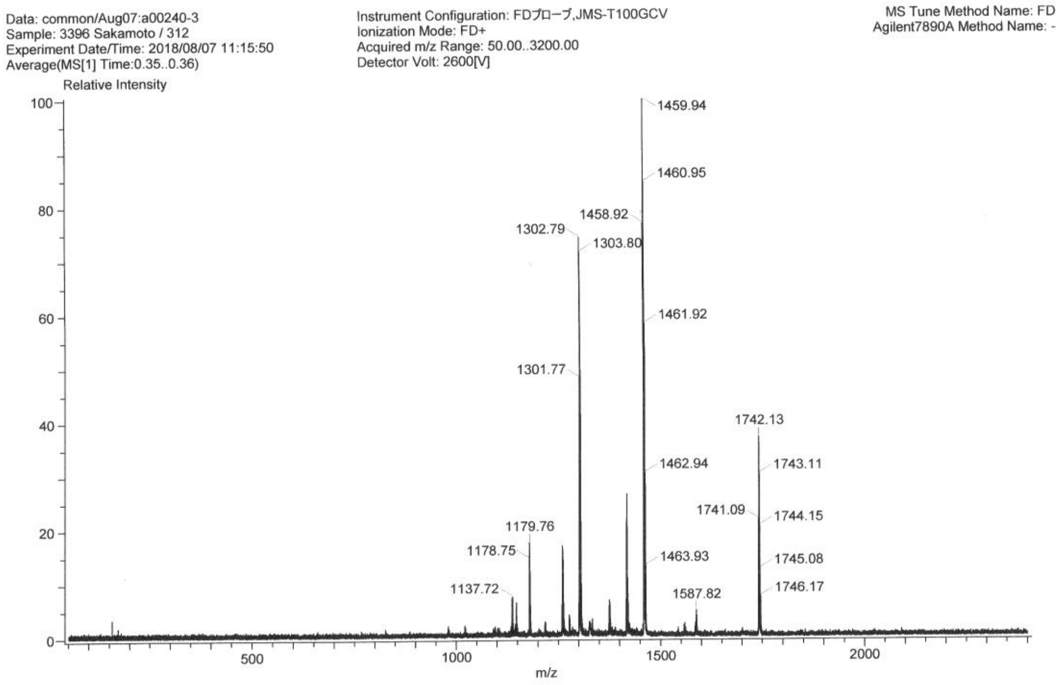

LRMS (FD) of **34**/**34H** (used to prepare **8**).

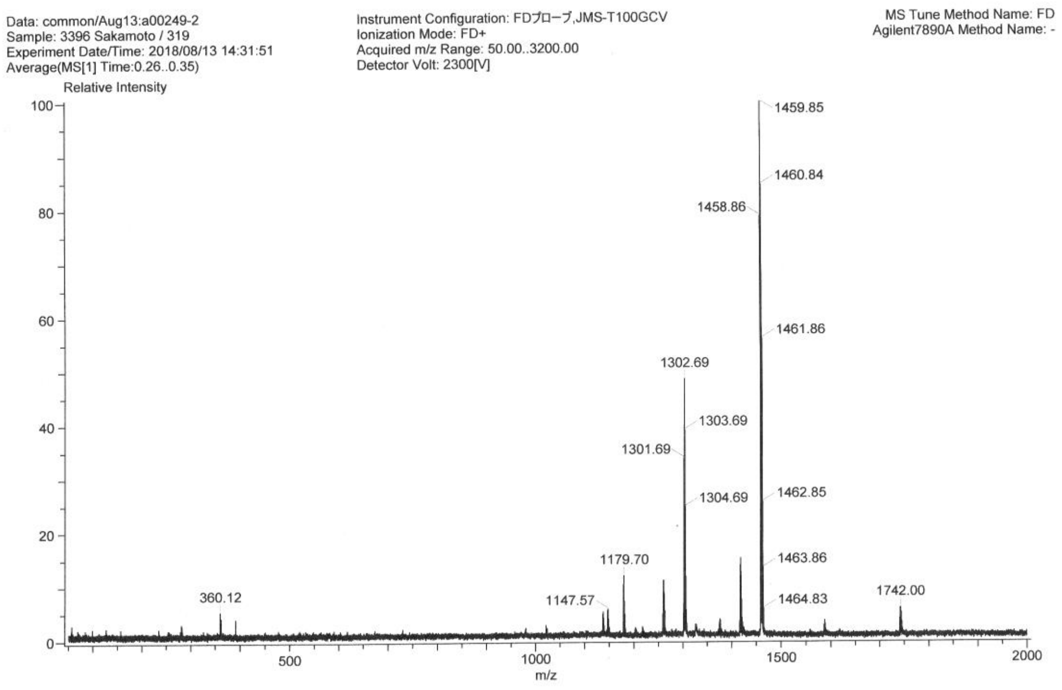

LRMS (FD) of **34**/**34H** (used to prepare **8B**).

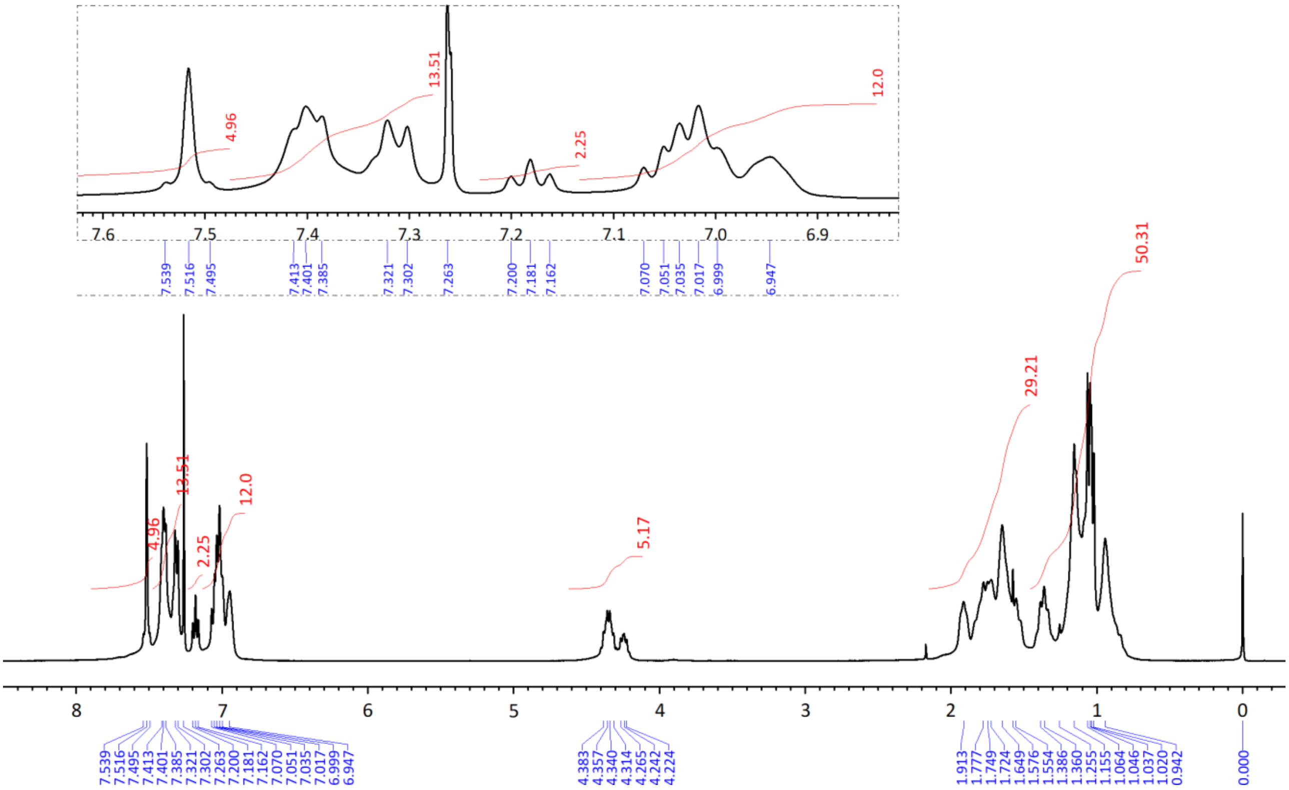

^{1}H NMR spectrum (400 MHz) of pr-**8**, measured in $CDCl_3$ at room temperature.

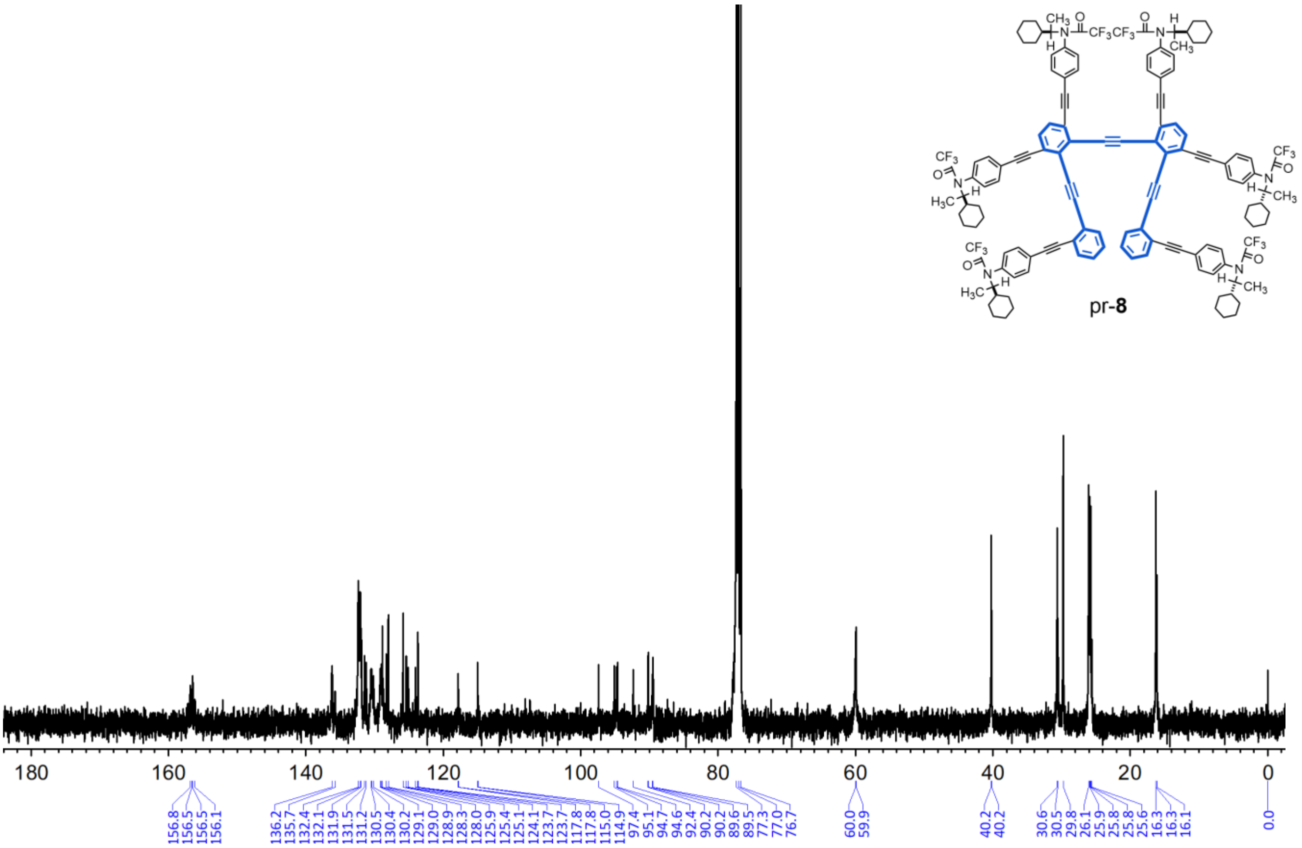

^{13}C NMR spectrum (100 MHz) of pr-**8**, measured in $CDCl_3$ at room temperature.

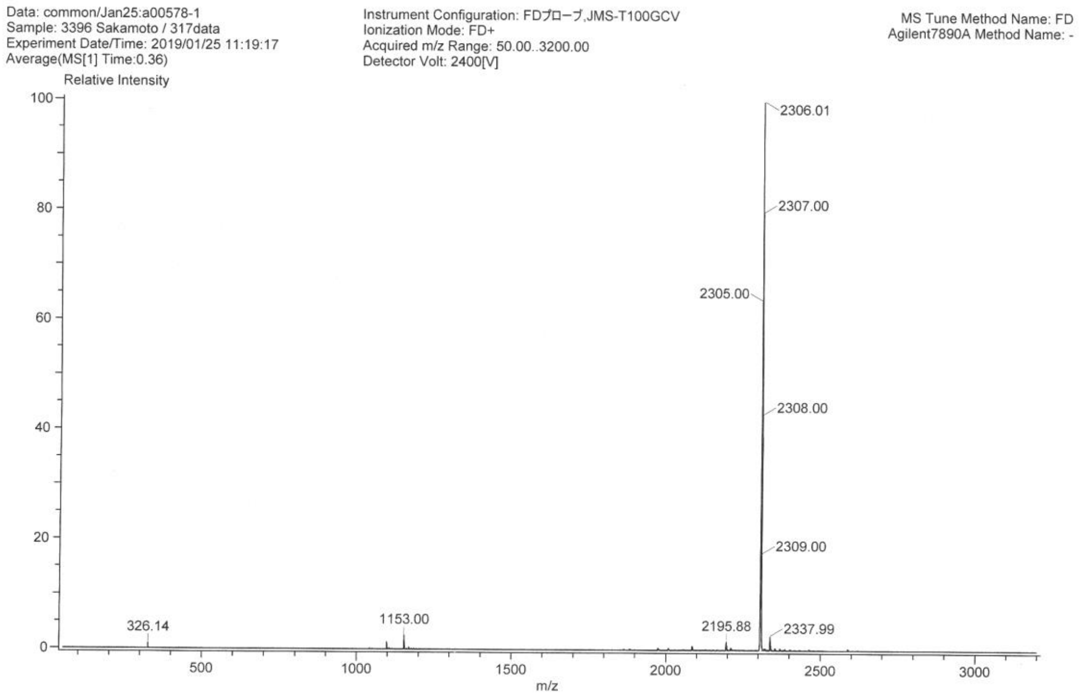

LRMS (FD) of pr-**8**.

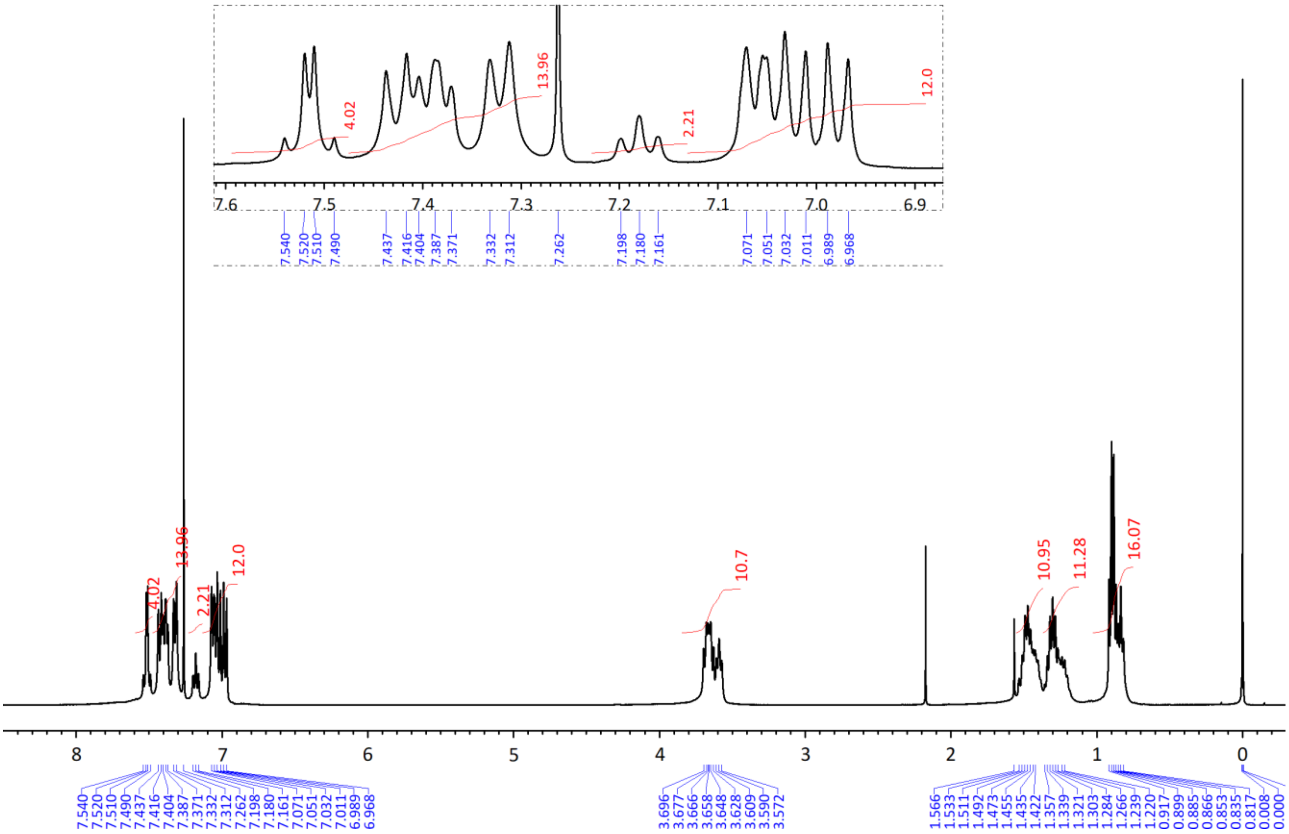

^{1}H NMR spectrum (400 MHz) of pr-**8B**, measured in $CDCl_3$ at room temperature.

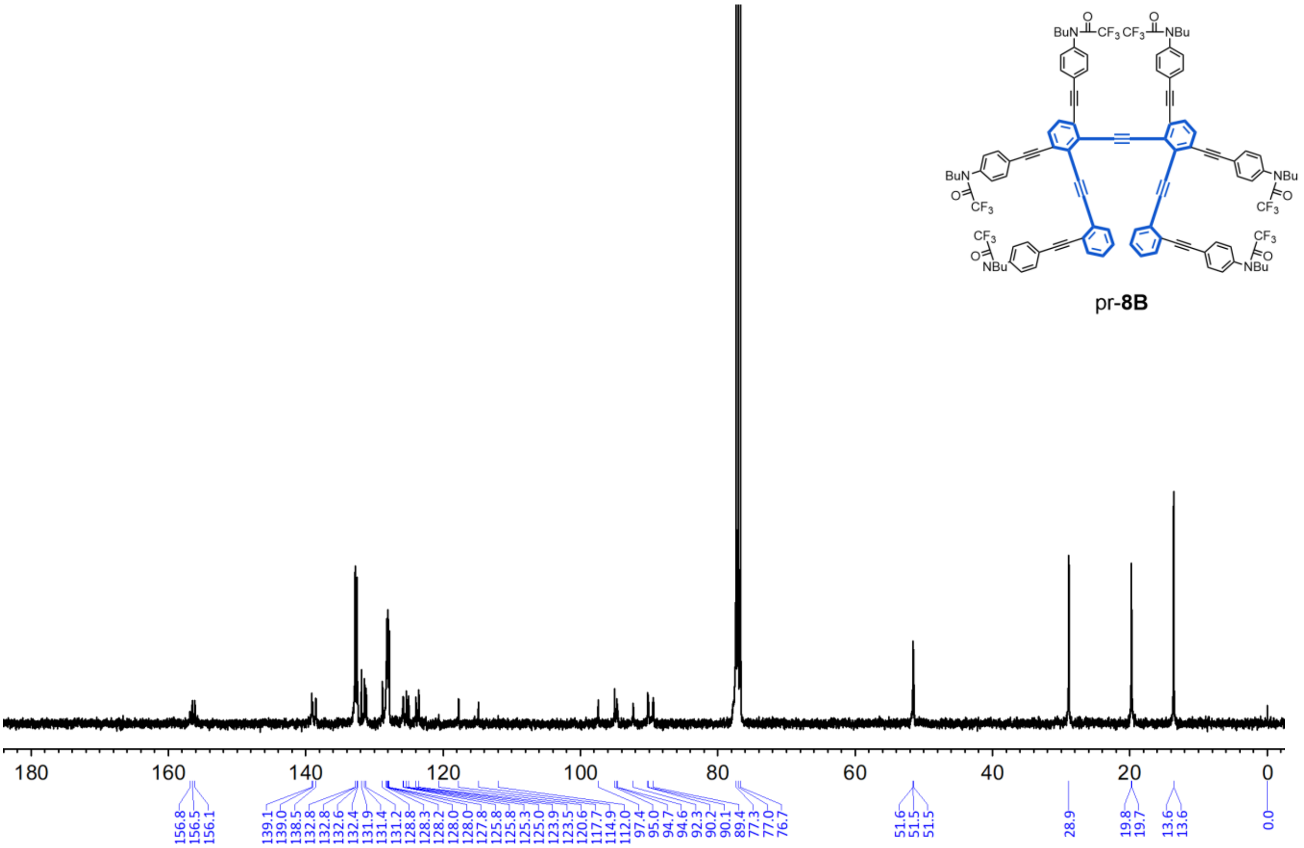

^{13}C NMR spectrum (100 MHz) of pr-**8B**, measured in $CDCl_3$ at room temperature.

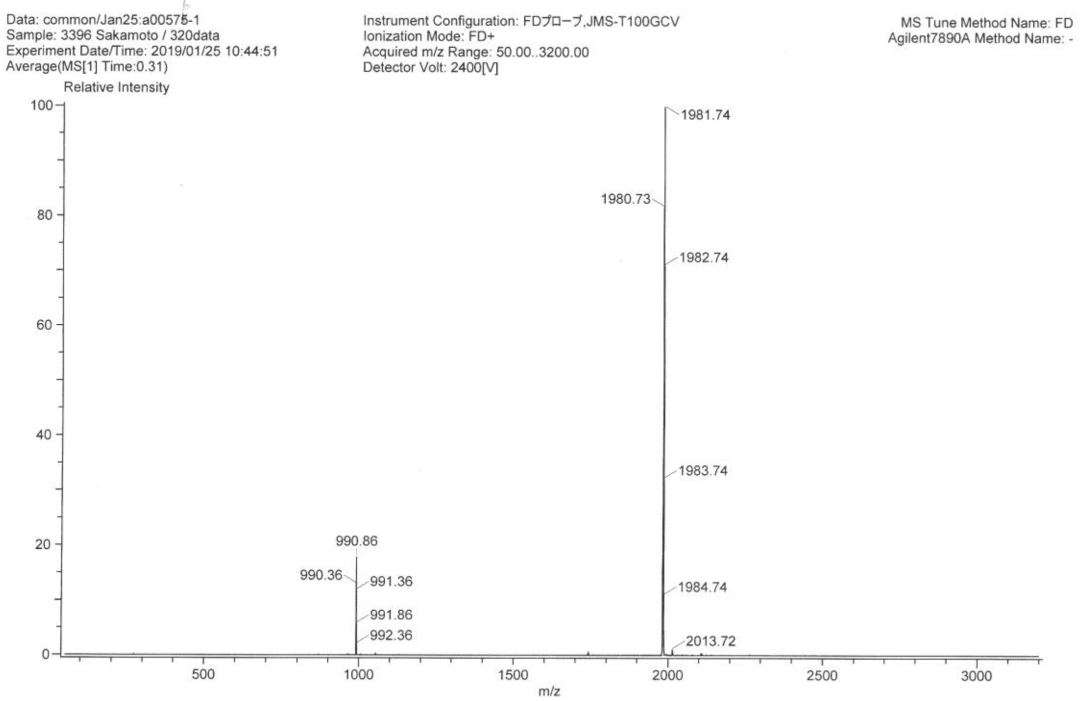

LRMS (FD) of pr-**8B**.

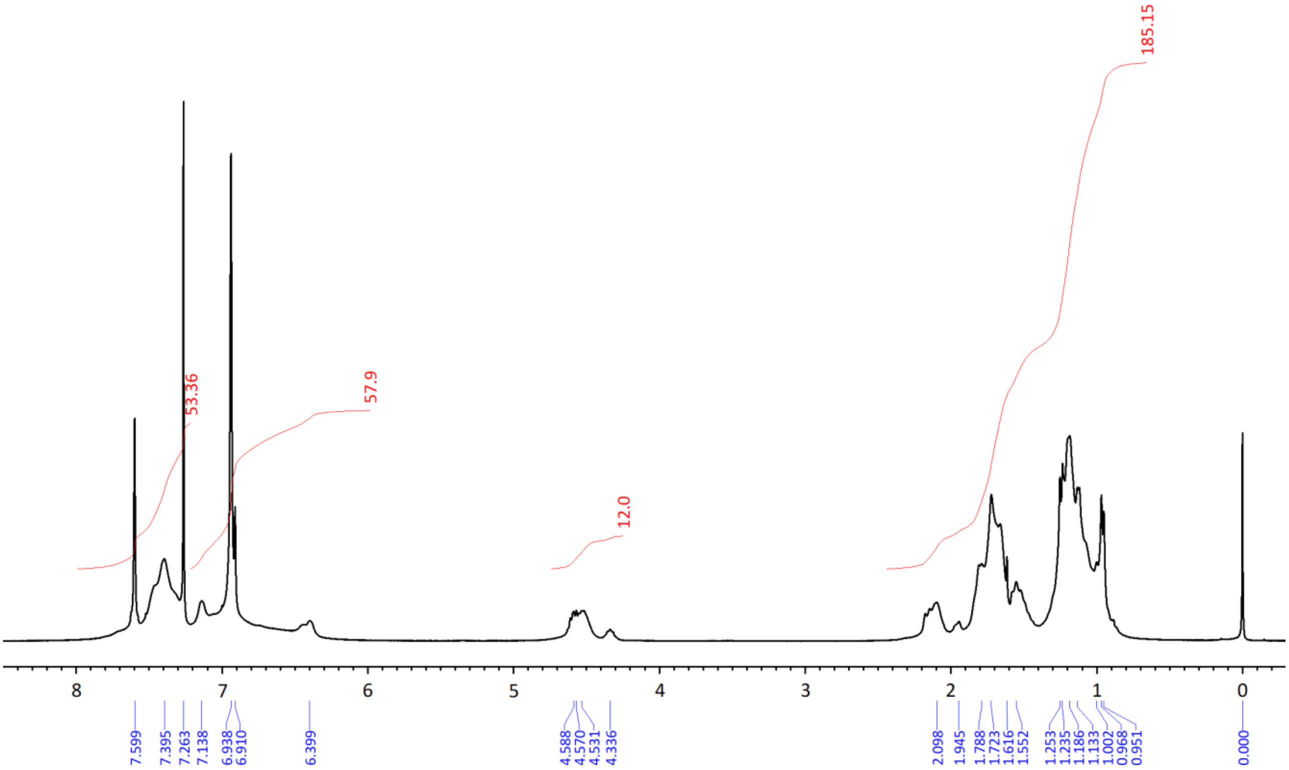

^{1}H NMR spectrum (400 MHz) of **8**, measured in $CDCl_3$ at room temperature.

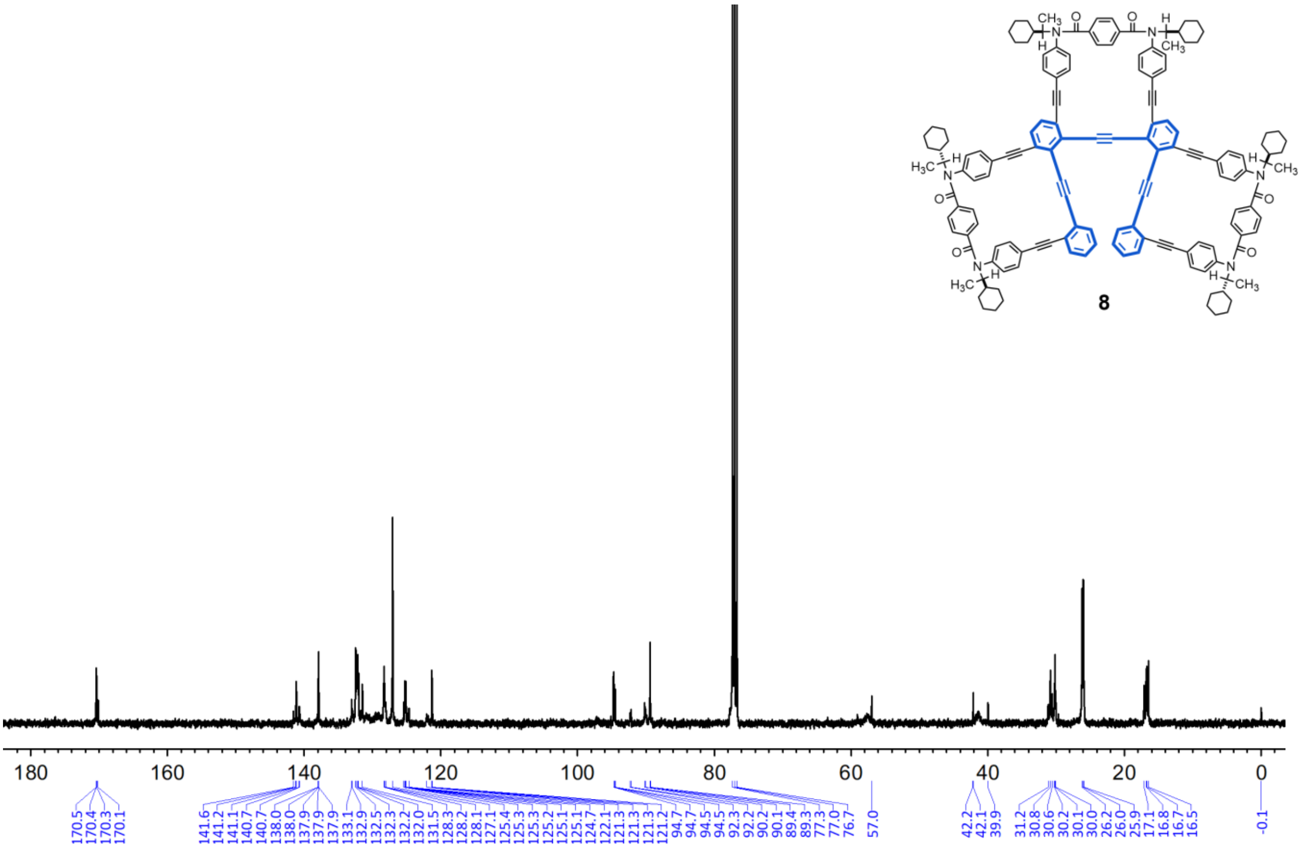

^{13}C NMR spectrum (100 MHz) of **8**, measured in $CDCl_3$ at room temperature.

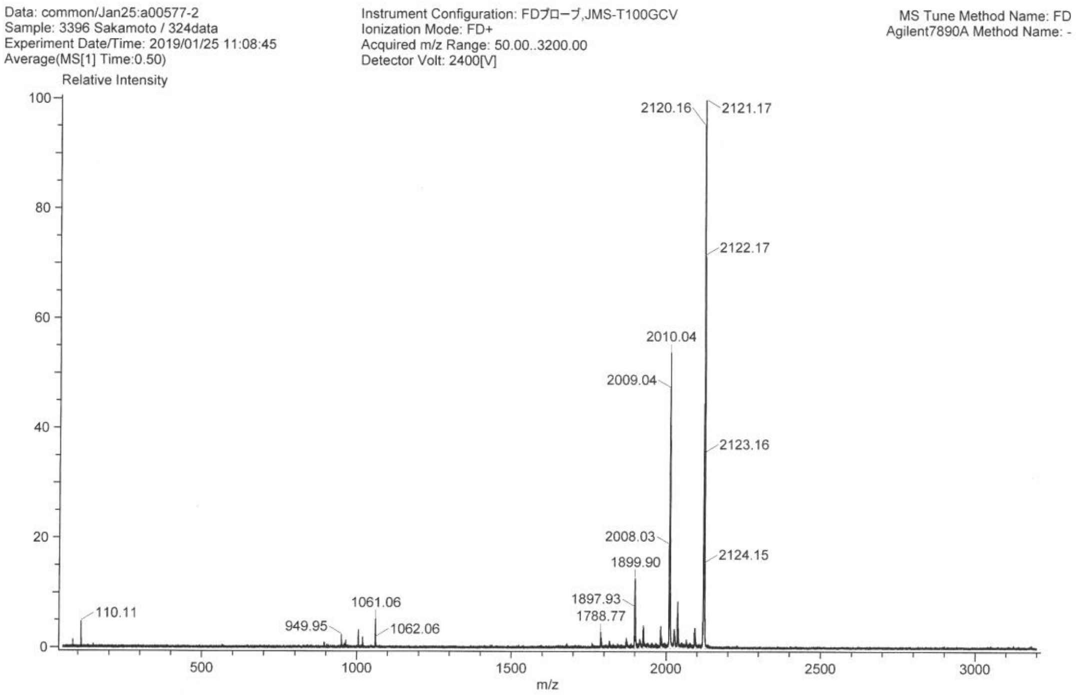
Data: common/Jan25:a00577-2
Sample: 3396 Sakamoto / 324data
Experiment Date/Time: 2019/01/25 11:08:45
Average(MS[1] Time:0.50)
Instrument Configuration: FDプローブ,JMS-T100GCV
Ionization Mode: FD+
Acquired m/z Range: 50.00..3200.00
Detector Volt: 2400[V]
MS Tune Method Name: FD
Agilent7890A Method Name: -
Relative Intensity
m/z
2120.16
2121.17
2122.17
2010.04
2009.04
2123.16
2008.03
2124.15
1899.90
1897.93
1788.77
1061.06
1062.06
949.95
110.11

LRMS (FD) of **8**.

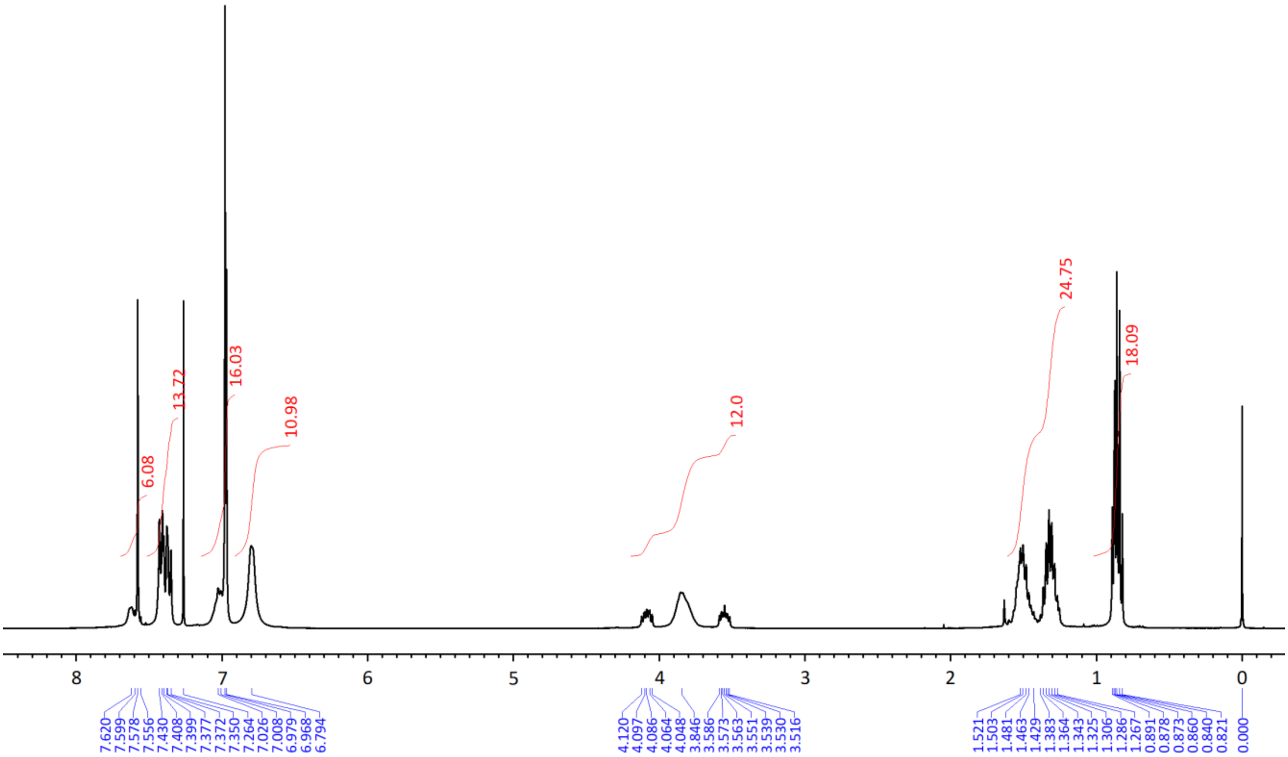

^{1}H NMR spectrum (400 MHz) of **8B**, measured in $CDCl_3$ at room temperature.

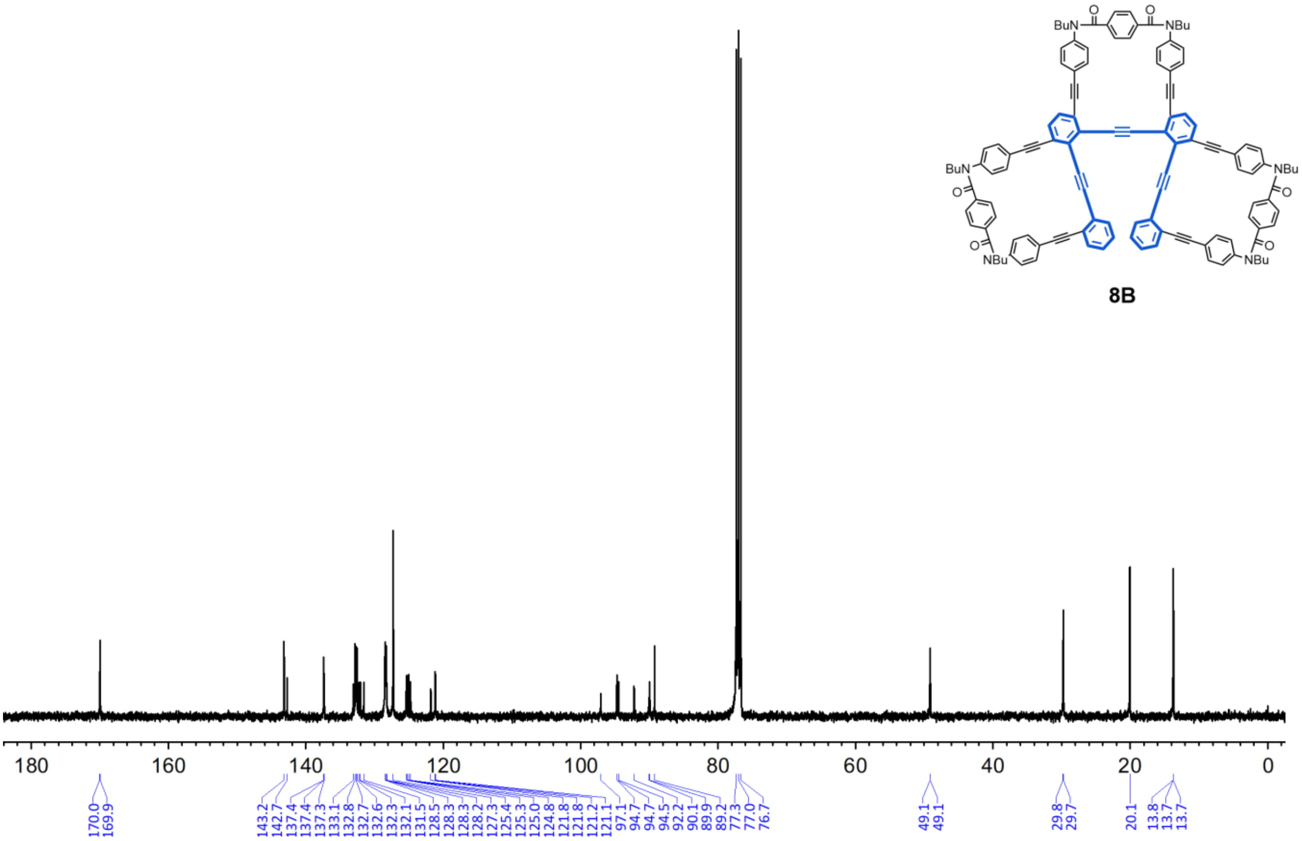

^{13}C NMR spectrum (100 MHz) of **8B**, measured in $CDCl_3$ at room temperature.

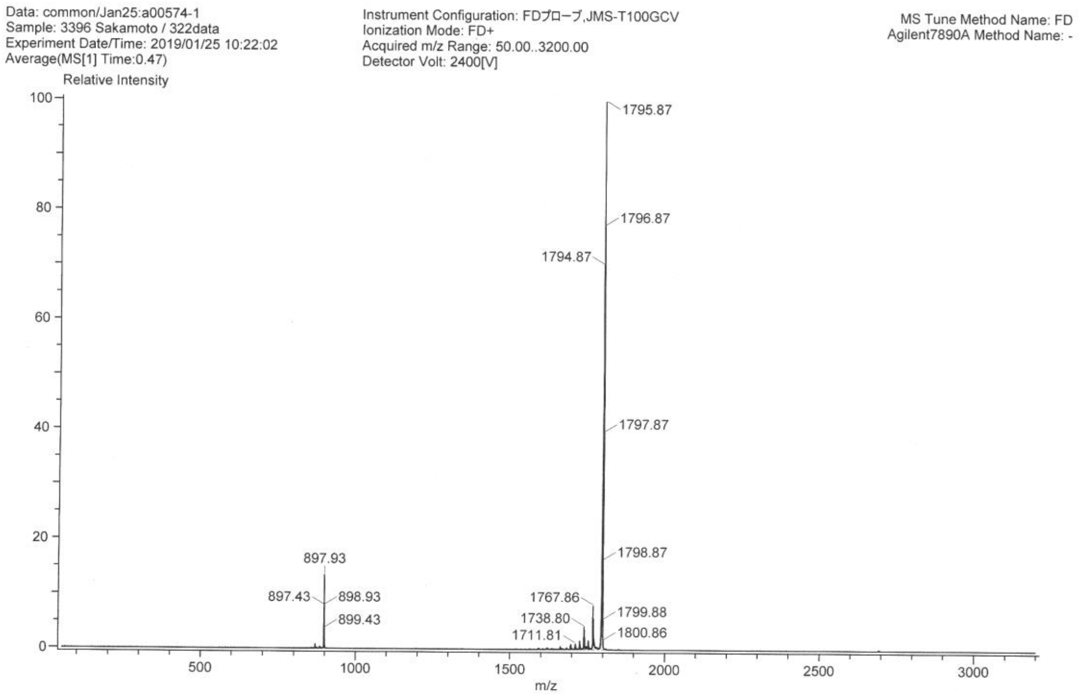
Data: common/Jan25:a00574-1
Sample: 3396 Sakamoto / 322data
Experiment Date/Time: 2019/01/25 10:22:02
Average(MS[1] Time:0.47)
Instrument Configuration: FDプローブ,JMS-T100GCV
Ionization Mode: FD+
Acquired m/z Range: 50.00..3200.00
Detector Volt: 2400[V]
MS Tune Method Name: FD
Agilent7890A Method Name: -
Relative Intensity
1795.87
1796.87
1794.87
1797.87
1798.87
897.93
897.43
898.93
899.43
1767.86
1738.80
1711.81
1799.88
1800.86
m/z

LRMS (FD) of **8B**.
